# *CALR* mutational status identifies different disease subtypes of essential thrombocythemia showing distinct expression profiles

**DOI:** 10.1038/s41408-017-0010-2

**Published:** 2017-12-08

**Authors:** Roberta Zini, Paola Guglielmelli, Daniela Pietra, Elisa Rumi, Chiara Rossi, Sebastiano Rontauroli, Elena Genovese, Tiziana Fanelli, Laura Calabresi, Elisa Bianchi, Simona Salati, Mario Cazzola, Enrico Tagliafico, Alessandro M. Vannucchi, Rossella Manfredini, on behalf of the AGIMM (AIRC Gruppo Italiano Malattie Mieloproliferative) investigators

**Affiliations:** 1 Centre for Regenerative Medicine, Department of Life Sciences, University of Modena and Reggio Emilia, Modena, Italy; 2 CRIMM, Center for Research and Innovation for Myeloproliferative Neoplasms, Department of Experimental and Clinical Medicine, AOU Careggi, University of Florence, Florence, Italy; 3 Department of Hematology Oncology, IRCCS Policlinico San Matteo Foundation & University of Pavia, Pavia, Italy; 4 Center for Genome Research, Department of Life Sciences, University of Modena and Reggio Emilia, Modena, Italy

## Abstract

Polycythemia vera (PV) and essential thrombocythemia (ET) are Philadelphia-negative myeloproliferative neoplasms (MPNs) characterized by erythrocytosis and thrombocytosis, respectively. Approximately 95% of PV and 50–70% of ET patients harbor the V617F mutation in the exon 14 of *JAK2* gene, while about 20–30% of ET patients carry CALRins5 or CALRdel52 mutations. These ET CALR-mutated subjects show higher platelet count and lower thrombotic risk compared to JAK2-mutated patients. Here, we showed that CALR-mutated and JAK2V617F-positive CD34+ cells display different gene and miRNA expression profiles. Indeed, we highlighted several pathways differentially activated between JAK2V617F- and CALR-mutated progenitors, i.e., mTOR, MAPK/PI3K, and MYC pathways. Furthermore, we unveiled that the expression of several genes involved in DNA repair, chromatin remodeling, splicing, and chromatid cohesion are decreased in CALR-mutated cells. According to the low risk of thrombosis in CALR-mutated patients, we also found the downregulation of several genes involved in thrombin signaling and platelet activation. As a whole, these data support the model that CALR-mutated ET could be considered as a distinct disease entity from JAK2V617F-positive MPNs and may provide the molecular basis supporting the different clinical features of these patients.

## Introduction

Philadelphia-negative myeloproliferative neoplasms (MPNs) are a heterogeneous group of clonal hematopoietic stem cell disorders with common molecular and clinical characteristics, and include polycythemia vera (PV), essential thrombocythemia (ET), and primary myelofibrosis (PMF)^1,2^. PV is characterized by erythrocytosis, while abnormal megakaryocytopoiesis and alterations in platelet count are distinctive features of PMF and ET^3,4^. Almost all PV patients harbor the *JAK2* mutation (mostly the V617F mutation in exon 14 and, more rarely, deletions/insertion in exon 12), while approximately 60% of PMF and ET subjects carry the JAK2V617F mutation^5,6^. In addition, mutations in exon 10 of thrombopoietin receptor (*MPL*) gene are present in about 5% of cases with ET or PMF^7^.

In 2013, somatic mutations in Calreticulin (*CALR*) gene have been reported in 50–70% of *JAK2* and *MPL*-negative MPNs^8,9^. The clinical course of CALR-mutated subjects appears to be more indolent than that of JAK2-mutated patients^10–12^. Moreover, as described by several authors, CALR-mutated ET patients show relevant differences in terms of clinical and hematologic parameters (thrombotic risk, platelet (PLT) count, white blood cell (WBC) count, hemoglobin (Hb) level) compared with JAK2V617F-positive patients^10,13,14^.

CALR is a multi-functional Ca^2+^-binding protein with chaperone activity mainly localized in the endoplasmic reticulum (ER). Somatic mutations of *CALR* frequently consist of deletions/insertions in exon 9, and generate a frameshift to a unique alternative reading frame resulting in a novel amino-acid sequence of C-terminal domain. Moreover, the mutated protein lacks the KDEL signal, which results in partial dislocation of CALR from the ER^8^. Recently, two different groups demonstrated that mutant CALR activates the JAK2 pathway through its association with MPL^15,16^ and induces thrombocytosis in a retroviral mouse model^17^. Unlike JAK2V617F transformed hematopoietic cells, PI3-K signaling seems to be less activated in CALR-mutated cells^15^, suggesting that a different activation of accessory signaling pathways could justify the differences in clinical features observed in CALR mutated patients.

In order to identify pathways deregulated by mutant CALR proteins in hematopoietic progenitors and unveil the molecular basis underlying the different clinical features of CALR-mutated ET patients, in this study we assessed gene (GEP) and miRNA expression profiles (miEP) in CD34+ cells from CALR-mutated ET patients and JAK2V617F-positive PV and ET subjects. Moreover, in order to predict deregulated mRNA–miRNA interactions involved in the disease pathogenesis and potentially affecting the clinical phenotype, we performed GEP and miEP integrative analysis.

Data analysis showed the differential activation of several signaling pathways, which could at least in part justify the distinct clinical features and outcomes of CALR-mutated and JAK2V617F-positive patients.

## Subjects and methods

### Patients and samples

Analysis was performed in a cohort of 50 patients diagnosed with PV (*n* = 26), or ET (*n* = 24) according to the World Health Organization (WHO)^2,18^. Their characteristics are reported in Table 1. PV and ET CD34+ cells were obtained from bone marrow (BM), as well as 15 controls from healthy donors (BM CTRs). All subjects provided informed written consent, and the study was performed under the local Institutional Review Board’s approved protocol. The study was conducted in accordance with the Declaration of Helsinki.

**Table 1:** Clinical characteristics of PV and ET patients

		JAK2V617F	CALR-mutated
	**PV**	ET	ET
No.	26	17	7
Sex (male/female)	10/16 (38%/62%)	5/12 (29%/71%)	6/1 (86%/14%)
Age at onset, years, median (range)	57 (40–77)	61 (37–72)	37 (26–72)
Hemoglobin, g/dL, median (range)	18.4 (14.3–25.1)	14.6 (11.9–18.1)	13.7 (10.6–16.2)
PLT count, X10^9^/L, median (range)	460 (210–902)	677 (481–1168)	979 (632–1400)
WBC count, X10^9^/L, median (range)	9.7 (6.1–89.7)	8.7 (5.9–18.2)	8.1 (5.1–10.4)
Thrombosis at diagnosis, no. (%)	7 (27)	3 (18)	0 (0)

The presence of the *JAK2*V617F mutation and the allele burden were determined via quantitative reverse transcription polymerase chain reaction (qRT-PCR), as previously described^19^. ET patients were further evaluated for *MPL* exon 10 and *CALR* exon 9 mutations using the Sanger technique^14^.

### GEP and miEP profiles and microarray data analysis

GEP and miEP were performed on the same RNA preparation using the Affymetrix technology (HG-U219 Array Strip and miRNA 2.0 array) as previously described^20^.

Differentially expressed genes (DEGs) and miRNAs (DEMs) were selected following a supervised approach by means of the analysis of variance module included in the Partek GS package. In particular, we considered as differentially expressed all the transcripts with a fold change contrast (FC) ≥1.5 and a false discovery rate (FDR) (*q*-value) < .05 in the pairwise comparisons. Downregulated genes/miRNAs in CALR-mutated samples are decreased vs. PV, JAK2V617F-positive ET and BM CTRs, as well as upregulated genes/miRNAs in CALR-mutated cells are increased vs. PV, JAK2V617F-positive ET and BM CTRs.

Functional analysis on microarray data was performed using ingenuity pathway analysis (IPA, version 01-08; Ingenuity Systems; Redwood City, CA, http://www.ingenuity.com). In order to identify regulating genes that could explain the gene expression changes in CALR-mutated ET patients, we performed IPA’s upstream regulator analysis that is able to predict the activation state of upstream transcriptional regulators based on expression of their known targets. Moreover, in order to find reliable miRNA-target interactions, we performed the integrative analysis of GEP and miEP data by using IPA’s MicroRNA Target Filter^20^.

In order to better compare GEP from PV and ET, gene set enrichment analysis (GSEA) was performed^21^. The detailed protocol for GSEA (v2.0.13; Broad Institute, Cambridge, USA) is available on the Broad Institute Gene Set Enrichment Analysis website (http://www.broad.mit.edu/gsea). The number of permutations was set to 1000.

## Results

### Gene and miRNA expression profiles of CD34+ cells from PV and ET patients according to ***CALR*** and ***JAK2*** mutations

We performed gene and miRNA expression profiling in CD34+ cells from 26 PV and 24 ET patients. The clinical features of the 50 MPN patients enrolled in the study are shown in Table 1. Among the 24 ET patients, CALR mutations were detected in 7 (29%), JAK2V617F in 17 (71%), whereas all PV patients (100%) were JAK2V617F positive. As a control, 15 BM samples from healthy donors were included.

**Fig. 1:**
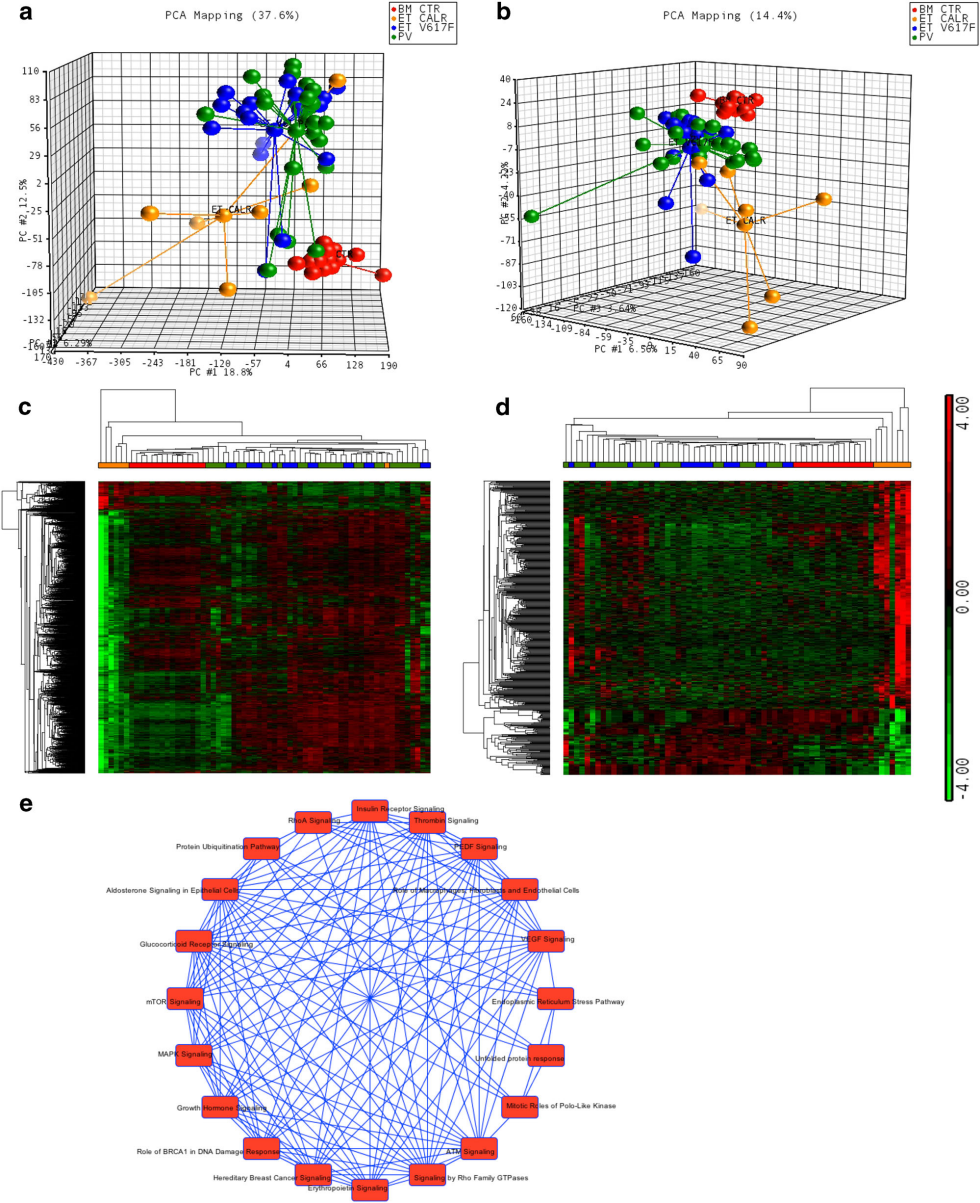
**Gene and miRNA expression profiles of PV and ET CD34+ cells according to** ***JAK2*** **and** ***CALR*** **mutational status.** **a**,** b** Principal component analysis (PCA) on gene and miRNA expression microarray data. The unsupervised PCA analysis on global gene expression **a** and miRNA expression data **b** were computed using Partek GS, version 6.6. BM control samples are shown as red spheres; PV samples are shown in green; JAK2V617F-positive ET samples are shown in blue; CALR-mutated ET samples are shown in orange; **c**,** d** The heat maps were computed on the gene list of DEG **c** and DEM **d** (provided in Supplementary Tables 1 and 2, respectively) using the clustering algorithm included in the Partek GS package by means of euclidean distance and average linkage. Gene coloring is based on normalized signals, as shown on the left; green indicates reduced expression, red increased expression in the pairwise comparison CALR-mutated vs. JAK2V617F-positive ET. CALR-mutated ET groups clustered separately in the dendrogram shown at the top of the heat map. **e** Ingenuity pathway analysis (IPA) of modulated genes in CALR-mutated ET vs. JAK2V617F-positive ET. The graph shows the most highly represented canonical pathways in the list of DEG and their overlapping connections

The unsupervised analysis of GEP and miEP data set through the principal component analysis (PCA, Fig. 1a) shows that that PV and ET samples cluster together and are clearly separated from BM CTRs (Supplementary Fig. 1A, B). According to PCA results, GSEA shows that the most of DEGs in PV or ET samples vs. BM CTRs is shared between the two diseases (Supplementary Fig. 1C, F). Therefore, neither GEP nor miEP could distinguish between PV and ET CD34+ samples.

Noteworthy, PCA performed on both GEP and miEP data (Fig. 1a, b) shows that JAK2V617F-positive ET and PV samples are grouped together, while the CALR-mutated samples are clearly separated from PV, JAK2V617F-positive ET and BM CTR clusters.

Therefore, in order to study the differences in gene expression according to different driver mutations, we compared GEP and miEP of CD34+ cells from CALR-mutated vs. JAK2V617F-positive ET and PV patients. We found 2040 coding transcripts and 488 miRNAs differentially expressed between CALR-mutated vs. JAK2V617F-positive ET CD34+ cells, which can distinguish ET patients based on their mutational status (Fig. 1c, d, Supplementary Tables 1 and 2), while we were unable to identify any modulated genes in the pairwise comparison between PV and JAK2V617F-positive ET samples. All microarray data were submitted to the Gene Expression Omnibus repository (GEO; http://www.ncbi.nlm.nih.gov/geo, series GSE103176 and GSE53482).

### ET CALR-mutated CD34+ cells show a different expression pattern of signal transducers involved in mTOR, MAPK, and PI3K pathways

**Fig. 2:**
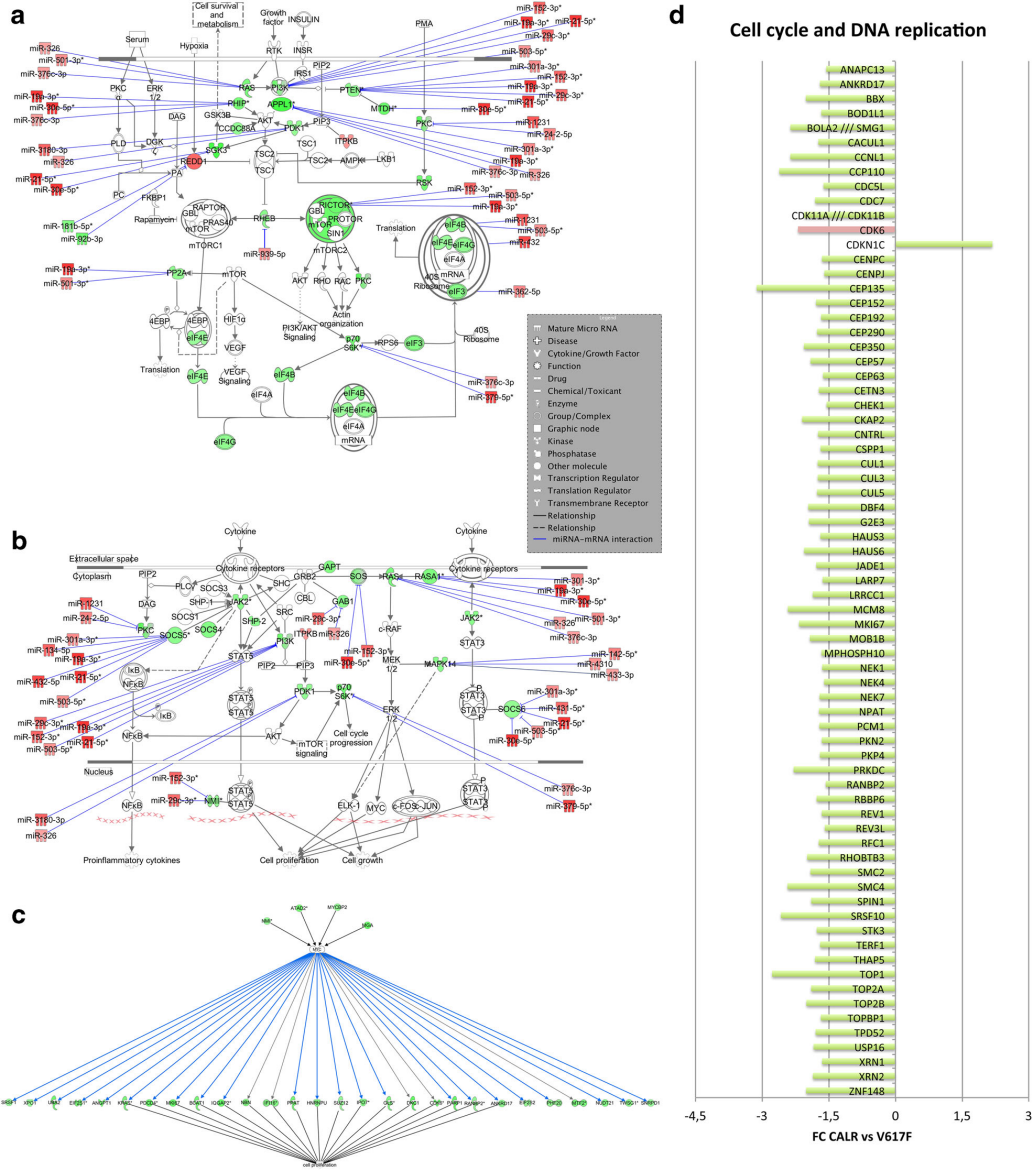
**IPA canonical pathways and upstream regulator analysis of DEG in CALR-mutated vs. JAK2V617F-positive ET.** **a**,** b** IPA canonical pathways more represented in the list of DEG **a** mTOR signaling; **b** MAPK/PI3K signaling. The networks also show the predicted mRNA–miRNA interactions identified by IPA’s miRNA Target Filter. Green and red colors indicate genes/miRNA down- and upregulated, respectively, in the pairwise comparison CALR-mutated vs. JAK2V617F-positive ET **c** IPA upstream regulator analysis indicated the inhibition of MYC activity predicted by the decreased expression of its targets. Green colors indicate genes downregulated in the pairwise comparison CALR-mutated vs. JAK2V617F-positive ET. The blue lines display the inhibitory effect of proteins, which was confirmed by IPA Knowledge database. The gray lines indicate that the protein interactions lacked literature support to predict the activation effect. Solid lines: direct interactions; dashed lines: indirect interactions; **d** The histogram shows the differential expression of genes involved in cell cycle regulation and DNA replication between CALR-mutated vs. JAK2V617F-positive samples. The green block indicates a decreased expression, while the red block indicates an increased expression in the pairwise comparison CALR-mutated vs. JAK2V617F-positive ET

The IPA functional analysis of modulated transcripts between ET CALR-mutated and JAK2V617F-positive CD34+ cells unveiled differentially activated pathways, such as “mTOR signaling”, “MAPK signaling”, “Role of BRCA1 in DNA damage response”, “ATM signaling”, “Endoplasmic Reticulum stress pathway”, “Protein ubiquitination pathway”, “RhoA signaling,” and “Thrombin signaling” (Fig. 1e). Of note, mTOR signaling appears to be inhibited in CALR-mutated ET patients, as demonstrated by the downregulation of the mTOR2 complex subunit *RICTOR*, and of several upstream and downstream factors (i.e., *PDK1*, *PHIP*, *RHEB*, *PP2A*, and *RPS6KB1*) (Fig. 2a). Similarly, we discovered a reduced expression of several signal transducers involved in MAPK and PI3K/AKT pathways (i.e., *KRAS*, *SOS1*, *MAPK14*, *PI3KCA*, *PI3KR1*, *SHP2*) in CALR-mutated ET samples (Fig. 2b).

In order to predict potentially deregulated mRNA–miRNA interactions, we performed integrative analysis of GEP and miEP by IPA’s miRNA Target Filter. In particular, we selected DEM-DEG pairs with an anti-correlated expression pattern; among the modulated probesets in CALR-mutated vs. JAK2V617F-positive samples, we identified 34 DEMs that have at least one anti-correlated target among DEGs, whereas 720 DEGs have at least one targeting DEM showing an anti-correlated expression. Therefore, 1387 anti-correlated miRNA-target pairs were identified. Table 2 shows the miRNAs with the highest number of predicted targets.

**Table 2:** Differentially expressed miRNA with the highest number of targets

miRNA	Number of targets
miR-30e-5p	131
miR-19a-3p	119
miR-301a-3p	111
miR-152-3p	104
miR-29c-3p	79
miR-140-5p	61
miR-21-5p	59
miR-376c-3p	58
miR-433-3p	51
miR-503-5p	49
miR-134-5p	48
miR-4310	45
miR-362-5p	45
miR-24-2-5p	44
miR-501-3p	43

As shown in Fig. 2a, b, the upregulation of several miRNAs (i.e., miR-326, miR-376c-3p, miR-19a-3p, miR-30e-5p, miR-21-5p, miR-152-3p, miR-503-5p) in CALR-mutated cells could cause the downregulation of mTOR/MAPK/PI3K signaling by targeting some pathway transducers. Among those, miR-152-3p is described as targeting mTOR, PI3K, and MAPK pathways in solid tumors^22–24^, whereas miR-503-5p, and miR-29c-3p were identified as PI3K signaling regulators in lung and bladder cancer, respectively^25,26^. Of note, miR-326 has been already described as regulator of MAPK pathway transducers in gliomas^27^. Moreover, the miR-326 upregulation could be probably ascribed to PI3K inhibition, as already reported in glioblastomas^28^.

Furthermore, several co-activators (i.e., *ATAD2*, *NMI*, *MYCBP2*) and target genes of MYC are downregulated. In fact, IPA upstream regulator analysis showed that MYC activity is lower in CALR-mutated vs. JAK2V617F-positive ET, based on the decreased expression of its known targets (Fig. 2c). Accordingly, we highlighted a decreased expression of cell cycle kinase *CDK6* and of several genes involved in cell proliferation and DNA replication (i.e., *MKI67*, *RFC1*, *BBX*, *DBF4*, *CDC7*), as well as the upregulation of the cell cycle inhibitor *CDKN1C* in CALR-mutated patients (Fig. 2d).

**Fig. 3:**
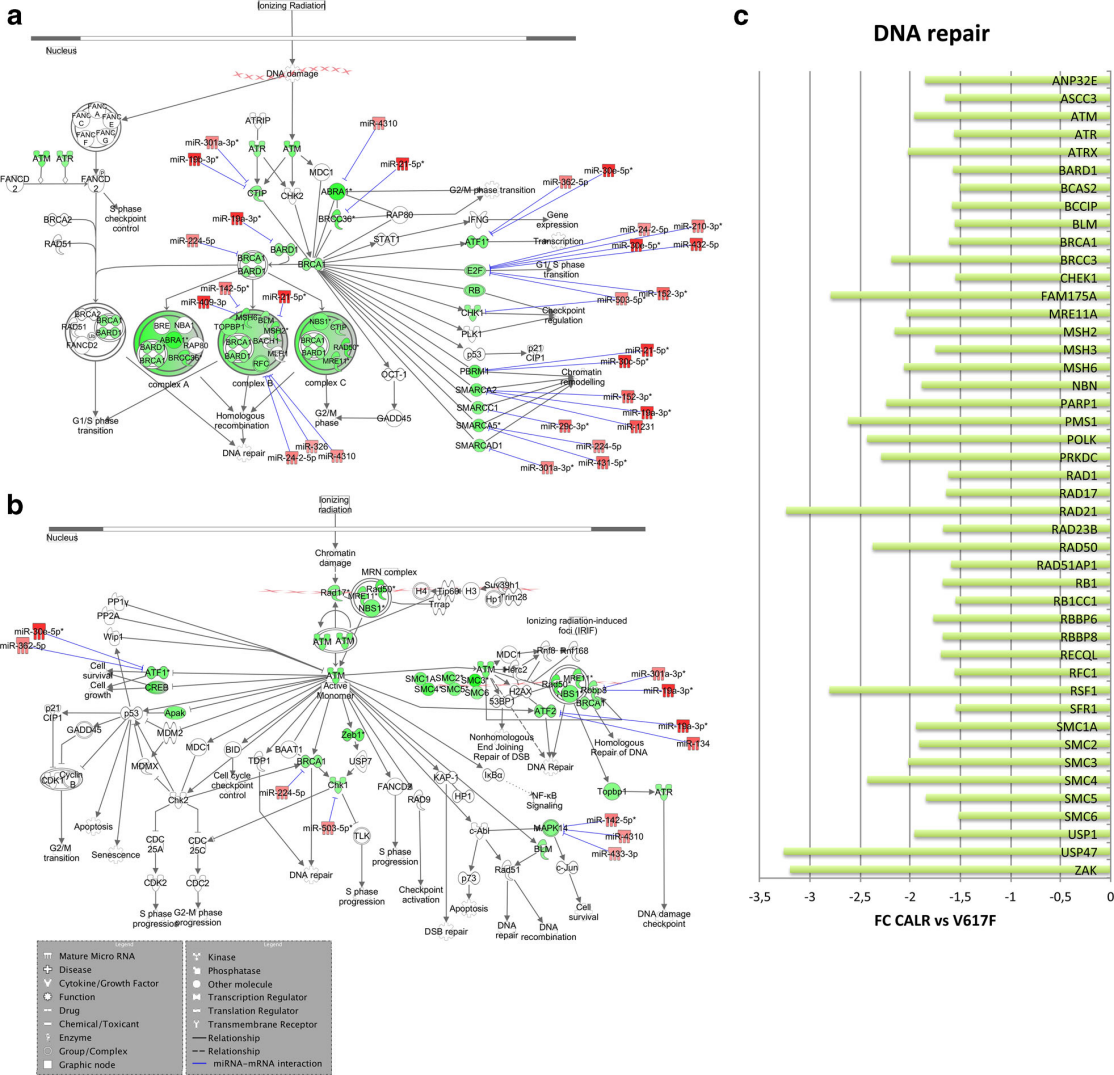
**DNA repair pathways in CALR-mutated vs. JAK2V617F-positive ET.** **a**,** b** IPA canonical pathways highly represented in the list of DEG **a** BRCA1 in the DNA damage response; **b** ATM signaling. The networks also show the predicted mRNA–miRNA interactions identified by IPA’s miRNA Target Filter. Green and red colors indicate genes/miRNA down- and upregulated, respectively, in the pairwise comparison CALR-mutated vs. JAK2V617F-positive ET. **c** The histogram shows the differential expression between CALR-mutated vs. JAK2V617F-positive samples of genes involved in DNA repair. The green block indicates a decreased expression, while the red block indicates an increased expression in the pairwise comparison CALR-mutated vs. JAK2V617F-positive ET

Moreover, DNA repair process seems to be less activated in CALR-mutated cells. In fact, genes involved in BRCA1-driven DNA damage response and ATM signaling, as well as in DNA repair mechanisms (i.e., *SMC*, *MSH*, and *RAD genes*, *SFR1*, *RB1*, *MRE11A*, *FAM175*) are downregulated in CALR-mutated patients (Fig. 3). As showed in Fig. 3a, b, the upregulation of miR-30e-5p, miR-19a-3p, miR-224-5p, miR-503-5p, miR-301a, miR-21-5p, and miR-4310 could justify the reduced expression of DNA repair genes. Interestingly, miR-19a-3p has been already described as regulator of *BARD1* expression in acute myeloid leukemia cells^29^.

### CALR-mutated CD34+ cells show a different expression of CSNK1A1, chromatin remodelers, sister chromatid cohesins, and splicing factors

**Fig. 4:**
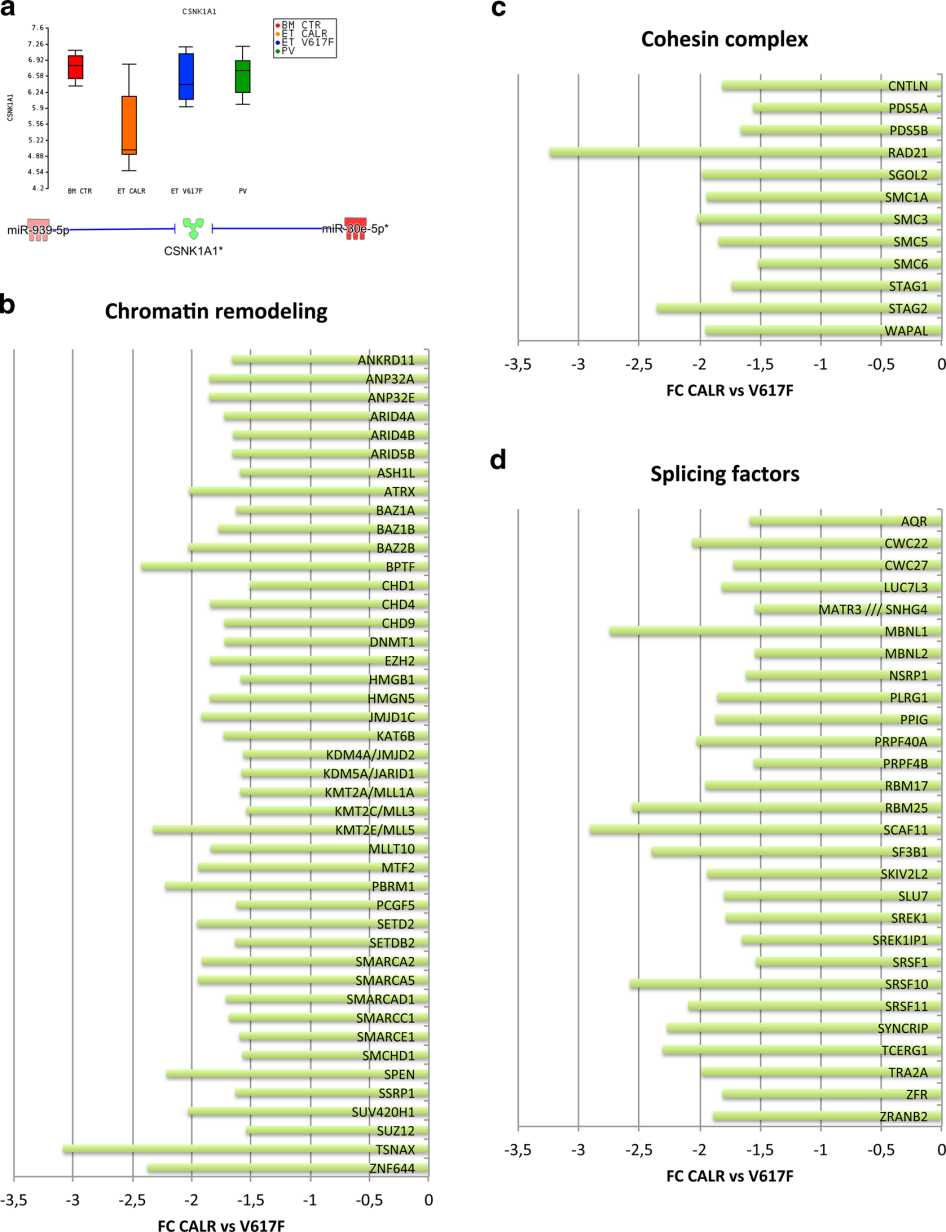
**Deregulated expression of** ***CSNK1A1*** **, chromatin remodeling, cohesin complex, and splicing factors in CALR-mutated ET.** **a** Box plot shows the decreased expression level of *CSNK1A1* in CALR-mutated ET. The lower part of the panel shows the overexpression of two miRNAs targeting *CSNK1A1*. Green and red colors indicate genes/miRNA down- and upregulated, respectively, in the pairwise comparison CALR-mutated vs. JAK2V617F-positive ET. **b–d** The histograms show the differential expression between CALR-mutated vs. JAK2V617F-positive samples of genes involved in **b** chromatin remodeling **c** cohesin complex **d** splicing factors. The green block indicates a decreased expression in the pairwise comparison CALR-mutated vs. JAK2V617F-positive ET

Among DEGs in CALR-mutated ET CD34+ cells, we underlined the downregulation of *CSNK1A1* (Fig. 4a) that has been reported as favoring the clonal expansion of CD34+ progenitors^30^. *CSNK1A1* downregulation could be ascribed to the increased expression of miR-939 and miR-30e-5p in CALR-mutated cells.

Interestingly, CALR-mutated cells show the decreased expression of several epigenetic regulators (i.e., *EZH2*, *SUZ12*, *DNMT1*, *SETD2*, *MLL3*, *ARID4A*, *ARID4B*, *SETDB2*), sister chromatid cohesins (i.e., *SMC1A*, *SMC3*, *RAD21*, *STAG2*), and splicing factors (i.e., *SF3B1*, *SRSF1*, *ZFR*), some of which were already described as mutated or inactivated in myeloid malignancies (Fig. 4b–d)^31–38^. As shown in Supplementary Fig. 2A, the IA identified miR-30e-5p, miR-301a-3p, miR-376c-3p, miR-152-3p, miR-19a-3p, miR-362-5p, and miR-29c-3p as key regulators of chromatin remodeling factors. The decreased expression of cohesins could be ascribed to miR-21-5p, miR-29c-3p, and miR-501-3p overexpression, whereas miR-301a-3p, miR-19a-3p, miR-30e-5p, miR-432-5p, and miR-433-3p might modulate the expression of splicing factors (Supplementary Fig. 2B, C).

Moreover, CALR-mutated cells exhibit the downregulation of several genes involved in protein ubiquitination (i.e., *UBE2J1*, *UBE2V2*, *UBE3A*, *UCHL5*, and *USPs*) and in Endoplasmic Reticulum stress (i.e., *PERK1*, *GRP94*, and *EIF2S1*) (data not shown).

**Fig. 5:**
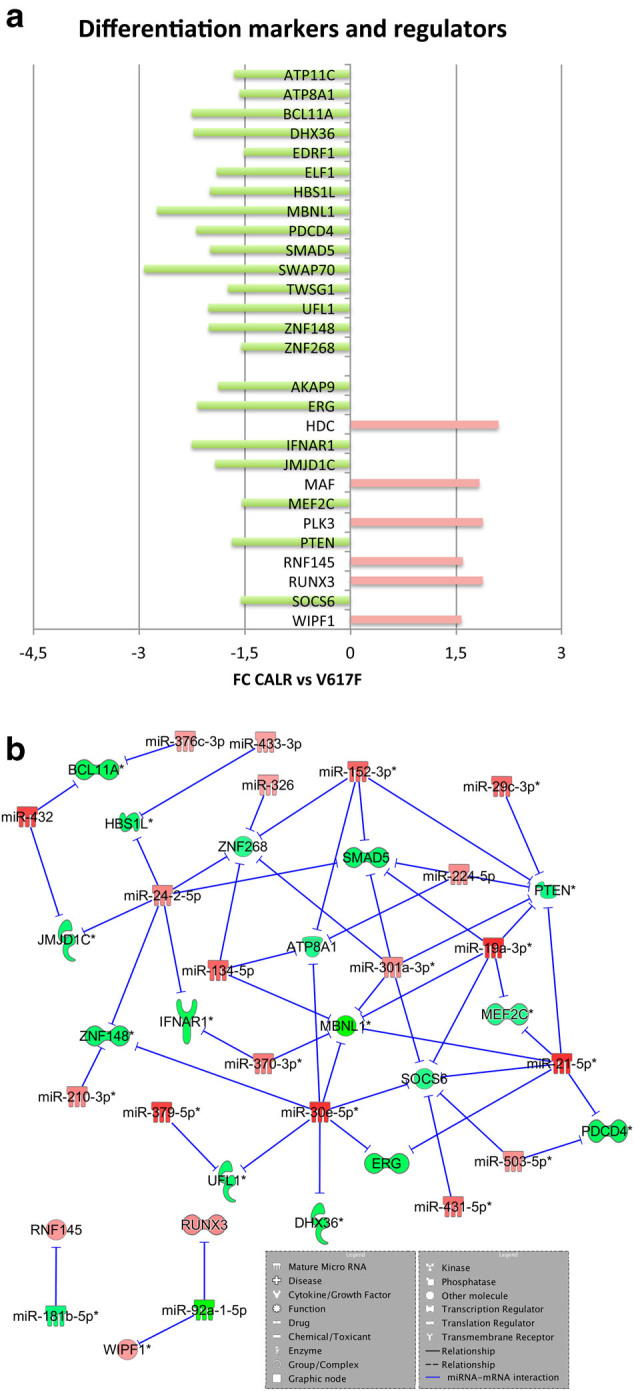
**Differential expression of erythroid and megakaryocyte regulators in CALR-mutated vs. JAK2V617F-positive ET.** **a** The histogram shows the differential expression between CALR-mutated vs. JAK2V617F-positive samples of erythroid and megakaryocyte regulators and markers. The green block indicates a decreased expression, while the red block indicates an increased expression in the pairwise comparison CALR-mutated vs. JAK2V617F-positive ET. **b** Regulatory networks showing the predicted mRNA–miRNA interactions identified by IPA’s miRNA Target Filter. Green and red colors indicate genes/miRNA down- and upregulated, respectively, in the pairwise comparison CALR-mutated vs. JAK2V617F-positive ET

As regards the expression of genes regulating cell differentiation, we found a decreased expression of several erythroid transcription factors (i.e., *ZNF148*, *ZNF268*, *TWSG1*, *PDCD4*, *BCL11A*, *MBNL1*, *UFL1*) in CALR-mutated cells (Fig. 5a). Of note, consistently with their role in erythropoiesis, we highlighted a direct correlation between *TWSG1*, *ZNF148*, or *PDCD4* expression and Hb level (*r* = 0.34, *r* = 0.31, *r* = 0.32, respectively) (Supplementary Table 3).

Moreover, the upregulation of megakaryocyte (MK) transcription factors and markers (i.e., *MAF*, *PLK3*, *WIPF1*)^39^ as well as the downregulation of inhibitors of MK commitment (i.e., *IFNAR1*, *PTEN*, *SOCS6*)^40–42^, could suggest that CALR-mutated CD34+ cells display the susceptibility to differentiate toward MK rather than erythroid lineage (Fig. 5a). In this regard, a positive correlation between *PLK3* or *WIPF1* expression levels and PLT count (*r* = 0.48, *r* = 0.40, respectively) was found. Consistently, we unveiled a negative correlation between *PTEN*, *IFNAR1*, *SOCS6*, or *ZNF148* expression and PLT count (*r* = −0.41, *r* = −0.41, *r* = −0.39, *r* = −0.52, respectively) (Supplementary Table 3). As shown in Fig. 5b, the upregulation of miR-21-5p, miR-19a-3p, miR-301a-3p, miR-503-5p, miR-307-3p, and miR-134-5p might lead to the downregulation of erythroid positive regulators (*PCDC4*, *MBNL1*, *ZNF268*) and MK inhibitors (*SOCS6*, *PTEN*, *IFNAR1*). Of note, the role of miR-503-5p in erythrocyte lineage expansion has been already described^43^.

### CALR-mutated cells display a reduced expression of genes related to platelet activation

**Fig. 6:**
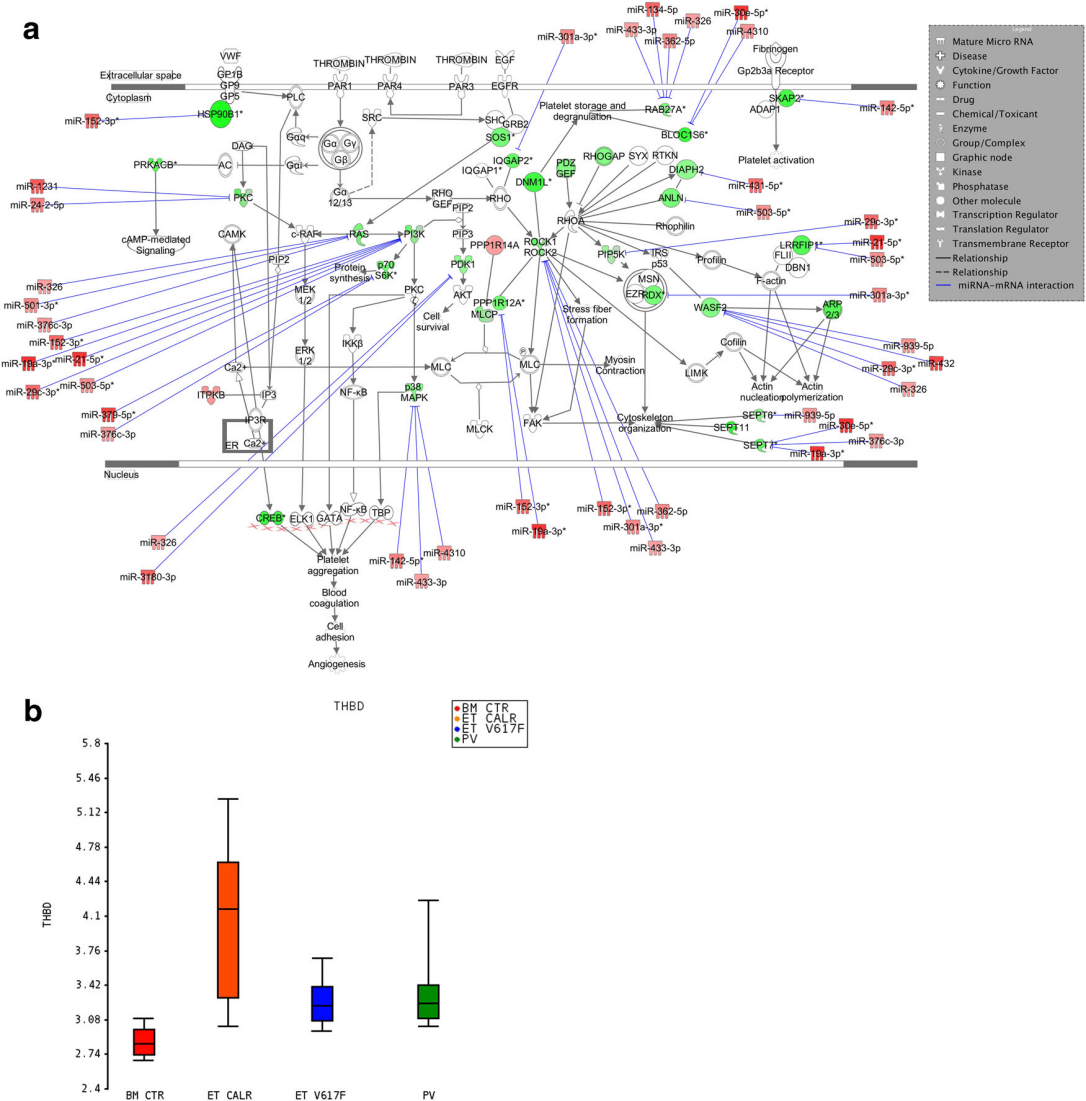
Differential expression of genes involved in platelet activation in CALR-mutated vs. JAK2V617F-positive ET. **a** Thrombin and RhoA signaling in CALR-mutated vs. JAK2V617F-positive ET. The network also shows the predicted mRNA–miRNA interactions identified by IPA’s miRNA Target Filter. Green and red colors indicate genes/miRNA down- and upregulated, respectively, in the pairwise comparison CALR-mutated vs. JAK2V617F-positive ET. **b** Box plot shows the increased expression level of *THBD* in CALR-mutated ET samples

Gene expression analysis highlighted the down-modulation of several proteins involved in thrombin and RhoA signaling (i.e., *ROCK1*, *ROCK2*, *PPP1R12A*, *IQGAP2*, *RAPGEF6*, and *PIP5K*), which regulate the platelet activation and aggregation (i.e., *WASF2*, *ACTR2*, *RDX*), in CALR-mutated patients (Fig. 6a). Moreover, we observed the reduced expression of some regulators of platelet storage and degranulation (i.e., *DNML1*, *RAB27A*, and *BLOC1S6*). Among the miRNAs predicted as modulators of thrombin and RhoA signaling, we underlined the upregulation of miR-376c-3p, which has been already described as involved in platelet activation^44^. Of note, mir-19a-3p was previously described as decreased in peripheral blood cells following acute ischemic stroke^45^.

Finally, CALR-mutated CD34+ cells showed higher expression level of thrombomodulin (*THBD*), an anti-thrombotic factor expressed by endothelial and hematopoietic stem/progenitor cells (Fig. 6b)^46^.

## Discussion

Somatic mutations in the *CALR* gene have been found in 50–70% of *JAK2* and *MPL* wild-type MPN patients^8,9^. Recently, the molecular mechanisms underlying the pathogenetic role of CALR mutated proteins have been unveiled, demonstrating that CALR mutants activates MPL receptor, thus inducing the JAK-STAT activation as well as in JAK2- and MPL-mutated cells^15–17^.

However, as already suggested^13^, CALR-mutated ET show clinical features different from JAKV617F-positive ET and might be considered as a distinct disease entity from JAK2V617F-positive ET; in fact, CALR-mutated ET patients present a higher PLT count coupled with a lower thrombotic risk if compared to JAK2V617F-positive ET patients^10,13,14^.

According to these observations, unsupervised analysis performed on GEP and miEP data shows that CALR-mutated ET samples are clearly separated from both JAK2V617F-positive ET and PV groups, which instead cluster together. Supervised data analysis shows the differential expression of 2040 genes and 488 miRNAs distinguishing ET patients based on their mutational status; on the contrary, we were unable to identify any modulated genes or miRNAs in the pairwise comparison between PV and JAK2V617F-positive ET samples. This evidence supports the hypothesis suggested by Campbell et al.^47^ that ET and PV JAK2V617F-positive are distinct phases of the same disease that exhibits two different phenotypes.

The functional analysis of modulated transcripts unveiled several pathways differentially activated between JAK2V617F- and CALR-mutated progenitor cells. For instance, the expression of mTOR transducers (e.g., *RICTOR*) is decreased in CALR-mutated samples. RICTOR is a key component of mTORC2 complex, which plays a relevant role in leukemic cell proliferation^48^. Interestingly, *RICTOR* deletion prevents leukemogenesis in (*PTEN*)-deficient mouse model showing prolonged lifespan, suggesting that mTORC2 downregulation might partly explain the more indolent phenotype of CALR-mutated ET patients^49^.

Similarly, MAPK and PI3K pathways seem to be less activated in CALR-mutated ET: these data are consistent with the results obtained by Chachoua et al.^15^, which have shown that there is a strong synergy between JAK2 and PI3-K inhibitors in restraining the cytokine-independent proliferation of JAK2V617F-positive cells, unlike what occurs in the CALR-mutated cells. According to MAPK and PI3K signaling inhibition, MYC activity appears decreased as demonstrated by the huge downregulation of its transcriptional targets. In addition, we observed a strong downregulation of several proteins involved in cell cycle control and DNA replication. These evidences suggest that CALR-mutated progenitors are less proliferating compared to their JAK2V617F-positive counterparts.

Our result show that CALR mutant protein could affect signaling pathways other than JAK-STAT; for instance, the decreased expression of *CSNK1A1* in CALR-mutated samples might favor the initial clonal expansion of CD34+ progenitors in ET patients^30^. Moreover, the downregulation of DNA repair pathways mediated by BRCA1 and ATM could suggest the impairment of DNA damage response as new pathogenetic mechanism of CALR-mutant proteins.

Similarly, the downregulation of epigenetic regulators^31–36^, cohesins^37^, and splicing factors^38^, which are frequently mutated or inactivated in several myeloid malignancies, could be involved in the pathogenesis of CALR-mutated disease. Furthermore, CALR mutations seem to impair essential cellular function as protein ubiquitination and ER stress response. The downregulation of genes related to “ER stress response” coupled to partial dislocation from the ER of mutant CALR lacking the KDEL signal^8^, suggests an inefficient response to unfolded protein accumulation in CALR-mutated cells as an additional pathogenetic mechanism (Salati S. et al., submitted).

The recent discovery of physical interaction between CALR-mutated protein and MPL receptor^16^ has definitely explained the link concerning CALR mutations and megakaryocytopoiesis. However, the increased of MK commitment regulator as MAF^39^ coupled to decreased of MK differentiation inhibitors, like *IFNAR1*, *PTEN* and *SOCS6* ^40–42^ could favor the higher PLT count in CALR-mutated subjects compared to JAK2V617F-positive ET patients. According to this hypothesis, our data disclosed a negative correlation between *PTEN*, *IFNAR1*, or *SOCS6* expression levels and PLT count. In particular, as described by Zhang et al.^41^, the simultaneous downregulation of PTEN and MYC signaling in CALR-mutated MPN patients might favor the switch from granulocyte- to MK- commitment. Moreover, we highlighted the increased level of *PLK3* that was already described as having a pivotal role in megakaryocyte polyploidization and differentiation and that might justify the higher number of PLT in CALR-mutated patients^50^. Consistently, a positive correlation between *PLK3* expression level and PLT count was found.

We also unveiled a direct correlation between the hemoglobin level and the expression of some erythroid positive regulators, which are decreased in CALR-mutated patients. Therefore, CALR mutant proteins could affect the expression of several erythroid and MK differentiation-related genes, then contributing to lineage fate decision of CALR-mutated CD34+ cells.

As for reduced thrombotic risk in ET CALR-mutated patients, we uncovered the down-modulation of several proteins involved in thrombin and RhoA signaling. Moreover, several genes involved in platelet activation, aggregation, and degranulation are decreased in CALR-mutated progenitors, while the anti-thrombotic factor *THBD* is upregulated. These data are consistent with the results obtained by Torregrosa et al.^51^, showing a reduced platelet activation in CALR-mutated ET patients compared to JAK2V617F-positive ET subjects. Overall, these observations could explain the low thrombotic risk in CALR-mutated patients, even though they have a higher PLT count as compared to JAK2V617F-positive patients.

Finally, by means of integrated analysis of GEP and miEP, we identified several miRNA–mRNA interactions, which could represent novel pathogenetic mechanisms mediated by CALR mutations and could affect the patient’s clinical phenotype. In particular, we unveiled the upregulation of several miRNAs (e.g., miR-30e-5p, miR-19a-3p, miR-301a-3p, miR-152-3p, miR-29c-3p, miR-21-5p, miR-503-5p, miR-376c-3p), which are involved in the inhibition of mTOR/MAPK/PI3K signaling, DNA damage response, chromatin remodeling, alternative spicing, and PLT activation.

As a whole, this study suggests that the molecular characterization of CD34+ cells from CALR-mutated and JAK2V617F-positive ET patients, could shed light on the signaling pathways deregulated in CALR-mutated patients and elucidate their contribution to typical features of these patients, such as high platelet count and low thrombotic risk.

Overall, this study supports the pathogenetic model in which JAK2V617F-positive ET and PV could be considered as different phenotypes or phases of a single MPN characterized by JAK2 mutation^47^, whereas CALR-mutated ET seems to be a distinct entity both at clinical^13^ and at molecular level.

## Electronic supplementary material

Supplementary Figure and Table legends

Supplementary Figure S1

Supplementary Figure S2

Supplementary Table S1

Supplementary Table S2

Supplementary Table S3
