# Supplementary material for: CALR mutational status identifies different disease subtypes of essential thrombocythemia showing distinct expression profiles
Source: Blood Cancer J. 2017 Dec 8;7(12):638. doi: 10.1038/s41408-017-0010-2 (PMC5802509; doi:10.1038/s41408-017-0010-2)
Supplement: Supplementary file 1 — Supplementary Figure and Table legends [file 41408_2017_10_MOESM1_ESM.docx]

**SUPPLEMENTARY FIGURE AND TABLE LEGENDS**

**Figure S1. Gene and miRNA expression profile of PV and ET CD34+ cells. (**A-B) Principal component analysis (PCA) on gene and miRNA expression microarray data. The PCA graph of global gene expression (A) and miRNA expression data (B) were computed using Partek GS, version 6.6. BM control samples are shown as red spheres; ET samples are shown in blue; PV samples are shown in green. (C-D) Gene Set Enrichment Analysis (GSEA) using the gene sets lists of increased (482) and decreased (326) gene probesets in the pairwise comparison ET versus BM CTR. The samples analyzed are: PV (n=26) and BM CTR (n=15). The normalized enrichment score (NES), and the false discovery rates (FDR) are indicated for each gene set. (E-F) Gene Set Enrichment Analysis (GSEA) using the gene sets lists of increased (448) and decreased (264) gene probesets in the pairwise comparison PV versus BM CTR. The samples analyzed are: ET (n=24) and BM CTR (n=15). The normalized enrichment score (NES), and the false discovery rates (FDR) are indicated for each gene set.

**Figure S2. Regulatory networks showing the predicted mRNA-miRNA interactions identified by IPA's miRNA Target Filter.** Regulatory networks of mRNA-miRNA interactions related to (A) chromatin remodeling (B) cohesin complex (C) splicing factors. Green and red colors indicate genes/miRNA down- and upregulated, respectively, in the pairwise comparison CALR-mutated versus JAK2V617F-positive ET.

**Table S1. DEG in the pairwise comparison CALR-mutated vs JAK2V617F-positive ET**

**Table S2. DEM in the pairwise comparison CALR-mutated vs JAK2V617F-positive ET**

**Table S3. Clinical correlations between hematological parameters and gene expression**
