# Supplementary material for: CALR mutational status identifies different disease subtypes of essential thrombocythemia showing distinct expression profiles
Source: Blood Cancer J. 2017 Dec 8;7(12):638. doi: 10.1038/s41408-017-0010-2 (PMC5802509; doi:10.1038/s41408-017-0010-2)
Supplement: Supplementary file 4 — Supplementary Table S1 [file 41408_2017_10_MOESM4_ESM.pdf]

Table S1. DEG in the pairwise comparison CALR-mutated vs JAK2V617F-positive ET

Probeset ID	Entrez Gene	Gene Symbol	Gene Title	q (ET CALR vs. PV)	FC (ET CALR vs. PV)	q (ET CALR vs. ET V617F)	FC (ET CALR vs. ET V617F)	q (ET V617F vs. PV)	FC (ET V617F vs. PV)	q (ET CALR vs. BM CTR)	FC (ET CALR vs. BM CTR)	q (ET V617F vs. BM CTR)	FC (ET V617F vs. BM CTR)	q (PV vs. BM CTR)	FC (PV vs. BM CTR)
11746371_a_at	60496	AASDHPPT	aminoadipate-semialdehyde dehydrogenase-phosphopantetheinyl transferase	0,02824	-1,62	0,03877	-1,69	0,924842	1,05	0,341535	1,24	3,95E-05	2,11	2,46E-05	2,02
11728968_a_at	60496	AASDHPPT	aminoadipate-semialdehyde dehydrogenase-phosphopantetheinyl transferase	0,006606	-1,66	0,047311	-1,53	0,79848	-1,09	0,101423	-1,36	0,44309	1,12	0,150072	1,22
11739600_s_at	10157	AASS	aminoadipate-semialdehyde synthase	0,005348	-1,90	0,046584	-1,68	0,764997	-1,13	3,07E-05	-2,85	0,003272	-1,70	0,014519	-1,50
11749150_a_at	5825	ABCD3	ATP-binding cassette, sub-family D (ALD), member 3	0,0139	-1,59	0,033864	-1,58	0,993191	-1,00	0,514381	1,14	0,00011	1,80	2,58E-05	1,81
11747617_a_at	5825	ABCD3	ATP-binding cassette, sub-family D (ALD), member 3	0,005024	-2,00	0,021502	-1,91	0,926322	-1,05	0,089347	-1,51	0,239292	1,26	0,132433	1,32
11752588_s_at	6059	ABCE1	ATP-binding cassette, sub-family E (OABP), member 1	0,000351	-2,42	0,003832	-2,21	0,826008	-1,10	0,91482	-1,03	4,09E-05	2,15	1,11E-06	2,36
11747859_s_at	6059	ABCE1	ATP-binding cassette, sub-family E (OABP), member 1	0,004903	-1,78	0,019308	-1,73	0,944226	-1,03	0,245187	-1,27	0,053998	1,36	0,023583	1,40
11743804_at	6059	ABCE1	ATP-binding cassette, sub-family E (OABP), member 1	0,002368	-1,72	0,018185	-1,60	0,827522	-1,07	0,016638	-1,52	0,719818	1,06	0,364099	1,13
11747860_x_at	6059	ABCE1	ATP-binding cassette, sub-family E (OABP), member 1	0,006316	-1,83	0,049829	-1,64	0,780195	-1,12	0,124114	-1,40	0,389008	1,17	0,10836	1,30
11739177_at	51104	ABHD17B	abhydrolase domain containing 17B	0,009253	-1,77	0,014967	-1,86	0,918114	1,05	0,428873	-1,20	0,011119	1,55	0,015055	1,48
11723703_s_at	51099	ABHD5	abhydrolase domain containing 5	0,005486	-1,68	0,014112	-1,69	0,985431	1,01	0,875387	1,03	0,000167	1,75	5,76E-05	1,73
11747346_x_at	34	ACADM	acyl-CoA dehydrogenase, C-4 to C-12 straight chain	0,000184	-2,21	0,001562	-2,12	0,911296	-1,05	0,002491	-1,83	0,353364	1,16	0,199712	1,21
11752919_x_at	34	ACADM	acyl-CoA dehydrogenase, C-4 to C-12 straight chain	0,000272	-1,86	0,002666	-1,78	0,879665	-1,05	0,007186	-1,54	0,247702	1,16	0,102278	1,21
11722357_a_at	34	ACADM	acyl-CoA dehydrogenase, C-4 to C-12 straight chain	0,000358	-2,30	0,00363	-2,12	0,84258	-1,08	0,003084	-1,92	0,590341	1,10	0,287362	1,20
11747345_s_at	34	ACADM	acyl-CoA dehydrogenase, C-4 to C-12 straight chain	0,000576	-1,79	0,00245	-1,80	0,99135	1,00	0,008977	-1,52	0,190908	1,18	0,183116	1,18
11747344_a_at	34	ACADM	acyl-CoA dehydrogenase, C-4 to C-12 straight chain	0,00041	-2,15	0,004887	-1,96	0,796211	-1,10	0,004712	-1,78	0,589281	1,10	0,230944	1,20
11746834_a_at	34	ACADM	acyl-CoA dehydrogenase, C-4 to C-12 straight chain	0,000522	-2,03	0,003648	-1,95	0,916564	-1,04	0,005818	-1,71	0,404936	1,14	0,245868	1,19
11743566_at	64746	ACBD3	acyl-CoA binding domain containing 3	0,000793	-1,77	0,017582	-1,55	0,644099	-1,14	6,90E-05	-2,01	0,041503	-1,29	0,309207	-1,14

11746650_a_at	55331	ACER3	alkaline ceramidase 3	0,085335	-1,32	0,026247	-1,52	0,623483	1,15	0,073751	-1,34	0,350803	1,13	0,9205	-1,02
11739261_s_at	2181	ACSL3	acyl-CoA synthetase long-chain family member 3	0,003452	-1,68	0,009116	-1,70	0,978346	1,01	0,024271	-1,48	0,332339	1,15	0,36002	1,13
11739035_x_at	10097	ACTR2	ARP2 actin-related protein 2 homolog (yeast)	0,003318	-2,26	0,01194	-2,22	0,975014	-1,02	0,574341	1,18	1,75E-05	2,61	2,44E-06	2,67
11748806_x_at	10097	ACTR2	ARP2 actin-related protein 2 homolog (yeast)	0,004804	-2,04	0,014446	-2,03	0,994152	-1,00	0,47925	1,20	1,35E-05	2,44	2,44E-06	2,45
11739033_a_at	10097	ACTR2	ARP2 actin-related protein 2 homolog (yeast)	0,003653	-2,55	0,014634	-2,44	0,952553	-1,04	0,641872	1,17	4,19E-05	2,87	4,81E-06	2,99
11739034_s_at	10097	ACTR2	ARP2 actin-related protein 2 homolog (yeast)	0,012383	-2,00	0,03308	-1,97	0,982086	-1,01	0,381506	1,29	3,93E-05	2,53	6,65E-06	2,57
11743580_s_at	10097	ACTR2	ARP2 actin-related protein 2 homolog (yeast)	0,013654	-1,83	0,043295	-1,76	0,947823	-1,04	0,391515	1,24	7,60E-05	2,19	8,82E-06	2,27
11745442_x_at	10097	ACTR2	ARP2 actin-related protein 2 homolog (yeast)	0,023664	-1,70	0,04921	-1,70	0,995227	1,00	0,456168	1,20	0,000162	2,04	4,80E-05	2,04
11739031_s_at	10097	ACTR2	ARP2 actin-related protein 2 homolog (yeast)	0,004568	-1,51	0,011492	-1,52	0,976444	1,01	0,017151	-1,41	0,514044	1,08	0,565379	1,07
11736026_at	102	ADAM10	ADAM metallopeptidase domain 10	0,059462	-1,63	0,04138	-1,85	0,791394	1,13	0,443811	1,24	0,000103	2,29	0,000295	2,02
11721606_a_at	120	ADD3	adducin 3 (gamma)	0,097902	-1,28	0,004657	-1,67	0,413308	1,30	8,27E-05	-1,91	0,25344	-1,15	0,000309	-1,49
11717348_a_at	120	ADD3	adducin 3 (gamma)	0,144268	-1,26	0,013065	-1,59	0,447888	1,26	0,000109	-1,95	0,096208	-1,23	0,000195	-1,55
11717346_a_at	120	ADD3	adducin 3 (gamma)	0,090016	-1,34	0,008606	-1,72	0,454739	1,28	0,008318	-1,60	0,637681	1,07	0,189686	-1,19
11726731_a_at	55109	AGGF1	angiogenic factor with G patch and FHA domains 1	3,41E-05	-1,98	0,000723	-1,84	0,773333	-1,08	0,322297	-1,16	9,32E-05	1,58	1,63E-06	1,71
11754726_a_at	55109	AGGF1	angiogenic factor with G patch and FHA domains 1	7,73E-05	-2,45	0,001283	-2,24	0,803092	-1,10	0,032946	-1,55	0,020694	1,45	0,001913	1,59
11726732_at	55109	AGGF1	angiogenic factor with G patch and FHA domains 1	0,000287	-1,70	0,003134	-1,62	0,846724	-1,05	0,010537	-1,42	0,21919	1,14	0,070735	1,20
11751538_a_at	55109	AGGF1	angiogenic factor with G patch and FHA domains 1	0,001362	-1,83	0,021886	-1,61	0,691407	-1,13	0,138083	-1,31	0,148395	1,23	0,011967	1,39
11720888_s_at	178	AGL	amylo-alpha-1, 6-glucosidase, 4-alpha-glucanotransferase	0,003817	-1,84	0,03283	-1,66	0,784723	-1,11	0,016298	-1,65	0,977229	1,01	0,523404	1,11
11730754_s_at	55326	AGPAT5	1-acylglycerol-3-phosphate O-acyltransferase 5	0,003733	-1,92	0,017033	-1,84	0,925978	-1,04	0,513063	-1,17	0,008706	1,58	0,001976	1,65
11758491_s_at	8540	AGPS	alkylglycerone phosphate synthase	0,002456	-1,88	0,033274	-1,64	0,703036	-1,15	0,000806	-2,03	0,179411	-1,24	0,639393	-1,08
11754549_a_at	23287	AGTPBP1	ATP/GTP binding protein 1	0,007347	-1,53	0,006712	-1,66	0,78613	1,08	0,026505	-1,42	0,229037	1,17	0,571044	1,08
11750571_a_at	23287	AGTPBP1	ATP/GTP binding protein 1	0,016832	-1,42	0,010253	-1,56	0,723912	1,10	0,008584	-1,48	0,70074	1,05	0,741283	-1,04
11744946_x_at	64853	AIDA	axin interactor, dorsalization associated	0,000518	-1,63	0,005845	-1,54	0,801767	-1,06	0,042867	-1,31	0,120362	1,18	0,022337	1,25

11748857_a_at	50808	AK3	adenylate kinase 3	0,001338	-2,02	0,015219	-1,81	0,767752	-1,12	0,056104	-1,50	0,262962	1,21	0,05455	1,35
11719493_s_at	6880 /// 102157402	AK6 /// TAF9	adenylate kinase 6 /// TAF9 RNA polymerase II, TATA box binding protein (TBP)-associate	0,000171	-1,73	0,004608	-1,56	0,6681	-1,10	0,000129	-1,73	0,350684	-1,11	0,993522	-1,00
11723297_a_at	11217 /// 445815	AKAP2 /// PALM2-AKAP2	A kinase (PRKA) anchor protein 2 /// PALM2-AKAP2 readthrough	0,005915	-1,82	0,021328	-1,77	0,952537	-1,03	0,010716	-1,75	0,942948	1,01	0,827379	1,04
11739979_a_at	11217 /// 445815	AKAP2 /// PALM2-AKAP2	A kinase (PRKA) anchor protein 2 /// PALM2-AKAP2 readthrough	0,00625	-1,82	0,02271	-1,76	0,948783	-1,03	0,021879	-1,65	0,717564	1,07	0,585215	1,10
11746746_a_at	10142	AKAP9	A kinase (PRKA) anchor protein 9	0,003703	-1,89	0,012038	-1,88	0,98907	-1,01	4,90E-06	-3,07	0,003818	-1,64	0,002066	-1,62
11760565_x_at	10142	AKAP9	A kinase (PRKA) anchor protein 9	0,005659	-1,67	0,015268	-1,68	0,995022	1,00	5,40E-05	-2,26	0,039973	-1,35	0,027131	-1,35
11751067_x_at	79647	AKIRIN1	akirin 1	0,007548	-1,75	0,019152	-1,75	0,994718	1,00	0,382477	-1,21	0,022213	1,45	0,015209	1,45
11758213_s_at	79647	AKIRIN1	akirin 1	0,006216	-2,02	0,043493	-1,80	0,805682	-1,12	0,293176	-1,32	0,117171	1,37	0,022066	1,53
11730745_x_at	79647	AKIRIN1	akirin 1	0,018957	-1,51	0,044007	-1,51	0,99368	-1,00	0,462503	-1,15	0,05014	1,31	0,034922	1,32
11716184_s_at	214	ALCAM	activated leukocyte cell adhesion molecule	0,004991	-2,15	0,022732	-2,02	0,912471	-1,06	0,972534	1,01	0,000848	2,04	9,70E-05	2,17
11722489_a_at	9724 /// 440138	ALG11 /// UTP14C	ALG11, alpha-1,2-mannosyltransferase /// UTP14, U3 small nucleolar ribonucleoprotein, h	0,000723	-1,79	0,01116	-1,62	0,723977	-1,11	0,030331	-1,42	0,332289	1,14	0,058332	1,26
11722485_a_at	9724 /// 440138	ALG11 /// UTP14C	ALG11, alpha-1,2-mannosyltransferase /// UTP14, U3 small nucleolar ribonucleoprotein, h	0,003735	-1,75	0,030916	-1,60	0,792121	-1,10	0,019005	-1,57	0,901194	1,02	0,461985	1,12
11758228_s_at	9949	AMMECR1	Alport syndrome, mental retardation, midface hypoplasia and elliptocytosis chromosomal	0,006115	-1,91	0,018024	-1,89	0,988389	-1,01	0,230997	-1,33	0,05406	1,42	0,036161	1,44
11754697_a_at	25847	ANAPC13	anaphase promoting complex subunit 13	0,004073	-1,55	0,011902	-1,56	0,997025	1,00	0,00023	-1,81	0,206213	-1,16	0,182169	-1,17
11726693_s_at	284	ANGPT1	angiopoietin 1	0,000631	-2,19	0,023192	-1,75	0,554958	-1,25	0,104252	-1,42	0,220969	1,23	0,006222	1,54
11726692_at	284	ANGPT1	angiopoietin 1	0,000993	-2,81	0,031071	-2,09	0,570226	-1,34	0,0064	-2,29	0,732713	-1,09	0,384782	1,23
11727064_a_at	29123	ANKRD11	ankyrin repeat domain 11	0,005496	-1,71	0,020733	-1,66	0,9463	-1,03	0,00011	-2,22	0,051271	-1,34	0,062684	-1,30
11757991_s_at	23253	ANKRD12	ankyrin repeat domain 12	0,011435	-1,77	0,032253	-1,74	0,975248	-1,02	0,710747	-1,10	0,009199	1,59	0,003712	1,61
11751384_a_at	23253	ANKRD12	ankyrin repeat domain 12	0,007942	-1,68	0,034734	-1,60	0,904376	-1,05	0,040342	-1,49	0,6704	1,07	0,446237	1,13
11747371_a_at	26057	ANKRD17	ankyrin repeat domain 17	9,78E-05	-1,85	0,002379	-1,70	0,723863	-1,09	1,41E-05	-2,00	0,141869	-1,18	0,507427	-1,08
11750739_a_at	84250	ANKRD32	ankyrin repeat domain 32	0,00313	-1,79	0,018107	-1,69	0,883468	-1,06	0,017838	-1,58	0,692025	1,07	0,427309	1,13
11742908_a_at	84250	ANKRD32	ankyrin repeat domain 32	0,00404	-1,62	0,019834	-1,56	0,907897	-1,04	0,048747	-1,39	0,384217	1,12	0,217777	1,17
11762617_x_at	375248	ANKRD36	ankyrin repeat domain 36	0,000905	-2,58	0,006049	-2,41	0,896148	-1,07	1,28E-05	-3,78	0,034043	-1,57	0,059282	-1,46

11745319_s_at	57730 /// 375248 /// 400986	ANKRD36 /// ANKRD36B /// ANKRD36C	ankyrin repeat domain 36 /// ankyrin repeat domain 36B /// ankyrin repeat domain 36C	0,000328	-2,83	0,007535	-2,32	0,667055	-1,22	1,62E-05	-3,63	0,032265	-1,57	0,228765	-1,28
11746606_s_at	375248 /// 400986	ANKRD36 /// ANKRD36C	ankyrin repeat domain 36 /// ankyrin repeat domain 36C	0,001147	-2,68	0,018979	-2,19	0,692552	-1,22	0,000115	-3,36	0,06125	-1,53	0,32308	-1,25
11724464_a_at	54443	ANLN	anillin, actin binding protein	0,00523	-2,13	0,033377	-1,92	0,837307	-1,11	0,034953	-1,76	0,707976	1,09	0,368621	1,21
11753749_x_at	8125	ANP32A	acidic (leucine-rich) nuclear phosphoprotein 32 family, member A	1,16E-05	-2,32	0,002166	-1,85	0,457902	-1,26	4,70E-07	-2,65	0,005726	-1,43	0,309043	-1,14
11725046_a_at	81611	ANP32E	acidic (leucine-rich) nuclear phosphoprotein 32 family, member E	0,000304	-2,10	0,005742	-1,85	0,711086	-1,13	6,36E-05	-2,28	0,163347	-1,23	0,586402	-1,09
11742062_a_at	81611	ANP32E	acidic (leucine-rich) nuclear phosphoprotein 32 family, member E	0,000242	-1,86	0,007046	-1,64	0,635793	-1,14	0,000544	-1,76	0,570172	-1,08	0,682424	1,06
11725047_x_at	81611	ANP32E	acidic (leucine-rich) nuclear phosphoprotein 32 family, member E	0,000488	-1,83	0,010419	-1,62	0,669716	-1,13	0,000201	-1,90	0,213593	-1,17	0,781968	-1,04
11749393_a_at	81611	ANP32E	acidic (leucine-rich) nuclear phosphoprotein 32 family, member E	0,000788	-1,80	0,01575	-1,59	0,667055	-1,13	0,00015	-1,96	0,101546	-1,24	0,510063	-1,09
11741574_s_at	55435	AP1AR	adaptor-related protein complex 1 associated regulatory protein	0,000901	-1,79	0,016172	-1,59	0,68209	-1,13	0,04287	-1,40	0,353959	1,13	0,046937	1,28
11719295_a_at	8546	AP3B1	adaptor-related protein complex 3, beta 1 subunit	0,0021	-2,00	0,021067	-1,79	0,772553	-1,12	0,428091	-1,20	0,020012	1,49	0,001344	1,67
11729630_a_at	26985	AP3M1	adaptor-related protein complex 3, mu 1 subunit	0,004006	-1,77	0,038101	-1,59	0,759335	-1,11	0,734615	1,08	0,000569	1,71	1,15E-05	1,90
11748791_a_at	55745	AP5M1	adaptor-related protein complex 5, mu 1 subunit	0,006393	-2,02	0,017274	-2,03	0,999238	1,00	0,985341	-1,01	0,000633	2,01	0,000215	2,01
11743455_s_at	55745	AP5M1	adaptor-related protein complex 5, mu 1 subunit	0,014589	-1,52	0,028324	-1,55	0,97118	1,01	0,753333	-1,06	0,005949	1,46	0,004303	1,43
11752481_a_at	351	APP	amyloid beta (A4) precursor protein	0,027955	-1,56	0,028092	-1,68	0,856905	1,07	0,013502	-1,67	0,977806	1,01	0,712016	-1,07
11751350_a_at	351	APP	amyloid beta (A4) precursor protein	0,048358	-1,52	0,036433	-1,67	0,802713	1,10	0,025673	-1,62	0,860733	1,03	0,736896	-1,06
11750384_a_at	351	APP	amyloid beta (A4) precursor protein	0,065166	-1,45	0,038369	-1,62	0,754062	1,12	0,036201	-1,53	0,756325	1,06	0,745871	-1,06
11758116_s_at	10513	APPBP2	amyloid beta precursor protein (cytoplasmic tail) binding protein 2	0,03311	-1,51	0,043816	-1,58	0,9213	1,04	0,000217	-2,18	0,035955	-1,38	0,011504	-1,44
11724572_s_at	10513	APPBP2	amyloid beta precursor protein (cytoplasmic tail) binding protein 2	0,001907	-1,57	0,004735	-1,61	0,945862	1,02	0,00035	-1,71	0,607781	-1,06	0,470205	-1,09
11722737_at	26060	APPL1	adaptor protein, phosphotyrosine interaction, PH domain and leucine zipper containing 1	2,11E-05	-2,79	0,000257	-2,66	0,911129	-1,05	4,13E-05	-2,56	0,83567	1,04	0,620963	1,09

11722735_at	26060	APPL1	adaptor protein, phosphotyrosine interaction, PH domain and leucine zipper containing 1	0,008285	-1,72	0,023064	-1,70	0,986509	-1,01	0,000147	-2,30	0,059433	-1,35	0,05203	-1,34
11722736_s_at	26060	APPL1	adaptor protein, phosphotyrosine interaction, PH domain and leucine zipper containing 1	0,011154	-1,86	0,039955	-1,78	0,93192	-1,05	0,000278	-2,57	0,053613	-1,45	0,073881	-1,38
11722730_at	26060	APPL1	adaptor protein, phosphotyrosine interaction, PH domain and leucine zipper containing 1	0,002479	-2,05	0,011926	-1,97	0,931702	-1,04	0,047982	-1,58	0,237177	1,24	0,13579	1,30
11722733_x_at	26060	APPL1	adaptor protein, phosphotyrosine interaction, PH domain and leucine zipper containing 1	0,004535	-1,88	0,025763	-1,75	0,869803	-1,07	0,033577	-1,59	0,614541	1,10	0,342658	1,18
11759408_at	9716	AQR	aquarius intron-binding spliceosomal factor	0,003025	-1,65	0,015879	-1,59	0,905803	-1,04	9,77E-05	-2,01	0,067662	-1,27	0,1128	-1,22
11719931_x_at	55082	ARGLU1	arginine and glutamate rich 1	0,002293	-1,98	0,017093	-1,82	0,83739	-1,09	0,000501	-2,22	0,252296	-1,22	0,513691	-1,12
11730292_a_at	394	ARHGAP5	Rho GTPase activating protein 5	0,000746	-2,06	0,025766	-1,68	0,559454	-1,23	0,186196	-1,31	0,121563	1,28	0,002269	1,57
11730291_a_at	394	ARHGAP5	Rho GTPase activating protein 5	0,005645	-1,59	0,013285	-1,61	0,97153	1,01	0,520892	-1,12	0,005356	1,44	0,003776	1,42
11730287_a_at	394	ARHGAP5	Rho GTPase activating protein 5	0,001195	-1,93	0,014943	-1,73	0,751938	-1,11	0,009651	-1,66	0,820918	1,04	0,329719	1,16
11730290_x_at	394	ARHGAP5	Rho GTPase activating protein 5	0,002037	-1,85	0,02927	-1,62	0,696894	-1,14	0,002091	-1,85	0,410012	-1,14	0,992421	1,00
11758442_s_at	5926	ARID4A	AT rich interactive domain 4A (RBP1-like)	0,000493	-1,87	0,004657	-1,76	0,847432	-1,06	0,000916	-1,79	0,918171	-1,02	0,75963	1,05
11740704_a_at	5926	ARID4A	AT rich interactive domain 4A (RBP1-like)	0,00208	-1,87	0,016872	-1,73	0,822176	-1,08	0,000176	-2,22	0,108094	-1,28	0,271486	-1,18
11743713_x_at	5926	ARID4A	AT rich interactive domain 4A (RBP1-like)	0,001101	-1,86	0,006038	-1,80	0,932951	-1,03	0,003269	-1,73	0,804489	1,04	0,636507	1,08
11743714_a_at	5926	ARID4A	AT rich interactive domain 4A (RBP1-like)	0,005044	-1,78	0,027252	-1,68	0,878346	-1,06	0,000475	-2,12	0,143085	-1,27	0,271836	-1,19
11743712_a_at	5926	ARID4A	AT rich interactive domain 4A (RBP1-like)	0,004683	-1,68	0,034506	-1,55	0,807898	-1,08	0,022016	-1,52	0,894995	1,02	0,486142	1,11
11747219_a_at	51742	ARID4B	AT rich interactive domain 4B (RBP1-like)	0,013124	-1,65	0,034466	-1,64	0,982569	-1,01	0,564608	-1,13	0,021236	1,44	0,011107	1,46
11721879_s_at	51742	ARID4B	AT rich interactive domain 4B (RBP1-like)	0,006093	-1,72	0,02587	-1,65	0,919139	-1,04	0,002612	-1,83	0,517112	-1,11	0,701287	-1,07
11750222_a_at	84159	ARID5B	AT rich interactive domain 5B (MRF1-like)	0,025951	-1,53	0,023064	-1,66	0,826008	1,08	0,000116	-2,24	0,047057	-1,35	0,007178	-1,46
11721645_x_at	400	ARL1	ADP-ribosylation factor-like 1	0,003318	-1,64	0,01872	-1,56	0,885801	-1,05	0,234708	-1,22	0,055656	1,28	0,014665	1,34
11754120_a_at	26225 /// 101929356	ARL5A /// LOC101929356	ADP-ribosylation factor-like 5A /// uncharacterized LOC101929356	0,000102	-1,83	0,000682	-1,80	0,959016	-1,02	2,02E-06	-2,16	0,107509	-1,20	0,125944	-1,18
11721801_at	55207	ARL8B	ADP-ribosylation factor-like 8B	0,008805	-1,57	0,028217	-1,54	0,960501	-1,02	0,662036	-1,08	0,009413	1,42	0,003229	1,44

11720264_at	10973	ASCC3	activating signal cointegrator 1 complex subunit 3	0,001876	-1,84	0,020814	-1,65	0,753803	-1,11	0,001964	-1,83	0,518753	-1,11	0,984022	1,00
11720788_at	25842	ASF1A	anti-silencing function 1A histone chaperone	0,002154	-1,75	0,005742	-1,78	0,964736	1,02	0,162085	-1,28	0,018413	1,39	0,017086	1,36
11739570_at	55870	ASH1L	ash1 (absent, small, or homeotic)-like (Drosophila)	0,00045	-1,76	0,007816	-1,59	0,713122	-1,10	0,000266	-1,79	0,346017	-1,12	0,903039	-1,02
11739366_at	444	ASPH	aspartate beta-hydroxylase	0,003	-1,84	0,027456	-1,66	0,777594	-1,11	0,008257	-1,71	0,871021	-1,03	0,673077	1,07
11744907_a_at	55726	ASUN	asunder spermatogenesis regulator	0,003262	-1,64	0,024596	-1,53	0,817286	-1,07	0,137078	-1,28	0,171363	1,19	0,041219	1,28
11725722_at	29028	ATAD2	ATPase family, AAA domain containing 2	0,003025	-2,07	0,034532	-1,79	0,729609	-1,16	0,000204	-2,60	0,047563	-1,45	0,21911	-1,25
11756885_a_at	29028	ATAD2	ATPase family, AAA domain containing 2	0,002074	-2,12	0,011439	-2,01	0,910391	-1,06	0,002755	-2,07	0,886569	-1,03	0,915911	1,02
11726883_s_at	54454	ATAD2B	ATPase family, AAA domain containing 2B	0,003508	-1,59	0,012248	-1,57	0,97894	-1,01	0,00922	-1,51	0,753166	1,04	0,692007	1,05
11736919_at	79915	ATAD5	ATPase family, AAA domain containing 5	0,004086	-1,79	0,043254	-1,59	0,735255	-1,13	0,004461	-1,79	0,477188	-1,12	0,991735	1,00
11751694_a_at	466	ATF1	activating transcription factor 1	0,013878	-1,58	0,025561	-1,62	0,960011	1,02	0,321605	1,21	1,35E-05	1,96	4,67E-06	1,92
11743163_at	466	ATF1	activating transcription factor 1	0,001707	-1,86	0,004516	-1,91	0,950341	1,03	0,862279	-1,04	8,33E-05	1,84	4,18E-05	1,79
11743164_x_at	466	ATF1	activating transcription factor 1	0,00221	-1,84	0,004293	-1,93	0,900487	1,05	0,848613	-1,04	8,31E-05	1,85	7,56E-05	1,76
11763586_s_at	1386	ATF2	activating transcription factor 2	0,000901	-1,98	0,008123	-1,83	0,832557	-1,08	0,001436	-1,90	0,822098	-1,04	0,83176	1,04
11726659_a_at	84938	ATG4C	autophagy related 4C, cysteine peptidase	0,001265	-1,90	0,01631	-1,70	0,74147	-1,12	0,022605	-1,55	0,561628	1,10	0,159472	1,23
11758965_at	84938	ATG4C	autophagy related 4C, cysteine peptidase	0,020204	-1,52	0,049816	-1,51	0,981793	-1,01	0,707565	-1,08	0,019264	1,40	0,009747	1,41
11741374_x_at	472	ATM	ATM serine/threonine kinase	0,003808	-1,80	0,004657	-1,96	0,81597	1,09	0,073686	-1,43	0,044419	1,37	0,127135	1,26
11739434_a_at	23200	ATP11B	ATPase, class VI, type 11B	0,011241	-1,46	0,015844	-1,51	0,898129	1,04	0,922508	-1,02	0,00078	1,49	0,000936	1,43
11739433_s_at	23200	ATP11B	ATPase, class VI, type 11B	0,001241	-1,57	0,006687	-1,53	0,933803	-1,02	0,001775	-1,54	0,963878	-1,01	0,885863	1,02
11723847_a_at	286410	ATP11C	ATPase, class VI, type 11C	0,002592	-1,77	0,017938	-1,65	0,848913	-1,07	0,000527	-1,96	0,249401	-1,18	0,487834	-1,11
11752072_a_at	27032	ATP2C1	ATPase, Ca++ transporting, type 2C, member 1	0,005764	-1,65	0,019306	-1,62	0,967817	-1,02	0,566668	-1,12	0,008107	1,45	0,002892	1,48
11755057_s_at	27032	ATP2C1	ATPase, Ca++ transporting, type 2C, member 1	0,002567	-1,60	0,016872	-1,52	0,86157	-1,05	0,118109	-1,27	0,131251	1,20	0,038724	1,26
11747379_x_at	10159	ATP6AP2	ATPase, H+ transporting, lysosomal accessory protein 2	0,042994	-1,39	0,029078	-1,51	0,776952	1,09	0,486622	1,13	6,55E-05	1,70	0,00021	1,56
11747378_a_at	10159	ATP6AP2	ATPase, H+ transporting, lysosomal accessory protein 2	0,027413	-1,48	0,026306	-1,58	0,846549	1,07	0,804439	1,05	0,000406	1,66	0,000768	1,55
11715643_a_at	10159	ATP6AP2	ATPase, H+ transporting, lysosomal accessory protein 2	0,026849	-1,62	0,03134	-1,73	0,889802	1,06	0,893104	1,03	0,000987	1,79	0,001326	1,68
11750028_a_at	10159	ATP6AP2	ATPase, H+ transporting, lysosomal accessory protein 2	0,034821	-1,54	0,032611	-1,67	0,845141	1,08	0,882722	1,04	0,000999	1,73	0,002043	1,60

11718120_at	523	ATP6V1A	ATPase, H+ transporting, lysosomal 70kDa, V1 subunit A	0,000578	-2,12	0,01671	-1,77	0,603971	-1,20	3,98E-05	-2,53	0,024892	-1,44	0,265021	-1,19
11718121_x_at	523	ATP6V1A	ATPase, H+ transporting, lysosomal 70kDa, V1 subunit A	0,000458	-2,07	0,013587	-1,74	0,604538	-1,19	5,77E-05	-2,34	0,051224	-1,34	0,42648	-1,13
11751178_a_at	523	ATP6V1A	ATPase, H+ transporting, lysosomal 70kDa, V1 subunit A	0,000939	-1,92	0,006793	-1,81	0,879559	-1,06	0,000125	-2,17	0,226713	-1,20	0,403521	-1,13
11722181_a_at	528	ATP6V1C1	ATPase, H+ transporting, lysosomal 42kDa, V1 subunit C1	0,003987	-1,47	0,007754	-1,51	0,924842	1,03	0,540735	-1,09	0,001821	1,38	0,001847	1,35
11745272_a_at	528	ATP6V1C1	ATPase, H+ transporting, lysosomal 42kDa, V1 subunit C1	0,011845	-1,50	0,019605	-1,54	0,933825	1,03	0,360838	-1,16	0,026019	1,32	0,033313	1,29
11725568_a_at	10396	ATP8A1	ATPase, aminophospholipid transporter (APLT), class I, type 8A, member 1	0,009904	-1,54	0,019115	-1,57	0,957425	1,02	0,000216	-1,94	0,111321	-1,23	0,064707	-1,26
11743838_a_at	545	ATR	ATR serine/threonine kinase	0,002842	-1,68	0,021594	-1,56	0,820873	-1,07	2,36E-05	-2,21	0,009007	-1,41	0,025995	-1,32
11754247_s_at	546	ATRX	alpha thalassemia/mental retardation syndrome X-linked	0,000539	-2,20	0,005317	-2,02	0,832154	-1,09	4,84E-06	-3,04	0,014814	-1,50	0,039848	-1,38
11721714_s_at	546	ATRX	alpha thalassemia/mental retardation syndrome X-linked	0,002992	-1,88	0,030866	-1,67	0,751938	-1,12	0,000154	-2,33	0,041886	-1,39	0,174582	-1,24
11726361_a_at	4287	ATXN3	ataxin 3	2,68E-05	-1,82	0,001088	-1,65	0,652646	-1,10	0,000293	-1,61	0,854011	1,02	0,214556	1,13
11744091_s_at	552889	ATXN7L3B	ataxin 7-like 3B	0,000779	-2,11	0,014153	-1,82	0,687257	-1,16	0,000171	-2,33	0,130904	-1,28	0,553517	-1,11
11757739_s_at	6791 /// 25782	AURKAPS1 /// RAB3GAP2	aurora kinase A pseudogene 1 /// RAB3 GTPase activating protein subunit 2 (non-catalyti	0,000569	-1,52	0,001085	-1,58	0,852943	1,04	2,04E-05	-1,71	0,372826	-1,09	0,155406	-1,13
11721949_s_at	6791 /// 25782	AURKAPS1 /// RAB3GAP2	aurora kinase A pseudogene 1 /// RAB3 GTPase activating protein subunit 2 (non-catalyti	0,00208	-1,78	0,029178	-1,58	0,701968	-1,13	4,17E-05	-2,27	0,010545	-1,44	0,072913	-1,28
11722010_a_at	64343	AZI2	5-azacytidine induced 2	0,001583	-1,46	0,002897	-1,51	0,864073	1,04	0,007496	-1,37	0,266148	1,11	0,486774	1,07
11731011_a_at	8706	B3GALNT1	beta-1,3-N-acetylgalactosaminyltransferase 1 (globoside blood group)	0,006613	-1,55	0,015452	-1,56	0,976593	1,01	0,473726	1,13	1,53E-05	1,76	4,29E-06	1,74
11737780_a_at	8706	B3GALNT1	beta-1,3-N-acetylgalactosaminyltransferase 1 (globoside blood group)	0,00063	-2,03	0,003842	-1,96	0,936704	-1,03	0,10555	-1,37	0,019268	1,43	0,006009	1,48
11731842_a_at	9331	B4GALT6	UDP-Gal:betaGlcNAc beta 1,4-galactosyltransferase, polypeptide 6	0,009453	-1,87	0,033173	-1,80	0,941613	-1,04	0,797347	-1,07	0,006259	1,68	0,001558	1,74
11753060_a_at	55024	BANK1	B-cell scaffold protein with ankyrin repeats 1	0,239249	-1,29	0,030651	-1,72	0,47436	1,33	0,040717	-1,56	0,613586	1,10	0,262888	-1,21
11756612_a_at	580	BARD1	BRCA1 associated RING domain 1	0,013183	-1,56	0,027324	-1,57	0,979242	1,01	8,02E-05	-2,16	0,024713	-1,37	0,012871	-1,39
11758327_s_at	11177	BAZ1A	bromodomain adjacent to zinc finger domain, 1A	0,00056	-1,60	0,001959	-1,63	0,961653	1,01	0,000872	-1,56	0,729613	1,04	0,829719	1,03

11746692_a_at	11177	BAZ1A	bromodomain adjacent to zinc finger domain, 1A	0,009236	-1,79	0,008332	-1,99	0,789112	1,11	0,486141	-1,18	0,002875	1,69	0,010417	1,52
11720443_s_at	11177	BAZ1A	bromodomain adjacent to zinc finger domain, 1A	0,008799	-1,59	0,018708	-1,61	0,970556	1,01	0,020935	-1,51	0,648689	1,07	0,723985	1,06
11716388_a_at	9031	BAZ1B	bromodomain adjacent to zinc finger domain, 1B	0,001118	-1,91	0,009764	-1,78	0,831445	-1,08	0,020223	-1,56	0,408116	1,14	0,157951	1,23
11748950_a_at	29994	BAZ2B	bromodomain adjacent to zinc finger domain, 2B	0,000225	-2,31	0,004362	-2,03	0,722746	-1,14	5,07E-06	-2,95	0,021924	-1,45	0,119278	-1,28
11755706_a_at	56987	BBX	bobby sox homolog (Drosophila)	9,85E-06	-2,19	0,000274	-2,01	0,757202	-1,09	2,88E-07	-2,49	0,071158	-1,24	0,263313	-1,14
11720656_a_at	56987	BBX	bobby sox homolog (Drosophila)	0,000988	-1,88	0,003603	-1,89	0,989357	1,01	1,39E-05	-2,43	0,080395	-1,29	0,058163	-1,29
11720654_a_at	56987	BBX	bobby sox homolog (Drosophila)	7,73E-05	-1,89	0,001217	-1,77	0,813877	-1,06	0,000156	-1,80	0,916978	-1,01	0,697454	1,05
11720653_a_at	56987	BBX	bobby sox homolog (Drosophila)	0,010464	-1,57	0,018871	-1,61	0,946556	1,03	2,85E-05	-2,26	0,014298	-1,40	0,004609	-1,44
11720655_s_at	56987	BBX	bobby sox homolog (Drosophila)	0,000395	-1,72	0,005767	-1,59	0,750014	-1,08	0,000354	-1,71	0,524382	-1,08	0,972186	1,00
11722591_s_at	55973	BCAP29	B-cell receptor-associated protein 29	0,000351	-1,61	0,00236	-1,58	0,938277	-1,02	0,948202	-1,01	1,07E-05	1,56	8,77E-07	1,60
11742764_at	10286	BCAS2	breast carcinoma amplified sequence 2	0,001377	-1,53	0,007178	-1,50	0,938138	-1,02	0,000197	-1,67	0,304225	-1,11	0,404956	-1,09
11720566_at	586	BCAT1	branched chain amino-acid transaminase 1, cytosolic	0,000428	-2,20	0,004216	-2,04	0,84045	-1,08	0,000253	-2,25	0,560523	-1,11	0,908183	-1,02
11720478_at	56647	BCCIP	BRCA2 and CDKN1A interacting protein	0,001181	-1,68	0,010541	-1,58	0,827229	-1,06	0,02795	-1,40	0,337574	1,13	0,116391	1,20
11732339_at	53335	BCL11A	B-cell CLL/lymphoma 11A (zinc finger protein)	0,026631	-1,47	0,03761	-1,51	0,928072	1,03	7,11E-05	-2,12	0,013235	-1,40	0,003468	-1,45
11732340_at	53335	BCL11A	B-cell CLL/lymphoma 11A (zinc finger protein)	0,001482	-2,24	0,004973	-2,25	0,991103	1,01	0,000229	-2,61	0,461479	-1,16	0,427401	-1,17
11745968_x_at	9774	BCLAF1	BCL2-associated transcription factor 1	2,68E-05	-4,53	0,002854	-3,12	0,500471	-1,45	0,006128	-2,40	0,305893	1,30	0,005735	1,89
11748597_s_at	9774	BCLAF1	BCL2-associated transcription factor 1	0,000286	-2,43	0,005981	-2,08	0,693021	-1,17	0,116776	-1,43	0,033354	1,46	0,001235	1,70
11728218_a_at	9774	BCLAF1	BCL2-associated transcription factor 1	0,000953	-2,43	0,008688	-2,19	0,8261	-1,11	0,184371	-1,41	0,02739	1,55	0,003458	1,72
11745967_x_at	9774	BCLAF1	BCL2-associated transcription factor 1	0,00109	-2,65	0,005472	-2,55	0,948944	-1,04	0,126916	-1,55	0,025644	1,65	0,009902	1,71
11728216_x_at	9774	BCLAF1	BCL2-associated transcription factor 1	0,00061	-2,53	0,019803	-1,98	0,579872	-1,28	0,010591	-1,93	0,915274	1,03	0,16367	1,31
11728217_a_at	9774	BCLAF1	BCL2-associated transcription factor 1	0,013308	-1,68	0,022478	-1,74	0,942867	1,03	0,034358	-1,56	0,54866	1,11	0,68468	1,08
11721969_s_at	55814	BDP1	B double prime 1, subunit of RNA polymerase III transcription initiation factor IIIB	0,007659	-1,89	0,037213	-1,76	0,884506	-1,07	0,000297	-2,49	0,063183	-1,41	0,120867	-1,32

11719816_s_at	10282	BET1	Bet1 golgi vesicular membrane trafficking protein	0,008352	-1,62	0,011054	-1,71	0,872018	1,06	0,035846	-1,47	0,286458	1,17	0,503914	1,10
11719815_at	10282	BET1	Bet1 golgi vesicular membrane trafficking protein	0,014465	-1,54	0,018025	-1,62	0,882954	1,05	0,018737	-1,52	0,672425	1,07	0,942457	1,01
11744694_a_at	329	BIRC2	baculoviral IAP repeat containing 2	0,001484	-1,59	0,003211	-1,65	0,900145	1,04	0,986478	1,00	1,40E-05	1,66	1,01E-05	1,60
11717628_a_at	329	BIRC2	baculoviral IAP repeat containing 2	0,000862	-2,54	0,003069	-2,58	0,978548	1,02	0,735789	-1,10	7,62E-05	2,34	2,60E-05	2,30
11757937_s_at	329	BIRC2	baculoviral IAP repeat containing 2	0,000802	-1,57	0,005022	-1,52	0,915388	-1,03	0,768326	-1,04	0,000196	1,46	1,81E-05	1,50
11750887_a_at	329	BIRC2	baculoviral IAP repeat containing 2	0,002826	-1,81	0,005438	-1,89	0,911451	1,04	0,994244	-1,00	5,45E-05	1,89	4,16E-05	1,80
11717627_a_at	329	BIRC2	baculoviral IAP repeat containing 2	0,012077	-1,51	0,015922	-1,58	0,88756	1,05	0,754241	1,06	0,000103	1,67	0,000113	1,60
11718093_x_at	54841	BIVM	basic, immunoglobulin-like variable motif containing	0,000135	-2,25	0,004134	-1,94	0,650962	-1,16	0,018895	-1,58	0,180658	1,23	0,01135	1,43
11747384_x_at	54841	BIVM	basic, immunoglobulin-like variable motif containing	0,000469	-2,15	0,010091	-1,85	0,66708	-1,17	0,049177	-1,50	0,194853	1,23	0,014909	1,44
11747383_a_at	54841	BIVM	basic, immunoglobulin-like variable motif containing	0,000418	-2,30	0,014928	-1,86	0,572433	-1,24	0,039583	-1,57	0,348786	1,18	0,017805	1,47
11752129_a_at	2073 /// 100533467	BIVM-ERCC5 /// ERCC5	BIVM-ERCC5 readthrough /// excision repair cross-complementation group 5	0,005169	-1,58	0,024928	-1,52	0,900222	-1,04	0,594868	-1,10	0,010772	1,38	0,00201	1,44
11756409_a_at	641	BLM	Bloom syndrome, RecQ helicase-like	0,003475	-1,66	0,023201	-1,56	0,843009	-1,07	0,002821	-1,68	0,585019	-1,08	0,925991	-1,02
11728393_a_at	63915 /// 100526837	BLOC1S5 /// EEF1E1-BLOC1S5	biogenesis of lysosomal organelles complex-1, subunit 5, muted /// EEF1E1-BLOC1S5 readt	0,002316	-1,90	0,030912	-1,66	0,707267	-1,14	0,150979	-1,34	0,196932	1,23	0,021506	1,41
11715395_a_at	81567 /// 100526836	BLOC1S5-TXNDC5 /// TXNDC5	BLOC1S5-TXNDC5 readthrough (NMD candidate) /// thioredoxin domain containing 5 (endopla	0,01496	-2,57	0,031532	-2,62	0,985079	1,02	0,013973	-2,63	0,987713	-1,01	0,953009	-1,02
11719807_x_at	26258	BLOC1S6	biogenesis of lysosomal organelles complex-1, subunit 6, pallidin	1,92E-06	-3,05	4,21E-05	-2,86	0,862912	-1,07	0,000207	-2,13	0,047013	1,35	0,009727	1,43
11719808_a_at	26258	BLOC1S6	biogenesis of lysosomal organelles complex-1, subunit 6, pallidin	0,001127	-1,93	0,031734	-1,61	0,586975	-1,20	0,012844	-1,62	0,957994	-1,01	0,24707	1,19
11759556_at	8548	BLZF1	basic leucine zipper nuclear factor 1	0,000708	-2,00	0,004134	-1,94	0,940338	-1,03	0,250567	-1,25	0,004262	1,55	0,000955	1,59
11755068_s_at	259282	BOD1L1	biorientation of chromosomes in cell division 1-like 1	0,007247	-1,72	0,02622	-1,67	0,945043	-1,03	0,000419	-2,11	0,133293	-1,27	0,174058	-1,23

11728238_x_at	23049 /// 552900 /// 101060386	BOLA2 /// LOC101060386 /// SMG1	bolA family member 2 /// serine/threonine-protein kinase SMG1-like /// SMG1 phosphatidy	0,000128	-2,85	0,003786	-2,36	0,662193	-1,21	0,000248	-2,63	0,607367	-1,11	0,70276	1,08
11752230_a_at	2186	BPTF	bromodomain PHD finger transcription factor	0,000102	-2,39	0,000459	-2,42	0,976671	1,01	2,76E-05	-2,56	0,765877	-1,05	0,700267	-1,07
11740211_s_at	2186 /// 146880 /// 102464831	BPTF /// LOC146880 /// MIR6080	bromodomain PHD finger transcription factor /// Rho GTPase activating protein 27 pseudo	0,001041	-1,70	0,004068	-1,70	0,99809	-1,00	0,000102	-1,92	0,337558	-1,13	0,324233	-1,13
11744317_x_at	672	BRCA1	breast cancer 1, early onset	0,000986	-1,91	0,025063	-1,61	0,60606	-1,18	0,000133	-2,15	0,04786	-1,34	0,406207	-1,13
11726531_x_at	79184	BRCC3	BRCA1/BRCA2-containing complex, subunit 3	1,32E-05	-2,33	0,000257	-2,19	0,840717	-1,07	3,61E-07	-2,72	0,095142	-1,25	0,223848	-1,17
11733211_x_at	79184	BRCC3	BRCA1/BRCA2-containing complex, subunit 3	0,000347	-2,16	0,003156	-2,03	0,872081	-1,06	0,000808	-2,01	0,95146	1,01	0,661513	1,08
11750003_s_at	79184	BRCC3	BRCA1/BRCA2-containing complex, subunit 3	0,001019	-1,93	0,018349	-1,68	0,676378	-1,15	0,003828	-1,76	0,78645	-1,05	0,552442	1,10
11748918_a_at	55299	BRIX1	BRX1, biogenesis of ribosomes, homolog (S. cerevisiae)	0,002316	-1,76	0,02918	-1,57	0,718524	-1,12	0,000154	-2,09	0,045218	-1,33	0,222877	-1,18
11722989_a_at	9044	BTAF1	BTAF1 RNA polymerase II, B-TFIID transcription factor-associated, 170kDa	0,005275	-1,85	0,037044	-1,69	0,813877	-1,10	0,002114	-2,00	0,335363	-1,19	0,681026	-1,08
11722990_s_at	9044	BTAF1	BTAF1 RNA polymerase II, B-TFIID transcription factor-associated, 170kDa	0,007267	-1,76	0,038115	-1,65	0,868424	-1,07	0,012696	-1,69	0,878322	-1,03	0,841849	1,04
11758492_x_at	55727	BTBD7	BTB (POZ) domain containing 7	0,000175	-1,58	0,001362	-1,55	0,930488	-1,02	4,28E-06	-1,79	0,093806	-1,16	0,133241	-1,13
11721803_a_at	9184	BUB3	BUB3 mitotic checkpoint protein	0,000975	-1,73	0,004909	-1,69	0,951003	-1,02	0,114849	-1,28	0,025339	1,32	0,009936	1,35
11748735_s_at	9689	BZW1	basic leucine zipper and W2 domains 1	0,003132	-1,75	0,012394	-1,71	0,960293	-1,02	0,205698	-1,27	0,038328	1,35	0,018044	1,38
11747433_a_at	28970	C11orf54	chromosome 11 open reading frame 54	0,004549	-1,66	0,031099	-1,54	0,827478	-1,07	0,133361	-1,30	0,228376	1,18	0,066369	1,27
11743153_at	29035	C16orf72	chromosome 16 open reading frame 72	0,051657	-1,50	0,04386	-1,63	0,834093	1,09	0,85063	1,05	0,00148	1,70	0,003492	1,57
11729886_at	162681	C18orf54	chromosome 18 open reading frame 54	0,00211	-1,80	0,029372	-1,59	0,703882	-1,13	0,013805	-1,59	0,98798	1,00	0,389254	1,14
11722431_a_at	54953	C1orf27	chromosome 1 open reading frame 27	0,000268	-1,87	0,008123	-1,63	0,621903	-1,14	0,075677	-1,32	0,091861	1,23	0,00282	1,41
11748778_a_at	54953	C1orf27	chromosome 1 open reading frame 27	0,000341	-1,71	0,005405	-1,57	0,73629	-1,09	0,000999	-1,60	0,873335	-1,02	0,590196	1,06

11722432_at	54953	C1orf27	chromosome 1 open reading frame 27	0,002955	-2,04	0,022282	-1,85	0,821308	-1,10	0,016161	-1,77	0,819829	1,05	0,440618	1,16
11725804_a_at	54149	C21orf91	chromosome 21 open reading frame 91	0,000687	-2,52	0,002765	-2,54	0,98832	1,01	0,005091	-2,08	0,337806	1,22	0,345435	1,21
11745149_a_at	9847	C2CD5	C2 calcium-dependent domain containing 5	0,002847	-1,85	0,024016	-1,68	0,79684	-1,10	0,024418	-1,57	0,700426	1,07	0,307237	1,17
11758647_s_at	9847	C2CD5	C2 calcium-dependent domain containing 5	0,011801	-1,55	0,042035	-1,50	0,930091	-1,03	0,002268	-1,73	0,311736	-1,15	0,42963	-1,12
11737765_a_at	25871	C3orf17	chromosome 3 open reading frame 17	0,000854	-1,97	0,004134	-1,94	0,967955	-1,02	0,048231	-1,47	0,065201	1,32	0,037012	1,35
11749950_a_at	25871	C3orf17	chromosome 3 open reading frame 17	0,001143	-1,94	0,002799	-2,03	0,912401	1,04	0,031584	-1,52	0,061047	1,33	0,09662	1,27
11748000_a_at	25871	C3orf17	chromosome 3 open reading frame 17	0,002291	-1,86	0,008612	-1,84	0,978096	-1,01	0,075677	-1,42	0,099614	1,29	0,067576	1,31
11741631_a_at	205428	C3orf58	chromosome 3 open reading frame 58	0,002547	-1,54	0,001662	-1,71	0,670731	1,11	0,194786	-1,20	0,001434	1,42	0,014731	1,28
11730176_x_at	401152	C4orf3	chromosome 4 open reading frame 3	9,74E-05	-1,79	0,000275	-1,86	0,881486	1,04	0,000387	-1,66	0,282587	1,12	0,482867	1,08
11730175_at	401152	C4orf3	chromosome 4 open reading frame 3	0,000688	-1,66	0,001124	-1,76	0,827787	1,06	0,005724	-1,49	0,132612	1,18	0,316193	1,12
11728834_x_at	132720	C4orf32	chromosome 4 open reading frame 32	0,000427	-1,71	0,008949	-1,54	0,673597	-1,11	0,016805	-1,40	0,429283	1,10	0,06224	1,22
11723224_s_at	79624	C6orf211	chromosome 6 open reading frame 211	0,003386	-1,74	0,019177	-1,65	0,883291	-1,06	0,058686	-1,42	0,319817	1,16	0,148074	1,22
11723621_at	154743	C7orf60	chromosome 7 open reading frame 60	0,007433	-1,66	0,039263	-1,56	0,865644	-1,06	0,00546	-1,70	0,580047	-1,09	0,879911	-1,03
11724103_x_at	23678 /// 100533105	C8orf44-SGK3 /// SGK3	C8orf44-SGK3 readthrough /// serum/glucocorticoid regulated kinase family, member 3	8,22E-05	-2,94	0,001179	-2,66	0,827962	-1,10	1,27E-05	-3,34	0,252218	-1,25	0,529883	-1,13
11759202_s_at	138199	C9orf41	chromosome 9 open reading frame 41	0,00056	-1,97	0,027399	-1,60	0,517637	-1,24	0,001558	-1,83	0,358324	-1,15	0,637471	1,08
11747183_a_at	143384	CACUL1	CDK2-associated, cullin domain 1	0,000555	-1,93	0,008572	-1,73	0,734733	-1,11	0,00057	-1,91	0,509613	-1,10	0,957922	1,01
11739218_a_at	143384	CACUL1	CDK2-associated, cullin domain 1	0,001465	-1,55	0,006785	-1,53	0,954808	-1,02	0,006703	-1,44	0,614879	1,06	0,49622	1,08
11725308_a_at	813	CALU	calumenin	0,001199	-1,67	0,015175	-1,53	0,748435	-1,09	0,568985	-1,10	0,005205	1,40	0,000169	1,52
11719937_a_at	55832	CAND1	cullin-associated and neddylation-dissociated 1	0,007313	-1,49	0,015933	-1,51	0,96775	1,01	0,678342	-1,07	0,003141	1,41	0,00213	1,39
11719939_a_at	55832	CAND1	cullin-associated and neddylation-dissociated 1	0,001879	-1,74	0,021208	-1,58	0,750208	-1,10	0,001576	-1,76	0,450003	-1,11	0,952934	-1,01
11719938_s_at	55832	CAND1	cullin-associated and neddylation-dissociated 1	0,003899	-1,85	0,033712	-1,66	0,782391	-1,11	0,062287	-1,48	0,494843	1,13	0,1605	1,25
11723567_s_at	23473	CAPN7	calpain 7	0,001583	-1,91	0,004473	-1,96	0,959499	1,02	0,034173	-1,52	0,108572	1,28	0,126798	1,26

11723569_a_at	23473	CAPN7	calpain 7	0,001237	-1,68	0,00832	-1,61	0,890882	-1,04	0,002003	-1,64	0,917212	-1,02	0,845708	1,03
11720455_a_at	4076	CAPRIN1	cell cycle associated protein 1	0,008448	-1,73	0,027543	-1,69	0,958332	-1,02	0,769205	-1,07	0,005275	1,58	0,001526	1,62
11739007_at	829	CAPZA1	capping protein (actin filament) muscle Z-line, alpha 1	0,018954	-2,02	0,035979	-2,07	0,973557	1,02	0,983811	1,01	0,002182	2,08	0,001292	2,04
11756721_a_at	9994	CASP8AP2	caspase 8 associated protein 2	0,003461	-1,90	0,017157	-1,81	0,911129	-1,05	0,000936	-2,10	0,389008	-1,16	0,56577	-1,11
11734716_at	79872	CBLL1	Cbl proto-oncogene-like 1, E3 ubiquitin protein ligase	0,001184	-2,17	0,008899	-2,01	0,865498	-1,08	0,363685	1,24	1,85E-06	2,50	5,77E-08	2,69
11742972_x_at	55749	CCAR1	cell division cycle and apoptosis regulator 1	0,001083	-1,73	0,018289	-1,54	0,689241	-1,12	5,91E-05	-2,01	0,033523	-1,30	0,207912	-1,17
11756908_a_at	55610	CCDC132	coiled-coil domain containing 132	0,010554	-1,49	0,011492	-1,58	0,837238	1,06	0,348746	-1,16	0,011852	1,36	0,030718	1,28
11754530_a_at	64770	CCDC14	coiled-coil domain containing 14	0,005431	-1,95	0,037016	-1,77	0,819508	-1,10	0,006672	-1,93	0,670577	-1,09	0,956314	1,01
11722125_a_at	51244	CCDC174	coiled-coil domain containing 174	0,002463	-1,59	0,017249	-1,50	0,848656	-1,06	0,000154	-1,83	0,092643	-1,22	0,20957	-1,15
11758016_s_at	91057	CCDC34	coiled-coil domain containing 34	0,004121	-1,61	0,029748	-1,50	0,819872	-1,07	0,000675	-1,79	0,17512	-1,19	0,409075	-1,11
11754718_a_at	57003	CCDC47	coiled-coil domain containing 47	0,00022	-2,12	0,009234	-1,76	0,569752	-1,20	0,215883	-1,26	0,022947	1,39	0,000168	1,67
11736416_s_at	79780	CCDC82	coiled-coil domain containing 82	0,000495	-1,84	0,004169	-1,75	0,877754	-1,05	0,000429	-1,84	0,726183	-1,05	0,999504	-1,00
11744443_x_at	79780	CCDC82	coiled-coil domain containing 82	0,001142	-1,66	0,004896	-1,64	0,978314	-1,01	0,001774	-1,61	0,909783	1,02	0,85416	1,03
11736417_a_at	79780	CCDC82	coiled-coil domain containing 82	0,001796	-1,62	0,006037	-1,63	0,997545	1,00	0,02209	-1,41	0,240579	1,15	0,22638	1,15
11744442_at	79780	CCDC82	coiled-coil domain containing 82	0,001437	-1,75	0,01343	-1,62	0,807519	-1,08	0,002419	-1,69	0,775911	-1,04	0,83293	1,03
11739606_x_at	55704	CCDC88A	coiled-coil domain containing 88A	0,007634	-1,66	0,040529	-1,56	0,863369	-1,06	8,87E-05	-2,25	0,014895	-1,44	0,03197	-1,35
11743939_a_at	60492	CCDC90B	coiled-coil domain containing 90B	0,000284	-1,54	0,002301	-1,50	0,913557	-1,02	0,000857	-1,46	0,795628	1,03	0,586303	1,05
11725156_x_at	55297	CCDC91	coiled-coil domain containing 91	0,003895	-1,68	0,016867	-1,63	0,935703	-1,03	0,000239	-1,99	0,142706	-1,23	0,197055	-1,19
11726985_x_at	892	CCNC	cyclin C	0,01035	-1,77	0,023448	-1,79	0,987633	1,01	0,598363	-1,14	0,009596	1,58	0,006224	1,56
11740980_x_at	900	CCNG1	cyclin G1	0,004428	-1,87	0,01596	-1,83	0,965336	-1,02	0,944638	1,02	0,000341	1,86	6,38E-05	1,90
11715674_s_at	900	CCNG1	cyclin G1	0,002589	-1,62	0,023192	-1,50	0,788695	-1,08	0,786831	-1,05	0,003518	1,43	0,000154	1,55
11749307_x_at	900	CCNG1	cyclin G1	0,003889	-2,33	0,014971	-2,25	0,958708	-1,03	0,664591	-1,15	0,003005	1,97	0,000766	2,03
11749306_a_at	900	CCNG1	cyclin G1	0,004457	-2,40	0,019034	-2,27	0,932241	-1,06	0,516443	-1,23	0,010324	1,84	0,002644	1,94
11715675_a_at	900	CCNG1	cyclin G1	0,010776	-2,43	0,047033	-2,21	0,891739	-1,10	0,859705	-1,07	0,008348	2,06	0,001317	2,26
11759759_s_at	57018	CCNL1	cyclin L1	0,000712	-2,53	0,005022	-2,36	0,896694	-1,07	0,003664	-2,16	0,68783	1,09	0,449367	1,17
11745826_a_at	57018	CCNL1	cyclin L1	0,012426	-2,01	0,019929	-2,11	0,930091	1,05	0,038063	-1,79	0,467042	1,18	0,620328	1,12
11728369_x_at	9738	CCP110	centriolar coiled coil protein 110kDa	1,16E-05	-3,08	0,000459	-2,61	0,669716	-1,18	2,88E-07	-3,79	0,030828	-1,45	0,217952	-1,23
11728368_s_at	9738	CCP110	centriolar coiled coil protein 110kDa	0,003337	-1,85	0,018724	-1,74	0,886462	-1,06	0,000179	-2,28	0,095126	-1,31	0,177514	-1,23

11745800_a_at	9738	CCP110	centriolar coiled coil protein 110kDa	0,001109	-1,99	0,005343	-1,94	0,957642	-1,02	0,028054	-1,56	0,169242	1,25	0,107901	1,28
11715420_a_at	10576	CCT2	chaperonin containing TCP1, subunit 2 (beta)	0,006025	-2,08	0,047857	-1,82	0,780937	-1,14	0,07908	-1,59	0,542562	1,14	0,185098	1,31
11724033_s_at	23607	CD2AP	CD2-associated protein	0,000501	-2,07	0,006021	-1,88	0,791394	-1,10	9,51E-05	-2,29	0,211905	-1,21	0,5279	-1,11
11715915_a_at	960	CD44	CD44 molecule (Indian blood group)	0,05139	-1,43	0,015825	-1,68	0,614899	1,18	0,876513	1,03	0,000218	1,74	0,004038	1,48
11742219_a_at	4179	CD46	CD46 molecule, complement regulatory protein	0,006926	-2,07	0,01873	-2,06	0,998101	-1,00	0,727873	1,11	0,000125	2,29	3,24E-05	2,29
11751532_x_at	4179	CD46	CD46 molecule, complement regulatory protein	0,004799	-1,86	0,031322	-1,71	0,83691	-1,09	0,781246	1,07	0,000502	1,83	2,30E-05	1,99
11743903_a_at	4179	CD46	CD46 molecule, complement regulatory protein	0,00287	-1,74	0,019234	-1,63	0,850546	-1,07	0,683399	-1,08	0,00433	1,51	0,000381	1,61
11735060_a_at	4179	CD46	CD46 molecule, complement regulatory protein	0,007702	-2,36	0,032423	-2,20	0,912671	-1,07	0,808859	-1,09	0,005679	2,01	0,001016	2,16
11743402_at	996	CDC27	cell division cycle 27	0,003014	-1,65	0,013334	-1,60	0,941155	-1,03	0,233946	-1,22	0,033778	1,31	0,012752	1,35
11720289_a_at	8476	CDC42BPA	CDC42 binding protein kinase alpha (DMPK-like)	0,00183	-2,07	0,036998	-1,71	0,633176	-1,21	0,058391	-1,53	0,554417	1,12	0,07486	1,35
11719081_a_at	988	CDC5L	cell division cycle 5-like	0,00096	-1,73	0,009357	-1,62	0,809057	-1,07	0,006694	-1,55	0,750893	1,05	0,361454	1,12
11719080_a_at	988	CDC5L	cell division cycle 5-like	0,002341	-1,85	0,030373	-1,63	0,713122	-1,14	0,095542	-1,39	0,314671	1,17	0,048393	1,33
11732058_a_at	8317	CDC7	cell division cycle 7	0,001482	-2,01	0,015955	-1,81	0,774248	-1,12	0,002727	-1,92	0,739002	-1,06	0,803849	1,05
11753163_a_at	79577	CDC73	cell division cycle 73	0,001539	-1,86	0,019641	-1,66	0,734674	-1,12	0,21785	-1,27	0,067346	1,31	0,005271	1,47
11755635_s_at	984 /// 728642	CDK11A /// CDK11B	cyclin-dependent kinase 11A /// cyclin-dependent kinase 11B	0,000479	-2,69	0,018093	-2,06	0,556151	-1,31	8,89E-05	-3,08	0,051042	-1,50	0,529745	-1,15
11719255_s_at	23097	CDK19	cyclin-dependent kinase 19	0,002767	-1,75	0,008106	-1,76	0,986166	1,01	0,002866	-1,75	0,961826	1,01	0,996276	1,00
11723244_at	1021	CDK6	cyclin-dependent kinase 6	0,00022	-2,32	0,002074	-2,19	0,891842	-1,06	1,26E-05	-2,79	0,146131	-1,27	0,25637	-1,20
11723243_at	1021	CDK6	cyclin-dependent kinase 6	0,00382	-1,76	0,02451	-1,64	0,849705	-1,07	0,000235	-2,12	0,087452	-1,29	0,197917	-1,21
11723242_at	1021	CDK6	cyclin-dependent kinase 6	0,010533	-2,03	0,041567	-1,91	0,912401	-1,06	0,7516	-1,10	0,011535	1,73	0,002495	1,84
11723241_at	1021	CDK6	cyclin-dependent kinase 6	0,010007	-1,88	0,031297	-1,83	0,961122	-1,03	0,034786	-1,68	0,682417	1,09	0,577208	1,12
11760818_x_at	51265	CDKL3	cyclin-dependent kinase-like 3	0,00194	-1,79	0,011926	-1,70	0,890082	-1,05	0,000102	-2,15	0,102651	-1,26	0,186527	-1,20
11723114_s_at	1060	CENPC	centromere protein C	0,000495	-1,92	0,013594	-1,65	0,61836	-1,16	0,002896	-1,71	0,830428	-1,03	0,398103	1,12
11723113_a_at	1060	CENPC	centromere protein C	0,001765	-1,65	0,02096	-1,50	0,74147	-1,09	0,158206	-1,24	0,118392	1,21	0,01329	1,32
11754425_a_at	55835	CENPJ	centromere protein J	0,002255	-1,71	0,017167	-1,60	0,832787	-1,07	0,000775	-1,82	0,342605	-1,14	0,656723	-1,07
11755168_x_at	91687	CENPL	centromere protein L	0,001927	-1,60	0,010969	-1,54	0,906393	-1,04	0,900258	1,02	0,000117	1,57	8,69E-06	1,63
11732601_at	9662	CEP135	centrosomal protein 135kDa	4,95E-05	-3,43	0,000652	-3,12	0,854454	-1,10	5,06E-06	-4,06	0,219384	-1,30	0,429497	-1,19
11754233_a_at	22995	CEP152	centrosomal protein 152kDa	0,001199	-1,98	0,013342	-1,78	0,777717	-1,11	0,000521	-2,09	0,333162	-1,17	0,751629	-1,06
11742422_a_at	55125	CEP192	centrosomal protein 192kDa	0,008018	-1,63	0,015496	-1,67	0,948456	1,03	0,001642	-1,82	0,587615	-1,09	0,457734	-1,12

11725230_a_at	80184	CEP290	centrosomal protein 290kDa	0,002874	-2,04	0,034702	-1,76	0,71967	-1,16	0,017796	-1,75	0,970831	1,01	0,402249	1,17
11719052_a_at	9857	CEP350	centrosomal protein 350kDa	0,000219	-2,03	0,000834	-2,06	0,969231	1,02	9,92E-07	-2,71	0,043984	-1,32	0,023197	-1,34
11758004_s_at	9857	CEP350	centrosomal protein 350kDa	0,000849	-1,82	0,00408	-1,79	0,969414	-1,01	6,85E-06	-2,38	0,033313	-1,33	0,031823	-1,31
11719053_s_at	9857	CEP350	centrosomal protein 350kDa	0,000837	-1,78	0,003328	-1,78	0,999903	1,00	1,04E-05	-2,26	0,066378	-1,27	0,051651	-1,27
11719054_at	9857	CEP350	centrosomal protein 350kDa	0,010642	-1,72	0,044168	-1,63	0,902312	-1,05	0,959102	1,01	0,002789	1,65	0,000367	1,74
11743544_a_at	9702	CEP57	centrosomal protein 57kDa	4,42E-05	-2,31	0,003163	-1,91	0,543817	-1,21	5,66E-05	-2,22	0,295162	-1,17	0,811351	1,04
11743541_a_at	9702	CEP57	centrosomal protein 57kDa	0,000137	-2,12	0,003328	-1,88	0,696115	-1,13	0,001847	-1,78	0,726549	1,06	0,194557	1,19
11748252_s_at	9702	CEP57	centrosomal protein 57kDa	0,00156	-1,67	0,006668	-1,64	0,967961	-1,01	0,007522	-1,53	0,568746	1,08	0,479833	1,09
11743542_s_at	9702	CEP57	centrosomal protein 57kDa	0,002037	-2,00	0,020571	-1,79	0,771179	-1,12	0,00207	-2,00	0,538288	-1,12	0,995496	1,00
11763342_s_at	9702	CEP57	centrosomal protein 57kDa	0,002101	-1,61	0,012555	-1,55	0,893296	-1,04	0,002306	-1,61	0,787766	-1,04	0,976353	1,00
11743543_x_at	9702	CEP57	centrosomal protein 57kDa	0,002577	-1,84	0,01671	-1,72	0,864402	-1,07	0,003673	-1,79	0,815338	-1,04	0,898358	1,02
11740958_x_at	80254	CEP63	centrosomal protein 63kDa	0,000273	-1,63	0,002415	-1,58	0,897695	-1,03	9,21E-05	-1,69	0,506294	-1,07	0,734479	-1,04
11724857_s_at	80254	CEP63	centrosomal protein 63kDa	0,001118	-1,57	0,004541	-1,56	0,990613	-1,00	0,000306	-1,66	0,589248	-1,06	0,609081	-1,06
11724856_a_at	80254	CEP63	centrosomal protein 63kDa	0,000835	-1,66	0,004254	-1,63	0,957817	-1,02	0,000475	-1,70	0,748754	-1,04	0,857219	-1,02
11760877_x_at	80321	CEP70	centrosomal protein 70kDa	0,000454	-2,03	0,028435	-1,61	0,48342	-1,27	0,175195	-1,29	0,148202	1,24	0,00106	1,57
11748356_a_at	80321	CEP70	centrosomal protein 70kDa	0,000707	-2,46	0,027897	-1,88	0,541087	-1,31	0,106883	-1,50	0,25988	1,25	0,007057	1,64
11748357_x_at	80321	CEP70	centrosomal protein 70kDa	0,001107	-2,22	0,024609	-1,83	0,63395	-1,21	0,117352	-1,44	0,206468	1,26	0,011993	1,54
11754581_s_at	253782	CERS6	ceramide synthase 6	0,000376	-1,60	0,002941	-1,56	0,905381	-1,03	2,40E-05	-1,78	0,170121	-1,14	0,277051	-1,11
11755520_s_at	253782	CERS6	ceramide synthase 6	0,003011	-1,75	0,029528	-1,58	0,763421	-1,10	0,389204	-1,18	0,041071	1,34	0,003365	1,48
11742903_at	1070	CETN3	centrin, EF-hand protein, 3	0,003519	-1,87	0,024433	-1,72	0,834468	-1,09	0,001512	-2,00	0,38856	-1,16	0,716374	-1,07
11742901_a_at	8545	CGGBP1	CGG triplet repeat binding protein 1	0,000304	-1,63	0,001894	-1,61	0,957486	-1,01	0,020483	-1,34	0,062199	1,20	0,031777	1,22
11742900_a_at	8545	CGGBP1	CGG triplet repeat binding protein 1	0,002415	-1,78	0,006902	-1,80	0,979986	1,01	0,302852	-1,22	0,00714	1,48	0,004787	1,47
11754992_a_at	1105	CHD1	chromodomain helicase DNA binding protein 1	0,01444	-1,53	0,040229	-1,51	0,970271	-1,01	0,035554	-1,45	0,782255	1,04	0,701235	1,06
11743464_a_at	1108	CHD4	chromodomain helicase DNA binding protein 4	0,00031	-1,98	0,003452	-1,84	0,830012	-1,07	0,000362	-1,93	0,756836	-1,05	0,892792	1,02
11743463_a_at	1108	CHD4	chromodomain helicase DNA binding protein 4	0,000376	-2,00	0,0055	-1,81	0,752589	-1,11	0,003619	-1,71	0,73159	1,06	0,267291	1,17
11754262_s_at	80205	CHD9	chromodomain helicase DNA binding protein 9	0,005248	-1,95	0,045339	-1,72	0,767343	-1,13	2,88E-05	-3,00	0,003174	-1,74	0,013807	-1,53
11754263_s_at	80205	CHD9	chromodomain helicase DNA binding protein 9	0,002078	-1,79	0,010419	-1,73	0,928973	-1,03	8,43E-05	-2,18	0,101701	-1,27	0,146331	-1,22
11759013_at	1111	CHEK1	checkpoint kinase 1	0,004425	-1,70	0,037298	-1,55	0,782959	-1,10	0,071273	-1,39	0,48317	1,11	0,155109	1,22
11723471_a_at	1121	CHM	choroideremia (Rab escort protein 1)	0,002596	-1,54	0,004254	-1,61	0,870569	1,05	0,000205	-1,74	0,49625	-1,08	0,254025	-1,13

11722935_x_at	1122	CHML	choroideremia-like (Rab escort protein 2)	0,000495	-2,23	0,015847	-1,82	0,587932	-1,22	4,12E-05	-2,64	0,027837	-1,45	0,315842	-1,18
11722934_s_at	1122	CHML	choroideremia-like (Rab escort protein 2)	0,000602	-2,09	0,020434	-1,71	0,572117	-1,22	6,50E-05	-2,41	0,031	-1,41	0,367703	-1,15
11743054_a_at	26973	CHORDC1	cysteine and histidine-rich domain (CHORD) containing 1	0,000256	-2,27	0,00283	-2,11	0,85001	-1,08	0,562154	-1,14	0,000192	1,86	8,41E-06	2,00
11742059_a_at	26973	CHORDC1	cysteine and histidine-rich domain (CHORD) containing 1	0,00208	-1,90	0,016415	-1,75	0,829712	-1,08	0,677446	-1,10	0,003323	1,60	0,000219	1,73
11742060_x_at	26973	CHORDC1	cysteine and histidine-rich domain (CHORD) containing 1	0,001645	-1,96	0,016052	-1,78	0,791814	-1,10	0,529115	-1,15	0,007175	1,55	0,000406	1,71
11743052_a_at	26973	CHORDC1	cysteine and histidine-rich domain (CHORD) containing 1	0,008941	-1,62	0,031724	-1,58	0,941396	-1,03	0,668538	-1,09	0,011054	1,45	0,003174	1,49
11721833_at	91612	CHURC1	churchill domain containing 1	0,000712	-3,62	0,004169	-3,41	0,939918	-1,06	0,000467	-3,77	0,753303	-1,10	0,903671	-1,04
11723651_x_at	9391	CIAO1	cytosolic iron-sulfur assembly component 1	3,70E-05	-1,87	0,001345	-1,70	0,674172	-1,10	0,000117	-1,75	0,796858	-1,03	0,538048	1,07
11723677_a_at	26586	CKAP2	cytoskeleton associated protein 2	0,001614	-2,03	0,005264	-2,04	0,989344	1,01	0,632284	-1,12	0,000496	1,82	0,000189	1,81
11748248_a_at	26586	CKAP2	cytoskeleton associated protein 2	0,006364	-1,98	0,019843	-1,95	0,977112	-1,02	0,975897	-1,01	0,000894	1,93	0,000228	1,96
11723678_at	26586	CKAP2	cytoskeleton associated protein 2	0,001023	-2,14	0,004757	-2,10	0,96775	-1,02	0,022817	-1,66	0,178278	1,27	0,123581	1,29
11764018_s_at	1182	CLCN3	chloride channel, voltage-sensitive 3	0,059662	-1,35	0,010568	-1,61	0,52198	1,20	0,112391	-1,29	0,077539	1,25	0,767206	1,04
11752627_a_at	1047	CLGN	calmegin	0,002866	-2,00	0,043204	-1,69	0,673091	-1,18	4,73E-05	-2,76	0,006284	-1,63	0,056614	-1,38
11749190_a_at	1047	CLGN	calmegin	0,004962	-1,93	0,041855	-1,71	0,775378	-1,13	0,000122	-2,61	0,020296	-1,52	0,079481	-1,35
11739345_x_at	1047	CLGN	calmegin	0,002576	-1,72	0,022569	-1,58	0,793379	-1,09	0,000152	-2,04	0,063459	-1,29	0,199463	-1,19
11756853_a_at	57396	CLK4	CDC-like kinase 4	0,006194	-1,83	0,030794	-1,72	0,887406	-1,06	0,203432	-1,33	0,132659	1,30	0,046894	1,38
11763757_a_at	10845	CLPX	caseinolytic mitochondrial matrix peptidase chaperone subunit	0,000348	-1,88	0,003459	-1,77	0,844885	-1,06	0,02215	-1,45	0,125685	1,22	0,032295	1,29
11740676_a_at	10845	CLPX	caseinolytic mitochondrial matrix peptidase chaperone subunit	0,000712	-1,70	0,007699	-1,59	0,79918	-1,07	0,010815	-1,46	0,508239	1,08	0,187195	1,16
11763746_a_at	10845	CLPX	caseinolytic mitochondrial matrix peptidase chaperone subunit	0,003252	-1,92	0,020398	-1,79	0,860536	-1,08	0,230977	-1,30	0,064776	1,37	0,014717	1,48
11763988_a_at	10845	CLPX	caseinolytic mitochondrial matrix peptidase chaperone subunit	0,001225	-1,63	0,008106	-1,57	0,894744	-1,04	0,021419	-1,40	0,326132	1,12	0,1634	1,17
11746341_a_at	1213	CLTC	clathrin, heavy chain (Hc)	0,001201	-1,88	0,015115	-1,69	0,750014	-1,11	0,002206	-1,80	0,692761	-1,07	0,802186	1,04
11739030_s_at	1213	CLTC	clathrin, heavy chain (Hc)	0,004037	-1,93	0,011054	-1,95	0,985589	1,01	0,043118	-1,58	0,242202	1,23	0,246503	1,22
11739029_a_at	1213	CLTC	clathrin, heavy chain (Hc)	0,007344	-2,21	0,020835	-2,18	0,987272	-1,01	0,067142	-1,71	0,307218	1,27	0,266424	1,29
11742743_a_at	1266	CNN3	calponin 3, acidic	0,103827	-1,45	0,024275	-1,82	0,571897	1,26	0,068296	-1,53	0,347736	1,19	0,806321	-1,05

11743298_a_at	57472	CNOT6	CCR4-NOT transcription complex, subunit 6	0,017962	-1,62	0,02918	-1,67	0,943578	1,03	0,937912	1,02	0,001116	1,70	0,000853	1,65
11758306_s_at	57472	CNOT6	CCR4-NOT transcription complex, subunit 6	0,001045	-1,60	0,0057	-1,57	0,935369	-1,02	0,015543	-1,40	0,305524	1,12	0,190579	1,15
11723185_s_at	246175	CNOT6L	CCR4-NOT transcription complex, subunit 6-like	0,000282	-1,79	0,003841	-1,67	0,791394	-1,07	0,000456	-1,73	0,783039	-1,04	0,791366	1,04
11746299_a_at	54875	CNTLN	centlein, centrosomal protein	0,001116	-2,40	0,043493	-1,81	0,533242	-1,33	3,52E-05	-3,23	0,004344	-1,78	0,126274	-1,35
11723378_a_at	11064	CNTRL	centriolin	0,005513	-1,74	0,015496	-1,74	0,99851	-1,00	0,000122	-2,27	0,085309	-1,31	0,070245	-1,31
11724049_a_at	57511	COG6	component of oligomeric golgi complex 6	0,00235	-1,73	0,016441	-1,62	0,850398	-1,06	0,103344	-1,33	0,149668	1,22	0,042945	1,30
11721537_a_at	10087	COL4A3BP	collagen, type IV, alpha 3 (Goodpasture antigen) binding protein	0,017414	-1,48	0,009069	-1,66	0,696022	1,12	0,203039	1,24	3,60E-07	2,06	1,86E-06	1,84
11758133_s_at	10087	COL4A3BP	collagen, type IV, alpha 3 (Goodpasture antigen) binding protein	0,002243	-1,76	0,007732	-1,76	0,992412	-1,00	0,698534	-1,08	0,000711	1,63	0,000218	1,63
11749683_a_at	10087	COL4A3BP	collagen, type IV, alpha 3 (Goodpasture antigen) binding protein	0,00117	-1,69	0,00426	-1,70	0,995	1,00	0,180726	-1,23	0,009017	1,37	0,005251	1,37
11724191_a_at	9318	COPS2	COP9 signalosome subunit 2	0,009209	-1,57	0,029098	-1,54	0,962483	-1,02	0,873648	-1,03	0,003316	1,49	0,000906	1,52
11727131_at	1362	CPD	carboxypeptidase D	0,004335	-1,51	0,006068	-1,59	0,859475	1,05	0,038993	-1,35	0,140361	1,18	0,289331	1,13
11739734_a_at	80315	CPEB4	cytoplasmic polyadenylation element binding protein 4	0,239774	-1,25	0,036464	-1,56	0,49192	1,25	0,297225	1,22	2,31E-05	1,91	0,002061	1,52
11745256_s_at	80315	CPEB4	cytoplasmic polyadenylation element binding protein 4	0,090746	-1,37	0,040662	-1,56	0,700166	1,14	0,58732	1,12	0,000262	1,74	0,002046	1,53
11730828_a_at	53981	CPSF2	cleavage and polyadenylation specific factor 2, 100kDa	0,003061	-1,92	0,018678	-1,79	0,871297	-1,07	0,697774	1,10	0,000102	1,97	5,09E-06	2,10
11732306_x_at	1385	CREB1	cAMP responsive element binding protein 1	2,55E-05	-3,09	0,000456	-2,79	0,806979	-1,11	8,47E-07	-3,82	0,083917	-1,37	0,234965	-1,24
11736195_a_at	1385	CREB1	cAMP responsive element binding protein 1	7,60E-05	-2,69	0,001134	-2,45	0,820931	-1,10	2,38E-06	-3,37	0,070406	-1,38	0,188021	-1,25
11732303_a_at	1385	CREB1	cAMP responsive element binding protein 1	4,95E-05	-2,29	0,001288	-2,05	0,726813	-1,12	4,28E-06	-2,59	0,098857	-1,27	0,38905	-1,13
11732302_a_at	1385	CREB1	cAMP responsive element binding protein 1	0,002693	-2,01	0,016775	-1,87	0,873472	-1,07	8,51E-05	-2,63	0,054282	-1,41	0,111227	-1,31
11732304_a_at	1385	CREB1	cAMP responsive element binding protein 1	0,002073	-1,48	0,004974	-1,50	0,944672	1,02	0,001551	-1,49	0,95798	1,01	0,914827	-1,01
11730750_a_at	51340	CRNKL1	crooked neck pre-mRNA splicing factor 1	0,01481	-1,67	0,032856	-1,67	0,992745	1,00	0,266252	-1,27	0,095561	1,32	0,08431	1,31
11748688_x_at	1452	CSNK1A1	casein kinase 1, alpha 1	0,002445	-1,56	0,012516	-1,52	0,919768	-1,03	0,451144	-1,12	0,00721	1,36	0,001474	1,40
11763319_at	1452	CSNK1A1	casein kinase 1, alpha 1	0,001083	-2,14	0,009341	-1,96	0,834875	-1,09	0,000181	-2,44	0,217481	-1,24	0,459321	-1,14

11763317_x_at	1452	CSNK1A1	casein kinase 1, alpha 1	0,001222	-2,13	0,006946	-2,03	0,923896	-1,05	0,000257	-2,39	0,374434	-1,18	0,521905	-1,12
11763316_at	1452	CSNK1A1	casein kinase 1, alpha 1	0,00335	-1,95	0,044781	-1,67	0,692951	-1,17	0,000315	-2,35	0,051715	-1,41	0,284909	-1,20
11736310_x_at	1452	CSNK1A1	casein kinase 1, alpha 1	0,004532	-1,63	0,020114	-1,58	0,923554	-1,03	0,234458	-1,23	0,061755	1,28	0,023234	1,33
11736309_a_at	1452	CSNK1A1	casein kinase 1, alpha 1	0,006321	-1,84	0,038244	-1,69	0,840945	-1,09	0,148721	-1,38	0,250728	1,23	0,082539	1,33
11726031_a_at	1456	CSNK1G3	casein kinase 1, gamma 3	0,001153	-1,65	0,017126	-1,50	0,715375	-1,10	0,87261	-1,03	0,001229	1,46	1,80E-05	1,61
11740087_s_at	1457	CSNK2A1	casein kinase 2, alpha 1 polypeptide	0,002567	-1,59	0,013347	-1,54	0,912674	-1,03	0,655996	-1,08	0,002553	1,43	0,000373	1,48
11734373_a_at	79848	CSPP1	centrosome and spindle pole associated protein 1	0,005713	-1,83	0,036288	-1,68	0,834301	-1,09	2,22E-05	-2,78	0,003388	-1,65	0,008487	-1,52
11720494_a_at	8727	CTNNAL1	catenin (cadherin-associated protein), alpha-like 1	0,013584	-1,65	0,017755	-1,75	0,890024	1,06	0,008752	-1,72	0,931382	1,02	0,827608	-1,04
11756160_x_at	8727	CTNNAL1	catenin (cadherin-associated protein), alpha-like 1	0,017657	-1,56	0,019125	-1,67	0,85688	1,07	0,013592	-1,61	0,814943	1,04	0,884215	-1,03
11748951_a_at	1499	CTNNB1	catenin (cadherin-associated protein), beta 1, 88kDa	0,028811	-1,45	0,024997	-1,55	0,823053	1,07	0,908541	1,02	0,000688	1,59	0,001676	1,48
11717769_at	9646	CTR9	CTR9, Paf1/RNA polymerase II complex component	0,001693	-1,84	0,006049	-1,83	0,99443	-1,00	0,004104	-1,73	0,731198	1,06	0,713377	1,06
11747353_a_at	8454	CUL1	cullin 1	0,002271	-1,70	0,005213	-1,75	0,939496	1,03	0,000269	-1,93	0,488019	-1,10	0,348123	-1,13
11748246_a_at	8453	CUL2	cullin 2	0,00052	-1,69	0,001662	-1,72	0,948456	1,02	0,107032	-1,26	0,004792	1,37	0,004271	1,34
11716507_at	8452	CUL3	cullin 3	0,001354	-1,81	0,007636	-1,74	0,923299	-1,04	6,33E-05	-2,17	0,117243	-1,25	0,175181	-1,20
11716505_a_at	8452	CUL3	cullin 3	0,000487	-1,71	0,002081	-1,72	0,995227	1,00	0,003142	-1,55	0,375446	1,11	0,369649	1,11
11748671_a_at	8451	CUL4A	cullin 4A	0,007793	-1,57	0,032119	-1,52	0,916658	-1,04	0,141837	-1,28	0,220502	1,18	0,112634	1,22
11720095_a_at	8450	CUL4B	cullin 4B	0,002466	-1,51	0,007963	-1,51	0,998625	1,00	0,021383	-1,36	0,322332	1,11	0,308066	1,11
11722479_a_at	8065	CUL5	cullin 5	0,000282	-1,94	0,004657	-1,76	0,745485	-1,10	0,000247	-1,93	0,515422	-1,10	0,9746	1,01
11722477_at	8065	CUL5	cullin 5	0,005843	-1,59	0,03142	-1,51	0,871283	-1,05	0,000176	-1,96	0,046381	-1,30	0,096142	-1,23
11720955_x_at	57703	CWC22	CWC22 spliceosome-associated protein homolog (S. cerevisiae)	3,23E-05	-2,41	0,000523	-2,22	0,818275	-1,08	1,31E-06	-2,83	0,098758	-1,27	0,256198	-1,18
11754743_a_at	57703	CWC22	CWC22 spliceosome-associated protein homolog (S. cerevisiae)	0,00016	-2,31	0,003022	-2,06	0,746972	-1,12	5,30E-06	-2,84	0,041732	-1,38	0,178248	-1,23
11752849_a_at	10283	CWC27	CWC27 spliceosome-associated protein homolog (S. cerevisiae)	7,47E-05	-1,86	0,001538	-1,72	0,761357	-1,08	1,44E-06	-2,19	0,029245	-1,27	0,121898	-1,18
11752850_x_at	10283	CWC27	CWC27 spliceosome-associated protein homolog (S. cerevisiae)	6,50E-05	-1,64	0,001521	-1,53	0,739718	-1,07	2,20E-06	-1,82	0,047303	-1,19	0,204908	-1,11
11740080_at	80231	CXorf21	chromosome X open reading frame 21	0,005428	-2,15	0,007422	-2,36	0,861852	1,10	0,00537	-2,17	0,72286	1,09	0,979705	-1,01
11755080_a_at	23002	DAAM1	dishevelled associated activator of morphogenesis 1	0,014684	-1,49	0,017981	-1,57	0,879478	1,05	0,078909	-1,34	0,223968	1,17	0,399527	1,12

11727088_a_at	1602	DACH1	dachshund family transcription factor 1	0,026007	-1,53	0,023335	-1,65	0,829251	1,08	2,59E-05	-2,45	0,008824	-1,49	0,000773	-1,61
11734713_s_at	10926	DBF4	DBF4 zinc finger	0,000139	-2,38	0,005615	-1,96	0,589978	-1,21	0,000189	-2,28	0,369238	-1,16	0,822974	1,04
11753043_s_at	10926	DBF4	DBF4 zinc finger	0,000113	-2,11	0,003346	-1,85	0,666436	-1,14	0,000474	-1,91	0,850832	-1,03	0,469517	1,11
11747731_a_at	10926	DBF4	DBF4 zinc finger	0,000896	-2,05	0,009732	-1,85	0,788648	-1,10	0,019732	-1,62	0,414214	1,15	0,125659	1,27
11734715_a_at	10926	DBF4	DBF4 zinc finger	0,002415	-1,73	0,020594	-1,59	0,801581	-1,08	0,061826	-1,39	0,32697	1,15	0,096193	1,24
11752175_x_at	10926	DBF4	DBF4 zinc finger	0,002101	-1,68	0,017294	-1,57	0,818182	-1,07	0,023715	-1,45	0,570431	1,08	0,244082	1,16
11755472_x_at	51163	DBR1	debranching RNA lariats 1	0,000901	-1,96	0,015371	-1,71	0,692951	-1,14	0,21359	-1,27	0,049312	1,34	0,002195	1,54
11743648_a_at	55827	DCAF6	DDB1 and CUL4 associated factor 6	0,000437	-1,69	0,003326	-1,63	0,897695	-1,04	0,177837	-1,21	0,005993	1,35	0,000924	1,40
11729625_a_at	10238	DCAF7	DDB1 and CUL4 associated factor 7	0,0144	-1,55	0,043254	-1,52	0,956171	-1,02	0,231039	-1,25	0,166022	1,22	0,104299	1,25
11749894_a_at	1633	DCK	deoxycytidine kinase	0,016262	-1,87	0,025762	-1,96	0,936763	1,05	0,766089	1,09	0,00031	2,14	0,00022	2,04
11720963_a_at	1633	DCK	deoxycytidine kinase	0,025288	-1,84	0,042221	-1,89	0,959269	1,03	0,877998	1,05	0,001603	1,99	0,001074	1,93
11719739_s_at	167227	DCP2	decapping mRNA 2	0,003053	-1,57	0,007598	-1,60	0,962649	1,02	0,007177	-1,51	0,634195	1,06	0,729764	1,05
11719738_at	167227	DCP2	decapping mRNA 2	0,002873	-1,68	0,018979	-1,58	0,854454	-1,06	0,032991	-1,44	0,49551	1,10	0,235086	1,17
11726327_at	54165	DCUN1D1	DCN1, defective in cullin neddylation 1, domain containing 1	0,000161	-2,83	0,00143	-2,66	0,908091	-1,06	0,942063	1,02	1,87E-06	2,72	9,55E-08	2,89
11757927_s_at	23142	DCUN1D4	DCN1, defective in cullin neddylation 1, domain containing 4	0,00093	-2,01	0,029259	-1,65	0,571801	-1,22	0,004659	-1,79	0,649404	-1,08	0,459608	1,13
11743035_a_at	1653	DDX1	DEAD (Asp-Glu-Ala-Asp) box helicase 1	0,001076	-1,67	0,0178	-1,51	0,694541	-1,11	0,061417	-1,32	0,283885	1,14	0,03532	1,26
11715474_at	10521	DDX17	DEAD (Asp-Glu-Ala-Asp) box helicase 17	0,00237	-2,38	0,030516	-1,99	0,714279	-1,20	0,529548	-1,20	0,020817	1,66	0,000773	1,98
11750750_a_at	1654	DDX3X	DEAD (Asp-Glu-Ala-Asp) box helicase 3, X-linked	0,008402	-2,18	0,029372	-2,09	0,946073	-1,04	0,226747	1,44	6,72E-06	3,00	5,98E-07	3,13
11750751_x_at	1654	DDX3X	DEAD (Asp-Glu-Ala-Asp) box helicase 3, X-linked	0,001758	-2,54	0,007386	-2,48	0,968138	-1,03	0,757462	1,10	2,08E-05	2,73	2,66E-06	2,81
11727832_s_at	11325	DDX42	DEAD (Asp-Glu-Ala-Asp) box helicase 42	0,00054	-1,76	0,018289	-1,52	0,576128	-1,16	5,61E-08	-2,76	3,33E-06	-1,81	8,55E-05	-1,56
11727818_a_at	9879	DDX46	DEAD (Asp-Glu-Ala-Asp) box polypeptide 46	0,000708	-1,68	0,003669	-1,65	0,964087	-1,01	0,009093	-1,46	0,29579	1,13	0,218829	1,15
11739627_a_at	11056	DDX52	DEAD (Asp-Glu-Ala-Asp) box polypeptide 52	0,004816	-1,61	0,028821	-1,52	0,856644	-1,06	0,006951	-1,58	0,793117	-1,04	0,905606	1,02
11749568_x_at	57696	DDX55	DEAD (Asp-Glu-Ala-Asp) box polypeptide 55	0,001492	-1,59	0,007177	-1,56	0,948783	-1,02	0,002446	-1,55	0,963918	1,01	0,84358	1,03
11749567_a_at	57696	DDX55	DEAD (Asp-Glu-Ala-Asp) box polypeptide 55	0,002365	-1,64	0,021135	-1,52	0,792213	-1,08	0,004353	-1,58	0,766654	-1,04	0,810018	1,03
11743652_a_at	7913	DEK	DEK proto-oncogene	8,23E-05	-2,17	0,00283	-1,90	0,660805	-1,15	0,000681	-1,88	0,95527	1,01	0,292964	1,16

11743650_s_at	7913	DEK	DEK proto-oncogene	0,000452	-1,87	0,002796	-1,83	0,948629	-1,02	0,059313	-1,37	0,026631	1,34	0,01033	1,37
11743653_x_at	7913	DEK	DEK proto-oncogene	0,00018	-1,93	0,003971	-1,74	0,711086	-1,11	0,001591	-1,69	0,844058	1,03	0,285503	1,14
11751258_x_at	7913	DEK	DEK proto-oncogene	0,003152	-2,57	0,015318	-2,40	0,919161	-1,07	0,475252	-1,26	0,008935	1,90	0,001924	2,03
11743651_a_at	7913	DEK	DEK proto-oncogene	0,001322	-2,67	0,006038	-2,59	0,962345	-1,03	0,153744	-1,53	0,021845	1,70	0,009189	1,75
11741575_a_at	7913	DEK	DEK proto-oncogene	0,001469	-3,06	0,008344	-2,85	0,917492	-1,07	0,170191	-1,60	0,029489	1,78	0,008659	1,91
11757280_s_at	7913	DEK	DEK proto-oncogene	0,000639	-1,77	0,004179	-1,72	0,920442	-1,03	0,015154	-1,47	0,225171	1,17	0,118285	1,20
11746333_a_at	55667	DENND4C	DENN/MADD domain containing 4C	0,000378	-2,38	0,003134	-2,24	0,891876	-1,06	0,004814	-1,92	0,397388	1,17	0,209494	1,24
11726426_a_at	55667	DENND4C	DENN/MADD domain containing 4C	0,023041	-1,53	0,049725	-1,53	0,999343	-1,00	0,003308	-1,76	0,347893	-1,15	0,331947	-1,15
11753261_x_at	8562	DENR	density-regulated protein	0,000473	-1,54	0,002452	-1,53	0,976769	-1,01	0,012075	-1,34	0,146835	1,14	0,105948	1,15
11733884_a_at	55635	DEPDC1	DEP domain containing 1	0,000441	-2,91	0,004727	-2,58	0,81786	-1,13	0,051293	-1,73	0,073132	1,49	0,012459	1,68
11730111_a_at	55635	DEPDC1	DEP domain containing 1	0,005705	-2,24	0,04138	-1,96	0,801015	-1,14	0,108502	-1,59	0,373096	1,23	0,117029	1,41
11743114_at	1665	DHX15	DEAH (Asp-Glu-Ala-His) box helicase 15	0,003587	-1,60	0,009235	-1,62	0,974981	1,01	0,100833	-1,30	0,07588	1,25	0,076387	1,23
11717857_a_at	54505	DHX29	DEAH (Asp-Glu-Ala-His) box polypeptide 29	0,003584	-1,67	0,008234	-1,71	0,952553	1,02	0,019602	-1,50	0,356533	1,14	0,439208	1,11
11719502_a_at	170506	DHX36	DEAH (Asp-Glu-Ala-His) box polypeptide 36	1,03E-05	-2,55	0,000426	-2,22	0,66374	-1,15	0,00168	-1,80	0,137994	1,24	0,008282	1,42
11719505_x_at	170506	DHX36	DEAH (Asp-Glu-Ala-His) box polypeptide 36	0,000222	-1,89	0,010262	-1,61	0,551789	-1,18	1,25E-05	-2,18	0,014914	-1,35	0,254105	-1,15
11750762_a_at	79665	DHX40	DEAH (Asp-Glu-Ala-His) box polypeptide 40	0,00096	-1,96	0,005405	-1,90	0,929775	-1,04	0,358136	-1,20	0,003094	1,58	0,000565	1,63
11716648_a_at	79665	DHX40	DEAH (Asp-Glu-Ala-His) box polypeptide 40	0,001749	-1,60	0,008688	-1,56	0,937077	-1,02	0,194675	-1,21	0,024868	1,29	0,008379	1,32
11743106_a_at	1660	DHX9	DEAH (Asp-Glu-Ala-His) box helicase 9	0,001292	-2,42	0,00908	-2,23	0,878749	-1,08	0,167893	-1,44	0,034667	1,55	0,00734	1,68
11722339_a_at	1730	DIAPH2	diaphanous-related formin 2	0,000692	-1,76	0,009018	-1,62	0,761801	-1,09	0,00012	-1,93	0,162756	-1,19	0,486017	-1,09
11757970_s_at	23405	DICER1	dicer 1, ribonuclease type III	0,000752	-1,84	0,004943	-1,77	0,910426	-1,04	0,186019	-1,26	0,011074	1,41	0,0023	1,46
11740190_s_at	23405	DICER1	dicer 1, ribonuclease type III	0,009656	-1,60	0,02905	-1,57	0,969414	-1,02	0,811137	-1,05	0,004624	1,50	0,001489	1,52
11748232_a_at	22894	DIS3	DIS3 exosome endoribonuclease and 3'-5' exoribonuclease	0,001071	-1,91	0,012243	-1,73	0,774248	-1,10	0,023467	-1,54	0,436812	1,13	0,126324	1,24
11726623_a_at	22894	DIS3	DIS3 exosome endoribonuclease and 3'-5' exoribonuclease	0,001002	-1,97	0,025921	-1,65	0,602457	-1,19	0,010896	-1,66	0,98322	-1,00	0,254384	1,19
11743467_at	1736 /// 677835 /// 100847052	DKC1 /// MIR664B /// SNORA56	dyskeratosis congenita 1, dyskerin /// microRNA 664b /// small nucleolar RNA, H/ACA box	0,000179	-2,65	0,003364	-2,30	0,738327	-1,15	0,002418	-2,10	0,645317	1,10	0,199608	1,26

11751509_a_at	5954 /// 440034	DKFZp686K 1684 /// RCN1	uncharacterized LOC440034 /// reticulocalbin 1, EF-hand calcium binding domain	0,002439	-1,74	0,028544	-1,57	0,734054	-1,11	0,014493	-1,55	0,960635	1,01	0,41639	1,12
11718108_a_at	1738	DLD	dihydrolipoamide dehydrogenase	0,001492	-1,57	0,008886	-1,52	0,907121	-1,03	0,205555	-1,19	0,023442	1,28	0,005789	1,32
11749540_x_at	1738	DLD	dihydrolipoamide dehydrogenase	0,001421	-1,57	0,00841	-1,52	0,91027	-1,03	0,135287	-1,23	0,043444	1,24	0,01315	1,28
11748488_a_at	1738	DLD	dihydrolipoamide dehydrogenase	0,004341	-1,68	0,012001	-1,69	0,990036	1,01	0,377646	-1,18	0,010516	1,43	0,006751	1,43
11718109_s_at	1738	DLD	dihydrolipoamide dehydrogenase	0,004058	-1,69	0,028849	-1,57	0,823069	-1,08	0,395315	-1,17	0,038437	1,34	0,00532	1,44
11739308_s_at	1739	DLG1	discs, large homolog 1 (Drosophila)	0,00364	-1,82	0,009494	-1,84	0,976939	1,01	0,069097	-1,44	0,125749	1,28	0,130683	1,26
11755657_s_at	1657	DMXL1	Dmx-like 1	0,004005	-1,84	0,015955	-1,79	0,94974	-1,03	0,000406	-2,19	0,231246	-1,22	0,291575	-1,19
11717189_x_at	3301	DNAJA1	DnaJ (Hsp40) homolog, subfamily A, member 1	0,002479	-1,54	0,006049	-1,57	0,95324	1,02	0,778956	-1,04	0,00027	1,50	0,000151	1,47
11761703_s_at	3301	DNAJA1	DnaJ (Hsp40) homolog, subfamily A, member 1	0,00271	-1,50	0,008375	-1,51	0,994681	1,00	0,617597	-1,07	0,001334	1,40	0,000541	1,40
11760005_x_at	3301	DNAJA1	DnaJ (Hsp40) homolog, subfamily A, member 1	0,000888	-1,70	0,005698	-1,64	0,907701	-1,04	0,063467	-1,32	0,073085	1,24	0,025247	1,28
11758820_at	54431	DNAJC10	DnaJ (Hsp40) homolog, subfamily C, member 10	0,001931	-1,60	0,009371	-1,56	0,936307	-1,03	0,071501	-1,30	0,119336	1,20	0,059494	1,23
11716844_s_at	23317	DNAJC13	DnaJ (Hsp40) homolog, subfamily C, member 13	0,011307	-1,59	0,03147	-1,57	0,977795	-1,01	0,012729	-1,58	0,958039	-1,01	0,987295	1,00
11735131_a_at	134218	DNAJC21	DnaJ (Hsp40) homolog, subfamily C, member 21	0,000397	-1,61	0,005069	-1,51	0,779503	-1,06	5,25E-05	-1,73	0,160812	-1,15	0,44977	-1,08
11726834_a_at	134218	DNAJC21	DnaJ (Hsp40) homolog, subfamily C, member 21	0,000175	-1,66	0,001495	-1,61	0,913168	-1,03	0,001767	-1,49	0,422209	1,08	0,254578	1,11
11743371_x_at	10059	DNM1L	dynamin 1-like	1,16E-05	-3,14	0,00039	-2,70	0,69669	-1,16	2,62E-05	-2,82	0,825494	-1,04	0,554486	1,11
11737604_a_at	10059	DNM1L	dynamin 1-like	0,000168	-1,83	0,001944	-1,74	0,856699	-1,05	0,018918	-1,41	0,069931	1,23	0,015832	1,30
11750026_a_at	10059	DNM1L	dynamin 1-like	0,003748	-2,07	0,018131	-1,96	0,912312	-1,06	0,375711	-1,25	0,021098	1,56	0,005357	1,65
11736405_a_at	1786	DNMT1	DNA (cytosine-5-)-methyltransferase 1	0,001948	-1,87	0,016502	-1,72	0,814146	-1,09	0,002599	-1,83	0,707892	-1,07	0,913354	1,02
11723372_a_at	139818	DOCK11	dedicator of cytokinesis 11	0,00045	-1,63	0,001427	-1,66	0,947934	1,02	0,003569	-1,47	0,247004	1,13	0,315391	1,11
11753987_x_at	85440	DOCK7	dedicator of cytokinesis 7	0,00012	-1,62	0,000204	-1,72	0,760706	1,06	5,90E-06	-1,79	0,691011	-1,04	0,250699	-1,11
11739884_a_at	85440	DOCK7	dedicator of cytokinesis 7	0,001084	-1,66	0,004735	-1,64	0,977612	-1,01	6,85E-05	-1,90	0,217179	-1,16	0,232463	-1,15
11739885_s_at	85440	DOCK7	dedicator of cytokinesis 7	0,002963	-1,78	0,020276	-1,66	0,844947	-1,07	0,000185	-2,15	0,088092	-1,29	0,204008	-1,20
11733040_a_at	285381	DPH3	diphthamide biosynthesis 3	0,001999	-1,66	0,003841	-1,74	0,891787	1,04	0,016955	-1,47	0,183094	1,18	0,314576	1,13
11716723_at	23333	DPY19L1	dpy-19-like 1 (C. elegans)	0,000704	-1,72	0,00603	-1,64	0,854454	-1,05	0,018406	-1,43	0,280508	1,14	0,105287	1,20
11716724_x_at	23333	DPY19L1	dpy-19-like 1 (C. elegans)	0,002963	-1,59	0,007646	-1,61	0,968278	1,01	0,172963	-1,23	0,025276	1,31	0,023625	1,29
11722178_at	1810	DR1	down-regulator of transcription 1, TBP-binding (negative cofactor 2)	0,001877	-1,42	0,001562	-1,52	0,711969	1,07	0,555796	1,07	1,73E-07	1,63	6,97E-07	1,52

11725523_at	1829	DSG2	desmoglein 2	0,0004	-2,20	0,010541	-1,85	0,631548	-1,19	3,26E-05	-2,57	0,040954	-1,39	0,327676	-1,17
11725524_s_at	1829	DSG2	desmoglein 2	0,012485	-1,64	0,038369	-1,60	0,957043	-1,02	8,86E-05	-2,33	0,016199	-1,45	0,015946	-1,42
11737993_s_at	667	DST	dystonin	0,004649	-1,64	0,033166	-1,53	0,816202	-1,08	5,82E-06	-2,44	0,000679	-1,60	0,001766	-1,48
11752100_s_at	1871	E2F3	E2F transcription factor 3	0,001544	-1,55	0,006785	-1,53	0,962743	-1,01	0,889991	1,02	4,11E-05	1,56	5,27E-06	1,58
11730877_a_at	9166	EBAG9	estrogen receptor binding site associated, antigen, 9	1,28E-05	-2,01	0,000919	-1,75	0,563721	-1,15	1,94E-06	-2,12	0,075147	-1,21	0,637605	-1,06
11717434_s_at	55862	ECHDC1	enoyl CoA hydratase domain containing 1	0,001551	-1,56	0,00891	-1,51	0,911666	-1,03	0,057706	-1,29	0,142809	1,17	0,062441	1,21
11717433_a_at	55862	ECHDC1	enoyl CoA hydratase domain containing 1	0,001262	-1,72	0,007825	-1,65	0,906645	-1,04	0,012777	-1,50	0,462941	1,10	0,276157	1,15
11739466_a_at	55862	ECHDC1	enoyl CoA hydratase domain containing 1	0,00331	-2,16	0,010696	-2,14	0,992249	-1,01	0,042103	-1,68	0,239546	1,27	0,208701	1,28
11741546_a_at	55862	ECHDC1	enoyl CoA hydratase domain containing 1	0,002666	-2,10	0,0138	-1,99	0,913789	-1,05	0,035639	-1,66	0,353253	1,20	0,202921	1,26
11717432_a_at	55862	ECHDC1	enoyl CoA hydratase domain containing 1	0,002731	-1,72	0,00982	-1,70	0,979111	-1,01	0,004981	-1,65	0,872427	1,03	0,817304	1,04
11743698_at	80267	EDEM3	ER degradation enhancer, mannosidase alpha-like 3	9,78E-05	-2,34	0,002393	-2,07	0,722746	-1,13	0,005069	-1,75	0,295433	1,18	0,047611	1,33
11757995_s_at	80267	EDEM3	ER degradation enhancer, mannosidase alpha-like 3	0,000147	-2,13	0,002796	-1,92	0,755471	-1,10	0,001524	-1,81	0,690459	1,06	0,244814	1,18
11745634_a_at	26098	EDRF1	erythroid differentiation regulatory factor 1	0,000969	-1,64	0,008402	-1,55	0,837574	-1,06	0,008258	-1,46	0,63008	1,06	0,309325	1,12
11718823_a_at	26098	EDRF1	erythroid differentiation regulatory factor 1	0,005287	-1,65	0,044201	-1,51	0,774248	-1,10	0,005096	-1,67	0,508694	-1,10	0,971723	-1,01
11719646_a_at	23301	EHBP1	EH domain binding protein 1	0,001153	-1,66	0,005821	-1,63	0,947823	-1,02	6,97E-05	-1,92	0,173102	-1,18	0,222138	-1,15
11740039_a_at	23301	EHBP1	EH domain binding protein 1	0,001201	-2,12	0,046396	-1,66	0,533245	-1,28	0,021518	-1,67	0,977799	-1,01	0,159115	1,27
11744914_s_at	23741	EID1	EP300 interacting inhibitor of differentiation 1	2,50E-05	-1,70	0,000913	-1,57	0,666428	-1,09	1,92E-06	-1,83	0,075174	-1,17	0,419366	-1,07
11716778_s_at	1964	EIF1AX	eukaryotic translation initiation factor 1A, X-linked	5,98E-05	-1,97	0,000238	-2,05	0,90075	1,04	0,032301	-1,38	0,001079	1,48	0,001297	1,43
11716779_at	1964	EIF1AX	eukaryotic translation initiation factor 1A, X-linked	0,00057	-1,97	0,00323	-1,93	0,956171	-1,02	0,00064	-1,94	0,964192	-1,01	0,934494	1,01
11730991_a_at	5610	EIF2AK2	eukaryotic translation initiation factor 2-alpha kinase 2	0,000688	-1,72	0,009223	-1,58	0,754362	-1,09	0,282186	-1,18	0,013413	1,34	0,000648	1,46
11722152_s_at	9451	EIF2AK3	eukaryotic translation initiation factor 2-alpha kinase 3	0,021925	-1,77	0,046766	-1,77	0,997485	1,00	4,64E-05	-3,07	0,005342	-1,73	0,002533	-1,74
11722151_a_at	9451	EIF2AK3	eukaryotic translation initiation factor 2-alpha kinase 3	0,018513	-1,85	0,034312	-1,89	0,96922	1,02	6,45E-05	-3,15	0,013651	-1,66	0,005559	-1,70
11722150_a_at	9451	EIF2AK3	eukaryotic translation initiation factor 2-alpha kinase 3	0,026655	-1,59	0,024667	-1,73	0,83694	1,09	9,21E-05	-2,46	0,03343	-1,42	0,004931	-1,54
11752101_s_at	1965	EIF2S1	eukaryotic translation initiation factor 2, subunit 1 alpha, 35kDa	0,00023	-2,15	0,00245	-2,02	0,865219	-1,06	0,000199	-2,14	0,730646	-1,06	0,97936	1,00

11716221_at	1965	EIF2S1	eukaryotic translation initiation factor 2, subunit 1 alpha, 35kDa	0,00183	-1,72	0,034064	-1,50	0,64768	-1,14	0,000707	-1,82	0,155331	-1,21	0,702225	-1,06
11749910_a_at	1965	EIF2S1	eukaryotic translation initiation factor 2, subunit 1 alpha, 35kDa	0,001878	-1,85	0,018941	-1,68	0,776499	-1,10	0,118468	-1,35	0,150885	1,24	0,02513	1,37
11723917_s_at	8894	EIF2S2	eukaryotic translation initiation factor 2, subunit 2 beta, 38kDa	3,96E-05	-1,60	0,000234	-1,62	0,959969	1,01	4,97E-05	-1,57	0,719957	1,03	0,823728	1,02
11719067_s_at	1968	EIF2S3	eukaryotic translation initiation factor 2, subunit 3 gamma, 52kDa	0,011801	-1,82	0,040277	-1,75	0,939418	-1,04	0,392933	-1,24	0,062067	1,42	0,026915	1,47
11721471_a_at	8661	EIF3A	eukaryotic translation initiation factor 3, subunit A	7,14E-05	-1,89	0,001111	-1,78	0,818705	-1,06	1,40E-05	-2,02	0,286526	-1,13	0,600023	-1,07
11752758_x_at	1975	EIF4B	eukaryotic translation initiation factor 4B	0,001577	-1,87	0,021749	-1,65	0,71607	-1,13	0,054546	-1,44	0,381812	1,15	0,069259	1,29
11730371_a_at	317649	EIF4E3	eukaryotic translation initiation factor 4E family member 3	0,063734	-1,47	0,048496	-1,61	0,812012	1,10	0,000544	-2,16	0,078533	-1,34	0,013041	-1,47
11751928_x_at	8672	EIF4G3	eukaryotic translation initiation factor 4 gamma, 3	0,000758	-1,46	0,001099	-1,53	0,802713	1,05	2,40E-05	-1,65	0,409886	-1,07	0,136173	-1,13
11746537_x_at	8672	EIF4G3	eukaryotic translation initiation factor 4 gamma, 3	0,003575	-1,43	0,003339	-1,53	0,755845	1,07	0,000156	-1,64	0,512037	-1,07	0,147381	-1,14
11721532_x_at	8672	EIF4G3	eukaryotic translation initiation factor 4 gamma, 3	0,005072	-1,58	0,014019	-1,59	0,993976	1,00	0,000213	-1,90	0,161901	-1,20	0,136581	-1,20
11748711_a_at	8672	EIF4G3	eukaryotic translation initiation factor 4 gamma, 3	0,003587	-1,52	0,003961	-1,63	0,790424	1,07	0,003402	-1,53	0,59802	1,07	0,972254	-1,00
11750029_a_at	7458	EIF4H	eukaryotic translation initiation factor 4H	0,002253	-2,33	0,041328	-1,87	0,643432	-1,24	0,018252	-1,89	0,967274	-1,01	0,326138	1,23
11743364_a_at	9669	EIF5B	eukaryotic translation initiation factor 5B	4,83E-05	-2,02	0,002945	-1,74	0,572571	-1,16	2,78E-07	-2,54	0,001733	-1,46	0,041399	-1,26
11740445_a_at	1997	ELF1	E74-like factor 1 (ets domain transcription factor)	0,00384	-2,06	0,025273	-1,88	0,8435	-1,10	0,589825	1,15	9,83E-05	2,17	3,64E-06	2,38
11719802_a_at	1997	ELF1	E74-like factor 1 (ets domain transcription factor)	0,00104	-2,08	0,01001	-1,90	0,811186	-1,10	0,000489	-2,19	0,409251	-1,15	0,787671	-1,05
11744931_x_at	1998	ELF2	E74-like factor 2 (ets domain transcription factor)	0,000162	-2,20	0,003112	-1,97	0,747925	-1,11	1,96E-06	-2,83	0,015363	-1,44	0,073887	-1,29
11764111_s_at	255520	ELMOD2	ELMO/CED-12 domain containing 2	0,00152	-2,05	0,019152	-1,80	0,736271	-1,14	0,000555	-2,21	0,241534	-1,23	0,687657	-1,08
11722886_s_at	133418 /// 647121	EMB /// EMBP1	embigin /// embigin pseudogene 1	0,020242	-1,69	0,014213	-1,92	0,761357	1,13	0,092755	-1,47	0,141988	1,30	0,440976	1,15
11716816_a_at	58478	ENOPH1	enolase-phosphatase 1	0,001839	-1,84	0,014943	-1,70	0,828818	-1,08	0,043053	-1,47	0,32629	1,16	0,111589	1,25
11727174_at	26122	EPC2	enhancer of polycomb homolog 2 (Drosophila)	0,000376	-1,93	0,003211	-1,84	0,886892	-1,05	0,000488	-1,88	0,892082	-1,02	0,861665	1,03
11715970_a_at	2058	EPRS	glutamyl-prolyl-tRNA synthetase	4,98E-06	-2,90	8,81E-05	-2,72	0,856767	-1,07	8,88E-05	-2,28	0,262153	1,19	0,096946	1,27
11715969_a_at	2058	EPRS	glutamyl-prolyl-tRNA synthetase	0,000282	-1,74	0,002406	-1,68	0,90542	-1,03	0,004492	-1,50	0,326226	1,12	0,172687	1,16

11751923_a_at	55914	ERBB2IP	erbb2 interacting protein	0,004121	-1,76	0,016286	-1,71	0,950569	-1,03	0,701853	-1,08	0,002894	1,58	0,000669	1,62
11734848_s_at	55914	ERBB2IP	erbb2 interacting protein	0,000788	-1,60	0,000977	-1,71	0,776418	1,07	0,020278	-1,36	0,027994	1,26	0,10656	1,17
11739357_a_at	2078	ERG	v-ets avian erythroblastosis virus E26 oncogene homolog	0,000687	-2,59	0,011893	-2,17	0,703036	-1,19	1,41E-07	-5,28	3,87E-05	-2,43	0,000263	-2,04
11750309_a_at	112479	ERI2	ERI1 exoribonuclease family member 2	0,004677	-2,39	0,041775	-2,03	0,765441	-1,18	0,812428	1,09	0,001086	2,21	2,71E-05	2,60
11725784_a_at	112479	ERI2	ERI1 exoribonuclease family member 2	0,000575	-2,12	0,006184	-1,94	0,81072	-1,09	0,061446	-1,47	0,085371	1,32	0,014568	1,44
11725099_at	51575	ESF1	ESF1, nucleolar pre-rRNA processing protein, homolog (S. cerevisiae)	5,98E-05	-2,06	0,002796	-1,79	0,611541	-1,15	2,99E-07	-2,63	0,002214	-1,47	0,036555	-1,28
11743705_at	54465	ETAA1	Ewing tumor-associated antigen 1	0,002372	-1,76	0,041722	-1,52	0,650055	-1,15	0,001064	-1,85	0,173843	-1,21	0,743121	-1,05
11749144_s_at	2110	ETFDH	electron-transferring-flavoprotein dehydrogenase	0,000219	-1,58	0,003013	-1,50	0,805169	-1,05	0,006951	-1,37	0,294741	1,10	0,084401	1,16
11743707_a_at	55763	EXOC1	exocyst complex component 1	0,002854	-1,88	0,010419	-1,86	0,976769	-1,01	0,001117	-2,02	0,634834	-1,09	0,693187	-1,07
11718497_a_at	55763	EXOC1	exocyst complex component 1	0,003967	-1,84	0,015318	-1,80	0,957043	-1,03	0,001512	-1,98	0,567304	-1,11	0,671502	-1,08
11719458_at	10640	EXOC5	exocyst complex component 5	0,00045	-1,77	0,003307	-1,71	0,905918	-1,04	0,071297	-1,31	0,027839	1,30	0,007138	1,35
11748690_a_at	2146	EZH2	enhancer of zeste 2 polycomb repressive complex 2 subunit	0,003405	-1,97	0,019781	-1,84	0,878346	-1,07	0,056898	-1,54	0,337565	1,19	0,154114	1,28
11741829_a_at	55179	FAIM	Fas apoptotic inhibitory molecule	0,001766	-1,71	0,024368	-1,53	0,712275	-1,11	2,46E-05	-2,17	0,007824	-1,41	0,051703	-1,27
11719313_a_at	63901	FAM111A	family with sequence similarity 111, member A	0,003246	-1,91	0,044805	-1,64	0,686786	-1,16	0,018697	-1,66	0,943161	-1,01	0,422905	1,15
11739207_x_at	9747 /// 100294033	FAM115A /// LOC100294033	family with sequence similarity 115, member A /// protein FAM115A-like	2,46E-05	-1,75	0,001118	-1,58	0,620127	-1,10	3,74E-07	-1,99	0,011285	-1,26	0,135137	-1,14
11725075_a_at	84668	FAM126A	family with sequence similarity 126, member A	0,004413	-1,75	0,021398	-1,67	0,905928	-1,05	0,00146	-1,90	0,425544	-1,14	0,623075	-1,08
11728050_s_at	285172	FAM126B	family with sequence similarity 126, member B	0,00045	-2,01	0,006255	-1,81	0,755741	-1,11	0,000673	-1,93	0,683929	-1,07	0,822278	1,04
11729279_x_at	84142	FAM175A	family with sequence similarity 175, member A	1,83E-05	-3,11	0,000335	-2,79	0,794163	-1,11	0,000197	-2,48	0,536827	1,12	0,196346	1,25
11729280_a_at	84142	FAM175A	family with sequence similarity 175, member A	0,001676	-1,71	0,014646	-1,59	0,817282	-1,07	0,006085	-1,58	0,965332	1,01	0,574956	1,08
11721180_at	23172	FAM175B	family with sequence similarity 175, member B	0,005629	-1,55	0,02393	-1,50	0,923264	-1,03	0,414543	-1,14	0,026071	1,31	0,007743	1,36
11755286_at	221786	FAM200A	family with sequence similarity 200, member A	0,001547	-1,72	0,020727	-1,55	0,722436	-1,11	0,126752	-1,29	0,155044	1,21	0,016976	1,34
11740430_x_at	23272	FAM208A	family with sequence similarity 208, member A	0,010141	-1,73	0,033638	-1,68	0,948629	-1,03	3,94E-05	-2,64	0,007796	-1,57	0,007466	-1,52

11739247_a_at	54906	FAM208B	family with sequence similarity 208, member B	0,003984	-1,83	0,041722	-1,62	0,736602	-1,13	0,000467	-2,15	0,082517	-1,33	0,32374	-1,17
11756668_x_at	84293	FAM213A	family with sequence similarity 213, member A	0,000417	-1,68	0,006712	-1,55	0,728262	-1,09	1,25E-05	-1,96	0,028163	-1,27	0,144243	-1,16
11742763_a_at	56204	FAM214A	family with sequence similarity 214, member A	0,017125	-1,56	0,029259	-1,60	0,950704	1,03	0,004947	-1,71	0,670117	-1,07	0,543505	-1,10
11743608_s_at	10447	FAM3C	family with sequence similarity 3, member C	0,000692	-1,90	0,006523	-1,77	0,831445	-1,07	1,78E-05	-2,36	0,04304	-1,33	0,113274	-1,24
11742232_s_at	10447	FAM3C	family with sequence similarity 3, member C	0,002383	-1,78	0,025007	-1,61	0,757411	-1,11	2,62E-05	-2,37	0,008015	-1,47	0,037514	-1,33
11755773_x_at	54629	FAM63B	family with sequence similarity 63, member B	2,68E-05	-2,20	0,000477	-2,04	0,803237	-1,08	0,000134	-1,97	0,792493	1,04	0,388031	1,12
11725847_s_at	143684	FAM76B	family with sequence similarity 76, member B	0,010479	-1,67	0,041274	-1,60	0,912978	-1,05	0,002619	-1,86	0,35193	-1,16	0,513029	-1,11
11719895_x_at	157769	FAM91A1	family with sequence similarity 91, member A1	0,010294	-1,85	0,038438	-1,77	0,926254	-1,05	0,93158	1,02	0,001825	1,81	0,000283	1,90
11719894_s_at	57234 /// 157769	FAM91A1 /// LINC00869	family with sequence similarity 91, member A1 /// long intergenic non-protein coding RN	0,004361	-2,10	0,049131	-1,78	0,71781	-1,18	0,597997	-1,16	0,031332	1,54	0,001474	1,82
11759139_at	283742	FAM98B	family with sequence similarity 98, member B	0,000923	-1,81	0,011331	-1,65	0,763084	-1,10	0,000151	-2,00	0,152649	-1,21	0,461726	-1,11
11755555_a_at	57697	FANCM	Fanconi anemia, complementation group M	0,005071	-1,69	0,043797	-1,53	0,768795	-1,10	0,024182	-1,52	0,960105	1,01	0,478644	1,11
11734737_a_at	84188	FAR1	fatty acyl CoA reductase 1	0,003106	-2,09	0,020369	-1,92	0,85334	-1,09	0,750177	1,09	0,000172	2,09	7,63E-06	2,27
11734735_a_at	84188	FAR1	fatty acyl CoA reductase 1	0,003431	-1,83	0,020828	-1,71	0,864903	-1,07	0,000893	-2,02	0,313165	-1,18	0,554834	-1,10
11734734_s_at	84188	FAR1	fatty acyl CoA reductase 1	0,001735	-1,91	0,008769	-1,84	0,933803	-1,04	0,009593	-1,69	0,598985	1,09	0,437778	1,13
11724765_a_at	22868	FASTKD2	FAST kinase domains 2	0,000569	-1,76	0,009166	-1,60	0,722688	-1,10	0,055064	-1,35	0,156013	1,19	0,017218	1,31
11758250_s_at	26224	FBXL3	F-box and leucine-rich repeat protein 3	0,009215	-1,77	0,017839	-1,81	0,954859	1,03	0,900006	1,03	0,000323	1,87	0,000184	1,82
11728800_s_at	26235	FBXL4	F-box and leucine-rich repeat protein 4	0,000407	-1,79	0,004887	-1,67	0,796008	-1,07	0,04495	-1,36	0,089799	1,23	0,013904	1,32
11750639_s_at	80204	FBXO11	F-box protein 11	0,047198	-1,57	0,045541	-1,69	0,861587	1,08	0,089573	-1,48	0,47513	1,15	0,772689	1,06
11731068_s_at	63979	FIGNL1	fidgetin-like 1	0,001581	-2,35	0,01833	-2,03	0,754021	-1,16	0,00158	-2,35	0,507625	-1,15	0,995676	1,00
11751233_a_at	81608	FIP1L1	factor interacting with PAPOLA and CPSF1	0,000149	-1,73	0,001662	-1,66	0,861668	-1,04	0,000136	-1,72	0,7561	-1,04	0,950012	1,01
11759533_s_at	81608	FIP1L1	factor interacting with PAPOLA and CPSF1	0,000238	-1,87	0,000919	-1,89	0,971454	1,01	0,000689	-1,74	0,533455	1,08	0,598768	1,07
11743594_s_at	2332	FMR1	fragile X mental retardation 1	0,006622	-1,57	0,028087	-1,52	0,917107	-1,04	0,020993	-1,47	0,824112	1,03	0,623075	1,07
11741357_s_at	22862	FNDC3A	fibronectin type III domain containing 3A	0,001974	-1,96	0,036847	-1,66	0,645526	-1,18	8,43E-05	-2,47	0,016371	-1,49	0,154037	-1,26

11744985_a_at	22862	FNDC3A	fibronectin type III domain containing 3A	0,00847	-1,94	0,029725	-1,87	0,945576	-1,04	0,272516	-1,33	0,080988	1,41	0,039647	1,46
11717380_x_at	123811	FOPNL	FGFR1OP N-terminal like	0,020212	-1,51	0,041412	-1,52	0,989169	1,01	0,898344	1,03	0,001741	1,56	0,000807	1,55
11733092_x_at	80020	FOXRED2	FAD-dependent oxidoreductase domain containing 2	9,02E-05	-1,93	0,001398	-1,81	0,814084	-1,07	8,97E-08	-2,66	0,00137	-1,47	0,003792	-1,38
11743375_s_at	285527	FRYL	FRY-like	0,003587	-1,85	0,015582	-1,79	0,93698	-1,04	1,41E-05	-2,75	0,008679	-1,54	0,009587	-1,48
11746570_s_at	285527	FRYL	FRY-like	0,000688	-2,10	0,005343	-1,98	0,879021	-1,06	0,001059	-2,02	0,897052	-1,02	0,840669	1,04
11719715_a_at	8087	FXR1	fragile X mental retardation, autosomal homolog 1	0,00055	-1,56	0,004502	-1,51	0,881415	-1,04	0,006467	-1,40	0,454621	1,08	0,235557	1,12
11727381_a_at	84248	FYTTD1	forty-two-three domain containing 1	0,011356	-1,94	0,044805	-1,83	0,910983	-1,06	0,13803	1,48	4,94E-06	2,71	2,84E-07	2,87
11743478_a_at	84248	FYTTD1	forty-two-three domain containing 1	0,005855	-1,92	0,033934	-1,77	0,853426	-1,09	0,609621	1,14	0,000207	2,01	9,51E-06	2,18
11755470_x_at	55632	G2E3	G2/M-phase specific E3 ubiquitin protein ligase	0,00027	-2,03	0,009018	-1,73	0,600253	-1,18	0,001413	-1,81	0,768195	-1,05	0,418308	1,12
11753320_s_at	55632	G2E3	G2/M-phase specific E3 ubiquitin protein ligase	0,000272	-2,18	0,004635	-1,94	0,74147	-1,12	0,001856	-1,89	0,864231	1,03	0,348515	1,16
11739170_a_at	55632	G2E3	G2/M-phase specific E3 ubiquitin protein ligase	0,001437	-1,67	0,015136	-1,54	0,781804	-1,08	0,022847	-1,42	0,526336	1,08	0,177228	1,17
11746111_a_at	10146	G3BP1	GTPase activating protein (SH3 domain) binding protein 1	0,010554	-1,75	0,04707	-1,65	0,888031	-1,06	0,982258	1,01	0,003531	1,66	0,000425	1,76
11731638_a_at	2549	GAB1	GRB2-associated binding protein 1	0,03614	-1,55	0,03483	-1,67	0,853447	1,08	0,000198	-2,33	0,045551	-1,40	0,008646	-1,50
11755033_a_at	2551	GABPA	GA binding protein transcription factor, alpha subunit 60kDa	0,003472	-1,88	0,022491	-1,74	0,850078	-1,08	0,002049	-1,96	0,500413	-1,13	0,822743	-1,04
11743291_a_at	2589	GALNT1	polypeptide N-acetylgalactosaminyltransferase 1	0,00065	-1,63	0,00687	-1,53	0,808909	-1,06	0,010958	-1,41	0,463332	1,08	0,169299	1,15
11743290_at	2589	GALNT1	polypeptide N-acetylgalactosaminyltransferase 1	0,001135	-1,67	0,013495	-1,54	0,764997	-1,08	0,056544	-1,33	0,229616	1,16	0,043417	1,25
11722410_at	51809	GALNT7	polypeptide N-acetylgalactosaminyltransferase 7	0,008306	-1,66	0,025216	-1,63	0,971838	-1,02	1,08E-05	-2,57	0,002591	-1,58	0,001609	-1,55
11722409_at	51809	GALNT7	polypeptide N-acetylgalactosaminyltransferase 7	0,001184	-2,76	0,009574	-2,47	0,847843	-1,11	0,256002	-1,42	0,017486	1,75	0,002283	1,95
11722408_at	51809	GALNT7	polypeptide N-acetylgalactosaminyltransferase 7	0,000509	-2,08	0,003327	-2,01	0,928516	-1,04	9,93E-05	-2,30	0,395149	-1,15	0,537307	-1,11
11732760_at	202309	GAPT	GRB2-binding adaptor protein, transmembrane	0,015937	-1,67	0,015064	-1,83	0,819872	1,09	0,031835	-1,59	0,418018	1,15	0,781897	1,05
11719353_s_at	9648	GCC2	GRIP and coiled-coil domain containing 2	0,004677	-1,58	0,019641	-1,54	0,933748	-1,03	0,000113	-1,96	0,057462	-1,27	0,078294	-1,23
11719350_a_at	9648	GCC2	GRIP and coiled-coil domain containing 2	0,002649	-2,17	0,049947	-1,76	0,632339	-1,23	0,000266	-2,66	0,03685	-1,51	0,304543	-1,22
11719351_s_at	9648	GCC2	GRIP and coiled-coil domain containing 2	0,00211	-1,86	0,022768	-1,67	0,757369	-1,11	0,008853	-1,68	0,970424	-1,01	0,528554	1,11

11719352_x_at	9648	GCC2	GRIP and coiled-coil domain containing 2	0,002992	-1,66	0,018239	-1,57	0,872371	-1,05	0,003076	-1,66	0,719162	-1,05	0,999704	1,00
11737066_x_at	148823	GCSAML	germinal center-associated, signaling and motility-like	0,000342	-3,07	0,019306	-2,18	0,50322	-1,41	0,886873	1,05	0,000354	2,28	2,52E-07	3,22
11747576_a_at	79893	GGNBP2	gametogenetin binding protein 2	0,000272	-1,89	0,002029	-1,84	0,929568	-1,03	0,000698	-1,78	0,812791	1,03	0,637355	1,07
11723995_a_at	113263	GLCCI1	glucocorticoid induced transcript 1	0,017931	-1,52	0,011217	-1,69	0,731426	1,11	0,001224	-1,82	0,63292	-1,08	0,184355	-1,20
11760037_a_at	2744	GLS	glutaminase	0,004134	-2,54	0,015329	-2,46	0,963462	-1,03	0,971702	-1,01	0,000537	2,43	0,000104	2,51
11760038_a_at	2744	GLS	glutaminase	0,00313	-1,99	0,016894	-1,88	0,897972	-1,06	0,05321	-1,55	0,306029	1,21	0,153353	1,28
11763320_x_at	2744	GLS	glutaminase	0,004631	-1,83	0,021942	-1,74	0,908746	-1,05	0,037876	-1,55	0,506104	1,12	0,313493	1,18
11763693_a_at	2744	GLS	glutaminase	0,005584	-1,80	0,023519	-1,73	0,924842	-1,04	0,057019	-1,49	0,394462	1,16	0,247062	1,21
11759115_x_at	64395	GMCL1	germ cell-less, spermatogenesis associated 1	0,002592	-1,76	0,006154	-1,80	0,949828	1,02	0,527505	-1,13	0,00129	1,59	0,000925	1,55
11737747_a_at	2764	GMFB	glia maturation factor, beta	0,006093	-1,62	0,030069	-1,55	0,889298	-1,05	0,016431	-1,53	0,939936	1,01	0,682927	1,06
11727778_a_at	2773	GNAI3	guanine nucleotide binding protein (G protein), alpha inhibiting activity polypeptide 3	0,003011	-1,62	0,008131	-1,63	0,976593	1,01	0,979109	-1,00	0,000138	1,63	5,30E-05	1,61
11727776_at	2773	GNAI3	guanine nucleotide binding protein (G protein), alpha inhibiting activity polypeptide 3	0,006703	-1,61	0,029514	-1,55	0,909905	-1,04	0,392535	-1,17	0,040273	1,32	0,01191	1,38
11759094_a_at	2776	GNAQ	guanine nucleotide binding protein (G protein), q polypeptide	0,003357	-1,58	0,008488	-1,60	0,96935	1,01	0,364616	-1,15	0,006459	1,39	0,004848	1,37
11752677_a_at	29889	GNL2	guanine nucleotide binding protein-like 2 (nucleolar)	0,00136	-1,99	0,008665	-1,89	0,897031	-1,06	0,058181	-1,48	0,136457	1,28	0,052712	1,35
11749333_a_at	26354 /// 100113381	GNL3 /// SNORD19B	guanine nucleotide binding protein-like 3 (nucleolar) /// small nucleolar RNA, C/D box	0,00221	-2,20	0,040561	-1,80	0,645111	-1,22	0,005519	-2,03	0,56963	-1,13	0,705959	1,08
11721866_a_at	132789	GNPDA2	glucosamine-6-phosphate deaminase 2	0,000839	-1,61	0,007042	-1,53	0,851037	-1,05	0,628862	-1,07	0,000901	1,43	5,39E-05	1,50
11719318_a_at	2803	GOLGA4	golgin A4	0,010082	-1,57	0,018364	-1,61	0,946976	1,03	7,46E-05	-2,13	0,041965	-1,32	0,017796	-1,36
11736122_a_at	27333	GOLIM4	golgi integral membrane protein 4	0,000857	-2,46	0,004965	-2,34	0,929762	-1,05	0,871984	-1,05	1,00E-04	2,23	9,65E-06	2,34
11741271_a_at	57120	GOPC	golgi-associated PDZ and coiled-coil motif containing	0,007161	-1,89	0,024324	-1,84	0,957817	-1,03	0,001612	-2,15	0,406827	-1,17	0,487719	-1,14
11721649_a_at	54865	GPATCH4	G patch domain containing 4	0,000147	-1,81	0,00426	-1,62	0,656915	-1,12	4,65E-06	-2,09	0,020772	-1,29	0,172609	-1,16

11754325_a_at	23131	GPATCH8	G patch domain containing 8	0,001569	-2,11	0,012553	-1,93	0,840988	-1,09	0,021861	-1,69	0,468696	1,15	0,202133	1,25
11723109_a_at	65056	GPBP1	GC-rich promoter binding protein 1	0,000976	-2,52	0,011424	-2,20	0,769917	-1,15	0,89641	1,04	0,000124	2,29	2,21E-06	2,63
11739427_a_at	2820	GPD2	glycerol-3-phosphate dehydrogenase 2 (mitochondrial)	0,003364	-1,61	0,014254	-1,57	0,945291	-1,02	0,300163	-1,18	0,023207	1,33	0,008309	1,36
11741092_a_at	2820	GPD2	glycerol-3-phosphate dehydrogenase 2 (mitochondrial)	0,001224	-1,61	0,012565	-1,51	0,794772	-1,07	0,000796	-1,65	0,459555	-1,09	0,880237	-1,02
11739977_at	160897	GPR180	G protein-coupled receptor 180	0,001101	-1,82	0,010347	-1,69	0,816362	-1,08	0,01571	-1,53	0,502182	1,10	0,199158	1,19
11720967_a_at	29899	GPSM2	G-protein signaling modulator 2	0,002565	-1,85	0,02662	-1,66	0,754929	-1,12	0,004945	-1,77	0,708802	-1,07	0,795763	1,05
11724786_s_at	51527	GSKIP	GSK3B interacting protein	0,000728	-1,70	0,015496	-1,51	0,656915	-1,12	0,13247	-1,25	0,1021	1,21	0,004742	1,36
11742750_a_at	2935	GSPT1	G1 to S phase transition 1	0,00285	-1,70	0,029755	-1,54	0,750347	-1,10	0,405797	-1,16	0,038005	1,33	0,002685	1,46
11753511_a_at	2963	GTF2F2	general transcription factor IIF, polypeptide 2, 30kDa	1,92E-06	-2,27	5,25E-05	-2,11	0,772892	-1,08	7,85E-06	-2,03	0,760661	1,04	0,315221	1,12
11739041_x_at	2965	GTF2H1	general transcription factor IIH, polypeptide 1, 62kDa	0,006091	-1,48	0,011052	-1,52	0,926108	1,03	0,757081	-1,05	0,001009	1,45	0,000925	1,41
11742758_a_at	2965	GTF2H1	general transcription factor IIH, polypeptide 1, 62kDa	0,002145	-1,79	0,006483	-1,80	0,985887	1,01	0,354381	-1,19	0,004412	1,51	0,002525	1,50
11749493_a_at	2965	GTF2H1	general transcription factor IIH, polypeptide 1, 62kDa	0,001924	-1,72	0,017167	-1,60	0,8005	-1,08	0,336601	-1,18	0,024778	1,35	0,002414	1,46
11749494_x_at	2965	GTF2H1	general transcription factor IIH, polypeptide 1, 62kDa	0,00237	-1,72	0,01203	-1,66	0,921453	-1,04	0,265826	-1,22	0,022813	1,36	0,006428	1,41
11753672_s_at	2966 /// 653238 /// 728340 /// 730394	GTF2H2 /// GTF2H2B /// GTF2H2C /// GTF2H2C_2	general transcription factor IIH, polypeptide 2, 44kDa /// general transcription factor	0,016316	-1,68	0,042043	-1,66	0,979748	-1,01	0,013676	-1,71	0,861568	-1,04	0,915678	-1,02
11749979_s_at	2966 /// 653238 /// 728340 /// 730394	GTF2H2 /// GTF2H2B /// GTF2H2C /// GTF2H2C_2	general transcription factor IIH, polypeptide 2, 44kDa /// general transcription factor	0,02853	-1,62	0,023663	-1,78	0,812802	1,10	0,085729	-1,46	0,274751	1,21	0,593093	1,10

11753453_s_at	2966 /// 653238 /// 728340 /// 730394	GTF2H2 /// GTF2H2B /// GTF2H2C /// GTF2H2C_2	general transcription factor IIH, polypeptide 2, 44kDa /// general transcription factor	0,028793	-1,53	0,043189	-1,57	0,942867	1,03	0,018342	-1,59	0,951926	-1,01	0,8131	-1,04
11749438_a_at	9330	GTF3C3	general transcription factor IIIC, polypeptide 3, 102kDa	0,002596	-1,84	0,026573	-1,65	0,758577	-1,11	0,799807	1,06	0,000403	1,75	7,52E-06	1,95
11722140_a_at	2982	GUCY1A3	guanylate cyclase 1, soluble, alpha 3	0,000437	-2,73	0,015445	-2,10	0,571804	-1,30	0,048521	-1,69	0,304866	1,25	0,013453	1,62
11741088_a_at	2982	GUCY1A3	guanylate cyclase 1, soluble, alpha 3	0,000383	-2,53	0,006865	-2,16	0,710195	-1,17	0,003348	-2,07	0,851599	1,04	0,28955	1,22
11739830_a_at	2982	GUCY1A3	guanylate cyclase 1, soluble, alpha 3	0,000458	-2,56	0,012747	-2,06	0,617846	-1,24	0,003619	-2,11	0,926548	-1,02	0,319197	1,22
11749919_a_at	2982	GUCY1A3	guanylate cyclase 1, soluble, alpha 3	0,000516	-3,40	0,016599	-2,49	0,587932	-1,36	0,004239	-2,62	0,866044	-1,05	0,315605	1,30
11722137_a_at	2982	GUCY1A3	guanylate cyclase 1, soluble, alpha 3	0,000759	-2,17	0,017797	-1,82	0,635793	-1,20	0,008246	-1,79	0,95084	1,01	0,253226	1,21
11722138_s_at	2982	GUCY1A3	guanylate cyclase 1, soluble, alpha 3	0,001153	-2,31	0,02821	-1,86	0,61209	-1,24	0,013223	-1,85	0,98606	1,00	0,245825	1,25
11724161_a_at	283464	GXYLT1	glucoside xylosyltransferase 1	0,000145	-1,93	0,0057	-1,67	0,593296	-1,16	0,011151	-1,49	0,390699	1,12	0,02719	1,29
11723254_a_at	8520	HAT1	histone acetyltransferase 1	0,000362	-1,65	0,003632	-1,57	0,845955	-1,05	0,000185	-1,68	0,530484	-1,07	0,86494	-1,02
11760154_at	79441	HAUS3	HAUS augmin-like complex, subunit 3	0,001454	-1,87	0,016408	-1,69	0,764757	-1,11	0,001025	-1,91	0,42879	-1,13	0,903346	-1,02
11750151_a_at	54801	HAUS6	HAUS augmin-like complex, subunit 6	0,00208	-2,22	0,008518	-2,17	0,967259	-1,02	0,474472	-1,21	0,003229	1,80	0,000926	1,84
11727487_at	54801	HAUS6	HAUS augmin-like complex, subunit 6	0,001144	-2,10	0,016494	-1,83	0,721084	-1,15	0,000746	-2,16	0,348013	-1,18	0,885863	-1,03
11727488_s_at	54801	HAUS6	HAUS augmin-like complex, subunit 6	0,000745	-2,54	0,017093	-2,05	0,641196	-1,24	0,002885	-2,22	0,72746	-1,08	0,535692	1,14
11722415_a_at	10767	HBS1L	HBS1-like translational GTPase	0,001317	-2,17	0,010818	-1,99	0,841438	-1,09	0,003926	-1,98	0,984159	1,00	0,640348	1,10
11745741_a_at	10767	HBS1L	HBS1-like translational GTPase	0,002671	-1,68	0,013698	-1,62	0,915855	-1,04	0,061576	-1,37	0,214469	1,18	0,108106	1,22
11727479_a_at	25831	HECTD1	HECT domain containing E3 ubiquitin protein ligase 1	0,011196	-1,66	0,038101	-1,61	0,941432	-1,03	0,006951	-1,73	0,671861	-1,08	0,819039	-1,04
11749691_a_at	3070	HELLS	helicase, lymphoid-specific	0,000854	-2,06	0,018111	-1,75	0,650516	-1,18	0,010229	-1,71	0,888714	1,03	0,232654	1,21
11746467_a_at	3070	HELLS	helicase, lymphoid-specific	0,001662	-2,03	0,02821	-1,73	0,672211	-1,17	0,005882	-1,84	0,746024	-1,06	0,582857	1,10
11724781_a_at	26091	HERC4	HECT and RLD domain containing E3 ubiquitin protein ligase 4	0,007336	-1,57	0,0138	-1,61	0,938579	1,03	0,004334	-1,63	0,95351	-1,01	0,80608	-1,04

11751957_a_at	26091	HERC4	HECT and RLD domain containing E3 ubiquitin protein ligase 4	0,008296	-1,55	0,021594	-1,55	0,997566	-1,00	0,004019	-1,63	0,730954	-1,05	0,735884	-1,05
11758560_s_at	26091	HERC4	HECT and RLD domain containing E3 ubiquitin protein ligase 4	0,007342	-1,62	0,02176	-1,60	0,979982	-1,01	0,005844	-1,65	0,853834	-1,03	0,907888	-1,02
11724782_x_at	26091	HERC4	HECT and RLD domain containing E3 ubiquitin protein ligase 4	0,011573	-1,53	0,022924	-1,56	0,968047	1,02	0,011553	-1,54	0,947482	1,01	0,975987	-1,01
11737098_a_at	6596	HLTF	helicase-like transcription factor	0,000452	-3,14	0,0057	-2,70	0,781421	-1,16	0,057724	-1,78	0,082526	1,51	0,010756	1,76
11739025_a_at	3146	HMGB1	high mobility group box 1	0,004877	-1,57	0,012311	-1,59	0,97894	1,01	7,73E-05	-1,98	0,076245	-1,25	0,049455	-1,26
11729348_at	79366	HMGN5	high mobility group nucleosome binding domain 5	0,001046	-2,33	0,029766	-1,85	0,585833	-1,26	0,00248	-2,15	0,450461	-1,17	0,706691	1,08
11723201_s_at	220988	HNRNPA3	heterogeneous nuclear ribonucleoprotein A3	6,12E-05	-3,02	0,002519	-2,47	0,639306	-1,22	0,00304	-2,11	0,443288	1,17	0,05057	1,43
11723197_at	220988	HNRNPA3	heterogeneous nuclear ribonucleoprotein A3	0,000359	-1,81	0,01469	-1,54	0,550535	-1,17	0,00023	-1,83	0,163078	-1,18	0,939683	-1,01
11722000_a_at	3192	HNRNPU	heterogeneous nuclear ribonucleoprotein U (scaffold attachment factor A)	0,000152	-2,06	0,004254	-1,81	0,66374	-1,14	7,34E-05	-2,12	0,256405	-1,17	0,87001	-1,03
11763725_a_at	3192	HNRNPU	heterogeneous nuclear ribonucleoprotein U (scaffold attachment factor A)	0,00056	-1,91	0,010419	-1,69	0,696417	-1,13	0,03076	-1,47	0,308437	1,16	0,041409	1,30
11744381_s_at	284702	HNRNPU-AS1	HNRNPU antisense RNA 1	0,000175	-3,03	0,007636	-2,32	0,571804	-1,30	0,000698	-2,60	0,617044	-1,12	0,490281	1,16
11725665_at	9653	HS2ST1	heparan sulfate 2-O-sulfotransferase 1	0,000287	-1,94	0,003869	-1,79	0,793119	-1,08	4,92E-05	-2,12	0,211285	-1,18	0,523737	-1,09
11718426_a_at	84263	HSDL2	hydroxysteroid dehydrogenase like 2	0,015458	-1,53	0,034323	-1,54	0,994718	1,00	0,490752	1,14	9,63E-05	1,75	2,65E-05	1,74
11718427_s_at	84263	HSDL2	hydroxysteroid dehydrogenase like 2	0,022418	-1,51	0,046937	-1,51	0,994647	1,00	0,875355	-1,03	0,007673	1,46	0,004332	1,46
11758308_s_at	3320	HSP90AA1	heat shock protein 90kDa alpha (cytosolic), class A member 1	0,002982	-1,64	0,010612	-1,63	0,979567	-1,01	0,1623	-1,26	0,045485	1,29	0,02689	1,31
11749934_s_at	3323	HSP90AA4P	heat shock protein 90kDa alpha (cytosolic), class A member 4, pseudogene	0,002394	-1,60	0,007366	-1,61	0,990765	1,00	0,31551	-1,17	0,00713	1,37	0,004199	1,37
11761734_x_at	3323	HSP90AA4P	heat shock protein 90kDa alpha (cytosolic), class A member 4, pseudogene	0,001813	-1,52	0,004657	-1,55	0,948456	1,02	0,140974	-1,21	0,016302	1,28	0,017327	1,25
11752967_x_at	7184	HSP90B1	heat shock protein 90kDa beta (Grp94), member 1	7,04E-05	-2,61	0,001839	-2,28	0,721044	-1,15	0,00074	-2,15	0,759691	1,06	0,244281	1,21
11753260_x_at	7184 /// 100500842	HSP90B1 /// MIR3652	heat shock protein 90kDa beta (Grp94), member 1 /// microRNA 3652	5,98E-05	-3,03	0,001068	-2,70	0,794708	-1,12	0,000524	-2,46	0,665181	1,10	0,278208	1,23

11754606_a_at	7184 /// 100500842	HSP90B1 /// MIR3652	heat shock protein 90kDa beta (Grp94), member 1 /// microRNA 3652	0,000147	-3,57	0,001521	-3,26	0,878808	-1,10	0,001874	-2,66	0,415675	1,23	0,205035	1,34
11741912_a_at	7184 /// 100500842	HSP90B1 /// MIR3652	heat shock protein 90kDa beta (Grp94), member 1 /// microRNA 3652	0,002096	-1,96	0,011634	-1,86	0,90871	-1,05	0,0004	-2,21	0,313868	-1,19	0,473575	-1,13
11758770_at	3308	HSPA4	heat shock 70kDa protein 4	0,002065	-1,83	0,029846	-1,61	0,696022	-1,14	0,004836	-1,73	0,653439	-1,08	0,72576	1,06
11752595_a_at	3313	HSPA9	heat shock 70kDa protein 9 (mortalin)	0,001531	-2,21	0,017093	-1,95	0,764582	-1,14	0,040514	-1,64	0,389229	1,19	0,098772	1,35
11746986_x_at	3329	HSPD1	heat shock 60kDa protein 1 (chaperonin)	8,53E-05	-2,51	0,001672	-2,24	0,764957	-1,12	0,036331	-1,55	0,026385	1,44	0,001847	1,61
11753017_s_at	3329	HSPD1	heat shock 60kDa protein 1 (chaperonin)	0,0002	-2,75	0,004081	-2,35	0,719535	-1,17	0,043309	-1,65	0,07301	1,42	0,005207	1,66
11753018_x_at	3329	HSPD1	heat shock 60kDa protein 1 (chaperonin)	0,000474	-1,93	0,008621	-1,72	0,70145	-1,13	0,106932	-1,33	0,06346	1,29	0,003548	1,45
11745851_x_at	3329	HSPD1	heat shock 60kDa protein 1 (chaperonin)	0,001241	-1,66	0,01051	-1,57	0,838738	-1,06	0,163277	-1,24	0,044362	1,27	0,007357	1,34
11717096_a_at	10808	HSPH1	heat shock 105kDa/110kDa protein 1	0,000141	-2,75	0,004735	-2,24	0,628629	-1,22	0,176891	-1,39	0,011184	1,62	0,000123	1,98
11747105_a_at	10808	HSPH1	heat shock 105kDa/110kDa protein 1	0,001199	-3,20	0,02096	-2,49	0,678125	-1,28	0,953993	-1,02	0,001143	2,44	1,05E-05	3,13
11748072_a_at	10808	HSPH1	heat shock 105kDa/110kDa protein 1	0,000238	-3,17	0,003841	-2,72	0,764582	-1,17	0,222298	-1,43	0,004834	1,90	0,000181	2,22
11755205_a_at	10808	HSPH1	heat shock 105kDa/110kDa protein 1	0,000631	-2,77	0,011234	-2,29	0,700735	-1,21	0,407299	-1,27	0,007842	1,80	0,000173	2,17
11755206_x_at	10808	HSPH1	heat shock 105kDa/110kDa protein 1	0,002444	-2,03	0,040292	-1,71	0,663048	-1,19	0,851983	-1,05	0,006625	1,63	8,90E-05	1,94
11749659_s_at	10808	HSPH1	heat shock 105kDa/110kDa protein 1	0,00197	-2,39	0,023063	-2,04	0,740128	-1,17	0,600359	-1,17	0,00922	1,75	0,000338	2,05
11720270_a_at	25998	IBTK	inhibitor of Bruton agammaglobulinemia tyrosine kinase	0,000106	-2,22	0,004478	-1,88	0,597258	-1,19	0,001778	-1,82	0,866044	1,03	0,160159	1,22
11720268_a_at	25998	IBTK	inhibitor of Bruton agammaglobulinemia tyrosine kinase	0,013576	-1,88	0,036268	-1,85	0,979295	-1,02	0,750552	-1,09	0,009168	1,69	0,003889	1,71
11720269_a_at	25998	IBTK	inhibitor of Bruton agammaglobulinemia tyrosine kinase	0,002483	-1,76	0,014079	-1,68	0,89828	-1,05	0,001254	-1,84	0,547113	-1,10	0,780886	-1,05
11722630_a_at	79664	ICE2	interactor of little elongator complex ELL subunit 2	0,001581	-1,84	0,011193	-1,73	0,868855	-1,06	0,010091	-1,62	0,682237	1,07	0,396361	1,13
11722631_s_at	79664	ICE2	interactor of little elongator complex ELL subunit 2	0,001437	-1,80	0,020398	-1,60	0,714489	-1,12	0,011477	-1,57	0,901613	1,02	0,332552	1,14
11722632_x_at	79664	ICE2	interactor of little elongator complex ELL subunit 2	0,002173	-1,59	0,014525	-1,51	0,868424	-1,05	0,020871	-1,40	0,540371	1,08	0,283739	1,13
11716795_s_at	3428	IFI16	interferon, gamma-inducible protein 16	0,002154	-2,02	0,020538	-1,81	0,78465	-1,12	0,000262	-2,37	0,120614	-1,31	0,354563	-1,18
11716796_s_at	3428	IFI16	interferon, gamma-inducible protein 16	0,00721	-1,74	0,023133	-1,71	0,967817	-1,02	0,001902	-1,93	0,468989	-1,13	0,537005	-1,11

11747926_s_at	3428	IFI16	interferon, gamma-inducible protein 16	0,009335	-1,63	0,029676	-1,60	0,960128	-1,02	0,003145	-1,77	0,520106	-1,11	0,61154	-1,08
11724346_a_at	64135	IFIH1	interferon induced with helicase C domain 1	0,003735	-1,81	0,023511	-1,68	0,853971	-1,07	0,274158	-1,25	0,058598	1,35	0,012246	1,44
11748615_a_at	3454	IFNAR1	interferon (alpha, beta and omega) receptor 1	0,000163	-2,20	0,000601	-2,25	0,957043	1,02	0,027982	-1,52	0,008995	1,48	0,008178	1,45
11723423_x_at	3454	IFNAR1	interferon (alpha, beta and omega) receptor 1	6,35E-05	-1,82	0,00074	-1,74	0,872047	-1,04	0,002178	-1,53	0,208117	1,14	0,077462	1,19
11723424_at	3454	IFNAR1	interferon (alpha, beta and omega) receptor 1	0,030189	-1,51	0,045541	-1,55	0,945048	1,03	0,180765	-1,30	0,240771	1,20	0,313041	1,16
11735527_a_at	80173	IFT74	intraflagellar transport 74 homolog (Chlamydomonas)	0,000191	-2,16	0,015922	-1,70	0,476225	-1,28	0,033502	-1,50	0,423525	1,13	0,007741	1,45
11724626_a_at	80173	IFT74	intraflagellar transport 74 homolog (Chlamydomonas)	0,000816	-1,68	0,015628	-1,51	0,673632	-1,11	0,002885	-1,57	0,766059	-1,04	0,566311	1,07
11732397_a_at	57560	IFT80	intraflagellar transport 80 homolog (Chlamydomonas)	0,001612	-2,12	0,019108	-1,86	0,748228	-1,14	2,40E-05	-2,93	0,011352	-1,58	0,055639	-1,38
11735715_a_at	28981	IFT81	intraflagellar transport 81 homolog (Chlamydomonas)	0,000625	-1,78	0,006373	-1,67	0,816394	-1,07	7,59E-06	-2,24	0,018106	-1,34	0,053678	-1,26
11728030_a_at	285313	IGSF10	immunoglobulin superfamily, member 10	0,341275	-1,37	0,020144	-2,37	0,413308	1,73	0,008059	-2,37	0,989888	-1,00	0,019569	-1,74
11759037_s_at	3572	IL6ST	interleukin 6 signal transducer	0,039064	-1,57	0,031588	-1,72	0,814146	1,10	0,369834	-1,23	0,050222	1,40	0,143712	1,28
11754162_a_at	3574	IL7	interleukin 7	0,002176	-1,79	0,049282	-1,51	0,602457	-1,18	0,664481	-1,09	0,023132	1,39	0,000263	1,64
11719142_a_at	51141	INSIG2	insulin induced gene 2	0,008984	-1,72	0,045468	-1,61	0,865309	-1,07	0,057158	-1,48	0,650292	1,08	0,365167	1,16
11733458_s_at	57508	INTS2	integrator complex subunit 2	0,005439	-1,69	0,021839	-1,64	0,935023	-1,03	0,00363	-1,75	0,690458	-1,07	0,852692	-1,03
11752061_x_at	26512	INTS6	integrator complex subunit 6	0,001357	-1,69	0,008131	-1,63	0,910773	-1,04	0,382499	-1,15	0,005301	1,42	0,000899	1,47
11754834_x_at	26512	INTS6	integrator complex subunit 6	0,001038	-1,86	0,003211	-1,90	0,958547	1,02	0,129487	-1,32	0,009696	1,44	0,00876	1,41
11727521_s_at	26512	INTS6	integrator complex subunit 6	0,005605	-1,69	0,004716	-1,87	0,75183	1,11	0,124494	-1,33	0,020656	1,40	0,094426	1,26
11756772_a_at	55656	INTS8	integrator complex subunit 8	0,002384	-1,56	0,005963	-1,59	0,955804	1,02	0,038477	-1,34	0,138095	1,18	0,168068	1,16
11721612_a_at	51194 /// 101180901	IPO11 /// IPO11-LRRC70	importin 11 /// IPO11-LRRC70 readthrough	0,000142	-1,71	0,005153	-1,52	0,609656	-1,12	0,000449	-1,60	0,639574	-1,05	0,555338	1,06
11725672_s_at	10527	IPO7	importin 7	0,00019	-2,03	0,007315	-1,73	0,590937	-1,18	4,47E-05	-2,18	0,08918	-1,26	0,62839	-1,07
11757753_s_at	10527	IPO7	importin 7	0,000532	-1,94	0,004722	-1,83	0,861343	-1,06	0,000146	-2,08	0,367687	-1,14	0,637471	-1,07
11725670_s_at	10527	IPO7	importin 7	0,001866	-1,76	0,037005	-1,52	0,635793	-1,16	0,000169	-2,03	0,035031	-1,34	0,288647	-1,16
11716766_a_at	8826	IQGAP1	IQ motif containing GTPase activating protein 1	0,035385	-1,75	0,027264	-1,97	0,79918	1,13	0,979874	1,01	0,001263	1,99	0,003921	1,76
11716767_at	8826	IQGAP1	IQ motif containing GTPase activating protein 1	0,05692	-1,39	0,009605	-1,70	0,516984	1,22	0,325779	-1,19	0,010469	1,42	0,264168	1,16
11721061_a_at	10788	IQGAP2	IQ motif containing GTPase activating protein 2	5,98E-05	-2,46	0,001085	-2,23	0,786226	-1,10	0,001533	-1,93	0,362899	1,16	0,10057	1,28

11745509_a_at	10788	IQGAP2	IQ motif containing GTPase activating protein 2	0,003503	-1,75	0,016165	-1,69	0,926254	-1,04	0,005665	-1,70	0,970236	-1,01	0,861424	1,03
11721363_x_at	3658	IREB2	iron-responsive element binding protein 2	0,002744	-1,68	0,025093	-1,54	0,782959	-1,09	1,41E-05	-2,27	0,003699	-1,47	0,014377	-1,35
11756521_a_at	3658	IREB2	iron-responsive element binding protein 2	0,000977	-2,22	0,008231	-2,04	0,844885	-1,09	2,37E-05	-2,94	0,041895	-1,44	0,102148	-1,33
11721364_s_at	3658	IREB2	iron-responsive element binding protein 2	0,002906	-2,04	0,027691	-1,80	0,769895	-1,13	0,000134	-2,62	0,042113	-1,45	0,158305	-1,28
11749666_a_at	83737	ITCH	itchy E3 ubiquitin protein ligase	0,000404	-1,74	0,003211	-1,67	0,8947	-1,04	0,527579	-1,10	0,000323	1,52	2,54E-05	1,58
11745275_x_at	83737	ITCH	itchy E3 ubiquitin protein ligase	0,001877	-1,65	0,022411	-1,50	0,737051	-1,10	0,111105	-1,28	0,198364	1,17	0,028164	1,29
11724345_at	3676	ITGA4	integrin, alpha 4 (antigen CD49D, alpha 4 subunit of VLA-4 receptor)	0,007619	-1,70	0,041331	-1,59	0,857824	-1,07	0,935222	1,02	0,002141	1,62	0,000166	1,73
11723180_a_at	50618	ITSN2	intersectin 2	0,001097	-1,95	0,009084	-1,81	0,843754	-1,08	0,0006	-2,02	0,498484	-1,12	0,833225	-1,04
11717669_a_at	55677	IWS1	IWS1 homolog (S. cerevisiae)	0,002141	-1,52	0,004766	-1,56	0,927108	1,03	0,008398	-1,42	0,403574	1,09	0,550473	1,07
11725072_at	79960	JADE1	jade family PHD finger 1	0,000191	-1,86	0,001995	-1,78	0,878346	-1,05	3,88E-07	-2,49	0,005168	-1,40	0,009346	-1,34
11725069_s_at	79960	JADE1	jade family PHD finger 1	0,001547	-1,52	0,003869	-1,55	0,935818	1,02	0,000147	-1,68	0,443708	-1,08	0,303939	-1,11
11733372_a_at	3717	JAK2	Janus kinase 2	0,009217	-1,74	0,028217	-1,71	0,96775	-1,02	0,725427	1,09	0,00029	1,86	5,48E-05	1,89
11733373_at	3717	JAK2	Janus kinase 2	0,000859	-1,86	0,01292	-1,66	0,72182	-1,12	0,132682	-1,31	0,080085	1,27	0,006124	1,42
11756857_a_at	3717	JAK2	Janus kinase 2	0,005198	-1,64	0,024997	-1,57	0,900353	-1,05	0,478184	-1,14	0,019937	1,38	0,004388	1,44
11741235_a_at	51528	JKAMP	JNK1/MAPK8-associated membrane protein	0,003273	-1,68	0,02235	-1,57	0,840789	-1,07	0,110108	-1,32	0,199801	1,19	0,059557	1,27
11718636_a_at	221037	JMJD1C	jumonji domain containing 1C	0,023992	-1,60	0,027287	-1,71	0,881947	1,06	0,012065	-1,71	0,997142	-1,00	0,729824	-1,07
11746223_a_at	221037	JMJD1C	jumonji domain containing 1C	0,037836	-1,77	0,041967	-1,92	0,887834	1,08	0,026988	-1,86	0,904005	1,03	0,852068	-1,05
11740234_a_at	23522	KAT6B	K(lysine) acetyltransferase 6B	0,006181	-1,74	0,017359	-1,73	0,995017	-1,00	0,000264	-2,17	0,14903	-1,26	0,136092	-1,25
11727275_a_at	143888	KDELC2	KDEL (Lys-Asp-Glu-Leu) containing 2	0,00232	-1,86	0,010966	-1,80	0,936686	-1,03	5,94E-05	-2,38	0,070337	-1,32	0,095168	-1,28
11726730_a_at	9682	KDM4A	lysine (K)-specific demethylase 4A	0,003134	-1,63	0,016877	-1,56	0,898626	-1,04	0,05986	-1,36	0,275415	1,15	0,13363	1,20
11743522_a_at	5927	KDM5A	lysine (K)-specific demethylase 5A	0,00235	-1,59	0,010419	-1,56	0,947465	-1,02	0,000707	-1,70	0,49555	-1,09	0,613503	-1,06
11743525_a_at	5927	KDM5A	lysine (K)-specific demethylase 5A	0,003123	-1,61	0,012495	-1,58	0,958741	-1,02	0,003197	-1,61	0,897334	-1,02	0,996741	-1,00
11748491_a_at	10657	KHDRBS1	KH domain containing, RNA binding, signal transduction associated 1	0,003496	-1,59	0,014729	-1,55	0,945403	-1,02	0,015385	-1,46	0,652155	1,06	0,511573	1,09
11715937_a_at	10657	KHDRBS1	KH domain containing, RNA binding, signal transduction associated 1	0,004677	-1,73	0,023447	-1,64	0,895431	-1,05	0,006397	-1,70	0,852089	-1,03	0,921838	1,02

11719725_at	9933	KIAA0020	KIAA0020	0,001018	-1,73	0,015955	-1,56	0,706852	-1,11	0,003045	-1,62	0,77519	-1,04	0,625999	1,07
11722628_at	23325	KIAA1033	KIAA1033	0,000976	-2,10	0,0036	-2,12	0,990765	1,01	0,000487	-2,20	0,836885	-1,04	0,812005	-1,05
11722626_at	23325	KIAA1033	KIAA1033	0,004304	-1,68	0,006945	-1,77	0,889867	1,05	0,000551	-1,92	0,580328	-1,09	0,344488	-1,14
11722627_at	23325	KIAA1033	KIAA1033	0,017092	-1,92	0,021502	-2,07	0,890772	1,08	0,307966	-1,33	0,041755	1,55	0,076457	1,44
11722699_a_at	84162	KIAA1109	KIAA1109	0,002597	-1,96	0,021124	-1,78	0,809699	-1,10	0,18637	-1,34	0,094654	1,33	0,016753	1,47
11722700_a_at	84162	KIAA1109	KIAA1109	0,017869	-1,62	0,024208	-1,70	0,906915	1,05	0,005142	-1,79	0,765877	-1,06	0,54117	-1,11
11743186_a_at	57587	KIAA1430	KIAA1430	0,001641	-1,80	0,010988	-1,70	0,880592	-1,06	0,00017	-2,06	0,174713	-1,21	0,320018	-1,15
11743187_at	57587	KIAA1430	KIAA1430	0,000839	-2,18	0,007372	-2,01	0,842132	-1,09	0,015843	-1,71	0,378871	1,17	0,150064	1,27
11732297_at	80856	KIAA1715	KIAA1715	0,002182	-1,63	0,009115	-1,61	0,961318	-1,02	0,97071	-1,01	0,000188	1,60	3,04E-05	1,62
11727165_a_at	85459	KIAA1731	KIAA1731	0,000779	-1,80	0,009233	-1,65	0,775475	-1,09	8,99E-06	-2,29	0,012175	-1,38	0,04912	-1,27
11723900_a_at	205717	KIAA2018	KIAA2018	9,25E-05	-1,59	0,000519	-1,58	0,990802	-1,00	4,17E-05	-1,62	0,825776	-1,02	0,853014	-1,02
11727594_a_at	158358	KIAA2026	KIAA2026	0,004083	-1,60	0,023181	-1,52	0,876389	-1,05	0,00026	-1,87	0,102888	-1,23	0,201915	-1,17
11743331_a_at	57498	KIDINS220	kinase D-interacting substrate, 220kDa	4,17E-05	-1,77	0,000379	-1,74	0,938301	-1,02	5,91E-05	-1,72	0,931384	1,01	0,78143	1,03
11746232_a_at	57498	KIDINS220	kinase D-interacting substrate, 220kDa	0,003417	-1,59	0,006427	-1,64	0,914564	1,03	0,001182	-1,68	0,852932	-1,03	0,647036	-1,06
11731888_a_at	9928	KIF14	kinesin family member 14	0,004724	-1,95	0,025476	-1,82	0,881696	-1,07	0,090395	-1,49	0,280663	1,22	0,122499	1,31
11725716_a_at	55605	KIF21A	kinesin family member 21A	0,083268	-1,37	0,007522	-1,77	0,447888	1,30	0,037144	-1,46	0,186483	1,21	0,651834	-1,07
11757982_s_at	55605	KIF21A	kinesin family member 21A	0,240629	-1,28	0,024721	-1,72	0,455116	1,34	0,025525	-1,60	0,696549	1,07	0,162512	-1,25
11756918_a_at	9493	KIF23	kinesin family member 23	0,004996	-2,20	0,021023	-2,09	0,928881	-1,05	0,214138	-1,42	0,076307	1,47	0,031956	1,55
11721932_a_at	9493	KIF23	kinesin family member 23	0,005095	-2,11	0,035336	-1,89	0,818182	-1,12	0,043076	-1,70	0,652258	1,11	0,300864	1,24
11750773_a_at	3796	KIF2A	kinesin heavy chain member 2A	0,017325	-1,74	0,014634	-1,94	0,795805	1,12	0,99886	1,00	0,000395	1,94	0,001218	1,74
11758822_at	3796	KIF2A	kinesin heavy chain member 2A	0,009793	-1,52	0,017582	-1,56	0,943039	1,03	0,268633	-1,20	0,040048	1,30	0,049338	1,26
11746185_a_at	3799	KIF5B	kinesin family member 5B	0,001357	-1,70	0,004887	-1,70	0,998012	1,00	0,448281	1,13	9,54E-07	1,93	1,64E-07	1,93
11736060_a_at	3799	KIF5B	kinesin family member 5B	0,001126	-1,91	0,003211	-1,96	0,946785	1,03	0,483395	-1,15	0,000434	1,71	0,000283	1,66
11757809_s_at	3799	KIF5B	kinesin family member 5B	0,00208	-1,61	0,009339	-1,57	0,948456	-1,02	0,057976	-1,33	0,150714	1,19	0,08838	1,21
11739068_a_at	22920	KIFAP3	kinesin-associated protein 3	0,001345	-1,98	0,004822	-1,98	0,995745	1,00	0,710915	-1,09	0,000248	1,82	7,68E-05	1,82
11756551_a_at	54813	KLHL28	kelch-like family member 28	0,013999	-1,51	0,021377	-1,56	0,924842	1,03	0,243231	-1,22	0,063181	1,28	0,092729	1,24
11721054_at	57542	KLHL42	kelch-like family member 42	6,94E-05	-2,16	0,000812	-2,04	0,875506	-1,06	4,15E-06	-2,48	0,154086	-1,22	0,293731	-1,15

11739747_a_at	55975	KLHL7	kelch-like family member 7	0,014871	-1,42	0,010802	-1,54	0,759201	1,08	0,389466	-1,14	0,008247	1,35	0,038123	1,25
11739748_s_at	55975	KLHL7	kelch-like family member 7	0,016612	-1,45	0,021162	-1,51	0,891876	1,04	0,020664	-1,44	0,708995	1,05	0,956552	1,01
11755157_x_at	57563	KLHL8	kelch-like family member 8	0,000146	-2,00	0,001283	-1,93	0,908232	-1,04	4,39E-07	-2,68	0,011678	-1,39	0,017413	-1,34
11731139_s_at	57563	KLHL8	kelch-like family member 8	0,000179	-1,80	0,000297	-1,92	0,795413	1,07	0,000223	-1,76	0,444294	1,09	0,861156	1,02
11745397_a_at	57563	KLHL8	kelch-like family member 8	0,003424	-1,57	0,002799	-1,73	0,716735	1,10	0,073144	-1,31	0,01968	1,32	0,113177	1,20
11744726_s_at	55958	KLHL9	kelch-like family member 9	0,003372	-1,74	0,039712	-1,55	0,718524	-1,12	0,11329	-1,34	0,34437	1,15	0,059042	1,30
11739290_a_at	4297	KMT2A	lysine (K)-specific methyltransferase 2A	0,000487	-1,68	0,005022	-1,59	0,820931	-1,06	0,000202	-1,74	0,422294	-1,10	0,784416	-1,03
11758623_s_at	4297	KMT2A	lysine (K)-specific methyltransferase 2A	0,002456	-1,69	0,017398	-1,59	0,846549	-1,06	0,01075	-1,54	0,855148	1,03	0,51432	1,09
11739408_at	58508	KMT2C	lysine (K)-specific methyltransferase 2C	0,00964	-1,54	0,023803	-1,54	0,998511	1,00	0,007609	-1,57	0,903928	-1,02	0,902673	-1,02
11726357_a_at	55904	KMT2E	lysine (K)-specific methyltransferase 2E	0,0002	-2,35	0,001117	-2,33	0,981795	-1,01	2,61E-06	-3,11	0,078185	-1,34	0,074378	-1,32
11743115_a_at	3837	KPNB1	karyopherin (importin) beta 1	2,50E-05	-1,94	0,002137	-1,67	0,533118	-1,16	0,000115	-1,77	0,612426	-1,06	0,397354	1,10
11744839_a_at	3837	KPNB1	karyopherin (importin) beta 1	3,23E-05	-1,82	0,001788	-1,62	0,59545	-1,12	0,000239	-1,64	0,910492	-1,01	0,297829	1,11
11717963_a_at	3845	KRAS	Kirsten rat sarcoma viral oncogene homolog	0,000243	-2,12	0,003364	-1,94	0,793739	-1,09	0,022148	-1,54	0,121946	1,26	0,021052	1,38
11717965_s_at	3845	KRAS	Kirsten rat sarcoma viral oncogene homolog	0,001843	-1,66	0,019702	-1,52	0,764775	-1,09	0,002748	-1,62	0,648283	-1,06	0,875618	1,02
11726003_a_at	51315	KRCC1	lysine-rich coiled-coil 1	0,000425	-2,21	0,001537	-2,25	0,972629	1,02	0,001012	-2,05	0,599246	1,10	0,666029	1,08
11726002_s_at	51315	KRCC1	lysine-rich coiled-coil 1	0,014788	-1,77	0,026733	-1,82	0,958855	1,03	0,013741	-1,79	0,953876	1,01	0,951185	-1,01
11740855_a_at	889	KRIT1	KRIT1, ankyrin repeat containing	0,00011	-2,42	0,00128	-2,26	0,862973	-1,07	0,041515	-1,52	0,014666	1,48	0,002079	1,59
11730379_x_at	889	KRIT1	KRIT1, ankyrin repeat containing	7,73E-05	-2,37	0,002746	-2,04	0,661968	-1,16	0,001525	-1,90	0,683645	1,07	0,140421	1,24
11749630_a_at	11103	KRR1	KRR1, small subunit (SSU) processome component, homolog (yeast)	7,73E-05	-2,20	0,000781	-2,10	0,896694	-1,05	1,76E-06	-2,69	0,082353	-1,28	0,144707	-1,22
11753350_x_at	11103	KRR1	KRR1, small subunit (SSU) processome component, homolog (yeast)	6,87E-05	-2,05	0,001495	-1,88	0,757579	-1,09	2,78E-06	-2,38	0,060145	-1,27	0,229445	-1,16
11728682_at	11103	KRR1	KRR1, small subunit (SSU) processome component, homolog (yeast)	3,16E-05	-2,58	0,0016	-2,16	0,607643	-1,19	0,001052	-1,98	0,601817	1,09	0,074378	1,30
11728683_x_at	11103	KRR1	KRR1, small subunit (SSU) processome component, homolog (yeast)	0,000353	-1,95	0,010908	-1,67	0,602457	-1,17	3,52E-05	-2,20	0,041811	-1,32	0,380346	-1,13
11717070_x_at	3895	KTN1	kinectin 1 (kinesin receptor)	0,001456	-1,54	0,004969	-1,54	0,993191	1,00	2,10E-06	-2,03	0,007029	-1,32	0,003359	-1,32
11756394_a_at	3895	KTN1	kinectin 1 (kinesin receptor)	0,000354	-1,99	0,000939	-2,06	0,922226	1,04	0,000346	-1,96	0,754636	1,05	0,941598	1,01

11743491_x_at	3895	KTN1	kinectin 1 (kinesin receptor)	0,000361	-2,03	0,001398	-2,05	0,979295	1,01	0,000191	-2,08	0,930988	-1,01	0,8797	-1,03
11744926_x_at	3895	KTN1	kinectin 1 (kinesin receptor)	0,000687	-1,90	0,003384	-1,87	0,970493	-1,02	0,000161	-2,07	0,512779	-1,10	0,578274	-1,09
11747672_a_at	3895	KTN1	kinectin 1 (kinesin receptor)	0,001207	-1,84	0,003841	-1,87	0,97082	1,02	0,000762	-1,89	0,944303	-1,01	0,872306	-1,03
11732409_a_at	3895	KTN1	kinectin 1 (kinesin receptor)	0,001362	-1,88	0,007408	-1,81	0,929542	-1,04	0,000468	-2,01	0,507625	-1,11	0,667672	-1,07
11717072_x_at	3895	KTN1	kinectin 1 (kinesin receptor)	0,004923	-1,48	0,008383	-1,53	0,905803	1,03	0,000533	-1,66	0,498037	-1,08	0,302297	-1,12
11751616_a_at	51110	LACTB2	lactamase, beta 2	0,00062	-1,82	0,005356	-1,72	0,856769	-1,06	0,008211	-1,56	0,456644	1,11	0,211027	1,17
11742823_a_at	51110	LACTB2	lactamase, beta 2	0,00088	-1,80	0,007199	-1,70	0,855918	-1,06	0,013996	-1,51	0,404836	1,12	0,177739	1,19
11750202_a_at	3920	LAMP2	lysosomal-associated membrane protein 2	0,005997	-2,23	0,028217	-2,07	0,899387	-1,08	0,639862	1,16	0,000171	2,40	1,26E-05	2,58
11717478_at	3920	LAMP2	lysosomal-associated membrane protein 2	0,000927	-2,29	0,007266	-2,11	0,863212	-1,08	0,592438	-1,15	0,001184	1,84	8,43E-05	1,99
11717142_at	10314	LANCL1	LanC lantibiotic synthetase component C-like 1 (bacterial)	0,000615	-2,54	0,017477	-2,02	0,60355	-1,26	0,259618	1,34	3,04E-06	2,71	5,20E-09	3,41
11717143_at	10314	LANCL1	LanC lantibiotic synthetase component C-like 1 (bacterial)	0,002358	-1,97	0,029791	-1,71	0,716735	-1,15	0,031129	-1,60	0,703252	1,07	0,205871	1,23
11719615_s_at	113251	LARP4	La ribonucleoprotein domain family, member 4	0,001144	-1,98	0,007557	-1,88	0,894857	-1,05	0,01198	-1,66	0,469115	1,13	0,265815	1,19
11719614_a_at	113251	LARP4	La ribonucleoprotein domain family, member 4	0,003472	-1,96	0,020398	-1,83	0,873646	-1,07	0,103984	-1,45	0,192807	1,26	0,070172	1,36
11719694_a_at	51574	LARP7	La ribonucleoprotein domain family, member 7	0,00034	-1,67	0,008098	-1,50	0,656915	-1,11	1,08E-05	-1,92	0,018621	-1,27	0,158404	-1,15
11719696_x_at	51574	LARP7	La ribonucleoprotein domain family, member 7	0,000113	-1,71	0,002014	-1,60	0,779612	-1,07	6,64E-05	-1,72	0,483684	-1,08	0,937615	-1,01
11719695_a_at	51574	LARP7	La ribonucleoprotein domain family, member 7	0,0002	-1,71	0,002137	-1,64	0,870787	-1,04	0,000184	-1,70	0,758161	-1,04	0,963544	1,01
11723233_a_at	123169	LEO1	Leo1, Paf1/RNA polymerase II complex component, homolog (S. cerevisiae)	0,000151	-1,72	0,003834	-1,57	0,686768	-1,10	0,000429	-1,62	0,767143	-1,03	0,592236	1,06
11758955_at	3964	LGALS8	lectin, galactoside-binding, soluble, 8	0,013311	-1,42	0,008766	-1,55	0,734807	1,09	0,096638	-1,27	0,074112	1,22	0,303945	1,12
11730301_a_at	11019	LIAS	lipoic acid synthetase	0,000177	-2,17	0,00245	-2,00	0,81565	-1,08	0,015973	-1,58	0,111426	1,27	0,02208	1,37
11760127_at	3987 /// 100288695	LIMS1 /// LIMS3L	LIM and senescent cell antigen-like domains 1 /// LIM and senescent cell antigen-like d	0,001418	-1,83	0,004822	-1,84	0,990031	1,01	0,019314	-1,53	0,212366	1,20	0,20796	1,19
11760128_x_at	3987 /// 100288695	LIMS1 /// LIMS3L	LIM and senescent cell antigen-like domains 1 /// LIM and senescent cell antigen-like d	0,002872	-1,71	0,006795	-1,75	0,953469	1,02	0,040552	-1,43	0,153766	1,22	0,189702	1,19
11743740_x_at	55327	LIN7C	lin-7 homolog C (C. elegans)	0,001583	-1,89	0,020715	-1,68	0,727584	-1,13	0,01915	-1,58	0,727938	1,06	0,232694	1,20
11728874_a_at	286826	LIN9	lin-9 homolog (C. elegans)	0,001619	-1,88	0,018793	-1,68	0,75307	-1,11	0,73438	-1,07	0,003159	1,57	9,23E-05	1,75

11724213_at	3998	LMAN1	lectin, mannose-binding, 1	0,002255	-2,59	0,019245	-2,27	0,804448	-1,15	0,470171	1,26	2,44E-05	2,85	5,15E-07	3,27
11747181_a_at	3998	LMAN1	lectin, mannose-binding, 1	5,63E-05	-2,79	0,000738	-2,57	0,84707	-1,09	3,81E-05	-2,79	0,665979	-1,09	0,995534	-1,00
11724211_a_at	3998	LMAN1	lectin, mannose-binding, 1	0,000122	-2,32	0,001299	-2,19	0,877658	-1,06	5,47E-05	-2,40	0,578099	-1,10	0,859175	-1,03
11724212_at	3998	LMAN1	lectin, mannose-binding, 1	0,001675	-1,93	0,015784	-1,76	0,798038	-1,10	0,230093	-1,28	0,045047	1,37	0,005349	1,51
11753947_a_at	79809 /// 1005061 24	LOC1005061 24 /// TTC21B	uncharacterized LOC100506124 /// tetratricopeptide repeat domain 21B	0,000309	-1,69	0,00191	-1,66	0,957817	-1,02	0,000789	-1,60	0,753513	1,04	0,641475	1,05
11746383_a_at	7690 /// 1005066 39	LOC1005066 39 /// ZNF131	uncharacterized LOC100506639 /// zinc finger protein 131	0,000573	-1,80	0,003211	-1,77	0,960142	-1,02	0,007606	-1,55	0,292726	1,15	0,211603	1,17
11749644_s_at	6935 /// 1009966 68	LOC1009966 68 /// ZEB1	uncharacterized LOC100996668 /// zinc finger E-box binding homeobox 1	0,000132	-2,44	0,002622	-2,17	0,75183	-1,13	3,82E-08	-4,18	0,000123	-1,93	0,000538	-1,71
11758473_s_at	6935 /// 1009966 68	LOC1009966 68 /// ZEB1	uncharacterized LOC100996668 /// zinc finger E-box binding homeobox 1	0,000138	-2,05	0,00245	-1,87	0,769917	-1,09	8,97E-08	-3,01	0,000493	-1,61	0,001954	-1,47
11721545_x_at	6935 /// 1009966 68	LOC1009966 68 /// ZEB1	uncharacterized LOC100996668 /// zinc finger E-box binding homeobox 1	0,000196	-2,17	0,0019	-2,06	0,888767	-1,05	1,07E-07	-3,37	0,001105	-1,64	0,001523	-1,55
11734686_x_at	6935 /// 1009966 68	LOC1009966 68 /// ZEB1	uncharacterized LOC100996668 /// zinc finger E-box binding homeobox 1	0,000602	-1,96	0,004068	-1,88	0,917813	-1,04	4,77E-07	-2,96	0,001832	-1,58	0,001982	-1,52
11721547_s_at	6935 /// 1009966 68	LOC1009966 68 /// ZEB1	uncharacterized LOC100996668 /// zinc finger E-box binding homeobox 1	0,007249	-1,73	0,043493	-1,60	0,836483	-1,08	2,64E-05	-2,57	0,003051	-1,61	0,007423	-1,49
11756248_a_at	5170 /// 1010607 47	LOC1010607 47 /// PDPK1	3-phosphoinositide-dependent protein kinase 1-like /// 3-phosphoinositide dependent pro	0,016924	-1,52	0,027297	-1,57	0,941362	1,03	0,020205	-1,51	0,827398	1,04	0,970675	1,01
11759602_a_at	57035 /// 1019281 89	LOC1019281 89 /// RSRP1	uncharacterized LOC101928189 /// arginine/serine-rich protein 1	0,001548	-2,11	0,006877	-2,06	0,960913	-1,03	0,904694	1,03	4,61E-05	2,13	5,92E-06	2,18
11752738_a_at	55183 /// 1019293 36	LOC1019293 36 /// RIF1	uncharacterized LOC101929336 /// replication timing regulatory factor 1	0,001252	-1,91	0,024479	-1,65	0,655136	-1,16	0,144357	-1,33	0,160014	1,24	0,009667	1,44
11759068_at	55183 /// 1019293 36	LOC1019293 36 /// RIF1	uncharacterized LOC101929336 /// replication timing regulatory factor 1	0,001569	-2,08	0,018475	-1,84	0,751012	-1,13	0,002524	-2,00	0,647026	-1,09	0,85123	1,04

11731683_a_at	55183 /// 101929336	LOC101929336 /// RIF1	uncharacterized LOC101929336 /// replication timing regulatory factor 1	0,003511	-1,70	0,035038	-1,54	0,752589	-1,11	0,003455	-1,71	0,483125	-1,11	0,982896	-1,00
11731686_x_at	55183 /// 101929336	LOC101929336 /// RIF1	uncharacterized LOC101929336 /// replication timing regulatory factor 1	0,003257	-1,97	0,03765	-1,71	0,723614	-1,15	0,061343	-1,53	0,542648	1,12	0,134433	1,29
11739925_x_at	7587 /// 101929740	LOC101929740 /// ZNF37A	zinc finger protein 761-like /// zinc finger protein 37A	1,97E-05	-2,36	0,003047	-1,86	0,457902	-1,27	9,69E-09	-3,46	1,33E-05	-1,86	0,002525	-1,47
11739923_x_at	7587 /// 101929740	LOC101929740 /// ZNF37A	zinc finger protein 761-like /// zinc finger protein 37A	0,000149	-2,09	0,004746	-1,80	0,636911	-1,16	1,04E-06	-2,72	0,003223	-1,51	0,041694	-1,30
11734230_a_at	54816 /// 145783	LOC145783 /// ZNF280D	uncharacterized LOC145783 /// zinc finger protein 280D	0,013183	-1,76	0,029179	-1,77	0,989857	1,01	0,92545	-1,03	0,002512	1,72	0,00123	1,71
11730963_a_at	54816 /// 145783	LOC145783 /// ZNF280D	uncharacterized LOC145783 /// zinc finger protein 280D	0,004782	-1,85	0,02039	-1,77	0,928935	-1,04	0,010903	-1,74	0,918613	1,02	0,74662	1,06
11746122_s_at	9877 /// 441155	LOC441155 /// ZC3H11A	zinc finger CCCH-type domain-containing-like /// zinc finger CCCH-type containing 11A	0,006376	-2,21	0,038045	-1,99	0,844211	-1,11	0,322669	-1,34	0,083688	1,48	0,018401	1,65
11739793_a_at	55791	LRIF1	ligand dependent nuclear receptor interacting factor 1	0,002883	-1,71	0,041412	-1,51	0,683528	-1,13	0,038688	-1,44	0,764405	1,05	0,200939	1,19
11724318_a_at	55791	LRIF1	ligand dependent nuclear receptor interacting factor 1	0,003104	-1,72	0,045245	-1,51	0,677877	-1,14	0,019203	-1,53	0,947857	-1,01	0,402791	1,13
11721582_a_at	4033	LRMP	lymphoid-restricted membrane protein	0,017428	-1,38	0,009494	-1,51	0,704482	1,10	0,027714	-1,35	0,309803	1,12	0,868565	1,02
11757952_s_at	84918	LRP11	low density lipoprotein receptor-related protein 11	0,013638	-1,49	0,028646	-1,50	0,982494	1,01	0,124209	-1,29	0,22741	1,17	0,236153	1,16
11723248_at	55631	LRRC40	leucine rich repeat containing 40	0,00376	-1,77	0,031991	-1,61	0,786563	-1,10	0,00558	-1,72	0,672641	-1,07	0,891596	1,02
11742721_x_at	116064	LRRC58	leucine rich repeat containing 58	2,46E-05	-2,80	0,000557	-2,48	0,744532	-1,13	1,63E-06	-3,23	0,1128	-1,30	0,394999	-1,15
11742720_at	116064	LRRC58	leucine rich repeat containing 58	4,83E-05	-2,32	0,001689	-2,03	0,672211	-1,15	3,40E-06	-2,66	0,060186	-1,31	0,350632	-1,14
11742722_at	116064	LRRC58	leucine rich repeat containing 58	0,005469	-1,74	0,035823	-1,61	0,828214	-1,08	0,000198	-2,20	0,043565	-1,37	0,11679	-1,26
11745063_x_at	23507	LRRC8B	leucine rich repeat containing 8 family, member B	2,75E-05	-2,03	0,001809	-1,75	0,572659	-1,16	4,28E-06	-2,17	0,067605	-1,24	0,580663	-1,07
11741199_a_at	85444	LRRCC1	leucine rich repeat and coiled-coil centrosomal protein 1	0,005007	-2,12	0,040653	-1,86	0,784853	-1,14	0,000261	-2,80	0,048927	-1,50	0,167268	-1,32
11722304_a_at	9208	LRRFIP1	leucine rich repeat (in FLII) interacting protein 1	1,64E-05	-2,47	0,00016	-2,45	0,983062	-1,01	1,07E-06	-2,79	0,381874	-1,14	0,403989	-1,13
11739298_a_at	9208	LRRFIP1	leucine rich repeat (in FLII) interacting protein 1	0,000126	-2,06	0,000325	-2,16	0,88458	1,05	8,65E-06	-2,35	0,558028	-1,09	0,318531	-1,14

11722303_x_at	9208	LRRFIP1	leucine rich repeat (in FLII) interacting protein 1	0,000162	-1,91	0,000652	-1,93	0,973558	1,01	7,77E-06	-2,20	0,297003	-1,14	0,234588	-1,15
11744070_s_at	55341	LSG1	large 60S subunit nuclear export GTPase 1	0,000823	-1,59	0,006143	-1,53	0,87967	-1,04	0,000685	-1,60	0,699737	-1,04	0,973156	-1,00
11724926_at	26046	LTN1	listerin E3 ubiquitin protein ligase 1	0,002416	-1,76	0,014614	-1,67	0,886892	-1,05	0,000129	-2,12	0,098038	-1,27	0,182198	-1,20
11751835_a_at	84946	LTV1	LTV1 ribosome biogenesis factor	0,000679	-2,90	0,015375	-2,29	0,64671	-1,27	0,00024	-3,18	0,159325	-1,39	0,714369	-1,10
11746136_a_at	84946	LTV1	LTV1 ribosome biogenesis factor	0,000522	-2,34	0,008884	-2,02	0,715881	-1,16	0,000306	-2,41	0,339473	-1,19	0,88595	-1,03
11719872_a_at	84946	LTV1	LTV1 ribosome biogenesis factor	0,001145	-1,86	0,013748	-1,68	0,763439	-1,10	0,000693	-1,91	0,392586	-1,14	0,861939	-1,03
11739092_a_at	51747	LUC7L3	LUC7-like 3 (S. cerevisiae)	0,000256	-2,28	0,013334	-1,82	0,533209	-1,26	0,000688	-2,10	0,400488	-1,15	0,623525	1,09
11715778_a_at	136319 /// 767558	LUZP6 /// MTPN	leucine zipper protein 6 /// myotrophin	0,024678	-1,66	0,037908	-1,72	0,943036	1,04	0,205149	-1,34	0,167325	1,28	0,221361	1,24
11749360_s_at	10434	LYPLA1	lysophospholipase I	0,004428	-1,52	0,014979	-1,51	0,977112	-1,01	0,529568	1,10	2,11E-05	1,66	3,10E-06	1,67
11718365_s_at	10434	LYPLA1	lysophospholipase I	0,002369	-1,78	0,005167	-1,84	0,93192	1,03	0,90516	-1,03	8,81E-05	1,80	5,67E-05	1,74
11729686_x_at	90624	LYRM7	LYR motif containing 7	7,45E-05	-1,93	0,007925	-1,59	0,47292	-1,21	0,000342	-1,76	0,402114	-1,11	0,441035	1,10
11729685_at	90624	LYRM7	LYR motif containing 7	0,000147	-2,09	0,020691	-1,60	0,431149	-1,31	0,000477	-1,92	0,197784	-1,20	0,544556	1,09
11729688_s_at	90624	LYRM7	LYR motif containing 7	0,002038	-2,06	0,020724	-1,83	0,769634	-1,13	0,000153	-2,52	0,071854	-1,38	0,248425	-1,22
11729687_at	90624	LYRM7	LYR motif containing 7	0,004061	-1,71	0,011439	-1,72	0,990808	1,00	0,00686	-1,66	0,823683	1,04	0,850621	1,03
11758398_s_at	116068	LYSMD3	LysM, putative peptidoglycan-binding, domain containing 3	0,001192	-1,67	0,00832	-1,59	0,884061	-1,04	0,05171	-1,34	0,14738	1,19	0,052634	1,24
11743774_a_at	54585	LZTFL1	leucine zipper transcription factor-like 1	0,002847	-1,47	0,00295	-1,57	0,763439	1,07	0,004823	-1,44	0,377543	1,09	0,843128	1,02
11754437_a_at	23499	MACF1	microtubule-actin crosslinking factor 1	0,003315	-1,54	0,007733	-1,57	0,954012	1,02	7,28E-06	-2,09	0,012759	-1,33	0,004343	-1,35
11717899_x_at	84061	MAGT1	magnesium transporter 1	0,00016	-1,80	0,002238	-1,69	0,818002	-1,06	2,88E-07	-2,35	0,003121	-1,39	0,008817	-1,31
11755605_s_at	378938	MALAT1	metastasis associated lung adenocarcinoma transcript 1 (non-protein coding)	0,000555	-1,88	0,007984	-1,71	0,749859	-1,10	0,001799	-1,74	0,899868	-1,02	0,587424	1,08
11759177_at	4121	MAN1A1	mannosidase, alpha, class 1A, member 1	0,024538	-1,53	0,010005	-1,77	0,655817	1,16	0,000184	-2,15	0,205572	-1,21	0,014744	-1,41
11736102_at	4121	MAN1A1	mannosidase, alpha, class 1A, member 1	0,006227	-1,81	0,002164	-2,23	0,572117	1,23	0,0275	-1,61	0,051792	1,39	0,501049	1,12
11736101_at	4121	MAN1A1	mannosidase, alpha, class 1A, member 1	0,034084	-1,54	0,007754	-1,91	0,550535	1,24	0,213107	-1,30	0,017795	1,47	0,287294	1,19
11725471_a_at	4124	MAN2A1	mannosidase, alpha, class 2A, member 1	0,026084	-1,45	0,017844	-1,59	0,765811	1,09	0,464129	-1,14	0,012305	1,39	0,052991	1,27
11725564_at	79694	MANEA	mannosidase, endo-alpha	0,007282	-2,00	0,039401	-1,84	0,861291	-1,09	0,08573	-1,56	0,436106	1,18	0,201993	1,29
11743919_a_at	6885	MAP3K7	mitogen-activated protein kinase kinase kinase 7	0,00323	-1,46	0,005022	-1,52	0,871595	1,04	0,00415	-1,45	0,646692	1,05	0,936897	1,01

11742225_a_at	6885	MAP3K7	mitogen-activated protein kinase kinase kinase 7	0,007282	-1,61	0,016961	-1,63	0,979041	1,01	0,029579	-1,47	0,486372	1,11	0,528554	1,10
11750993_x_at	6885	MAP3K7	mitogen-activated protein kinase kinase kinase 7	0,039895	-1,43	0,038977	-1,52	0,859375	1,06	0,375861	-1,18	0,064831	1,29	0,142998	1,22
11724907_a_at	11183	MAP4K5	mitogen-activated protein kinase kinase kinase kinase 5	3,41E-05	-2,32	0,001299	-2,03	0,670364	-1,14	1,89E-06	-2,68	0,049521	-1,32	0,308678	-1,15
11724905_a_at	11183	MAP4K5	mitogen-activated protein kinase kinase kinase kinase 5	0,000452	-2,29	0,00597	-2,04	0,769917	-1,12	0,03747	-1,58	0,142528	1,29	0,022142	1,45
11724906_a_at	11183	MAP4K5	mitogen-activated protein kinase kinase kinase kinase 5	0,003658	-1,95	0,032416	-1,74	0,7778	-1,12	0,000776	-2,22	0,178855	-1,27	0,48899	-1,14
11759946_at	1432	MAPK14	mitogen-activated protein kinase 14	0,001177	-1,99	0,022346	-1,70	0,662649	-1,17	0,001473	-1,95	0,412677	-1,15	0,922757	1,02
11722796_a_at	5597	MAPK6	mitogen-activated protein kinase 6	0,023933	-1,67	0,033628	-1,74	0,925035	1,05	0,924662	1,03	0,001355	1,79	0,001317	1,71
11745507_a_at	64844	MARCH7	membrane-associated ring finger (C3HC4) 7, E3 ubiquitin protein ligase	0,005215	-2,21	0,020835	-2,11	0,937494	-1,05	0,521517	-1,21	0,011808	1,74	0,003309	1,83
11746613_a_at	64844	MARCH7	membrane-associated ring finger (C3HC4) 7, E3 ubiquitin protein ligase	0,004549	-1,97	0,016742	-1,92	0,960187	-1,03	0,313035	-1,28	0,027429	1,50	0,011989	1,54
11719890_a_at	64844	MARCH7	membrane-associated ring finger (C3HC4) 7, E3 ubiquitin protein ligase	0,00378	-1,76	0,02203	-1,66	0,872153	-1,06	0,020205	-1,57	0,735833	1,06	0,447296	1,12
11756390_a_at	9782 /// 724102	MATR3 /// SNHG4	matrin 3 /// small nucleolar RNA host gene 4 (non-protein coding)	0,001723	-2,27	0,005674	-2,28	0,992184	1,01	0,389421	-1,25	0,002692	1,82	0,001285	1,81
11743393_x_at	9782 /// 724102	MATR3 /// SNHG4	matrin 3 /// small nucleolar RNA host gene 4 (non-protein coding)	0,006977	-1,59	0,019252	-1,59	0,994388	-1,00	0,280915	-1,21	0,0422	1,31	0,02875	1,32
11743392_a_at	9782 /// 724102	MATR3 /// SNHG4	matrin 3 /// small nucleolar RNA host gene 4 (non-protein coding)	0,003496	-1,58	0,016993	-1,53	0,914412	-1,03	0,083677	-1,31	0,197814	1,17	0,096818	1,21
11749416_a_at	9782 /// 724102	MATR3 /// SNHG4	matrin 3 /// small nucleolar RNA host gene 4 (non-protein coding)	0,007805	-1,63	0,039401	-1,54	0,875568	-1,06	0,09316	-1,36	0,406269	1,13	0,196128	1,20
11755652_x_at	153364	MBLAC2	metallo-beta-lactamase domain containing 2	0,001839	-1,81	0,008123	-1,78	0,957642	-1,02	0,053116	-1,43	0,138808	1,24	0,085412	1,27
11756591_a_at	4154	MBNL1	muscleblind-like splicing regulator 1	0,003574	-2,95	0,013336	-2,85	0,967159	-1,04	0,937912	-1,03	0,000494	2,76	9,94E-05	2,86
11744794_a_at	4154	MBNL1	muscleblind-like splicing regulator 1	0,013834	-2,26	0,025101	-2,35	0,957642	1,04	0,955955	-1,02	0,001593	2,30	0,001089	2,21
11743661_a_at	4154	MBNL1	muscleblind-like splicing regulator 1	0,00045	-2,86	0,002844	-2,74	0,943334	-1,04	0,002383	-2,39	0,561249	1,15	0,421276	1,20
11722767_s_at	10150	MBNL2	muscleblind-like splicing regulator 2	0,002625	-1,73	0,032244	-1,54	0,719645	-1,12	0,003384	-1,70	0,509178	-1,10	0,931301	1,01
11730901_at	129642	MBOAT2	membrane bound O-acyltransferase domain containing 2	1,92E-06	-2,85	3,74E-05	-2,78	0,955552	-1,02	0,00033	-1,97	0,012786	1,42	0,004413	1,45
11751377_a_at	129642	MBOAT2	membrane bound O-acyltransferase domain containing 2	0,006065	-1,96	0,019373	-1,92	0,974325	-1,02	0,490717	1,19	2,85E-05	2,30	4,13E-06	2,34

11739930_a_at	84515	MCM8	minichromosome maintenance complex component 8	0,000175	-2,56	0,001528	-2,42	0,906215	-1,06	0,006216	-1,89	0,16725	1,28	0,073213	1,36
11734972_x_at	84515	MCM8	minichromosome maintenance complex component 8	0,00019	-2,26	0,003961	-1,99	0,717072	-1,14	0,006748	-1,73	0,393463	1,15	0,07386	1,31
11739931_x_at	84515	MCM8	minichromosome maintenance complex component 8	0,000452	-2,16	0,00733	-1,90	0,726493	-1,13	0,001165	-1,99	0,798139	-1,05	0,645471	1,08
11728399_at	29969	MDFIC	MyoD family inhibitor domain containing	0,093283	-1,34	0,028746	-1,55	0,623483	1,16	0,015448	-1,53	0,942592	1,01	0,315453	-1,15
11721009_at	5469	MED1	mediator complex subunit 1	8,97E-05	-1,77	0,005933	-1,53	0,531428	-1,16	1,13E-07	-2,30	0,000117	-1,50	0,006144	-1,30
11739633_s_at	9969	MED13	mediator complex subunit 13	0,00156	-1,61	0,003946	-1,65	0,937359	1,02	0,000392	-1,72	0,727163	-1,05	0,566571	-1,07
11724815_at	23389	MED13L	mediator complex subunit 13-like	0,008223	-1,56	0,019922	-1,57	0,990802	1,00	0,000595	-1,84	0,244193	-1,17	0,210717	-1,18
11760834_x_at	10001	MED6	mediator complex subunit 6	0,000376	-1,80	0,003559	-1,71	0,857646	-1,05	1,27E-07	-2,63	0,000431	-1,54	0,000736	-1,46
11718097_a_at	4205	MEF2A	myocyte enhancer factor 2A	0,089908	-1,36	0,014505	-1,68	0,511803	1,24	1,47E-05	-2,42	0,010425	-1,44	2,19E-05	-1,78
11723215_s_at	4208	MEF2C	myocyte enhancer factor 2C	0,015625	-1,51	0,027409	-1,54	0,954734	1,02	0,000755	-1,82	0,209781	-1,19	0,136701	-1,21
11723214_a_at	4208	MEF2C	myocyte enhancer factor 2C	0,024271	-1,47	0,022509	-1,58	0,834299	1,07	0,003196	-1,69	0,637681	-1,07	0,310811	-1,15
11723029_x_at	4232	MEST	mesoderm specific transcript	0,00855	-1,92	0,04122	-1,78	0,880591	-1,08	0,13211	-1,45	0,306754	1,23	0,137411	1,32
11758268_s_at	4232	MEST	mesoderm specific transcript	0,011732	-1,81	0,036429	-1,76	0,957425	-1,03	0,156105	-1,40	0,225556	1,26	0,152202	1,29
11716177_a_at	10988	METAP2	methionyl aminopeptidase 2	0,000395	-1,77	0,003364	-1,69	0,878346	-1,05	0,036732	-1,37	0,069532	1,24	0,018416	1,30
11755477_a_at	10988	METAP2	methionyl aminopeptidase 2	0,000754	-1,77	0,00456	-1,72	0,930112	-1,03	0,051633	-1,37	0,0688	1,26	0,028237	1,30
11742808_at	57721	METTL14	methyltransferase like 14	3,30E-05	-1,90	0,002666	-1,65	0,543036	-1,16	2,00E-06	-2,11	0,020721	-1,28	0,334545	-1,11
11729907_a_at	196074	METTL15	methyltransferase like 15	0,003786	-1,96	0,039829	-1,71	0,737942	-1,14	0,000207	-2,48	0,039674	-1,45	0,178687	-1,26
11734142_a_at	79828	METTL8	methyltransferase like 8	0,000312	-1,63	0,00405	-1,54	0,796333	-1,06	0,042495	-1,29	0,073336	1,19	0,010501	1,26
11721251_at	4236	MFAP1	microfibrillar-associated protein 1	0,001671	-1,76	0,020035	-1,59	0,743364	-1,10	0,130165	-1,30	0,143655	1,22	0,018177	1,35
11743706_a_at	23269	MGA	MGA, MAX dimerization protein	5,98E-05	-2,39	0,000877	-2,21	0,830443	-1,08	0,000121	-2,24	0,952354	-1,01	0,688699	1,07
11726638_at	57534	MIB1	mindbomb E3 ubiquitin protein ligase 1	0,000267	-2,45	0,007161	-2,04	0,64667	-1,20	0,030446	-1,63	0,213904	1,25	0,01451	1,50
11726637_at	57534	MIB1	mindbomb E3 ubiquitin protein ligase 1	0,001296	-1,78	0,009281	-1,69	0,872695	-1,06	0,000199	-1,99	0,236124	-1,18	0,427243	-1,12
11726641_at	57534	MIB1	mindbomb E3 ubiquitin protein ligase 1	0,001811	-1,99	0,009732	-1,91	0,917492	-1,05	0,000644	-2,15	0,491549	-1,13	0,674256	-1,08
11717683_s_at	221154	MICU2	mitochondrial calcium uptake 2	0,00026	-1,72	0,003979	-1,60	0,769895	-1,07	5,12E-05	-1,84	0,213733	-1,15	0,569353	-1,07
11729391_a_at	57708	MIER1	mesoderm induction early response 1, transcriptional regulator	0,001035	-1,62	0,006476	-1,56	0,906778	-1,03	0,141784	-1,23	0,02783	1,27	0,007199	1,32
11740072_s_at	57708	MIER1	mesoderm induction early response 1, transcriptional regulator	0,004641	-1,59	0,014357	-1,58	0,990761	-1,00	0,000298	-1,86	0,207001	-1,18	0,201394	-1,17
11725771_at	166968	MIER3	mesoderm induction early response 1, family member 3	0,011206	-1,55	0,037786	-1,51	0,943688	-1,03	0,45529	-1,15	0,041538	1,32	0,016909	1,35

11743355_x_at	27250 /// 100616113	MIR4680 /// PDCD4	microRNA 4680 /// programmed cell death 4 (neoplastic transformation inhibitor)	0,000112	-2,28	0,000951	-2,19	0,916612	-1,04	0,002064	-1,84	0,269764	1,19	0,146374	1,24
11741114_x_at	27250 /// 100616113	MIR4680 /// PDCD4	microRNA 4680 /// programmed cell death 4 (neoplastic transformation inhibitor)	0,003777	-1,61	0,017294	-1,56	0,924182	-1,03	0,001824	-1,68	0,572326	-1,08	0,753128	-1,05
11725145_a_at	79003	MIS12	MIS12 kinetochore complex component	0,001872	-1,83	0,024459	-1,62	0,719452	-1,13	0,001434	-1,86	0,374434	-1,15	0,924433	-1,02
11721143_a_at	4288	MKI67	marker of proliferation Ki-67	0,000688	-2,35	0,0057	-2,17	0,863099	-1,08	4,51E-05	-2,89	0,121903	-1,34	0,250587	-1,23
11715519_a_at	9761	MLEC	malectin	0,002671	-1,87	0,028445	-1,67	0,749881	-1,12	0,000372	-2,15	0,110662	-1,29	0,382889	-1,15
11754846_a_at	8028	MLLT10	myeloid/lymphoid or mixed-lineage leukemia (trithorax homolog, Drosophila); translocate	0,006027	-1,91	0,023223	-1,84	0,939554	-1,04	0,005261	-1,94	0,790228	-1,06	0,940102	-1,02
11748235_a_at	22915	MMRN1	multimerin 1	0,007678	-2,41	0,028544	-2,28	0,938277	-1,06	0,701029	-1,15	0,007918	1,98	0,002016	2,10
11758939_at	55233	MOB1A	MOB kinase activator 1A	0,002891	-2,06	0,008572	-2,08	0,990749	1,01	0,183538	-1,38	0,027064	1,51	0,020221	1,50
11719226_at	92597	MOB1B	MOB kinase activator 1B	0,005173	-1,91	0,013627	-1,92	0,987633	1,01	0,008712	-1,83	0,816874	1,05	0,852922	1,04
11751901_x_at	23041	MON2	MON2 homolog (S. cerevisiae)	0,003472	-1,68	0,011968	-1,67	0,98045	-1,01	0,146094	-1,29	0,06286	1,29	0,040032	1,30
11751900_a_at	23041	MON2	MON2 homolog (S. cerevisiae)	0,003948	-1,99	0,019702	-1,88	0,905928	-1,06	0,044091	-1,61	0,412662	1,17	0,236187	1,24
11723125_s_at	23515	MORC3	MORC family CW-type zinc finger 3	0,002671	-1,87	0,011272	-1,82	0,95325	-1,03	0,310755	-1,24	0,014944	1,48	0,005256	1,51
11743866_at	10199	MPHOSPH10	M-phase phosphoprotein 10 (U3 small nucleolar ribonucleoprotein)	0,00012	-1,82	0,002819	-1,66	0,717971	-1,09	9,80E-05	-1,81	0,46607	-1,09	0,973437	1,00
11727639_a_at	10198	MPHOSPH9	M-phase phosphoprotein 9	0,00172	-1,85	0,018969	-1,67	0,761801	-1,11	0,116966	-1,35	0,15349	1,24	0,022916	1,37
11730403_a_at	4361	MRE11A	MRE11 meiotic recombination 11 homolog A (S. cerevisiae)	0,000381	-2,09	0,002312	-2,04	0,957321	-1,02	0,005623	-1,72	0,263978	1,19	0,183164	1,21
11725792_a_at	4361	MRE11A	MRE11 meiotic recombination 11 homolog A (S. cerevisiae)	0,000683	-1,77	0,007884	-1,64	0,789112	-1,08	0,003963	-1,60	0,851686	1,03	0,4122	1,11
11725791_a_at	4361	MRE11A	MRE11 meiotic recombination 11 homolog A (S. cerevisiae)	0,010869	-1,81	0,010701	-2,00	0,814129	1,11	0,071208	-1,52	0,138808	1,31	0,346836	1,19
11743334_a_at	51318	MRPL35	mitochondrial ribosomal protein L35	0,000512	-1,55	0,003694	-1,51	0,910387	-1,03	0,201855	-1,17	0,005499	1,30	0,000937	1,33
11743805_s_at	28977	MRPL42	mitochondrial ribosomal protein L42	0,001394	-1,81	0,028603	-1,56	0,641196	-1,16	0,118999	-1,32	0,238041	1,18	0,016601	1,37
11722264_a_at	55173	MRPS10	mitochondrial ribosomal protein S10	2,85E-05	-2,28	0,000457	-2,13	0,83438	-1,07	1,68E-05	-2,30	0,61823	-1,08	0,973323	-1,01
11722263_at	55173	MRPS10	mitochondrial ribosomal protein S10	3,26E-05	-1,91	0,001995	-1,68	0,586975	-1,14	0,000127	-1,77	0,671125	-1,05	0,473844	1,08
11749237_a_at	23107	MRPS27	mitochondrial ribosomal protein S27	0,009626	-1,62	0,030195	-1,59	0,961653	-1,02	0,43934	1,16	5,09E-05	1,85	6,69E-06	1,88
11749490_x_at	4436	MSH2	mutS homolog 2	0,000612	-2,27	0,004283	-2,16	0,907614	-1,06	0,004786	-1,91	0,528347	1,13	0,329848	1,19
11744721_s_at	4436	MSH2	mutS homolog 2	0,001711	-2,01	0,012554	-1,86	0,85604	-1,08	0,005846	-1,82	0,916259	1,02	0,593801	1,10

11719416_s_at	4436	MSH2	mutS homolog 2	0,002023	-1,95	0,014943	-1,81	0,847103	-1,08	0,007261	-1,77	0,916904	1,02	0,577083	1,10
11731033_at	4437	MSH3	mutS homolog 3	0,000162	-2,03	0,005529	-1,75	0,618717	-1,16	1,78E-05	-2,26	0,054763	-1,29	0,419796	-1,12
11731032_at	4437	MSH3	mutS homolog 3	0,000788	-1,72	0,00426	-1,69	0,948246	-1,02	0,056106	-1,34	0,056879	1,26	0,026318	1,29
11749000_a_at	2956	MSH6	mutS homolog 6	0,005819	-2,29	0,033823	-2,06	0,853044	-1,11	0,032191	-1,89	0,746587	1,09	0,425751	1,21
11741168_a_at	253827	MSRB3	methionine sulfoxide reductase B3	0,000472	-2,07	0,020732	-1,68	0,534516	-1,24	0,000157	-2,20	0,073494	-1,31	0,714067	-1,06
11758515_x_at	92140	MTDH	metadherin	8,91E-06	-2,54	0,000492	-2,15	0,589291	-1,18	1,07E-06	-2,72	0,090054	-1,27	0,636623	-1,07
11722189_at	92140	MTDH	metadherin	0,0036	-1,62	0,027473	-1,51	0,81072	-1,07	0,000142	-1,94	0,045038	-1,29	0,132968	-1,20
11722188_at	92140	MTDH	metadherin	0,010031	-1,68	0,032423	-1,63	0,955216	-1,03	0,620203	-1,11	0,014643	1,47	0,005206	1,51
11723347_a_at	51001	MTERF3	mitochondrial transcription termination factor 3	0,001019	-1,71	0,00317	-1,74	0,959603	1,02	0,013443	-1,47	0,179277	1,18	0,213715	1,16
11726605_x_at	22823	MTF2	metal response element binding transcription factor 2	0,00016	-1,87	0,000456	-1,94	0,905928	1,04	3,78E-06	-2,22	0,266019	-1,14	0,133036	-1,19
11747742_a_at	22823	MTF2	metal response element binding transcription factor 2	0,000372	-1,95	0,001703	-1,95	0,994647	-1,00	3,16E-06	-2,53	0,055625	-1,30	0,044587	-1,29
11747743_x_at	22823	MTF2	metal response element binding transcription factor 2	0,000407	-1,65	0,000723	-1,73	0,845291	1,05	6,86E-06	-1,95	0,250137	-1,13	0,084773	-1,18
11726607_a_at	22823	MTF2	metal response element binding transcription factor 2	0,000631	-1,63	0,001439	-1,70	0,888767	1,04	4,46E-05	-1,83	0,483733	-1,08	0,267083	-1,12
11746584_x_at	22823	MTF2	metal response element binding transcription factor 2	0,003528	-1,53	0,006194	-1,58	0,90075	1,04	0,000174	-1,78	0,316795	-1,12	0,162904	-1,16
11726609_at	22823	MTF2	metal response element binding transcription factor 2	0,002947	-1,67	0,015144	-1,60	0,911129	-1,04	0,000205	-1,95	0,142166	-1,22	0,226138	-1,17
11726610_s_at	22823	MTF2	metal response element binding transcription factor 2	0,002929	-1,85	0,015786	-1,76	0,900533	-1,05	0,000311	-2,16	0,192176	-1,23	0,315031	-1,17
11726608_s_at	22823	MTF2	metal response element binding transcription factor 2	0,003853	-1,55	0,015597	-1,52	0,948154	-1,02	0,000521	-1,73	0,284551	-1,14	0,360873	-1,11
11720898_a_at	4534	MTM1	myotubularin 1	0,004787	-1,51	0,014446	-1,51	0,993901	-1,00	0,005419	-1,51	0,995898	1,00	0,981455	1,00
11730081_a_at	25821	MTO1	mitochondrial tRNA translation optimization 1	0,001488	-1,62	0,009774	-1,55	0,885658	-1,04	0,224588	-1,20	0,023356	1,30	0,004662	1,35
11739412_a_at	4548	MTR	5-methyltetrahydrofolate-homocysteine methyltransferase	0,001172	-1,86	0,015445	-1,67	0,73806	-1,11	0,006656	-1,65	0,960958	1,01	0,427496	1,12
11723653_at	4594	MUT	methylmalonyl CoA mutase	0,000585	-1,80	0,004169	-1,73	0,906915	-1,04	0,028731	-1,42	0,120243	1,22	0,048039	1,27
11727121_at	23077	MYCBP2	MYC binding protein 2, E3 ubiquitin protein ligase	0,002686	-1,57	0,006037	-1,61	0,940197	1,02	8,25E-06	-2,09	0,021749	-1,30	0,007279	-1,33
11736044_a_at	4628	MYH10	myosin, heavy chain 10, non-muscle	9,25E-05	-2,41	0,003273	-2,05	0,644572	-1,18	0,136433	-1,35	0,009451	1,51	0,000118	1,78
11735200_a_at	55728	N4BP2	NEDD4 binding protein 2	0,004725	-1,83	0,018349	-1,78	0,947934	-1,03	0,057565	-1,50	0,311104	1,19	0,210985	1,23
11719185_x_at	10443	N4BP2L2	NEDD4 binding protein 2-like 2	0,00741	-1,58	0,032423	-1,51	0,907614	-1,04	0,014043	-1,52	0,979354	-1,00	0,811606	1,04
11745181_a_at	80155	NAA15	N(alpha)-acetyltransferase 15, NatA auxiliary subunit	1,43E-05	-3,99	0,000311	-3,49	0,788695	-1,14	4,51E-05	-3,42	0,936474	1,02	0,488691	1,17

11745182_a_at	80155	NAA15	N(alpha)-acetyltransferase 15, NatA auxiliary subunit	0,003741	-1,92	0,028092	-1,74	0,812952	-1,10	0,849227	-1,05	0,003547	1,66	0,000204	1,83
11743709_at	80155	NAA15	N(alpha)-acetyltransferase 15, NatA auxiliary subunit	0,000128	-1,85	0,003406	-1,67	0,679975	-1,11	0,002081	-1,59	0,698107	1,05	0,1634	1,17
11745183_a_at	80155	NAA15	N(alpha)-acetyltransferase 15, NatA auxiliary subunit	0,000957	-1,93	0,006521	-1,83	0,889908	-1,05	0,020493	-1,56	0,279593	1,18	0,128967	1,24
11739508_a_at	122830	NAA30	N(alpha)-acetyltransferase 30, NatC catalytic subunit	2,50E-05	-1,75	0,000784	-1,62	0,688325	-1,08	0,021677	-1,31	0,019389	1,24	0,000531	1,34
11756128_a_at	80218	NAA50	N(alpha)-acetyltransferase 50, NatE catalytic subunit	0,001636	-2,11	0,010742	-1,98	0,88458	-1,07	0,828834	-1,06	0,000625	1,87	4,92E-05	2,00
11721799_s_at	80218	NAA50	N(alpha)-acetyltransferase 50, NatE catalytic subunit	0,000728	-1,70	0,017159	-1,50	0,636834	-1,13	0,00602	-1,51	0,964658	-1,01	0,317777	1,12
11733996_a_at	133686	NADK2	NAD kinase 2, mitochondrial	0,001787	-1,91	0,007161	-1,88	0,975918	-1,01	0,238891	-1,27	0,012414	1,48	0,005383	1,50
11749234_x_at	4673	NAP1L1	nucleosome assembly protein 1-like 1	0,000594	-1,40	0,000297	-1,52	0,593296	1,09	0,001029	-1,37	0,144171	1,11	0,79598	1,02
11749233_s_at	4673	NAP1L1	nucleosome assembly protein 1-like 1	0,020198	-1,49	0,010466	-1,68	0,697141	1,13	0,405563	-1,16	0,007053	1,45	0,052341	1,29
11752735_a_at	4677	NARS	asparaginyl-tRNA synthetase	0,0029	-1,82	0,017844	-1,71	0,871512	-1,06	0,249626	-1,26	0,046759	1,36	0,01037	1,45
11720396_x_at	4683	NBN	nibrin	0,001881	-1,69	0,013946	-1,59	0,849865	-1,06	0,217347	-1,23	0,041002	1,30	0,007263	1,38
11720397_a_at	4683	NBN	nibrin	0,000452	-2,10	0,006194	-1,89	0,761856	-1,11	0,007186	-1,71	0,553453	1,10	0,172687	1,23
11720395_a_at	4683	NBN	nibrin	0,001083	-1,85	0,008231	-1,75	0,865691	-1,06	0,053695	-1,42	0,139936	1,23	0,043628	1,31
11729219_at	4690	NCK1	NCK adaptor protein 1	0,000467	-1,76	0,001488	-1,80	0,947934	1,02	0,06692	-1,32	0,008761	1,37	0,008634	1,34
11721343_a_at	8648	NCOA1	nuclear receptor coactivator 1	0,002446	-1,78	0,015955	-1,67	0,866921	-1,06	0,003095	-1,75	0,78063	-1,05	0,936168	1,01
11718026_a_at	135112	NCOA7	nuclear receptor coactivator 7	0,009756	-2,04	0,032861	-1,96	0,947934	-1,04	0,798179	1,08	0,00061	2,12	0,0001	2,21
11754951_a_at	135112	NCOA7	nuclear receptor coactivator 7	0,018259	-1,54	0,044332	-1,53	0,987677	-1,01	0,108957	-1,34	0,394548	1,14	0,352419	1,14
11751590_a_at	9611	NCOR1	nuclear receptor corepressor 1	0,001785	-1,82	0,009602	-1,75	0,919031	-1,04	0,001142	-1,87	0,671904	-1,07	0,867027	-1,03
11740287_a_at	121441	NEDD1	neural precursor cell expressed, developmentally down-regulated 1	0,0014	-1,63	0,015491	-1,51	0,770131	-1,08	1,50E-05	-2,05	0,009495	-1,35	0,04002	-1,25
11739546_a_at	121441	NEDD1	neural precursor cell expressed, developmentally down-regulated 1	0,014813	-1,64	0,030038	-1,66	0,97894	1,01	0,919976	-1,02	0,002734	1,62	0,001576	1,60
11741727_a_at	121441	NEDD1	neural precursor cell expressed, developmentally down-regulated 1	0,018865	-1,84	0,040239	-1,85	0,991998	1,01	0,997602	-1,00	0,002994	1,85	0,001463	1,84
11739545_s_at	121441	NEDD1	neural precursor cell expressed, developmentally down-regulated 1	0,001751	-1,56	0,005033	-1,58	0,97179	1,01	0,015871	-1,39	0,267571	1,13	0,299466	1,12

11725574_a_at	4734	NEDD4	neural precursor cell expressed, developmentally down-regulated 4, E3 ubiquitin protein	0,001457	-2,16	0,011172	-1,99	0,853378	-1,09	0,000344	-2,43	0,296073	-1,22	0,551464	-1,12
11750554_a_at	4734	NEDD4	neural precursor cell expressed, developmentally down-regulated 4, E3 ubiquitin protein	0,000891	-1,96	0,006967	-1,84	0,863212	-1,07	0,006152	-1,71	0,652884	1,08	0,36383	1,15
11729396_a_at	4750	NEK1	NIMA-related kinase 1	0,00194	-1,78	0,016527	-1,65	0,813235	-1,08	0,001173	-1,84	0,463012	-1,12	0,849067	-1,03
11757406_s_at	4750	NEK1	NIMA-related kinase 1	0,005056	-1,64	0,037601	-1,51	0,802598	-1,08	0,000854	-1,83	0,163601	-1,21	0,413648	-1,12
11728264_at	6787	NEK4	NIMA-related kinase 4	0,002804	-1,50	0,004169	-1,57	0,850398	1,05	0,000502	-1,63	0,776611	-1,03	0,4486	-1,08
11750683_a_at	6787	NEK4	NIMA-related kinase 4	0,002749	-1,78	0,027852	-1,60	0,759819	-1,11	0,00447	-1,72	0,65663	-1,07	0,857391	1,03
11717452_x_at	140609	NEK7	NIMA-related kinase 7	0,000361	-1,75	0,001726	-1,75	0,989434	-1,00	0,00809	-1,48	0,14833	1,18	0,118856	1,19
11717451_at	140609	NEK7	NIMA-related kinase 7	0,001137	-1,74	0,005515	-1,71	0,955552	-1,02	0,011129	-1,52	0,381766	1,12	0,281882	1,15
11739242_x_at	9147	NEMF	nuclear export mediator factor	9,85E-06	-1,95	0,000393	-1,77	0,663048	-1,10	2,04E-07	-2,21	0,027516	-1,25	0,207508	-1,13
11739241_a_at	9147	NEMF	nuclear export mediator factor	6,14E-05	-1,93	0,003211	-1,68	0,589291	-1,15	1,66E-06	-2,25	0,011767	-1,34	0,170666	-1,17
11739243_a_at	9147	NEMF	nuclear export mediator factor	0,001817	-1,79	0,016961	-1,65	0,796008	-1,09	7,99E-05	-2,17	0,050468	-1,32	0,160159	-1,21
11753917_x_at	9147	NEMF	nuclear export mediator factor	0,002101	-1,79	0,030942	-1,57	0,692299	-1,14	0,000473	-1,97	0,12255	-1,25	0,522698	-1,10
11739057_a_at	4780	NFE2L2	nuclear factor, erythroid 2-like 2	0,00367	-1,65	0,02581	-1,54	0,829026	-1,07	0,031267	-1,44	0,641264	1,07	0,306394	1,14
11733067_at	4801	NFYB	nuclear transcription factor Y, beta	0,000517	-1,78	0,003223	-1,73	0,940894	-1,03	0,290374	-1,18	0,001888	1,47	0,00035	1,50
11733065_a_at	4801	NFYB	nuclear transcription factor Y, beta	0,000275	-1,60	0,003429	-1,52	0,809417	-1,05	0,000118	-1,65	0,426167	-1,08	0,811351	-1,03
11743377_a_at	51199	NIN	ninein (GSK3B interacting protein)	0,00449	-1,66	0,014053	-1,65	0,99039	-1,01	0,00023	-2,00	0,171918	-1,21	0,165541	-1,20
11725117_a_at	57486	NLN	neurolysin (metallopeptidase M3 family)	0,002673	-1,64	0,019842	-1,54	0,831207	-1,07	0,479289	-1,13	0,013594	1,37	0,001402	1,46
11751081_x_at	51068	NMD3	NMD3 ribosome export adaptor	0,005535	-2,59	0,016737	-2,55	0,987228	-1,01	0,525168	1,26	2,62E-05	3,21	4,52E-06	3,25
11746028_x_at	9111	NMI	N-myc (and STAT) interactor	0,000149	-1,60	0,001521	-1,55	0,882064	-1,03	0,000128	-1,59	0,774948	-1,03	0,966196	1,00
11746027_a_at	9111	NMI	N-myc (and STAT) interactor	0,00018	-1,80	0,005374	-1,60	0,642595	-1,12	0,000189	-1,78	0,38211	-1,11	0,918224	1,01
11718158_a_at	9111	NMI	N-myc (and STAT) interactor	0,000482	-1,60	0,004887	-1,52	0,828199	-1,05	0,038832	-1,30	0,10724	1,17	0,022888	1,23
11736431_a_at	55035	NOL8	nucleolar protein 8	2,46E-05	-3,27	0,000398	-2,94	0,81597	-1,11	0,004039	-2,04	0,055732	1,44	0,008415	1,60
11747090_a_at	51602	NOP58	NOP58 ribonucleoprotein	0,001063	-2,06	0,008468	-1,92	0,854454	-1,08	0,000203	-2,32	0,264085	-1,21	0,50055	-1,12
11750572_a_at	4863	NPAT	nuclear protein, ataxia-telangiectasia locus	0,000139	-1,81	0,005724	-1,58	0,587043	-1,14	0,010461	-1,44	0,411252	1,10	0,028189	1,26
11749197_a_at	4863	NPAT	nuclear protein, ataxia-telangiectasia locus	0,000407	-1,65	0,005889	-1,53	0,753461	-1,08	0,016948	-1,37	0,293505	1,12	0,058525	1,20

11743180_at	4863	NPAT	nuclear protein, ataxia-telangiectasia locus	0,002439	-1,76	0,010511	-1,72	0,952621	-1,02	0,008552	-1,62	0,713019	1,06	0,589111	1,08
11722087_a_at	9975	NR1D2	nuclear receptor subfamily 1, group D, member 2	0,018967	-2,05	0,032792	-2,13	0,956278	1,04	0,727101	-1,13	0,008719	1,89	0,007916	1,82
11735194_a_at	2908	NR3C1	nuclear receptor subfamily 3, group C, member 1 (glucocorticoid receptor)	0,003854	-1,76	0,00283	-2,01	0,698481	1,14	6,91E-05	-2,31	0,3774	-1,15	0,059282	-1,31
11741227_a_at	2908	NR3C1	nuclear receptor subfamily 3, group C, member 1 (glucocorticoid receptor)	0,003508	-1,71	0,002706	-1,93	0,702083	1,13	7,67E-05	-2,17	0,42181	-1,13	0,074378	-1,27
11742190_a_at	2908	NR3C1	nuclear receptor subfamily 3, group C, member 1 (glucocorticoid receptor)	0,005024	-1,68	0,0032	-1,91	0,680674	1,14	7,85E-05	-2,19	0,362195	-1,14	0,048573	-1,31
11736504_a_at	2908	NR3C1	nuclear receptor subfamily 3, group C, member 1 (glucocorticoid receptor)	0,004584	-1,70	0,003112	-1,93	0,690229	1,14	9,35E-05	-2,18	0,41328	-1,13	0,065654	-1,29
11742425_a_at	2908	NR3C1	nuclear receptor subfamily 3, group C, member 1 (glucocorticoid receptor)	0,008537	-1,66	0,005405	-1,88	0,712431	1,13	0,000127	-2,22	0,290876	-1,18	0,042351	-1,33
11743737_a_at	2908	NR3C1	nuclear receptor subfamily 3, group C, member 1 (glucocorticoid receptor)	0,005068	-1,61	0,003797	-1,79	0,718524	1,11	0,000144	-1,99	0,447286	-1,11	0,092652	-1,24
11720154_at	8204	NRIP1	nuclear receptor interacting protein 1	0,000177	-3,51	0,002057	-3,16	0,856905	-1,11	0,006547	-2,33	0,209155	1,36	0,0708	1,51
11720153_s_at	8204	NRIP1	nuclear receptor interacting protein 1	0,005643	-1,93	0,022838	-1,85	0,932241	-1,04	0,000151	-2,61	0,061693	-1,41	0,085506	-1,35
11724802_at	84081	NSRP1	nuclear speckle splicing regulatory protein 1	0,00237	-1,86	0,034818	-1,62	0,685752	-1,15	1,78E-05	-2,59	0,002776	-1,60	0,024028	-1,39
11722566_a_at	22978	NT5C2	5'-nucleotidase, cytosolic II	0,00426	-1,77	0,012526	-1,78	0,99851	1,00	0,225858	-1,28	0,032877	1,39	0,023175	1,39
11720204_at	221294	NT5DC1	5'-nucleotidase domain containing 1	0,000342	-1,66	0,005659	-1,54	0,729461	-1,08	1,46E-05	-1,89	0,04401	-1,23	0,205904	-1,14
11746359_a_at	4925	NUCB2	nucleobindin 2	0,001021	-1,64	0,007261	-1,57	0,883277	-1,04	0,000121	-1,82	0,210068	-1,15	0,37053	-1,11
11728648_a_at	64710	NUCKS1	nuclear casein kinase and cyclin-dependent kinase substrate 1	0,000256	-1,71	0,009204	-1,51	0,590363	-1,13	7,35E-06	-1,98	0,009711	-1,31	0,146616	-1,16
11738840_a_at	170685	NUDT10	nudix (nucleoside diphosphate linked moiety X)-type motif 10	0,06662	-1,42	0,026959	-1,64	0,676032	1,15	0,002432	-1,83	0,481097	-1,12	0,078338	-1,29
11756899_a_at	11051	NUDT21	nudix (nucleoside diphosphate linked moiety X)-type motif 21	0,001757	-1,99	0,013913	-1,84	0,8385	-1,08	0,004057	-1,87	0,929802	-1,02	0,729582	1,07
11718960_at	57532	NUFIP2	nuclear fragile X mental retardation protein interacting protein 2	0,000118	-2,06	0,000864	-2,00	0,941964	-1,03	0,038969	-1,42	0,009532	1,41	0,002656	1,45
11718965_s_at	57532	NUFIP2	nuclear fragile X mental retardation protein interacting protein 2	0,001419	-1,70	0,015243	-1,57	0,776952	-1,09	0,001076	-1,73	0,469319	-1,10	0,92565	-1,01
11718966_at	57532	NUFIP2	nuclear fragile X mental retardation protein interacting protein 2	0,004839	-1,62	0,027741	-1,54	0,865219	-1,06	0,033361	-1,44	0,647748	1,07	0,362774	1,13

11750468_a_at	129401	NUP35	nucleoporin 35kDa	0,002934	-1,60	0,017033	-1,53	0,885798	-1,05	0,027376	-1,41	0,51393	1,09	0,285788	1,14
11731490_a_at	348995	NUP43	nucleoporin 43kDa	0,008842	-1,87	0,022955	-1,87	0,995745	-1,00	0,982223	-1,01	0,001148	1,85	0,000398	1,86
11746714_a_at	348995	NUP43	nucleoporin 43kDa	0,012928	-1,62	0,037044	-1,59	0,969146	-1,02	0,967555	-1,01	0,00306	1,57	0,000888	1,60
11756835_a_at	53371	NUP54	nucleoporin 54kDa	0,001173	-1,99	0,011598	-1,82	0,802863	-1,10	0,020139	-1,61	0,466768	1,13	0,166242	1,24
11743662_a_at	53371	NUP54	nucleoporin 54kDa	0,001341	-1,99	0,019867	-1,74	0,709675	-1,15	0,00641	-1,77	0,923126	-1,02	0,483218	1,12
11743663_x_at	53371	NUP54	nucleoporin 54kDa	0,004423	-1,66	0,024682	-1,57	0,876608	-1,06	0,025711	-1,48	0,693133	1,06	0,41632	1,12
11747065_x_at	4927	NUP88	nucleoporin 88kDa	0,001598	-1,50	0,004909	-1,51	0,97894	1,01	0,024605	-1,32	0,173599	1,14	0,182448	1,13
11721510_x_at	116150	NUS1	nuclear undecaprenyl pyrophosphate synthase 1 homolog (S. cerevisiae)	0,009041	-1,54	0,016172	-1,58	0,939263	1,03	0,74272	-1,06	0,002277	1,49	0,002027	1,45
11736793_a_at	55916	NXT2	nuclear transport factor 2-like export factor 2	0,014502	-1,55	0,040167	-1,53	0,971292	-1,01	0,029545	-1,48	0,851247	1,03	0,776121	1,05
11727457_a_at	8481	OFD1	oral-facial-digital syndrome 1	0,000344	-1,68	0,004068	-1,59	0,807461	-1,06	4,34E-06	-2,02	0,021142	-1,27	0,066511	-1,20
11731585_a_at	115209	OMA1	OMA1 zinc metallopeptidase	0,002682	-1,64	0,005167	-1,71	0,910009	1,04	0,389647	-1,16	0,002282	1,48	0,002766	1,42
11757614_s_at	4976	OPA1	optic atrophy 1 (autosomal dominant)	0,001249	-2,14	0,033112	-1,74	0,594177	-1,23	0,000216	-2,45	0,053751	-1,41	0,461528	-1,14
11718488_a_at	23595	ORC3	origin recognition complex, subunit 3	0,001437	-1,89	0,015087	-1,72	0,782953	-1,10	0,006586	-1,70	0,953821	1,01	0,495351	1,11
11730783_a_at	5000	ORC4	origin recognition complex, subunit 4	0,001373	-1,63	0,015042	-1,51	0,775378	-1,08	0,999381	1,00	0,000424	1,51	9,69E-06	1,63
11718645_a_at	114882	OSBPL8	oxysterol binding protein-like 8	0,009819	-2,25	0,022481	-2,27	0,987226	1,01	0,192141	1,51	2,39E-06	3,44	5,12E-07	3,40
11718644_x_at	114882	OSBPL8	oxysterol binding protein-like 8	0,000997	-2,81	0,006033	-2,63	0,915092	-1,07	0,397793	-1,30	0,002845	2,03	0,000437	2,16
11755548_a_at	114882	OSBPL8	oxysterol binding protein-like 8	0,00354	-2,25	0,034702	-1,94	0,756421	-1,16	0,885393	-1,05	0,00428	1,85	0,000143	2,15
11718643_a_at	114882	OSBPL8	oxysterol binding protein-like 8	0,001977	-2,41	0,034842	-1,94	0,656914	-1,24	0,635175	-1,15	0,015313	1,69	0,000268	2,09
11748211_a_at	114883	OSBPL9	oxysterol binding protein-like 9	0,001048	-1,66	0,00764	-1,59	0,877754	-1,05	0,000661	-1,70	0,599517	-1,07	0,880825	-1,02
11726539_a_at	114883	OSBPL9	oxysterol binding protein-like 9	0,001241	-1,60	0,007553	-1,55	0,911129	-1,03	0,001119	-1,61	0,772485	-1,04	0,97823	-1,00
11723734_s_at	51633	OTUD6B	OTU domain containing 6B	0,001367	-1,90	0,02966	-1,62	0,631954	-1,17	0,036563	-1,50	0,640604	1,08	0,099792	1,27
11749874_a_at	5019	OXCT1	3-oxoacid CoA transferase 1	0,001076	-1,70	0,012121	-1,57	0,777562	-1,08	0,294073	-1,18	0,018843	1,33	0,001297	1,44
11745194_a_at	5019	OXCT1	3-oxoacid CoA transferase 1	0,000351	-1,86	0,007264	-1,66	0,683424	-1,12	0,011634	-1,51	0,462991	1,10	0,076632	1,24
11739772_s_at	55074	OXR1	oxidation resistance 1	0,005664	-1,86	0,015955	-1,86	0,997889	-1,00	0,999427	-1,00	0,000487	1,86	0,000152	1,86
11745780_x_at	55074	OXR1	oxidation resistance 1	0,001274	-1,87	0,003841	-1,90	0,963017	1,02	0,161237	-1,30	0,00916	1,46	0,007862	1,44
11739771_a_at	55074	OXR1	oxidation resistance 1	0,012585	-1,65	0,041655	-1,60	0,94227	-1,03	0,924179	1,02	0,00204	1,64	0,000392	1,69

11723341_x_at	55074	OXR1	oxidation resistance 1	0,011845	-1,44	0,014525	-1,51	0,868424	1,05	0,002275	-1,58	0,72223	-1,05	0,428913	-1,10
11749028_a_at	167153	PAPD4	PAP associated domain containing 4	0,006576	-2,00	0,026723	-1,91	0,926414	-1,05	0,502907	-1,20	0,019529	1,59	0,005504	1,67
11745873_x_at	10914	PAPOLA	poly(A) polymerase alpha	0,000487	-2,48	0,00323	-2,36	0,926881	-1,05	0,825712	-1,06	5,08E-05	2,22	4,29E-06	2,33
11748732_x_at	10914	PAPOLA	poly(A) polymerase alpha	0,00063	-2,89	0,003625	-2,77	0,948456	-1,04	0,850549	-1,06	5,47E-05	2,60	6,18E-06	2,71
11745872_a_at	10914	PAPOLA	poly(A) polymerase alpha	0,00186	-2,53	0,021759	-2,14	0,742062	-1,18	0,730931	1,12	0,000176	2,39	2,45E-06	2,83
11719009_a_at	10914	PAPOLA	poly(A) polymerase alpha	0,000441	-2,77	0,007819	-2,32	0,707661	-1,19	0,254275	-1,37	0,012636	1,70	0,00036	2,03
11744391_a_at	10914	PAPOLA	poly(A) polymerase alpha	0,000419	-1,96	0,016925	-1,63	0,549947	-1,20	0,005894	-1,64	0,965207	-1,01	0,19143	1,19
11745871_a_at	10914	PAPOLA	poly(A) polymerase alpha	0,004973	-1,53	0,009751	-1,57	0,936575	1,03	0,049005	-1,34	0,190093	1,17	0,260005	1,14
11726249_a_at	142	PARP1	poly (ADP-ribose) polymerase 1	0,000161	-2,50	0,002695	-2,24	0,78465	-1,12	0,010633	-1,77	0,179695	1,27	0,034379	1,41
11722679_a_at	79668	PARP8	poly (ADP-ribose) polymerase family, member 8	0,950727	-1,02	0,047109	-1,85	0,348052	1,81	0,714707	1,11	0,000777	2,06	0,562996	1,14
11748736_x_at	55010	PARPBP	PARP1 binding protein	0,002673	-2,13	0,033327	-1,82	0,716651	-1,17	0,632498	-1,14	0,014404	1,60	0,000478	1,87
11726186_x_at	55010	PARPBP	PARP1 binding protein	0,004471	-1,66	0,040587	-1,51	0,764775	-1,10	0,188305	-1,26	0,202156	1,20	0,035563	1,31
11719541_a_at	94104	PAXBP1	PAX3 and PAX7 binding protein 1	0,005202	-2,04	0,025155	-1,91	0,899125	-1,07	0,001943	-2,24	0,443979	-1,17	0,6588	-1,10
11719542_a_at	94104	PAXBP1	PAX3 and PAX7 binding protein 1	0,017754	-1,81	0,025984	-1,91	0,924142	1,05	0,094459	-1,53	0,274768	1,25	0,393625	1,19
11744007_s_at	55193	PBRM1	polybromo 1	4,42E-05	-2,23	0,000292	-2,22	0,992355	-1,00	0,00016	-2,03	0,524559	1,10	0,495871	1,10
11722295_at	51585	PCF11	PCF11 cleavage and polyadenylation factor subunit	0,001735	-1,85	0,004426	-1,91	0,945576	1,03	0,001259	-1,89	0,966556	1,01	0,908269	-1,02
11724554_s_at	84333	PCGF5	polycomb group ring finger 5	0,003413	-1,64	0,011618	-1,62	0,982602	-1,01	0,00016	-1,95	0,156403	-1,20	0,158342	-1,19
11746595_a_at	5108	PCM1	pericentriolar material 1	0,001019	-1,79	0,006767	-1,71	0,893879	-1,05	0,001156	-1,77	0,824361	-1,03	0,944517	1,01
11718735_at	115294	PCMTD1	protein-L-isoaspartate (D-aspartate) O-methyltransferase domain containing 1	0,011617	-1,60	0,024533	-1,62	0,979041	1,01	0,006165	-1,68	0,820415	-1,04	0,763909	-1,05
11749902_a_at	57092	PCNP	PEST proteolytic signal containing nuclear protein	0,008547	-1,64	0,024261	-1,62	0,982949	-1,01	0,435849	-1,16	0,023665	1,39	0,012733	1,41
11734957_a_at	64430	PCNXL4	pecanex-like 4 (Drosophila)	0,001908	-1,94	0,018088	-1,75	0,790009	-1,10	0,03889	-1,53	0,422962	1,14	0,131159	1,26
11756695_x_at	64430	PCNXL4	pecanex-like 4 (Drosophila)	0,002019	-1,88	0,023247	-1,68	0,741918	-1,12	0,045354	-1,49	0,465662	1,13	0,115782	1,26
11740371_s_at	8050	PDHX	pyruvate dehydrogenase complex, component X	0,000287	-1,70	0,007636	-1,53	0,646232	-1,11	0,000181	-1,72	0,268533	-1,13	0,927295	-1,01
11724953_a_at	149420	PDIK1L	PDLIM1 interacting kinase 1 like	0,001256	-1,85	0,020207	-1,63	0,696022	-1,13	0,002579	-1,76	0,615107	-1,08	0,767517	1,05
11717182_a_at	23244	PDS5A	PDS5, regulator of cohesion maintenance, homolog A (S. cerevisiae)	0,004774	-1,62	0,021596	-1,56	0,916133	-1,04	0,000147	-1,99	0,065137	-1,28	0,100671	-1,23

11737960_a_at	23244	PDS5A	PDS5, regulator of cohesion maintenance, homolog A (S. cerevisiae)	0,000779	-2,08	0,006663	-1,93	0,848713	-1,08	0,00876	-1,73	0,520136	1,12	0,245334	1,20
11717183_a_at	23244	PDS5A	PDS5, regulator of cohesion maintenance, homolog A (S. cerevisiae)	0,017103	-1,52	0,022151	-1,60	0,896157	1,05	0,090451	-1,35	0,243805	1,18	0,397974	1,13
11730905_a_at	23047	PDS5B	PDS5, regulator of cohesion maintenance, homolog B (S. cerevisiae)	0,005513	-1,62	0,010963	-1,66	0,940756	1,03	0,000132	-2,03	0,141659	-1,22	0,076443	-1,25
11752696_a_at	55276	PGM2	phosphoglucomutase 2	0,01219	-1,91	0,049065	-1,79	0,904202	-1,07	0,55083	1,18	0,000307	2,12	2,64E-05	2,25
11758556_s_at	55276	PGM2	phosphoglucomutase 2	0,00313	-1,72	0,037298	-1,54	0,719868	-1,12	0,022961	-1,51	0,910567	1,02	0,350067	1,14
11740688_a_at	9749	PHACTR2	phosphatase and actin regulator 2	0,00022	-1,82	0,005494	-1,63	0,670407	-1,12	0,216509	-1,21	0,009881	1,35	0,000169	1,51
11740993_a_at	51808	PHAX	phosphorylated adaptor for RNA export	0,00045	-1,83	0,011439	-1,61	0,635793	-1,14	0,000383	-1,83	0,311447	-1,14	0,992085	1,00
11753933_x_at	51808	PHAX	phosphorylated adaptor for RNA export	0,000369	-1,86	0,010631	-1,62	0,61534	-1,15	0,000673	-1,78	0,475375	-1,10	0,748751	1,05
11730377_a_at	9678	PHF14	PHD finger protein 14	5,98E-05	-1,86	0,000941	-1,75	0,807156	-1,06	1,06E-07	-2,41	0,003188	-1,38	0,009864	-1,30
11718681_a_at	9678	PHF14	PHD finger protein 14	0,000142	-2,18	0,004735	-1,87	0,629233	-1,17	0,068699	-1,40	0,050593	1,33	0,001163	1,56
11750127_a_at	9678	PHF14	PHD finger protein 14	0,000474	-1,92	0,004887	-1,79	0,82544	-1,07	0,181116	-1,27	0,011376	1,41	0,001053	1,52
11736333_at	51230	PHF20	PHD finger protein 20	0,000102	-1,64	0,000864	-1,60	0,921067	-1,02	0,000167	-1,59	0,950538	1,01	0,764502	1,03
11727530_a_at	51105	PHF20L1	PHD finger protein 20-like 1	5,98E-05	-1,67	4,81E-05	-1,85	0,609776	1,11	3,21E-05	-1,69	0,329486	1,09	0,920853	-1,01
11733292_a_at	51105	PHF20L1	PHD finger protein 20-like 1	5,98E-05	-2,10	0,000287	-2,13	0,963241	1,02	0,000965	-1,76	0,143722	1,21	0,167198	1,19
11742454_s_at	51105	PHF20L1	PHD finger protein 20-like 1	0,000432	-1,39	0,000248	-1,51	0,586975	1,09	0,000299	-1,40	0,267909	1,08	0,956314	-1,00
11727529_at	51105	PHF20L1	PHD finger protein 20-like 1	0,003139	-1,58	0,006346	-1,63	0,926017	1,03	0,002861	-1,59	0,868837	1,02	0,963544	-1,01
11727531_at	51105	PHF20L1	PHD finger protein 20-like 1	0,016964	-1,41	0,011548	-1,54	0,747925	1,09	0,027468	-1,38	0,370166	1,11	0,861537	1,02
11742826_s_at	23469	PHF3	PHD finger protein 3	0,000455	-2,22	0,006246	-1,98	0,761309	-1,12	0,000418	-2,21	0,535969	-1,12	0,978366	1,01
11742828_s_at	23469	PHF3	PHD finger protein 3	0,000427	-2,42	0,011618	-1,98	0,621439	-1,22	0,000936	-2,23	0,5342	-1,13	0,693611	1,08
11742830_a_at	23469	PHF3	PHD finger protein 3	0,000837	-1,94	0,00532	-1,86	0,911192	-1,04	0,000394	-2,03	0,58813	-1,09	0,796131	-1,04
11742829_x_at	23469	PHF3	PHD finger protein 3	0,00338	-1,97	0,047655	-1,67	0,68035	-1,18	0,005724	-1,89	0,5149	-1,13	0,84468	1,04
11720847_s_at	84295	PHF6	PHD finger protein 6	0,003125	-1,85	0,033645	-1,64	0,740203	-1,13	0,037414	-1,53	0,686011	1,07	0,224963	1,21
11720363_at	55023	PHIP	pleckstrin homology domain interacting protein	0,000238	-2,13	0,002415	-2,01	0,878346	-1,06	3,66E-05	-2,36	0,2867	-1,18	0,495351	-1,11
11720362_at	55023	PHIP	pleckstrin homology domain interacting protein	0,006254	-1,70	0,027199	-1,63	0,913189	-1,04	0,000123	-2,22	0,039494	-1,36	0,060728	-1,31
11719316_s_at	10464	PIBF1	progesterone immunomodulatory binding factor 1	0,002234	-1,74	0,004257	-1,82	0,896694	1,05	0,012173	-1,56	0,275702	1,17	0,442359	1,11

11719315_a_at	10464	PIBF1	progesterone immunomodulatory binding factor 1	0,004451	-1,80	0,020443	-1,72	0,916245	-1,05	0,123028	-1,37	0,158454	1,26	0,073801	1,31
11719317_x_at	10464	PIBF1	progesterone immunomodulatory binding factor 1	0,003106	-1,81	0,016894	-1,72	0,896694	-1,05	0,019988	-1,58	0,621158	1,09	0,391084	1,14
11721858_x_at	10026	PIGK	phosphatidylinositol glycan anchor biosynthesis, class K	0,006891	-1,57	0,009479	-1,65	0,874403	1,05	0,019796	-1,47	0,402221	1,12	0,659471	1,06
11748524_x_at	10026	PIGK	phosphatidylinositol glycan anchor biosynthesis, class K	0,006719	-1,56	0,014311	-1,59	0,958997	1,02	0,039361	-1,40	0,337784	1,14	0,404492	1,11
11749549_a_at	54965	PIGX	phosphatidylinositol glycan anchor biosynthesis, class X	6,35E-05	-1,96	0,00283	-1,72	0,621123	-1,14	9,52E-05	-1,89	0,453971	-1,10	0,786821	1,04
11745214_a_at	5290	PIK3CA	phosphatidylinositol-4,5-bisphosphate 3-kinase, catalytic subunit alpha	9,14E-05	-2,35	0,000443	-2,38	0,977744	1,01	0,005618	-1,74	0,042545	1,37	0,038851	1,35
11740212_x_at	5294	PIK3CG	phosphatidylinositol-4,5-bisphosphate 3-kinase, catalytic subunit gamma	0,00055	-2,01	0,002622	-2,00	0,992745	-1,00	0,000259	-2,09	0,792916	-1,05	0,812295	-1,04
11739539_a_at	5295	PIK3R1	phosphoinositide-3-kinase, regulatory subunit 1 (alpha)	0,006925	-1,87	0,049259	-1,68	0,798038	-1,11	4,89E-06	-3,31	0,000237	-1,97	0,000669	-1,77
11721029_a_at	8395	PIP5K1B	phosphatidylinositol-4-phosphate 5-kinase, type I, beta	0,005814	-1,64	0,030631	-1,55	0,877744	-1,06	0,967228	1,01	0,001459	1,56	0,000124	1,65
11719114_a_at	9867	PJA2	praja ring finger 2, E3 ubiquitin protein ligase	0,009027	-2,49	0,019177	-2,56	0,971834	1,03	0,550894	1,25	4,24E-05	3,20	1,46E-05	3,11
11719113_a_at	9867	PJA2	praja ring finger 2, E3 ubiquitin protein ligase	0,001015	-1,86	0,003328	-1,88	0,974635	1,01	0,253515	-1,23	0,002849	1,53	0,001743	1,51
11724841_a_at	5586	PKN2	protein kinase N2	0,00106	-1,86	0,015618	-1,66	0,717504	-1,12	3,04E-06	-2,62	0,001354	-1,58	0,008998	-1,41
11756270_x_at	8502	PKP4	plakophilin 4	0,000495	-2,05	0,017387	-1,70	0,572117	-1,21	0,017254	-1,59	0,686476	1,07	0,07499	1,29
11727754_a_at	5321	PLA2G4A	phospholipase A2, group IVA (cytosolic, calcium-dependent)	0,000904	-1,83	0,028137	-1,55	0,572407	-1,18	0,288603	-1,21	0,068392	1,28	0,000981	1,52
11757854_s_at	54477	PLEKHA5	pleckstrin homology domain containing, family A member 5	0,000649	-2,06	0,013142	-1,78	0,673099	-1,16	4,55E-05	-2,45	0,041318	-1,38	0,265368	-1,19
11757742_x_at	55041	PLEKHB2	pleckstrin homology domain containing, family B (evectins) member 2	0,000631	-1,99	0,007547	-1,81	0,786563	-1,10	0,020818	-1,55	0,31335	1,17	0,080592	1,28
11753027_a_at	55041	PLEKHB2	pleckstrin homology domain containing, family B (evectins) member 2	0,00146	-1,96	0,013925	-1,79	0,803157	-1,10	0,011716	-1,68	0,722708	1,06	0,331947	1,17
11747114_a_at	10733	PLK4	polo-like kinase 4	0,007786	-1,69	0,035695	-1,60	0,8947	-1,05	0,02922	-1,53	0,805084	1,04	0,556106	1,10
11721333_a_at	5352	PLOD2	procollagen-lysine, 2-oxoglutarate 5-dioxygenase 2	0,000504	-2,11	0,033659	-1,63	0,477274	-1,29	0,652776	-1,10	0,01324	1,48	1,65E-05	1,91
11759078_at	5356	PLRG1	pleiotropic regulator 1	0,000303	-1,91	0,002081	-1,85	0,939445	-1,03	0,000799	-1,78	0,784915	1,04	0,630208	1,07
11759079_s_at	5356	PLRG1	pleiotropic regulator 1	0,003398	-1,66	0,016991	-1,59	0,91027	-1,04	0,000414	-1,88	0,219265	-1,18	0,340202	-1,14
11741315_a_at	57088	PLSCR4	phospholipid scramblase 4	0,001032	-2,18	0,030069	-1,75	0,580959	-1,24	0,167329	-1,37	0,167902	1,28	0,005014	1,59

11736207_a_at	10154	PLXNC1	plexin C1	0,04045	-1,83	0,049396	-1,96	0,909898	1,07	0,146651	1,55	7,12E-06	3,03	4,40E-06	2,83
11748652_x_at	5378	PMS1	PMS1 postmeiotic segregation increased 1 (S. cerevisiae)	0,00029	-2,64	0,00149	-2,62	0,990106	-1,01	0,022304	-1,76	0,041407	1,49	0,026743	1,50
11748651_a_at	5378	PMS1	PMS1 postmeiotic segregation increased 1 (S. cerevisiae)	0,000419	-2,50	0,00323	-2,35	0,899447	-1,06	0,028123	-1,70	0,087931	1,38	0,03009	1,47
11763518_x_at	5378	PMS1	PMS1 postmeiotic segregation increased 1 (S. cerevisiae)	0,00096	-2,36	0,00307	-2,42	0,964518	1,03	0,048529	-1,63	0,041259	1,49	0,042561	1,45
11721699_a_at	5378	PMS1	PMS1 postmeiotic segregation increased 1 (S. cerevisiae)	0,00146	-1,82	0,006145	-1,79	0,972755	-1,01	0,006436	-1,65	0,586018	1,09	0,508736	1,10
11744502_a_at	25957	PNISR	PNN-interacting serine/arginine-rich protein	0,000238	-1,92	0,004169	-1,75	0,743045	-1,10	4,38E-07	-2,63	0,001522	-1,51	0,007996	-1,37
11746196_a_at	25957	PNISR	PNN-interacting serine/arginine-rich protein	3,26E-05	-2,72	0,000424	-2,56	0,884116	-1,06	1,23E-06	-3,31	0,128482	-1,29	0,237935	-1,21
11755634_x_at	25957	PNISR	PNN-interacting serine/arginine-rich protein	0,000348	-1,75	0,005509	-1,61	0,735975	-1,09	2,80E-06	-2,18	0,008028	-1,35	0,043867	-1,24
11754203_a_at	50640	PNPLA8	patatin-like phospholipase domain containing 8	0,00951	-1,62	0,008725	-1,77	0,794391	1,09	0,124764	-1,33	0,051626	1,33	0,165291	1,22
11738628_a_at	50640	PNPLA8	patatin-like phospholipase domain containing 8	0,017427	-1,55	0,015189	-1,69	0,802868	1,09	0,006424	-1,68	0,964296	1,01	0,62621	-1,08
11745750_a_at	50640	PNPLA8	patatin-like phospholipase domain containing 8	0,025058	-1,49	0,026959	-1,58	0,869265	1,06	0,016108	-1,55	0,902766	1,02	0,817652	-1,04
11734726_x_at	87178	PNPT1	polyribonucleotide nucleotidyltransferase 1	8,97E-05	-2,00	0,005405	-1,69	0,541194	-1,18	0,000647	-1,77	0,736128	-1,05	0,325935	1,13
11734725_a_at	87178	PNPT1	polyribonucleotide nucleotidyltransferase 1	0,005091	-1,67	0,035307	-1,55	0,818182	-1,08	0,242777	-1,24	0,121051	1,25	0,025313	1,35
11716989_s_at	55629	PNRC2	proline-rich nuclear receptor coactivator 2	0,002628	-1,73	0,018979	-1,61	0,837969	-1,07	0,252819	-1,23	0,049959	1,31	0,00862	1,40
11758304_s_at	55629	PNRC2	proline-rich nuclear receptor coactivator 2	0,002012	-2,74	0,01701	-2,39	0,813365	-1,15	0,177519	-1,54	0,075909	1,56	0,012651	1,78
11755428_a_at	51426	POLK	polymerase (DNA directed) kappa	0,013539	-1,56	0,02915	-1,57	0,985561	1,01	0,652654	1,09	0,000197	1,72	6,88E-05	1,70
11757974_s_at	51426	POLK	polymerase (DNA directed) kappa	0,000122	-2,81	0,002632	-2,43	0,735941	-1,16	3,69E-05	-3,02	0,267209	-1,24	0,733557	-1,07
11737479_a_at	5445	PON2	paraoxonase 2	0,000141	-2,11	0,003308	-1,88	0,703758	-1,13	0,000276	-1,99	0,691652	-1,06	0,704112	1,06
11741354_a_at	5445	PON2	paraoxonase 2	0,000478	-1,77	0,004911	-1,66	0,824219	-1,06	0,002248	-1,62	0,83662	1,03	0,459361	1,09
11737480_x_at	5445	PON2	paraoxonase 2	0,000911	-1,85	0,013172	-1,66	0,728262	-1,11	0,001556	-1,78	0,641406	-1,07	0,808855	1,04
11733079_a_at	25913	POT1	protection of telomeres 1	0,002383	-1,68	0,012747	-1,61	0,911129	-1,04	0,110133	-1,30	0,101286	1,24	0,039765	1,29
11731899_s_at	5471	PPAT	phosphoribosyl pyrophosphate amidotransferase	0,001772	-2,01	0,005376	-2,03	0,98045	1,01	0,016253	-1,68	0,279167	1,21	0,296818	1,19
11742882_at	5481	PPID	peptidylprolyl isomerase D	4,59E-05	-2,17	0,000501	-2,07	0,889938	-1,05	0,309449	-1,19	5,38E-05	1,74	3,02E-06	1,82

11754211_a_at	9360	PPIG	peptidylprolyl isomerase G (cyclophilin G)	6,82E-06	-1,79	0,000204	-1,69	0,759398	-1,06	5,12E-10	-2,40	6,49E-05	-1,42	0,00025	-1,34
11754212_a_at	9360	PPIG	peptidylprolyl isomerase G (cyclophilin G)	1,12E-05	-1,95	0,000328	-1,80	0,714509	-1,09	4,83E-08	-2,37	0,006516	-1,32	0,042783	-1,21
11754213_x_at	9360	PPIG	peptidylprolyl isomerase G (cyclophilin G)	1,16E-05	-1,94	0,000257	-1,84	0,821171	-1,06	4,96E-08	-2,36	0,013422	-1,29	0,038725	-1,22
11746095_a_at	9360	PPIG	peptidylprolyl isomerase G (cyclophilin G)	4,17E-05	-1,79	0,000811	-1,68	0,780106	-1,07	1,14E-07	-2,22	0,004923	-1,32	0,019487	-1,24
11754214_a_at	9360	PPIG	peptidylprolyl isomerase G (cyclophilin G)	7,32E-05	-2,09	0,002081	-1,87	0,701376	-1,12	4,65E-06	-2,39	0,061036	-1,28	0,307293	-1,14
11730267_a_at	9360	PPIG	peptidylprolyl isomerase G (cyclophilin G)	0,001101	-1,69	0,014795	-1,54	0,736924	-1,09	0,000248	-1,82	0,172716	-1,18	0,550999	-1,08
11726148_a_at	23262	PPIP5K2	diphosphoinositol pentakisphosphate kinase 2	0,005329	-2,47	0,029886	-2,22	0,865219	-1,11	0,928186	1,03	0,001106	2,30	7,93E-05	2,56
11726149_a_at	23262	PPIP5K2	diphosphoinositol pentakisphosphate kinase 2	0,001349	-2,00	0,007422	-1,92	0,927292	-1,04	0,005712	-1,79	0,701945	1,07	0,522387	1,11
11726151_x_at	23262	PPIP5K2	diphosphoinositol pentakisphosphate kinase 2	0,002874	-1,81	0,0138	-1,74	0,926935	-1,04	0,00385	-1,78	0,91586	-1,02	0,920839	1,02
11726150_s_at	23262	PPIP5K2	diphosphoinositol pentakisphosphate kinase 2	0,005826	-1,66	0,033554	-1,55	0,85568	-1,06	0,003328	-1,73	0,489482	-1,11	0,799777	-1,04
11719713_a_at	5495	PPM1B	protein phosphatase, Mg2+/Mn2+ dependent, 1B	0,001412	-1,85	0,020118	-1,64	0,714509	-1,13	0,007733	-1,65	0,977598	-1,00	0,440618	1,12
11725615_a_at	5500	PPP1CB	protein phosphatase 1, catalytic subunit, beta isozyme	0,013526	-2,10	0,04016	-2,04	0,961122	-1,03	0,032161	1,91	1,69E-07	3,90	1,35E-08	4,03
11733076_x_at	4659	PPP1R12A	protein phosphatase 1, regulatory subunit 12A	4,20E-05	-1,92	0,000204	-1,97	0,928211	1,03	0,000458	-1,68	0,159325	1,17	0,229097	1,14
11733077_a_at	4659	PPP1R12A	protein phosphatase 1, regulatory subunit 12A	0,000305	-1,62	0,000459	-1,72	0,79918	1,06	6,82E-06	-1,88	0,367248	-1,09	0,113429	-1,16
11733075_a_at	4659	PPP1R12A	protein phosphatase 1, regulatory subunit 12A	5,98E-05	-2,00	0,000238	-2,07	0,905878	1,04	0,000637	-1,74	0,143156	1,19	0,234971	1,15
11733078_a_at	4659	PPP1R12A	protein phosphatase 1, regulatory subunit 12A	0,004725	-1,95	0,010696	-2,01	0,958434	1,03	0,525762	-1,17	0,003536	1,71	0,00273	1,67
11733074_s_at	4659	PPP1R12A	protein phosphatase 1, regulatory subunit 12A	0,001657	-1,78	0,008572	-1,73	0,932197	-1,03	0,002123	-1,76	0,921064	-1,02	0,925665	1,02
11748297_s_at	5529	PPP2R5E	protein phosphatase 2, regulatory subunit B', epsilon isoform	0,001067	-1,58	0,004006	-1,59	0,995183	1,00	0,141381	-1,22	0,012412	1,30	0,007662	1,30
11730272_a_at	5529	PPP2R5E	protein phosphatase 2, regulatory subunit B', epsilon isoform	0,001719	-1,60	0,012135	-1,53	0,864475	-1,05	0,372354	-1,14	0,011086	1,34	0,001472	1,40
11748296_a_at	5529	PPP2R5E	protein phosphatase 2, regulatory subunit B', epsilon isoform	0,001322	-1,69	0,018838	-1,53	0,717072	-1,10	0,160267	-1,25	0,10114	1,22	0,008418	1,35
11736432_x_at	151987	PPP4R2	protein phosphatase 4, regulatory subunit 2	0,007864	-1,79	0,04233	-1,66	0,858177	-1,08	0,741314	-1,08	0,012477	1,53	0,001617	1,65
11759561_s_at	151987	PPP4R2	protein phosphatase 4, regulatory subunit 2	0,00053	-2,09	0,016527	-1,74	0,593296	-1,20	0,006641	-1,73	0,98891	1,00	0,222145	1,21

11746892_a_at	23398	PPWD1	peptidylprolyl isomerase domain and WD repeat containing 1	0,000655	-1,79	0,006199	-1,68	0,833565	-1,07	0,001433	-1,70	0,927969	-1,01	0,721702	1,05
11750421_a_at	23398	PPWD1	peptidylprolyl isomerase domain and WD repeat containing 1	0,001023	-1,65	0,008363	-1,57	0,850653	-1,05	0,00186	-1,60	0,891693	-1,02	0,79515	1,03
11744565_s_at	23398	PPWD1	peptidylprolyl isomerase domain and WD repeat containing 1	0,003967	-1,79	0,044876	-1,58	0,721165	-1,13	0,002468	-1,86	0,311629	-1,18	0,834759	-1,04
11744564_a_at	23398	PPWD1	peptidylprolyl isomerase domain and WD repeat containing 1	0,008905	-1,67	0,037522	-1,59	0,907614	-1,05	0,007542	-1,69	0,707673	-1,07	0,926988	-1,02
11755434_a_at	5567	PRKACB	protein kinase, cAMP-dependent, catalytic, beta	0,003671	-2,45	0,012121	-2,42	0,98663	-1,01	0,831635	1,08	9,29E-05	2,60	1,93E-05	2,64
11716703_a_at	5567	PRKACB	protein kinase, cAMP-dependent, catalytic, beta	0,003044	-1,60	0,011207	-1,58	0,97452	-1,01	0,085964	-1,30	0,117125	1,21	0,079547	1,22
11726772_a_at	5584	PRKCI	protein kinase C, iota	0,000124	-2,96	0,001521	-2,70	0,846549	-1,10	0,004511	-2,10	0,219552	1,29	0,0708	1,41
11726773_a_at	5584	PRKCI	protein kinase C, iota	0,005186	-1,66	0,020009	-1,61	0,945428	-1,03	0,000556	-1,91	0,23074	-1,19	0,299684	-1,16
11739583_s_at	23683	PRKD3	protein kinase D3	0,004677	-1,62	0,018253	-1,58	0,947823	-1,02	0,08651	-1,34	0,207103	1,18	0,128198	1,21
11719391_a_at	5591	PRKDC	protein kinase, DNA-activated, catalytic polypeptide	0,00123	-2,75	0,016798	-2,29	0,731426	-1,20	0,006424	-2,29	0,994715	-1,00	0,455798	1,20
11735731_a_at	5612	PRKRIR	protein-kinase, interferon-inducible double stranded RNA dependent inhibitor, repressor	0,006649	-1,63	0,009937	-1,71	0,890542	1,05	0,551269	-1,12	0,00272	1,52	0,004074	1,45
11737782_a_at	11212	PROSC	proline synthetase co-transcribed homolog (bacterial)	0,013111	-1,61	0,041295	-1,56	0,949522	-1,03	0,196603	-1,28	0,195502	1,22	0,121561	1,25
11730803_a_at	55119	PRPF38B	pre-mRNA processing factor 38B	0,00531	-1,49	0,008379	-1,54	0,891876	1,04	0,057019	-1,31	0,134209	1,18	0,236424	1,14
11730804_s_at	55119	PRPF38B	pre-mRNA processing factor 38B	0,002883	-1,61	0,009339	-1,61	0,994718	-1,00	0,015129	-1,47	0,478678	1,10	0,453868	1,10
11743385_a_at	55660	PRPF40A	PRP40 pre-mRNA processing factor 40 homolog A (S. cerevisiae)	0,00019	-1,71	0,003946	-1,57	0,719241	-1,09	2,87E-06	-2,02	0,015093	-1,28	0,088693	-1,18
11743382_a_at	55660	PRPF40A	PRP40 pre-mRNA processing factor 40 homolog A (S. cerevisiae)	2,14E-05	-2,17	0,000376	-2,03	0,813803	-1,07	3,45E-05	-2,06	0,918368	-1,02	0,695535	1,05
11743384_at	55660	PRPF40A	PRP40 pre-mRNA processing factor 40 homolog A (S. cerevisiae)	0,00639	-1,79	0,038783	-1,65	0,840731	-1,08	0,000216	-2,31	0,043108	-1,40	0,107752	-1,29
11743381_at	55660	PRPF40A	PRP40 pre-mRNA processing factor 40 homolog A (S. cerevisiae)	0,002037	-1,78	0,013335	-1,68	0,875568	-1,06	0,009436	-1,61	0,7809	1,05	0,492773	1,11
11718233_a_at	8899	PRPF4B	pre-mRNA processing factor 4B	0,000294	-2,90	0,002717	-2,69	0,889035	-1,08	0,160885	-1,47	0,00474	1,83	0,000616	1,97
11718236_s_at	8899	PRPF4B	pre-mRNA processing factor 4B	0,002372	-1,83	0,047421	-1,55	0,623077	-1,18	0,025136	-1,55	0,978948	1,00	0,256143	1,19
11756290_a_at	133619	PRRC1	proline-rich coiled-coil 1	0,000376	-1,75	0,006373	-1,60	0,719645	-1,10	0,044532	-1,34	0,129032	1,19	0,012443	1,31
11716566_a_at	23215	PRRC2C	proline-rich coiled-coil 2C	0,000732	-2,91	0,00841	-2,52	0,788378	-1,16	0,08808	-1,67	0,079856	1,51	0,010866	1,75
11745956_a_at	23215	PRRC2C	proline-rich coiled-coil 2C	0,013104	-1,56	0,046672	-1,50	0,926838	-1,04	0,001286	-1,82	0,180344	-1,21	0,260959	-1,17

11742933_a_at	11168	PSIP1	PC4 and SFRS1 interacting protein 1	0,000495	-2,29	0,003134	-2,21	0,941964	-1,04	0,001275	-2,11	0,804827	1,05	0,655897	1,09
11753142_a_at	5717	PSMD11	proteasome (prosome, macropain) 26S subunit, non-ATPase, 11	0,003384	-1,69	0,04056	-1,51	0,715375	-1,12	0,013039	-1,55	0,86181	-1,03	0,556371	1,09
11758266_s_at	5708	PSMD2	proteasome (prosome, macropain) 26S subunit, non-ATPase, 2	0,001142	-1,81	0,035216	-1,53	0,569752	-1,19	0,002271	-1,73	0,367927	-1,14	0,772601	1,05
11743102_at	55037	PTCD3	pentatricopeptide repeat domain 3	0,00029	-1,87	0,004068	-1,73	0,786228	-1,08	0,089424	-1,31	0,027084	1,32	0,002316	1,43
11743104_at	55037	PTCD3	pentatricopeptide repeat domain 3	0,006245	-1,59	0,025321	-1,54	0,927899	-1,03	0,009335	-1,56	0,944438	-1,01	0,894001	1,02
11719324_x_at	5728	PTEN	phosphatase and tensin homolog	0,006215	-1,46	0,011008	-1,51	0,922226	1,03	1,65E-06	-2,15	0,00123	-1,43	0,000168	-1,47
11719323_at	5728	PTEN	phosphatase and tensin homolog	0,004229	-1,79	0,024171	-1,68	0,873352	-1,06	8,56E-06	-2,74	0,002117	-1,63	0,003645	-1,53
11746800_s_at	51495	PTPLAD1	protein tyrosine phosphatase-like A domain containing 1	0,000573	-1,73	0,009005	-1,58	0,729889	-1,10	0,069391	-1,31	0,116954	1,20	0,011645	1,32
11746799_a_at	51495	PTPLAD1	protein tyrosine phosphatase-like A domain containing 1	0,000376	-1,87	0,010054	-1,63	0,630782	-1,15	0,016305	-1,48	0,481817	1,10	0,05541	1,26
11715864_a_at	51495	PTPLAD1	protein tyrosine phosphatase-like A domain containing 1	0,001959	-1,73	0,027324	-1,55	0,705006	-1,12	0,072637	-1,36	0,367905	1,13	0,059476	1,27
11729206_at	5781	PTPN11	protein tyrosine phosphatase, non-receptor type 11	0,0007	-1,74	0,005374	-1,66	0,880591	-1,05	0,002089	-1,63	0,89937	1,02	0,620375	1,07
11718805_a_at	23369	PUM2	pumilio RNA-binding family member 2	0,008701	-1,52	0,027066	-1,50	0,966677	-1,02	0,821017	1,04	0,000475	1,56	9,44E-05	1,59
11748996_a_at	23369	PUM2	pumilio RNA-binding family member 2	0,005419	-1,64	0,022567	-1,59	0,927921	-1,03	0,668981	-1,09	0,006214	1,46	0,001334	1,51
11752622_a_at	54899	PXK	PX domain containing serine/threonine kinase	0,016196	-1,73	0,049648	-1,68	0,948422	-1,03	0,230843	-1,32	0,194896	1,27	0,119842	1,31
11751040_a_at	22931	RAB18	RAB18, member RAS oncogene family	0,006758	-1,62	0,022484	-1,59	0,964087	-1,02	0,751156	1,06	0,000214	1,69	3,62E-05	1,72
11753494_x_at	22931	RAB18	RAB18, member RAS oncogene family	0,000993	-1,67	0,004716	-1,64	0,964884	-1,01	0,011866	-1,45	0,310898	1,13	0,233083	1,14
11725426_a_at	5873	RAB27A	RAB27A, member RAS oncogene family	0,027023	-1,57	0,009732	-1,87	0,635793	1,19	0,338377	-1,23	0,009791	1,52	0,109983	1,28
11725425_s_at	5873	RAB27A	RAB27A, member RAS oncogene family	0,016759	-1,48	0,004779	-1,74	0,572797	1,17	0,021146	-1,47	0,193363	1,19	0,951612	1,01
11742071_x_at	5873	RAB27A	RAB27A, member RAS oncogene family	0,038684	-1,40	0,012919	-1,61	0,625164	1,15	0,002541	-1,67	0,807825	-1,04	0,160589	-1,19
11725424_a_at	5873	RAB27A	RAB27A, member RAS oncogene family	0,026203	-1,56	0,012443	-1,81	0,686946	1,15	0,028631	-1,56	0,383281	1,16	0,995724	1,00
11753390_x_at	7879	RAB7A	RAB7A, member RAS oncogene family	0,000536	-1,58	0,005456	-1,50	0,824849	-1,05	0,000186	-1,64	0,373365	-1,09	0,713594	-1,04
11718614_s_at	9135	RABEP1	rabaptin, RAB GTPase binding effector protein 1	0,001252	-1,69	0,005761	-1,66	0,964087	-1,02	0,124948	-1,27	0,028515	1,31	0,012939	1,33
11750906_at	5810	RAD1	RAD1 homolog (S. pombe)	5,98E-05	-2,23	0,01873	-1,62	0,413308	-1,38	0,000107	-2,11	0,057899	-1,30	0,727512	1,06
11739778_a_at	5884	RAD17	RAD17 homolog (S. pombe)	0,000651	-1,85	0,003273	-1,82	0,972293	-1,01	0,0359	-1,43	0,0678	1,28	0,040458	1,29

11720838_a_at	5884	RAD17	RAD17 homolog (S. pombe)	0,00163	-1,73	0,011618	-1,64	0,864484	-1,06	0,013419	-1,52	0,602617	1,08	0,325463	1,14
11716436_s_at	5885	RAD21	RAD21 homolog (S. pombe)	1,16E-05	-2,95	0,000204	-2,78	0,887247	-1,06	0,011395	-1,72	0,003965	1,62	0,000482	1,72
11716437_a_at	5885	RAD21	RAD21 homolog (S. pombe)	3,34E-05	-3,30	0,00032	-3,17	0,943578	-1,04	0,019694	-1,82	0,005265	1,75	0,001277	1,82
11716438_at	5885	RAD21	RAD21 homolog (S. pombe)	5,98E-05	-3,46	0,000588	-3,24	0,906786	-1,07	0,022291	-1,88	0,012094	1,72	0,002481	1,84
11754276_a_at	5887	RAD23B	RAD23 homolog B (S. cerevisiae)	4,97E-05	-1,77	0,000864	-1,67	0,798649	-1,06	0,000886	-1,54	0,445159	1,08	0,151772	1,15
11730282_a_at	10111	RAD50	RAD50 homolog (S. cerevisiae)	2,50E-05	-2,69	0,000637	-2,37	0,731369	-1,13	7,67E-05	-2,41	0,937888	-1,01	0,512719	1,12
11730281_a_at	10111	RAD50	RAD50 homolog (S. cerevisiae)	0,001126	-1,68	0,005639	-1,64	0,949971	-1,02	0,010574	-1,48	0,401601	1,11	0,292343	1,13
11747886_a_at	10635	RAD51AP1	RAD51 associated protein 1	0,001101	-1,74	0,013423	-1,59	0,760705	-1,09	0,019702	-1,46	0,528897	1,09	0,159178	1,19
11722815_at	5903	RANBP2	RAN binding protein 2	0,001839	-1,82	0,035038	-1,57	0,643432	-1,16	6,78E-05	-2,25	0,013931	-1,43	0,137405	-1,23
11754283_s_at	5903	RANBP2	RAN binding protein 2	0,001144	-1,87	0,019848	-1,64	0,681494	-1,14	0,001436	-1,83	0,448327	-1,12	0,921549	1,02
11758005_s_at	5903	RANBP2	RAN binding protein 2	0,001439	-1,80	0,024667	-1,58	0,674601	-1,14	0,005061	-1,66	0,751665	-1,05	0,583551	1,08
11722816_at	5903	RANBP2	RAN binding protein 2	0,002371	-1,73	0,025063	-1,57	0,754979	-1,10	0,006423	-1,63	0,824641	-1,04	0,675889	1,06
11722814_s_at	5903 /// 84220 /// 285190 /// 400966 /// 653489 /// 727851 /// 729540 /// 729857	RANBP2 /// RGPD1 /// RGPD2 /// RGPD3 /// RGPD4 /// RGPD5 /// RGPD6 /// RGPD8	RAN binding protein 2 /// RANBP2-like and GRIP domain containing 1 /// RANBP2-like and	0,002023	-2,69	0,019331	-2,31	0,786228	-1,17	0,068469	-1,76	0,27924	1,31	0,067231	1,53
11720369_at	26953	RANBP6	RAN binding protein 6	0,004069	-1,74	0,040373	-1,56	0,749656	-1,11	7,08E-05	-2,27	0,011683	-1,45	0,056806	-1,31
11753623_s_at	5908	RAP1B	RAP1B, member of RAS oncogene family	0,039724	-1,75	0,049494	-1,86	0,912502	1,06	0,84375	-1,06	0,009859	1,75	0,013812	1,64
11719402_a_at	5911	RAP2A	RAP2A, member of RAS oncogene family	0,017137	-1,44	0,017033	-1,52	0,834875	1,06	0,007316	-1,52	0,970553	1,01	0,677446	-1,06
11758868_at	51735	RAPGEF6	Rap guanine nucleotide exchange factor (GEF) 6	0,001951	-1,81	0,010038	-1,74	0,925899	-1,04	0,000545	-1,96	0,436317	-1,13	0,593784	-1,09
11716999_a_at	5921	RASA1	RAS p21 protein activator (GTPase activating protein) 1	0,001643	-1,76	0,007386	-1,72	0,958573	-1,02	0,007873	-1,60	0,59354	1,08	0,483089	1,10
11747139_a_at	5921	RASA1	RAS p21 protein activator (GTPase activating protein) 1	0,009237	-1,87	0,025054	-1,85	0,987863	-1,01	0,377999	-1,25	0,034313	1,49	0,020947	1,50

11717702_a_at	5925	RB1	retinoblastoma 1	0,005749	-1,79	0,030912	-1,68	0,872371	-1,07	0,001265	-2,01	0,275397	-1,20	0,486797	-1,12
11743480_s_at	9821	RB1CC1	RB1-inducible coiled-coil 1	0,011438	-1,53	0,022827	-1,55	0,968876	1,02	0,000681	-1,81	0,234718	-1,17	0,172182	-1,19
11743481_at	9821	RB1CC1	RB1-inducible coiled-coil 1	0,008679	-1,62	0,006718	-1,80	0,755363	1,11	0,049123	-1,44	0,123051	1,25	0,404859	1,13
11755842_a_at	5930	RBBP6	retinoblastoma binding protein 6	0,000176	-1,75	0,000652	-1,77	0,964057	1,01	5,71E-07	-2,21	0,035772	-1,25	0,017099	-1,27
11743328_a_at	5930	RBBP6	retinoblastoma binding protein 6	0,000272	-1,65	0,001099	-1,66	0,979643	1,01	9,11E-07	-2,06	0,02895	-1,24	0,015701	-1,25
11727547_s_at	5932	RBBP8	retinoblastoma binding protein 8	0,002299	-1,72	0,010091	-1,68	0,948965	-1,02	0,000796	-1,83	0,542232	-1,09	0,660149	-1,07
11732560_s_at	389677	RBM12B	RNA binding motif protein 12B	0,00055	-2,08	0,010497	-1,81	0,691754	-1,15	0,01132	-1,66	0,61665	1,09	0,138049	1,25
11721678_a_at	84991	RBM17	RNA binding motif protein 17	0,000124	-2,11	0,00189	-1,95	0,804043	-1,08	2,39E-05	-2,29	0,265814	-1,17	0,593377	-1,08
11743408_x_at	58517	RBM25	RNA binding motif protein 25	4,09E-06	-2,50	0,000257	-2,17	0,616751	-1,15	5,45E-09	-3,37	0,000764	-1,56	0,013233	-1,35
11743407_a_at	58517	RBM25	RNA binding motif protein 25	1,28E-05	-2,13	0,000638	-1,87	0,621854	-1,14	4,56E-08	-2,70	0,001885	-1,44	0,028757	-1,27
11743410_a_at	58517	RBM25	RNA binding motif protein 25	6,23E-06	-2,81	0,000172	-2,56	0,785777	-1,10	1,94E-06	-2,85	0,491728	-1,12	0,936593	-1,01
11743411_a_at	58517	RBM25	RNA binding motif protein 25	0,000993	-1,89	0,010253	-1,74	0,797278	-1,09	0,026318	-1,51	0,344809	1,15	0,101129	1,25
11732327_a_at	64062	RBM26	RNA binding motif protein 26	0,000361	-1,87	0,002819	-1,80	0,908702	-1,04	2,72E-05	-2,13	0,197569	-1,18	0,311759	-1,14
11732328_a_at	64062	RBM26	RNA binding motif protein 26	0,002087	-1,80	0,024609	-1,61	0,736602	-1,12	0,113728	-1,34	0,213884	1,20	0,031551	1,34
11754499_a_at	10179	RBM7	RNA binding motif protein 7	0,003014	-1,64	0,026743	-1,52	0,784924	-1,08	0,9449	-1,01	0,001921	1,50	6,80E-05	1,62
11758378_s_at	494115	RBMXL1	RNA binding motif protein, X-linked-like 1	0,004032	-1,70	0,037704	-1,54	0,763467	-1,10	0,224942	-1,25	0,146169	1,23	0,021612	1,36
11739392_a_at	3516	RBPJ	recombination signal binding protein for immunoglobulin kappa J region	9,54E-05	-1,91	0,001114	-1,82	0,865219	-1,05	0,000433	-1,75	0,748332	1,04	0,447365	1,10
11729124_x_at	5955	RCN2	reticulocalbin 2, EF-hand calcium binding domain	0,003344	-1,54	0,010818	-1,54	0,99135	-1,00	0,040382	-1,35	0,252405	1,14	0,220384	1,15
11729123_a_at	5955	RCN2	reticulocalbin 2, EF-hand calcium binding domain	0,004162	-1,69	0,021632	-1,61	0,893435	-1,05	0,009573	-1,60	0,985078	1,00	0,743836	1,05
11717524_s_at	5962	RDX	radixin	0,001757	-1,62	0,00844	-1,59	0,943189	-1,02	0,158617	1,24	1,64E-07	1,96	1,03E-08	2,01
11717526_a_at	5962	RDX	radixin	4,64E-05	-1,75	0,000274	-1,76	0,978452	1,01	2,64E-05	-1,76	0,995388	1,00	0,952924	-1,01
11717525_s_at	5962	RDX	radixin	0,001009	-1,92	0,004068	-1,92	0,993976	-1,00	0,020662	-1,55	0,161703	1,23	0,136396	1,24
11739728_a_at	5965	RECQL	RecQ helicase-like	0,006364	-1,73	0,013357	-1,77	0,954734	1,03	0,743765	-1,07	0,001554	1,65	0,001094	1,61
11739729_a_at	5965	RECQL	RecQ helicase-like	0,011013	-1,59	0,045583	-1,52	0,901419	-1,05	0,000123	-2,13	0,0178	-1,40	0,028739	-1,34
11750695_x_at	5965	RECQL	RecQ helicase-like	0,000975	-1,72	0,005975	-1,66	0,912043	-1,04	0,075835	-1,32	0,061803	1,26	0,021298	1,30
11750694_a_at	5965	RECQL	RecQ helicase-like	0,000495	-1,61	0,005064	-1,53	0,824692	-1,05	0,001643	-1,52	0,949212	1,01	0,570685	1,06
11743006_x_at	5965	RECQL	RecQ helicase-like	0,001045	-1,85	0,011348	-1,69	0,786964	-1,09	0,00095	-1,85	0,549009	-1,09	0,990552	-1,00

11750334_a_at	5966	REL	v-rel avian reticuloendotheliosis viral oncogene homolog	0,009185	-1,64	0,024557	-1,64	0,990406	-1,01	0,001007	-1,92	0,291585	-1,18	0,292762	-1,17
11722899_a_at	51455	REV1	REV1, polymerase (DNA directed)	8,60E-05	-1,76	0,001481	-1,66	0,794391	-1,06	6,56E-06	-1,94	0,122088	-1,17	0,341189	-1,10
11740540_a_at	5980	REV3L	REV3-like, polymerase (DNA directed), zeta, catalytic subunit	0,00985	-1,68	0,043493	-1,59	0,891787	-1,06	0,001548	-1,93	0,226362	-1,21	0,379942	-1,15
11718894_a_at	5981	RFC1	replication factor C (activator 1) 1, 145kDa	0,004421	-1,63	0,023103	-1,56	0,890024	-1,05	3,02E-06	-2,48	0,00061	-1,59	0,000781	-1,52
11718893_at	5981	RFC1	replication factor C (activator 1) 1, 145kDa	0,001076	-1,82	0,007984	-1,72	0,872371	-1,06	0,000601	-1,88	0,553523	-1,09	0,840944	-1,03
11758440_s_at	64864	RFX7	regulatory factor X, 7	5,66E-05	-2,47	0,001521	-2,17	0,713945	-1,14	0,013186	-1,65	0,079152	1,32	0,005495	1,50
11721955_at	64864	RFX7	regulatory factor X, 7	0,000647	-2,45	0,012593	-2,05	0,680674	-1,20	0,318662	-1,29	0,016913	1,59	0,000406	1,90
11754028_s_at	6009	RHEB	Ras homolog enriched in brain	0,000469	-1,72	0,002471	-1,71	0,974635	-1,01	3,42E-05	-1,94	0,2664	-1,14	0,293213	-1,13
11716456_at	22836	RHOBTB3	Rho-related BTB domain containing 3	6,51E-05	-2,31	0,002676	-1,98	0,64096	-1,16	9,31E-05	-2,22	0,474512	-1,12	0,80352	1,04
11716457_x_at	22836	RHOBTB3	Rho-related BTB domain containing 3	8,36E-05	-2,47	0,002714	-2,12	0,674014	-1,16	9,63E-05	-2,39	0,479601	-1,13	0,871571	1,03
11758664_s_at	22836	RHOBTB3	Rho-related BTB domain containing 3	0,000573	-1,82	0,014854	-1,59	0,629198	-1,15	0,032562	-1,42	0,404814	1,12	0,039389	1,28
11746384_s_at	253260	RICTOR	RPTOR independent companion of MTOR, complex 2	0,006153	-2,08	0,015318	-2,11	0,984781	1,01	0,006151	-2,09	0,982912	1,01	0,981031	-1,01
11733924_a_at	83732	RIOK1	RIO kinase 1	0,000162	-2,71	0,002729	-2,40	0,780243	-1,13	4,93E-05	-2,92	0,313069	-1,22	0,718155	-1,08
11733925_a_at	83732	RIOK1	RIO kinase 1	0,001234	-1,67	0,012788	-1,55	0,793056	-1,08	0,005226	-1,54	0,96025	1,01	0,522419	1,08
11723454_at	55781	RIOK2	RIO kinase 2	0,00218	-1,81	0,029262	-1,60	0,710195	-1,13	0,018578	-1,56	0,885865	1,03	0,314356	1,16
11725951_at	8780	RIOK3	RIO kinase 3	0,052874	-1,48	0,036473	-1,64	0,787001	1,11	0,309708	1,24	2,60E-05	2,03	6,88E-05	1,84
11745203_a_at	80010	RMI1	RecQ mediated genome instability 1	0,013464	-1,84	0,033655	-1,82	0,990508	-1,01	0,617045	1,14	0,000213	2,08	5,27E-05	2,10
11723840_s_at	80010	RMI1	RecQ mediated genome instability 1	0,003348	-2,37	0,024671	-2,10	0,821023	-1,13	0,012844	-2,06	0,942645	1,02	0,558029	1,15
11745202_a_at	80010	RMI1	RecQ mediated genome instability 1	0,006025	-2,15	0,024368	-2,04	0,930177	-1,05	0,089698	-1,60	0,270428	1,28	0,159113	1,34
11755631_x_at	55005	RMND1	required for meiotic nuclear division 1 homolog (S. cerevisiae)	0,002042	-1,63	0,015026	-1,54	0,846549	-1,06	0,103666	-1,28	0,133775	1,20	0,035709	1,27
11723231_s_at	51444 /// 379013	RNF138 /// RNF138P1	ring finger protein 138, E3 ubiquitin protein ligase /// ring finger protein 138, E3 ub	0,000573	-1,65	0,004131	-1,60	0,906915	-1,03	0,138043	-1,23	0,01374	1,30	0,002917	1,34
11756637_a_at	79596	RNF219	ring finger protein 219	0,001528	-1,95	0,019051	-1,73	0,737741	-1,13	0,013648	-1,66	0,811932	1,04	0,304176	1,18
11720198_s_at	6049	RNF6	ring finger protein (C3H2C3 type) 6	0,000395	-1,75	0,002081	-1,73	0,975045	-1,01	0,00014	-1,83	0,680618	-1,05	0,74662	-1,04
11746183_a_at	6049	RNF6	ring finger protein (C3H2C3 type) 6	0,005757	-1,65	0,016178	-1,65	0,99781	-1,00	0,344495	-1,19	0,021333	1,38	0,013286	1,39
11721328_a_at	8732	RNGTT	RNA guanylyltransferase and 5'-phosphatase	0,006372	-1,76	0,025849	-1,70	0,927108	-1,04	0,116946	-1,38	0,218915	1,22	0,119458	1,27

11739450_x_at	6093	ROCK1	Rho-associated, coiled-coil containing protein kinase 1	0,000665	-1,83	0,003364	-1,80	0,96761	-1,02	0,000452	-1,86	0,831525	-1,03	0,91808	-1,02
11739448_s_at	6093 /// 727758	ROCK1 /// ROCK1P1	Rho-associated, coiled-coil containing protein kinase 1 /// Rho-associated, coiled-coil	0,008448	-1,76	0,02587	-1,72	0,969414	-1,02	0,758175	-1,08	0,005006	1,60	0,001636	1,63
11761875_x_at	727758	ROCK1P1	Rho-associated, coiled-coil containing protein kinase 1 pseudogene 1	0,000835	-1,80	0,007211	-1,69	0,846235	-1,06	0,000684	-1,81	0,634178	-1,07	0,968718	-1,01
11739385_x_at	9475	ROCK2	Rho-associated, coiled-coil containing protein kinase 2	3,08E-05	-2,80	0,000577	-2,52	0,789112	-1,11	1,35E-06	-3,35	0,092453	-1,33	0,280368	-1,20
11724595_at	6102	RP2	retinitis pigmentosa 2 (X-linked recessive)	0,003925	-1,54	0,011492	-1,54	0,995745	1,00	0,124187	-1,26	0,075348	1,23	0,062321	1,23
11750757_a_at	79871	RPAP2	RNA polymerase II associated protein 2	0,00285	-1,58	0,005332	-1,63	0,906778	1,04	0,286954	-1,18	0,005172	1,39	0,007145	1,34
11729183_a_at	79657	RPAP3	RNA polymerase II associated protein 3	0,004247	-1,64	0,024997	-1,55	0,865219	-1,06	0,068482	-1,37	0,356319	1,14	0,156456	1,20
11741509_a_at	6120	RPE	ribulose-5-phosphate-3-epimerase	0,00158	-1,67	0,009497	-1,61	0,901791	-1,04	0,431822	-1,14	0,005142	1,41	0,000787	1,47
11755341_x_at	6120	RPE	ribulose-5-phosphate-3-epimerase	0,002349	-1,78	0,012554	-1,71	0,911129	-1,04	0,479675	-1,15	0,006141	1,49	0,001094	1,55
11726065_a_at	6120	RPE	ribulose-5-phosphate-3-epimerase	0,001735	-1,95	0,009057	-1,87	0,926473	-1,04	0,085812	-1,42	0,091194	1,31	0,03927	1,37
11742812_a_at	6197	RPS6KA3	ribosomal protein S6 kinase, 90kDa, polypeptide 3	0,000602	-1,78	0,006037	-1,67	0,824117	-1,07	0,147501	-1,26	0,023244	1,33	0,002708	1,42
11742811_at	6197	RPS6KA3	ribosomal protein S6 kinase, 90kDa, polypeptide 3	0,008722	-1,77	0,010739	-1,91	0,857003	1,08	0,049473	-1,53	0,206173	1,24	0,403986	1,16
11723301_s_at	6198	RPS6KB1	ribosomal protein S6 kinase, 70kDa, polypeptide 1	0,000666	-1,94	0,005185	-1,83	0,879093	-1,06	3,77E-05	-2,30	0,117581	-1,25	0,225859	-1,19
11747836_a_at	6198	RPS6KB1	ribosomal protein S6 kinase, 70kDa, polypeptide 1	0,000376	-2,00	0,010834	-1,71	0,613614	-1,17	0,014283	-1,56	0,551767	1,09	0,065067	1,28
11723300_at	6198	RPS6KB1	ribosomal protein S6 kinase, 70kDa, polypeptide 1	0,000594	-2,15	0,004766	-2,02	0,880147	-1,06	0,000832	-2,08	0,876412	-1,03	0,863969	1,03
11750648_x_at	6198	RPS6KB1	ribosomal protein S6 kinase, 70kDa, polypeptide 1	0,000993	-1,76	0,020009	-1,55	0,653377	-1,14	0,057902	-1,36	0,336733	1,14	0,034287	1,29
11723299_a_at	6198	RPS6KB1	ribosomal protein S6 kinase, 70kDa, polypeptide 1	0,001156	-1,84	0,024214	-1,59	0,643497	-1,16	0,0265	-1,49	0,672969	1,07	0,120824	1,23
11758606_s_at	54665	RSBN1	round spermatid basic protein 1	0,00018	-2,17	0,000715	-2,20	0,970311	1,02	8,63E-05	-2,24	0,928411	-1,02	0,85419	-1,03
11724547_a_at	54665	RSBN1	round spermatid basic protein 1	0,000487	-1,89	0,001654	-1,92	0,961678	1,02	0,003414	-1,66	0,293854	1,15	0,348861	1,13
11743176_s_at	51773	RSF1	remodeling and spacing factor 1	2,14E-05	-3,15	0,000438	-2,81	0,782417	-1,12	2,13E-05	-3,02	0,718032	-1,08	0,838766	1,04
11743179_a_at	51773	RSF1	remodeling and spacing factor 1	5,98E-05	-1,72	0,000724	-1,65	0,863848	-1,04	6,45E-06	-1,85	0,234714	-1,12	0,439208	-1,08
11750256_a_at	51773	RSF1	remodeling and spacing factor 1	0,00019	-1,99	0,004887	-1,76	0,672211	-1,13	0,000299	-1,91	0,549664	-1,09	0,787833	1,04

11754462_a_at	89970	RSPRY1	ring finger and SPRY domain containing 1	0,000497	-1,55	0,001118	-1,60	0,88932	1,03	0,022369	-1,31	0,029306	1,22	0,053678	1,18
11733656_s_at	65117	RSRC2	arginine/serine-rich coiled-coil 2	0,000171	-2,04	0,002344	-1,90	0,821375	-1,07	0,003155	-1,69	0,403274	1,13	0,147923	1,21
11743490_a_at	65117	RSRC2	arginine/serine-rich coiled-coil 2	0,000993	-2,18	0,004959	-2,12	0,953108	-1,03	0,012181	-1,77	0,323241	1,20	0,228021	1,23
11743270_a_at	65117	RSRC2	arginine/serine-rich coiled-coil 2	0,000898	-2,33	0,004961	-2,23	0,936847	-1,04	0,007092	-1,93	0,470288	1,15	0,327637	1,20
11720370_x_at	8634	RTCA	RNA 3'-terminal phosphate cyclase	6,52E-05	-1,50	0,000356	-1,51	0,992069	1,00	0,154195	-1,14	0,000152	1,32	4,66E-05	1,32
11747758_a_at	8634	RTCA	RNA 3'-terminal phosphate cyclase	0,000982	-1,68	0,016742	-1,52	0,690186	-1,11	0,017152	-1,43	0,670694	1,06	0,161199	1,17
11722397_at	201965	RWDD4	RWD domain containing 4	0,003671	-1,81	0,047224	-1,58	0,697138	-1,15	0,192076	-1,30	0,232279	1,21	0,025914	1,39
11722398_s_at	201965	RWDD4	RWD domain containing 4	0,002182	-1,68	0,027432	-1,52	0,721938	-1,11	0,013152	-1,51	0,977873	1,00	0,413112	1,11
11717540_a_at	23429	RYBP	RING1 and YY1 binding protein	0,0021	-1,56	0,007711	-1,55	0,984919	-1,01	0,000459	-1,69	0,485753	-1,08	0,512202	-1,08
11755387_a_at	22908	SACM1L	SAC1 suppressor of actin mutations 1-like (yeast)	0,004915	-1,55	0,015226	-1,54	0,988159	-1,01	0,027627	-1,41	0,466864	1,10	0,425402	1,10
11724139_s_at	26278	SACS	sacsin molecular chaperone	0,004724	-1,85	0,049493	-1,62	0,730412	-1,14	0,000536	-2,19	0,075565	-1,35	0,314041	-1,18
11739233_a_at	9667	SAFB2	scaffold attachment factor B2	0,000318	-2,43	0,007366	-2,05	0,667877	-1,19	0,005571	-1,90	0,706374	1,08	0,155989	1,28
11752362_a_at	55206	SBNO1	strawberry notch homolog 1 (Drosophila)	2,68E-05	-2,56	0,000873	-2,22	0,684394	-1,15	0,000184	-2,19	0,926273	1,02	0,30667	1,17
11743925_s_at	55206	SBNO1	strawberry notch homolog 1 (Drosophila)	3,31E-05	-2,29	0,000971	-2,04	0,713945	-1,12	4,21E-05	-2,21	0,597417	-1,08	0,823457	1,04
11759521_a_at	55206	SBNO1	strawberry notch homolog 1 (Drosophila)	0,00022	-1,83	0,006003	-1,63	0,652646	-1,13	0,080024	-1,30	0,057726	1,25	0,001849	1,41
11752363_x_at	55206	SBNO1	strawberry notch homolog 1 (Drosophila)	0,000108	-2,39	0,001882	-2,16	0,784448	-1,11	0,000488	-2,11	0,89819	1,02	0,444049	1,13
11754753_a_at	9169	SCAF11	SR-related CTD-associated factor 11	1,54E-05	-3,21	0,000292	-2,91	0,813365	-1,11	1,68E-05	-3,06	0,807785	-1,05	0,811232	1,05
11723357_x_at	9169	SCAF11	SR-related CTD-associated factor 11	2,11E-05	-3,18	0,000276	-2,96	0,878832	-1,07	2,25E-05	-3,03	0,920389	-1,02	0,814016	1,05
11723358_s_at	9169	SCAF11	SR-related CTD-associated factor 11	0,00313	-1,96	0,024903	-1,78	0,807484	-1,10	0,001641	-2,07	0,403291	-1,17	0,786821	-1,06
11733055_a_at	9522	SCAMP1	secretory carrier membrane protein 1	0,000113	-2,08	0,002714	-1,86	0,720053	-1,11	0,023645	-1,47	0,080625	1,26	0,006082	1,41
11733051_a_at	9522	SCAMP1	secretory carrier membrane protein 1	0,002096	-1,55	0,007963	-1,54	0,980107	-1,01	0,007179	-1,46	0,652923	1,05	0,595146	1,06
11715895_at	950	SCARB2	scavenger receptor class B, member 2	0,000977	-2,10	0,024266	-1,74	0,6122	-1,21	0,234594	-1,30	0,079361	1,34	0,001996	1,62
11717197_x_at	23256	SCFD1	sec1 family domain containing 1	0,000242	-1,76	0,003223	-1,66	0,805227	-1,07	0,000249	-1,74	0,676202	-1,05	0,92703	1,01
11750876_a_at	23256	SCFD1	sec1 family domain containing 1	0,000473	-2,01	0,006194	-1,82	0,768888	-1,10	0,017092	-1,56	0,30793	1,17	0,070371	1,29
11751213_s_at	60592	SCOC	short coiled-coil protein	0,002972	-1,79	0,014153	-1,72	0,926947	-1,04	0,191941	-1,29	0,051649	1,34	0,019199	1,39
11750164_a_at	79634	SCRN3	secernin 3	0,000276	-1,73	0,004006	-1,61	0,779909	-1,07	0,000334	-1,70	0,671579	-1,05	0,880422	1,02
11759298_s_at	55681	SCYL2	SCY1-like 2 (S. cerevisiae)	0,000426	-1,81	0,002057	-1,80	0,989357	-1,01	0,008623	-1,51	0,164538	1,19	0,13338	1,19

11739336_at	55681	SCYL2	SCY1-like 2 (S. cerevisiae)	0,013189	-1,68	0,04176	-1,63	0,948456	-1,03	0,625712	-1,12	0,021441	1,46	0,00786	1,51
11756685_a_at	11196	SEC23IP	SEC23 interacting protein	0,002854	-1,82	0,006483	-1,87	0,946383	1,03	0,06564	-1,43	0,084111	1,30	0,106541	1,27
11749637_a_at	10427	SEC24B	SEC24 family member B	0,010563	-1,71	0,014929	-1,80	0,8947	1,06	0,092782	-1,42	0,150262	1,27	0,259314	1,20
11752899_s_at	7095	SEC62	SEC62 homolog (S. cerevisiae)	0,000177	-1,66	0,001134	-1,64	0,959746	-1,01	0,002363	-1,47	0,282317	1,11	0,201925	1,13
11731449_s_at	7095	SEC62	SEC62 homolog (S. cerevisiae)	0,000563	-1,75	0,006302	-1,63	0,800222	-1,07	0,013955	-1,46	0,365647	1,12	0,113277	1,20
11747209_s_at	7095	SEC62	SEC62 homolog (S. cerevisiae)	0,001211	-1,78	0,002819	-1,86	0,904632	1,04	0,011418	-1,55	0,179668	1,20	0,292345	1,15
11731453_at	7095	SEC62	SEC62 homolog (S. cerevisiae)	0,010719	-1,71	0,046148	-1,62	0,894182	-1,06	0,779691	-1,07	0,011975	1,51	0,002171	1,60
11731450_s_at	7095	SEC62	SEC62 homolog (S. cerevisiae)	0,002436	-1,58	0,003648	-1,67	0,84258	1,06	0,014931	-1,43	0,194573	1,16	0,407075	1,10
11758436_s_at	7095	SEC62	SEC62 homolog (S. cerevisiae)	0,001499	-1,84	0,011717	-1,72	0,847843	-1,07	0,002174	-1,79	0,797863	-1,04	0,884511	1,03
11731452_s_at	7095	SEC62	SEC62 homolog (S. cerevisiae)	0,001688	-1,92	0,008123	-1,86	0,945733	-1,03	0,004769	-1,78	0,800798	1,05	0,660963	1,08
11718863_at	11231	SEC63	SEC63 homolog (S. cerevisiae)	0,003672	-1,58	0,011834	-1,57	0,990749	-1,00	0,000548	-1,75	0,383004	-1,12	0,389067	-1,11
11744132_at	9728	SECISBP2L	SECIS binding protein 2-like	0,008913	-1,71	0,049947	-1,58	0,842132	-1,08	0,015002	-1,65	0,819463	-1,04	0,852448	1,04
11717203_at	6400	SEL1L	sel-1 suppressor of lin-12-like (C. elegans)	0,038151	-1,44	0,041129	-1,52	0,881947	1,05	0,917889	1,02	0,001914	1,55	0,00303	1,47
11716028_x_at	51714	SELT	selenoprotein T	0,032511	-1,52	0,025047	-1,66	0,798081	1,10	0,460023	1,17	3,98E-05	1,94	9,51E-05	1,77
11716027_at	51714	SELT	selenoprotein T	0,034568	-1,45	0,030042	-1,56	0,828214	1,07	0,555114	1,12	0,000113	1,74	0,000225	1,62
11717388_x_at	55752	SEPT11	septin 11	1,92E-06	-3,32	4,21E-05	-2,99	0,764326	-1,11	1,60E-05	-2,62	0,421395	1,14	0,111622	1,27
11725008_a_at	23157	SEPT6	septin 6	0,000671	-2,09	0,034911	-1,64	0,500822	-1,28	0,000513	-2,12	0,113801	-1,29	0,954544	-1,01
11734791_x_at	23157	SEPT6	septin 6	0,000708	-2,00	0,023835	-1,65	0,569198	-1,21	0,000595	-2,01	0,202089	-1,22	0,979737	-1,00
11741022_x_at	989	SEPT7	septin 7	0,000745	-2,07	0,004943	-1,98	0,908988	-1,05	0,027252	-1,57	0,158417	1,26	0,069529	1,32
11736062_s_at	989	SEPT7	septin 7	0,001427	-2,35	0,006462	-2,28	0,959738	-1,03	0,082542	-1,56	0,06184	1,46	0,032232	1,50
11745246_x_at	989	SEPT7	septin 7	0,005645	-1,67	0,021821	-1,62	0,941254	-1,03	0,341525	-1,20	0,034726	1,36	0,013214	1,40
11718574_at	26135	SERBP1	SERPINE1 mRNA binding protein 1	4,42E-05	-2,35	0,000546	-2,21	0,869026	-1,06	0,000289	-2,04	0,603534	1,08	0,332067	1,15
11758800_x_at	26135	SERBP1	SERPINE1 mRNA binding protein 1	0,001405	-1,70	0,012851	-1,59	0,813444	-1,07	0,405563	-1,15	0,009946	1,38	0,000788	1,48
11731539_at	91404	SESTD1	SEC14 and spectrin domains 1	0,001772	-1,55	0,000831	-1,75	0,607643	1,13	0,000652	-1,63	0,514748	1,08	0,685135	-1,05
11716194_a_at	29072	SETD2	SET domain containing 2	0,001944	-2,22	0,019331	-1,95	0,77759	-1,13	0,001461	-2,27	0,463088	-1,16	0,917484	-1,02
11755008_x_at	83852	SETDB2	SET domain, bifurcated 2	0,000529	-1,64	0,002695	-1,63	0,983101	-1,01	0,003422	-1,50	0,429742	1,09	0,378161	1,10
11742784_at	23064	SETX	senataxin	0,008803	-1,66	0,034005	-1,59	0,924842	-1,04	0,000463	-2,04	0,106349	-1,28	0,157504	-1,23
11716085_at	23451	SF3B1	splicing factor 3b, subunit 1, 155kDa	0,002089	-1,71	0,013644	-1,62	0,876301	-1,05	0,173123	-1,26	0,057978	1,29	0,014254	1,35
11745835_a_at	23451	SF3B1	splicing factor 3b, subunit 1, 155kDa	0,000642	-2,88	0,010385	-2,40	0,719684	-1,20	0,003652	-2,39	0,989085	1,00	0,419196	1,21
11716084_at	23451	SF3B1	splicing factor 3b, subunit 1, 155kDa	0,003633	-1,58	0,010573	-1,58	0,993738	1,00	0,063952	-1,33	0,154978	1,19	0,143868	1,19
11740064_a_at	119392	SFR1	SWI5-dependent recombination repair 1	0,000691	-1,76	0,016029	-1,55	0,642595	-1,14	2,23E-05	-2,09	0,014025	-1,35	0,139277	-1,19
11739456_at	259230	SGMS1	sphingomyelin synthase 1	0,00457	-1,58	0,024297	-1,51	0,88583	-1,05	0,938537	1,01	0,00078	1,54	6,45E-05	1,61
11753254_a_at	151246	SGOL2	shugoshin-like 2 (S. pombe)	0,003172	-2,10	0,016638	-1,98	0,903698	-1,06	0,000128	-2,78	0,081396	-1,40	0,137392	-1,32
11724054_a_at	151246	SGOL2	shugoshin-like 2 (S. pombe)	0,011301	-1,88	0,047421	-1,76	0,897265	-1,07	0,14039	-1,45	0,330154	1,22	0,168557	1,30

11718847_at	51100	SH3GLB1	SH3-domain GRB2-like endophilin B1	0,000639	-1,84	0,002821	-1,84	0,998639	-1,00	6,50E-05	-2,09	0,36443	-1,13	0,351169	-1,13
11724817_a_at	30011	SH3KBP1	SH3-domain kinase binding protein 1	0,00677	-2,06	0,017159	-2,08	0,990802	1,01	0,023924	-1,82	0,563455	1,14	0,581071	1,13
11728404_at	79801	SHCBP1	SHC SH2-domain binding protein 1	0,003061	-2,04	0,03381	-1,77	0,735941	-1,15	0,000685	-2,31	0,15264	-1,30	0,510232	-1,13
11722754_a_at	257218	SHPRH	SNF2 histone linker PHD RING helicase, E3 ubiquitin protein ligase	0,005208	-1,71	0,033725	-1,59	0,833694	-1,08	0,011407	-1,63	0,893409	-1,02	0,75985	1,05
11724011_at	80143	SIKE1	suppressor of IKBKE 1	0,006672	-1,71	0,033934	-1,61	0,879722	-1,06	0,000325	-2,12	0,077717	-1,31	0,152534	-1,24
11758114_x_at	8935	SKAP2	src kinase associated phosphoprotein 2	0,000162	-1,47	7,57E-05	-1,63	0,534516	1,11	0,00014	-1,47	0,15568	1,11	0,972267	1,00
11749275_a_at	8935	SKAP2	src kinase associated phosphoprotein 2	0,000446	-2,33	0,000459	-2,66	0,73629	1,14	0,016909	-1,71	0,012416	1,55	0,065496	1,36
11720465_a_at	23517	SKIV2L2	superkiller viralicidic activity 2-like 2 (S. cerevisiae)	0,000775	-1,97	0,003841	-1,94	0,96775	-1,02	0,017478	-1,58	0,177822	1,23	0,123129	1,25
11723721_a_at	57606	SLAIN2	SLAIN motif family, member 2	0,014941	-1,52	0,036525	-1,52	0,99135	-1,00	0,932133	-1,02	0,00362	1,49	0,001479	1,50
11745260_x_at	6558	SLC12A2	solute carrier family 12 (sodium/potassium/chloride transporter), member 2	0,005357	-1,79	0,032611	-1,66	0,847103	-1,08	0,007285	-1,75	0,75667	-1,06	0,923444	1,02
11717498_a_at	55186	SLC25A36	solute carrier family 25 (pyrimidine nucleotide carrier), member 36	8,41E-06	-2,89	0,000459	-2,40	0,589291	-1,21	5,04E-05	-2,43	0,93836	-1,01	0,265964	1,19
11717497_at	55186	SLC25A36	solute carrier family 25 (pyrimidine nucleotide carrier), member 36	0,000793	-1,90	0,012837	-1,69	0,711086	-1,13	0,001492	-1,82	0,637731	-1,08	0,776525	1,05
11747286_a_at	91137	SLC25A46	solute carrier family 25, member 46	0,003984	-1,93	0,035695	-1,71	0,772171	-1,12	0,162324	-1,37	0,211211	1,25	0,040267	1,40
11754594_a_at	1836	SLC26A2	solute carrier family 26 (anion exchanger), member 2	0,002748	-1,80	0,047033	-1,55	0,650333	-1,16	0,249901	-1,25	0,163351	1,24	0,009542	1,44
11732283_x_at	64924	SLC30A5	solute carrier family 30 (zinc transporter), member 5	0,002342	-1,76	0,006865	-1,77	0,984612	1,01	0,159887	-1,29	0,024833	1,37	0,019509	1,36
11744973_a_at	64924	SLC30A5	solute carrier family 30 (zinc transporter), member 5	0,000676	-1,79	0,005106	-1,71	0,884061	-1,05	0,018841	-1,46	0,234132	1,17	0,097726	1,22
11732282_a_at	64924	SLC30A5	solute carrier family 30 (zinc transporter), member 5	0,001653	-1,71	0,006461	-1,70	0,980855	-1,01	0,101515	-1,31	0,046487	1,29	0,027884	1,31
11759084_a_at	64924	SLC30A5	solute carrier family 30 (zinc transporter), member 5	0,006083	-1,86	0,028315	-1,75	0,90075	-1,06	0,021747	-1,67	0,814917	1,05	0,578209	1,11
11748617_a_at	10463	SLC30A9	solute carrier family 30 (zinc transporter), member 9	0,003124	-1,50	0,008659	-1,51	0,981535	1,01	0,515435	1,10	5,68E-06	1,66	1,36E-06	1,65
11762508_x_at	23443	SLC35A3	solute carrier family 35 (UDP-N-acetylglucosamine (UDP-GlcNAc) transporter), member A3	6,82E-06	-3,14	0,000275	-2,69	0,678886	-1,17	0,002309	-1,96	0,056133	1,38	0,002322	1,61
11730455_a_at	23443	SLC35A3	solute carrier family 35 (UDP-N-acetylglucosamine (UDP-GlcNAc) transporter), member A3	0,00234	-1,66	0,029098	-1,50	0,720984	-1,11	0,043753	-1,39	0,563996	1,08	0,14131	1,20
11758026_s_at	55032	SLC35A5	solute carrier family 35, member A5	0,002102	-1,95	0,012665	-1,84	0,891787	-1,06	0,013012	-1,70	0,648014	1,09	0,405292	1,15

11755882_a_at	51000	SLC35B3	solute carrier family 35 (adenosine 3'-phospho 5'-phosphosulfate transporter), member B	0,004537	-1,66	0,00569	-1,78	0,83438	1,07	0,090029	-1,35	0,045661	1,32	0,117707	1,23
11717102_a_at	81539	SLC38A1	solute carrier family 38, member 1	0,056033	-1,75	0,014664	-2,31	0,584843	1,32	9,53E-07	-5,43	0,000319	-2,35	7,46E-07	-3,11
11754821_s_at	81539	SLC38A1	solute carrier family 38, member 1	0,419347	-1,19	0,024056	-1,71	0,413308	1,44	0,00045	-2,15	0,15788	-1,26	0,000102	-1,81
11739000_a_at	54407	SLC38A2	solute carrier family 38, member 2	0,002044	-1,88	0,007211	-1,87	0,991824	-1,00	0,068167	-1,44	0,090275	1,30	0,0686	1,31
11722365_a_at	22950	SLC4A1AP	solute carrier family 4 (anion exchanger), member 1, adaptor protein	6,35E-05	-2,08	0,000604	-2,01	0,913189	-1,04	0,002302	-1,67	0,159949	1,20	0,073212	1,24
11746090_a_at	9748	SLK	STE20-like kinase	0,003526	-1,52	0,006523	-1,57	0,912078	1,03	0,796819	-1,04	0,000285	1,51	0,000263	1,46
11725220_a_at	9748	SLK	STE20-like kinase	0,000928	-1,74	0,002399	-1,80	0,917871	1,03	0,099528	-1,30	0,009211	1,38	0,012246	1,34
11725219_a_at	9748	SLK	STE20-like kinase	0,003318	-1,73	0,027402	-1,59	0,796008	-1,09	0,008867	-1,63	0,895277	-1,02	0,684956	1,07
11739194_s_at	7871	SLMAP	sarcolemma associated protein	0,000676	-1,59	0,004518	-1,54	0,912671	-1,03	0,159564	-1,20	0,012431	1,29	0,002755	1,32
11743583_at	51012	SLMO2	slowmo homolog 2 (Drosophila)	0,000976	-1,79	0,008131	-1,68	0,846549	-1,06	0,000571	-1,84	0,530793	-1,09	0,863963	-1,03
11755061_a_at	79811	SLTM	SAFB-like, transcription modulator	0,00141	-1,86	0,010755	-1,74	0,855918	-1,07	0,000698	-1,95	0,481642	-1,12	0,789983	-1,05
11742718_a_at	79811	SLTM	SAFB-like, transcription modulator	0,002411	-1,79	0,016286	-1,68	0,859179	-1,07	0,001067	-1,89	0,441775	-1,13	0,736767	-1,06
11742719_s_at	79811	SLTM	SAFB-like, transcription modulator	0,002596	-1,72	0,019049	-1,61	0,834564	-1,07	0,002836	-1,71	0,671775	-1,07	0,97937	1,00
11724469_at	10569	SLU7	SLU7 splicing factor homolog (S. cerevisiae)	0,000132	-1,88	0,001398	-1,80	0,879559	-1,05	6,13E-05	-1,93	0,58881	-1,07	0,865462	-1,02
11729717_a_at	4090	SMAD5	SMAD family member 5	0,000602	-2,22	0,007489	-1,99	0,777803	-1,12	0,000823	-2,15	0,676736	-1,08	0,872587	1,03
11743340_a_at	6595	SMARCA2	SWI/SNF related, matrix associated, actin dependent regulator of chromatin, subfamily a	0,000804	-2,23	0,01354	-1,91	0,703831	-1,16	0,03247	-1,62	0,373898	1,18	0,060662	1,37
11718423_a_at	8467	SMARCA5	SWI/SNF related, matrix associated, actin dependent regulator of chromatin, subfamily a	0,000469	-2,00	0,002922	-1,95	0,944918	-1,03	0,010804	-1,61	0,197798	1,21	0,118258	1,24
11764117_s_at	8467	SMARCA5	SWI/SNF related, matrix associated, actin dependent regulator of chromatin, subfamily a	0,001101	-1,83	0,004677	-1,81	0,982081	-1,01	0,000559	-1,90	0,759082	-1,05	0,807705	-1,04
11758006_s_at	56916	SMARCAD1	SWI/SNF-related, matrix-associated actin-dependent regulator of chromatin, subfamily a,	0,004114	-1,91	0,035196	-1,71	0,782949	-1,12	0,002732	-1,98	0,416678	-1,16	0,855404	-1,04
11742781_a_at	6599	SMARCC1	SWI/SNF related, matrix associated, actin dependent regulator of chromatin, subfamily c	3,16E-05	-1,79	0,000622	-1,68	0,78038	-1,06	3,57E-06	-1,92	0,184972	-1,14	0,497321	-1,07

11752770_a_at	6605	SMARCE1	SWI/SNF related, matrix associated, actin dependent regulator of chromatin, subfamily e	0,004628	-1,62	0,016527	-1,60	0,96568	-1,02	0,083482	-1,34	0,19239	1,19	0,132628	1,21
11752632_a_at	8243	SMC1A	structural maintenance of chromosomes 1A	0,001482	-1,88	0,003723	-1,94	0,933398	1,03	0,010883	-1,63	0,259329	1,19	0,355924	1,15
11745424_a_at	10592	SMC2	structural maintenance of chromosomes 2	0,005024	-1,99	0,043481	-1,75	0,768781	-1,14	0,000848	-2,33	0,136132	-1,33	0,413785	-1,17
11736527_a_at	10592	SMC2	structural maintenance of chromosomes 2	0,002735	-1,88	0,013635	-1,80	0,921225	-1,04	0,001352	-1,98	0,583472	-1,10	0,771396	-1,05
11740119_x_at	10592	SMC2	structural maintenance of chromosomes 2	0,010718	-1,60	0,026552	-1,60	0,997566	-1,00	0,365254	-1,19	0,040356	1,35	0,028109	1,35
11744868_a_at	10592	SMC2	structural maintenance of chromosomes 2	0,003528	-1,96	0,014928	-1,90	0,945035	-1,03	0,017244	-1,72	0,616765	1,10	0,47602	1,14
11740118_a_at	10592	SMC2	structural maintenance of chromosomes 2	0,005617	-1,97	0,020588	-1,91	0,950569	-1,03	0,019824	-1,77	0,712385	1,08	0,58434	1,12
11725679_at	9126	SMC3	structural maintenance of chromosomes 3	9,85E-06	-3,51	0,000278	-3,05	0,740224	-1,15	1,61E-07	-4,53	0,037541	-1,48	0,16853	-1,29
11725680_at	9126	SMC3	structural maintenance of chromosomes 3	6,35E-05	-2,41	0,003047	-2,02	0,603634	-1,19	6,87E-07	-3,13	0,004784	-1,55	0,074743	-1,30
11758700_s_at	9126	SMC3	structural maintenance of chromosomes 3	0,002865	-2,08	0,025652	-1,85	0,785347	-1,13	0,000755	-2,34	0,225006	-1,26	0,564422	-1,12
11733876_x_at	10051	SMC4	structural maintenance of chromosomes 4	9,21E-06	-2,43	0,000257	-2,22	0,756421	-1,10	7,62E-07	-2,66	0,173845	-1,20	0,518254	-1,09
11733875_a_at	10051	SMC4	structural maintenance of chromosomes 4	9,85E-06	-2,69	0,000274	-2,43	0,758332	-1,11	1,79E-06	-2,86	0,289745	-1,18	0,726044	-1,06
11733874_a_at	10051	SMC4	structural maintenance of chromosomes 4	0,00165	-2,16	0,006282	-2,13	0,984864	-1,01	0,080119	-1,51	0,061995	1,41	0,041211	1,43
11754320_a_at	10051	SMC4	structural maintenance of chromosomes 4	0,001199	-2,00	0,009023	-1,87	0,864073	-1,07	0,002158	-1,91	0,902948	-1,02	0,8082	1,05
11722517_a_at	23137	SMC5	structural maintenance of chromosomes 5	0,000629	-2,07	0,009018	-1,84	0,742542	-1,12	0,000323	-2,15	0,345697	-1,17	0,837715	-1,04
11724352_a_at	79677	SMC6	structural maintenance of chromosomes 6	0,002078	-1,56	0,010765	-1,52	0,923437	-1,03	0,001615	-1,58	0,740146	-1,04	0,925991	-1,01
11720586_at	23347	SMCHD1	structural maintenance of chromosomes flexible hinge domain containing 1	0,00598	-1,60	0,021007	-1,57	0,957135	-1,02	0,000729	-1,83	0,268127	-1,16	0,325824	-1,14
11720585_a_at	23347	SMCHD1	structural maintenance of chromosomes flexible hinge domain containing 1	0,002644	-1,66	0,009547	-1,64	0,979399	-1,01	0,027712	-1,44	0,311963	1,14	0,257912	1,15
11726056_a_at	57223	SMEK2	SMEK homolog 2, suppressor of mek1 (Dictyostelium)	0,001709	-1,56	0,00844	-1,53	0,938948	-1,02	0,002069	-1,55	0,918368	-1,01	0,943019	1,01
11723580_at	221710	SMIM13	small integral membrane protein 13	0,003824	-1,56	0,014525	-1,53	0,962673	-1,02	0,014118	-1,45	0,675888	1,06	0,573512	1,07
11734707_at	643155	SMIM15	small integral membrane protein 15	0,001248	-1,78	0,009751	-1,68	0,853971	-1,06	0,264738	-1,22	0,016903	1,38	0,002312	1,47

11753596_a_at	643155	SMIM15	small integral membrane protein 15	0,001657	-1,70	0,017623	-1,56	0,771336	-1,09	0,073636	-1,34	0,235989	1,17	0,047485	1,27
11739239_a_at	6619	SNAPC3	small nuclear RNA activating complex, polypeptide 3, 50kDa	0,008962	-1,63	0,018258	-1,66	0,964171	1,02	0,777565	-1,06	0,002378	1,57	0,00161	1,54
11724203_at	6632	SNRPD1	small nuclear ribonucleoprotein D1 polypeptide 16kDa	0,002456	-1,62	0,020715	-1,51	0,803932	-1,07	0,002532	-1,62	0,598641	-1,07	0,996814	1,00
11756871_x_at	29887	SNX10	sorting nexin 10	0,012718	-1,48	0,014006	-1,57	0,846019	1,06	0,266523	1,20	1,95E-06	1,87	2,19E-06	1,77
11754515_a_at	23161	SNX13	sorting nexin 13	0,000695	-1,74	0,001809	-1,80	0,914197	1,03	0,018439	-1,44	0,066502	1,25	0,103957	1,21
11747967_a_at	23161	SNX13	sorting nexin 13	0,004836	-1,52	0,015955	-1,51	0,978039	-1,01	0,017177	-1,42	0,649989	1,06	0,585751	1,07
11730052_a_at	64089	SNX16	sorting nexin 16	0,000119	-1,83	0,003328	-1,65	0,673881	-1,11	0,001336	-1,61	0,829561	1,03	0,227485	1,14
11742742_a_at	6643	SNX2	sorting nexin 2	0,001856	-1,48	0,001386	-1,61	0,686968	1,09	2,85E-05	-1,76	0,386712	-1,09	0,057114	-1,19
11721446_a_at	8723	SNX4	sorting nexin 4	0,000572	-1,64	0,002507	-1,64	0,997465	1,00	0,048707	-1,30	0,029995	1,26	0,021169	1,26
11736895_s_at	122809	SOCS4	suppressor of cytokine signaling 4	0,001067	-2,07	0,018979	-1,77	0,6787	-1,17	9,07E-05	-2,46	0,048422	-1,39	0,290884	-1,19
11729319_a_at	9655	SOCS5	suppressor of cytokine signaling 5	0,002265	-1,73	0,01624	-1,62	0,847336	-1,07	0,528324	1,12	2,52E-05	1,83	8,13E-07	1,95
11729320_a_at	9655	SOCS5	suppressor of cytokine signaling 5	0,000504	-1,83	0,008448	-1,65	0,718371	-1,11	0,014399	-1,49	0,455407	1,11	0,095462	1,22
11758088_s_at	9306	SOCS6	suppressor of cytokine signaling 6	0,001086	-1,88	0,03719	-1,55	0,550409	-1,21	2,71E-05	-2,35	0,00417	-1,52	0,102006	-1,25
11720892_at	6654	SOS1	son of sevenless homolog 1 (Drosophila)	0,000228	-2,26	0,004887	-1,97	0,701368	-1,15	0,021281	-1,60	0,197425	1,23	0,020636	1,41
11720893_s_at	6654	SOS1	son of sevenless homolog 1 (Drosophila)	0,004096	-1,88	0,030933	-1,71	0,809316	-1,10	0,037777	-1,57	0,646441	1,09	0,284816	1,20
11756963_a_at	6655	SOS2	son of sevenless homolog 2 (Drosophila)	0,006275	-1,60	0,031233	-1,52	0,886892	-1,05	0,291581	-1,20	0,077102	1,27	0,022717	1,33
11731618_a_at	6670	SP3	Sp3 transcription factor	0,058429	-1,40	0,04438	-1,52	0,810258	1,08	0,028017	1,49	1,63E-07	2,27	2,21E-07	2,09
11761980_a_at	6670	SP3	Sp3 transcription factor	0,002073	-1,70	0,005567	-1,73	0,965352	1,02	0,524779	-1,12	0,001083	1,55	0,00063	1,52
11723294_x_at	6670	SP3	Sp3 transcription factor	0,000376	-1,96	0,002569	-1,90	0,934358	-1,03	0,079482	-1,36	0,014986	1,40	0,004294	1,44
11725243_a_at	6683	SPAST	spastin	0,001969	-1,72	0,020035	-1,57	0,771737	-1,09	0,004092	-1,64	0,769862	-1,04	0,768108	1,04
11719949_a_at	54908	SPDL1	spindle apparatus coiled-coil protein 1	0,003784	-1,97	0,017782	-1,88	0,919139	-1,05	0,085627	-1,49	0,20322	1,26	0,103106	1,33
11758513_s_at	23013	SPEN	spen family transcriptional repressor	0,001877	-2,33	0,009609	-2,21	0,926461	-1,05	0,156904	-1,45	0,042142	1,52	0,014708	1,60
11743665_at	23013	SPEN	spen family transcriptional repressor	0,005842	-2,44	0,030038	-2,22	0,882209	-1,10	0,263306	-1,44	0,086835	1,54	0,025643	1,69
11748660_a_at	23111	SPG20	spastic paraplegia 20 (Troyer syndrome)	0,000857	-1,90	0,005343	-1,82	0,913685	-1,04	0,100593	-1,35	0,035542	1,35	0,01065	1,41
11730220_a_at	23111	SPG20	spastic paraplegia 20 (Troyer syndrome)	0,002567	-1,70	0,015439	-1,61	0,882479	-1,05	0,081857	-1,35	0,181621	1,20	0,068808	1,26

11720100_a_at	10927	SPIN1	spindlin 1	0,001213	-2,03	0,009005	-1,89	0,86875	-1,07	0,007953	-1,76	0,673272	1,08	0,388362	1,15
11749815_a_at	10927	SPIN1	spindlin 1	0,00238	-1,97	0,006311	-2,01	0,968002	1,02	0,032895	-1,59	0,175041	1,26	0,198079	1,24
11720102_a_at	10927	SPIN1	spindlin 1	0,003221	-1,78	0,017701	-1,69	0,891842	-1,05	0,041688	-1,48	0,392678	1,14	0,205802	1,20
11720645_at	9517	SPTLC2	serine palmitoyltransferase, long chain base subunit 2	0,003539	-1,58	0,020695	-1,51	0,875506	-1,05	0,065634	-1,33	0,310748	1,13	0,135498	1,19
11755038_s_at	140890	SREK1	splicing regulatory glutamine/lysine-rich protein 1	0,007222	-1,96	0,042603	-1,78	0,840789	-1,10	0,002836	-2,15	0,360841	-1,20	0,667296	-1,10
11739378_a_at	140890	SREK1	splicing regulatory glutamine/lysine-rich protein 1	0,010141	-1,86	0,043331	-1,75	0,897592	-1,06	0,016008	-1,80	0,902938	-1,03	0,87461	1,04
11725844_at	285672	SREK1IP1	SREK1-interacting protein 1	0,005874	-1,67	0,018838	-1,65	0,975046	-1,01	0,002244	-1,79	0,601135	-1,09	0,662105	-1,07
11736452_at	153443	SRFBP1	serum response factor binding protein 1	0,004623	-1,60	0,017391	-1,56	0,955218	-1,02	0,089214	-1,32	0,190128	1,18	0,121606	1,21
11748482_a_at	6729	SRP54	signal recognition particle 54kDa	0,000626	-1,82	0,004887	-1,73	0,882209	-1,05	0,046043	-1,39	0,087862	1,25	0,02607	1,31
11743300_a_at	6731	SRP72	signal recognition particle 72kDa	0,000151	-1,71	0,003144	-1,58	0,731426	-1,08	0,000322	-1,63	0,774174	-1,03	0,679146	1,05
11743301_a_at	6731	SRP72	signal recognition particle 72kDa	0,000911	-1,70	0,013419	-1,55	0,723602	-1,10	0,005366	-1,54	0,969851	1,01	0,408807	1,11
11753597_s_at	6726	SRP9	signal recognition particle 9kDa	0,00357	-1,81	0,01928	-1,71	0,891311	-1,06	0,000176	-2,23	0,090334	-1,30	0,163722	-1,23
11718851_a_at	6732	SRPK1	SRSF protein kinase 1	0,000444	-2,20	0,013064	-1,82	0,604382	-1,21	0,000572	-2,13	0,361948	-1,17	0,867813	1,03
11716512_a_at	23524	SRRM2	serine/arginine repetitive matrix 2	0,005408	-1,72	0,022033	-1,66	0,932241	-1,04	0,013142	-1,62	0,888413	1,03	0,72125	1,06
11743269_a_at	6426	SRSF1	serine/arginine-rich splicing factor 1	0,002725	-1,67	0,024498	-1,54	0,787075	-1,08	0,010717	-1,54	0,99796	1,00	0,550151	1,09
11721309_x_at	10772	SRSF10	serine/arginine-rich splicing factor 10	2,85E-05	-2,82	0,000478	-2,57	0,817418	-1,10	5,71E-07	-3,58	0,05406	-1,39	0,151583	-1,27
11721311_s_at	10772	SRSF10	serine/arginine-rich splicing factor 10	0,00022	-2,27	0,001894	-2,17	0,908309	-1,05	9,69E-06	-2,76	0,131505	-1,27	0,21313	-1,21
11718505_s_at	10772	SRSF10	serine/arginine-rich splicing factor 10	0,000745	-2,06	0,006767	-1,91	0,837535	-1,08	2,79E-05	-2,57	0,063661	-1,34	0,158384	-1,24
11758797_s_at	10772	SRSF10	serine/arginine-rich splicing factor 10	0,001969	-1,85	0,012791	-1,74	0,878699	-1,06	0,006755	-1,70	0,877307	1,03	0,592664	1,09
11754191_x_at	9295	SRSF11	serine/arginine-rich splicing factor 11	0,000993	-1,75	0,011293	-1,61	0,776952	-1,09	9,58E-05	-1,99	0,103374	-1,23	0,323227	-1,13
11754190_a_at	9295	SRSF11	serine/arginine-rich splicing factor 11	0,002386	-1,60	0,017437	-1,52	0,84045	-1,06	0,000235	-1,81	0,134228	-1,20	0,303131	-1,13
11761781_a_at	9295	SRSF11	serine/arginine-rich splicing factor 11	0,004244	-2,49	0,040605	-2,09	0,755363	-1,19	0,002462	-2,67	0,342867	-1,27	0,809579	-1,07
11747986_a_at	6760	SS18	synovial sarcoma translocation, chromosome 18	0,00016	-2,04	0,006879	-1,73	0,572797	-1,18	0,004034	-1,66	0,791413	1,04	0,106818	1,23
11722800_a_at	6760	SS18	synovial sarcoma translocation, chromosome 18	0,000592	-2,12	0,041069	-1,62	0,469931	-1,31	0,131332	-1,37	0,307131	1,18	0,003428	1,55
11763739_s_at	6745	SSR1	signal sequence receptor, alpha	0,003439	-1,50	0,00654	-1,55	0,917871	1,03	0,146779	-1,22	0,026775	1,27	0,039107	1,23
11715750_at	6749	SSRP1	structure specific recognition protein 1	3,23E-05	-1,99	0,004735	-1,63	0,462216	-1,22	1,32E-05	-2,04	0,051197	-1,25	0,862819	-1,02

11753914_a_at	117178	SSX2IP	synovial sarcoma, X breakpoint 2 interacting protein	0,000771	-1,72	0,0033	-1,71	0,99135	-1,00	0,203349	-1,22	0,004457	1,41	0,001902	1,41
11753386_s_at	6767 /// 145165	ST13 /// ST13P4	suppression of tumorigenicity 13 (colon carcinoma) (Hsp70 interacting protein) /// supp	0,000446	-1,90	0,015329	-1,61	0,575306	-1,18	4,10E-06	-2,44	0,001942	-1,52	0,044523	-1,29
11743454_s_at	6767 /// 144106 /// 145165	ST13 /// ST13P4 /// ST13P5	suppression of tumorigenicity 13 (colon carcinoma) (Hsp70 interacting protein) /// supp	0,000469	-1,59	0,004961	-1,50	0,81756	-1,05	6,79E-05	-1,70	0,202369	-1,13	0,461349	-1,08
11743276_s_at	10274	STAG1	stromal antigen 1	0,003254	-1,70	0,008007	-1,74	0,962972	1,02	0,00146	-1,79	0,833088	-1,03	0,734006	-1,05
11739903_a_at	10735	STAG2	stromal antigen 2	4,09E-06	-2,65	4,21E-05	-2,60	0,959994	-1,02	0,004828	-1,65	0,001153	1,57	0,000247	1,60
11743520_a_at	10735	STAG2	stromal antigen 2	2,73E-06	-2,48	4,81E-05	-2,35	0,864732	-1,05	0,00061	-1,77	0,022394	1,33	0,003689	1,40
11731107_a_at	10735	STAG2	stromal antigen 2	0,001322	-1,87	0,017398	-1,67	0,735345	-1,12	0,001188	-1,88	0,446476	-1,13	0,976428	-1,01
11731594_at	10254	STAM2	signal transducing adaptor molecule (SH3 domain and ITAM motif) 2	0,007062	-1,79	0,03509	-1,68	0,882552	-1,07	9,71E-05	-2,49	0,020841	-1,48	0,039728	-1,39
11731595_x_at	10254	STAM2	signal transducing adaptor molecule (SH3 domain and ITAM motif) 2	0,010686	-1,69	0,045301	-1,60	0,897391	-1,05	0,000133	-2,32	0,019837	-1,45	0,033211	-1,38
11731592_at	10254	STAM2	signal transducing adaptor molecule (SH3 domain and ITAM motif) 2	0,002844	-1,64	0,014006	-1,59	0,922215	-1,03	0,000755	-1,77	0,408118	-1,12	0,56691	-1,08
11748216_a_at	27067	STAU2	staufen double-stranded RNA binding protein 2	2,50E-05	-2,24	0,000443	-2,08	0,810311	-1,08	0,006385	-1,58	0,037237	1,31	0,004529	1,41
11726372_a_at	27067	STAU2	staufen double-stranded RNA binding protein 2	0,003519	-1,70	0,012672	-1,67	0,973413	-1,01	0,882326	-1,03	0,000631	1,62	0,000144	1,65
11751932_x_at	6491	STIL	SCL/TAL1 interrupting locus	0,004952	-1,81	0,027311	-1,70	0,873293	-1,07	0,172682	-1,33	0,144415	1,27	0,047632	1,36
11732661_x_at	6491	STIL	SCL/TAL1 interrupting locus	0,002902	-1,93	0,037804	-1,67	0,704505	-1,15	0,011715	-1,73	0,854856	-1,04	0,54155	1,11
11758670_s_at	6788	STK3	serine/threonine kinase 3	0,00156	-1,91	0,012769	-1,77	0,834875	-1,08	0,000105	-2,29	0,0959	-1,29	0,231602	-1,20
11721154_a_at	6788	STK3	serine/threonine kinase 3	0,000489	-1,94	0,014006	-1,66	0,611602	-1,17	0,000368	-1,96	0,250039	-1,18	0,967802	-1,01
11727157_a_at	55342	STRBP	spermatid perinuclear RNA binding protein	0,002003	-1,59	0,006524	-1,59	0,997485	1,00	0,000164	-1,80	0,288134	-1,13	0,265348	-1,13
11759036_at	201595	STT3B	STT3B, subunit of the oligosaccharyltransferase complex (catalytic)	0,004302	-1,72	0,008953	-1,77	0,940338	1,03	0,90235	1,03	7,79E-05	1,82	4,43E-05	1,76
11756482_x_at	201595	STT3B	STT3B, subunit of the oligosaccharyltransferase complex (catalytic)	0,000175	-2,86	0,003047	-2,49	0,764775	-1,15	2,36E-05	-3,31	0,158832	-1,33	0,470325	-1,16
11754645_x_at	201595	STT3B	STT3B, subunit of the oligosaccharyltransferase complex (catalytic)	0,000441	-2,26	0,006468	-2,00	0,743248	-1,13	0,000288	-2,30	0,425765	-1,15	0,937754	-1,02
11759035_x_at	201595	STT3B	STT3B, subunit of the oligosaccharyltransferase complex (catalytic)	0,010464	-1,51	0,014279	-1,57	0,887953	1,05	0,053644	-1,36	0,25569	1,16	0,430893	1,11

11759034_at	201595	STT3B	STT3B, subunit of the oligosaccharyltransferase complex (catalytic)	0,011368	-1,49	0,018727	-1,54	0,931874	1,03	0,081357	-1,32	0,227106	1,17	0,316836	1,13
11719190_a_at	6814	STXBP3	syntaxin binding protein 3	0,001222	-1,59	0,007301	-1,55	0,913849	-1,03	0,021869	-1,38	0,291342	1,12	0,159115	1,16
11736429_at	6815	STYX	serine/threonine/tyrosine interacting protein	0,004549	-1,64	0,019148	-1,60	0,933803	-1,03	0,015002	-1,53	0,780021	1,04	0,612711	1,08
11748619_a_at	8803	SUCLA2	succinate-CoA ligase, ADP-forming, beta subunit	0,000405	-2,55	0,004131	-2,32	0,835755	-1,10	0,13647	-1,44	0,014007	1,60	0,001519	1,76
11748620_x_at	8803	SUCLA2	succinate-CoA ligase, ADP-forming, beta subunit	0,000165	-2,45	0,00323	-2,15	0,736602	-1,14	0,025541	-1,62	0,098308	1,33	0,009624	1,51
11755492_a_at	8803	SUCLA2	succinate-CoA ligase, ADP-forming, beta subunit	0,001472	-2,21	0,011217	-2,03	0,854454	-1,09	0,217938	-1,35	0,029748	1,50	0,004931	1,64
11759519_a_at	8803	SUCLA2	succinate-CoA ligase, ADP-forming, beta subunit	0,003477	-1,83	0,026173	-1,68	0,814146	-1,09	0,191262	-1,31	0,119466	1,28	0,024208	1,40
11755266_x_at	8803	SUCLA2	succinate-CoA ligase, ADP-forming, beta subunit	0,005741	-1,68	0,042792	-1,54	0,796008	-1,09	0,223747	-1,26	0,171554	1,22	0,035391	1,33
11733477_at	56670	SUCNR1	succinate receptor 1	0,161707	-1,37	0,049365	-1,67	0,619166	1,22	0,070403	-1,51	0,602543	1,11	0,61532	-1,10
11717622_s_at	51430	SUCO	SUN domain containing ossification factor	0,001058	-1,89	0,009732	-1,75	0,82008	-1,08	0,00232	-1,79	0,894508	-1,02	0,733533	1,06
11717623_a_at	51430	SUCO	SUN domain containing ossification factor	0,004892	-1,63	0,01234	-1,65	0,97894	1,01	0,054815	-1,39	0,216144	1,19	0,229456	1,17
11739402_a_at	51111	SUV420H1	suppressor of variegation 4-20 homolog 1 (Drosophila)	0,000376	-2,27	0,002378	-2,20	0,949522	-1,03	9,64E-05	-2,47	0,527842	-1,12	0,64425	-1,09
11739403_at	51111	SUV420H1	suppressor of variegation 4-20 homolog 1 (Drosophila)	0,000598	-1,97	0,001783	-2,03	0,937494	1,03	8,76E-05	-2,20	0,598985	-1,09	0,444544	-1,12
11752144_a_at	51111	SUV420H1	suppressor of variegation 4-20 homolog 1 (Drosophila)	0,005726	-1,70	0,020154	-1,66	0,958547	-1,02	0,005465	-1,71	0,865272	-1,03	0,968319	-1,01
11739485_a_at	23512	SUZ12	SUZ12 polycomb repressive complex 2 subunit	0,000638	-2,47	0,003211	-2,43	0,975529	-1,02	0,354531	-1,27	0,001092	1,91	0,000281	1,95
11739486_at	23512	SUZ12	SUZ12 polycomb repressive complex 2 subunit	0,002626	-1,76	0,040239	-1,54	0,673909	-1,14	2,53E-05	-2,36	0,003337	-1,53	0,031558	-1,34
11759039_at	258010	SVIP	small VCP/p97-interacting protein	0,002417	-1,62	0,013142	-1,56	0,908405	-1,04	0,981403	1,00	0,000284	1,57	2,54E-05	1,63
11721501_x_at	23075	SWAP70	SWAP switching B-cell complex 70kDa subunit	1,21E-06	-3,27	3,74E-05	-2,93	0,74061	-1,12	6,00E-07	-3,03	0,834649	-1,04	0,639666	1,08
11721502_at	23075	SWAP70	SWAP switching B-cell complex 70kDa subunit	0,006218	-1,73	0,032694	-1,62	0,875506	-1,06	0,005409	-1,75	0,653447	-1,08	0,939193	-1,01
11720665_s_at	10492	SYNCRIP	synaptotagmin binding, cytoplasmic RNA interacting protein	6,73E-05	-2,67	0,002282	-2,27	0,67531	-1,17	8,53E-05	-2,56	0,506619	-1,13	0,84439	1,04
11744468_at	10492	SYNCRIP	synaptotagmin binding, cytoplasmic RNA interacting protein	0,005329	-1,94	0,043468	-1,72	0,780106	-1,13	0,000152	-2,61	0,024618	-1,51	0,093021	-1,34
11749175_a_at	9014	TAF1B	TATA box binding protein (TBP)-associated factor, RNA polymerase I, B, 63kDa	0,000922	-1,59	0,0056	-1,54	0,91644	-1,03	0,481726	-1,10	0,001425	1,40	0,00019	1,44

11721533_at	6873	TAF2	TAF2 RNA polymerase II, TATA box binding protein (TBP)-associated factor, 150kDa	0,000918	-2,04	0,010782	-1,84	0,771563	-1,11	0,001814	-1,93	0,774803	-1,05	0,762272	1,06
11734833_s_at	51616	TAF9B	TAF9B RNA polymerase II, TATA box binding protein (TBP)-associated factor, 31kDa	0,001101	-1,71	0,0065	-1,65	0,91689	-1,03	0,001418	-1,68	0,905164	-1,02	0,912696	1,02
11752780_a_at	10010	TANK	TRAF family member-associated NFKB activator	0,00672	-1,50	0,012946	-1,53	0,941239	1,02	0,43389	-1,13	0,008513	1,36	0,008989	1,33
11752781_s_at	10010	TANK	TRAF family member-associated NFKB activator	0,003476	-1,86	0,006189	-1,95	0,903849	1,05	0,125735	-1,38	0,031527	1,42	0,051976	1,35
11724596_s_at	10010	TANK	TRAF family member-associated NFKB activator	0,001854	-1,63	0,018165	-1,52	0,784723	-1,08	0,004548	-1,56	0,849162	-1,03	0,708361	1,05
11728444_a_at	57551	TAOK1	TAO kinase 1	0,008806	-1,82	0,009057	-2,01	0,817668	1,10	0,449955	-1,20	0,004209	1,68	0,012179	1,52
11718272_a_at	8887	TAX1BP1	Tax1 (human T-cell leukemia virus type I) binding protein 1	0,000495	-1,76	0,005725	-1,64	0,796008	-1,07	6,15E-05	-1,94	0,160088	-1,18	0,419005	-1,10
11718052_a_at	55773	TBC1D23	TBC1 domain family, member 23	0,001101	-1,79	0,023785	-1,56	0,639298	-1,15	0,009041	-1,57	0,959687	-1,01	0,324739	1,14
11725535_a_at	93594	TBC1D31	TBC1 domain family, member 31	0,005855	-1,67	0,008899	-1,75	0,889298	1,05	0,018338	-1,55	0,401342	1,13	0,628105	1,08
11755464_a_at	93594	TBC1D31	TBC1 domain family, member 31	0,007784	-1,56	0,025101	-1,54	0,964594	-1,02	0,037697	-1,42	0,560994	1,08	0,464416	1,10
11717932_at	29110	TBK1	TANK-binding kinase 1	0,004113	-1,78	0,047655	-1,57	0,715734	-1,14	0,682209	-1,09	0,020215	1,43	0,000757	1,63
11745230_a_at	285343	TCAIM	T cell activation inhibitor, mitochondrial	0,037452	-1,41	0,022481	-1,56	0,746994	1,10	0,87261	1,03	0,000444	1,61	0,002193	1,46
11745231_a_at	285343	TCAIM	T cell activation inhibitor, mitochondrial	0,002621	-1,67	0,006516	-1,70	0,959982	1,02	0,017946	-1,48	0,311328	1,14	0,371502	1,12
11755437_a_at	10915	TCERG1	transcription elongation regulator 1	0,00016	-2,77	0,004317	-2,30	0,669226	-1,20	0,000187	-2,67	0,462583	-1,16	0,878725	1,04
11748475_a_at	10915	TCERG1	transcription elongation regulator 1	0,002184	-1,82	0,042356	-1,56	0,6354	-1,17	0,004357	-1,74	0,478297	-1,12	0,785243	1,05
11745727_a_at	6938	TCF12	transcription factor 12	0,001199	-2,98	0,003607	-3,09	0,959493	1,04	0,335247	-1,38	0,001624	2,24	0,001089	2,16
11752247_a_at	6938	TCF12	transcription factor 12	0,012958	-1,53	0,018357	-1,60	0,906356	1,04	0,881716	1,03	0,000308	1,65	0,000308	1,58
11720580_a_at	6938	TCF12	transcription factor 12	0,000647	-2,03	0,004068	-1,96	0,930904	-1,04	0,002543	-1,84	0,702958	1,07	0,529633	1,11
11726957_a_at	6938	TCF12	transcription factor 12	0,000798	-2,00	0,003307	-1,99	0,996548	-1,00	0,002485	-1,84	0,62302	1,09	0,60814	1,09
11720581_s_at	6938	TCF12	transcription factor 12	0,006479	-1,52	0,016613	-1,52	0,990802	1,00	0,000637	-1,72	0,309311	-1,13	0,274804	-1,14
11745765_x_at	6925	TCF4	transcription factor 4	0,039555	-1,64	0,047652	-1,74	0,906124	1,06	1,60E-06	-3,87	5,51E-05	-2,22	3,70E-06	-2,36
11724244_s_at	6925	TCF4	transcription factor 4	0,015426	-1,83	0,017164	-1,99	0,858847	1,09	1,89E-06	-3,95	0,000604	-1,98	3,61E-05	-2,16
11724243_at	6925	TCF4	transcription factor 4	0,000655	-2,41	0,001321	-2,61	0,865219	1,08	1,20E-06	-3,94	0,031053	-1,51	0,005735	-1,63
11724241_at	6925	TCF4	transcription factor 4	0,011055	-1,79	0,015304	-1,90	0,893631	1,06	2,14E-05	-2,95	0,014404	-1,55	0,002721	-1,65

11763309_at	6925	TCF4	transcription factor 4	0,009901	-1,79	0,025684	-1,78	0,992391	-1,00	0,000152	-2,50	0,053288	-1,41	0,043343	-1,40
11724242_s_at	6925	TCF4	transcription factor 4	0,011285	-2,16	0,022286	-2,23	0,967159	1,03	0,000744	-2,94	0,257337	-1,32	0,189053	-1,36
11717404_x_at	51567	TDP2	tyrosyl-DNA phosphodiesterase 2	0,000446	-1,53	0,002354	-1,51	0,976769	-1,01	0,012159	-1,33	0,136587	1,14	0,097365	1,15
11717490_a_at	81550	TDRD3	tudor domain containing 3	0,001497	-1,61	0,014638	-1,50	0,796211	-1,07	0,00167	-1,60	0,619529	-1,06	0,964159	1,01
11732463_x_at	7013	TERF1	telomeric repeat binding factor (NIMA-interacting) 1	0,000199	-1,72	0,001356	-1,69	0,949668	-1,02	0,0001	-1,76	0,744965	-1,04	0,870832	-1,02
11757793_s_at	7035	TFPI	tissue factor pathway inhibitor (lipoprotein-associated coagulation inhibitor)	0,000901	-2,34	0,039184	-1,78	0,520002	-1,32	0,002387	-2,14	0,346966	-1,20	0,661084	1,09
11749320_a_at	23483	TGDS	TDP-glucose 4,6-dehydratase	0,000199	-1,97	0,003211	-1,81	0,773652	-1,09	0,003277	-1,65	0,518891	1,09	0,166117	1,19
11755595_a_at	23483	TGDS	TDP-glucose 4,6-dehydratase	0,000622	-1,89	0,011668	-1,67	0,689241	-1,13	0,02732	-1,47	0,376446	1,13	0,055149	1,28
11739341_a_at	23483	TGDS	TDP-glucose 4,6-dehydratase	0,000649	-1,70	0,006411	-1,60	0,823179	-1,06	0,018338	-1,42	0,301009	1,13	0,09693	1,20
11725142_a_at	96764	TGS1	trimethylguanosine synthase 1	0,00029	-1,87	0,017436	-1,55	0,504498	-1,21	0,00156	-1,69	0,523289	-1,09	0,41482	1,11
11757971_s_at	168451	THAP5	THAP domain containing 5	0,000175	-2,00	0,003384	-1,81	0,733614	-1,11	3,25E-05	-2,17	0,176234	-1,20	0,568909	-1,08
11740401_s_at	168451	THAP5	THAP domain containing 5	0,001456	-2,10	0,033948	-1,72	0,61209	-1,22	0,000194	-2,45	0,046005	-1,42	0,386574	-1,17
11758455_s_at	152815	THAP6	THAP domain containing 6	0,001601	-1,86	0,022036	-1,65	0,71607	-1,13	0,001945	-1,84	0,493998	-1,11	0,940591	1,01
11724862_at	79896	THNSL1	threonine synthase-like 1 (S. cerevisiae)	0,007575	-1,68	0,019783	-1,68	0,999283	1,00	0,183346	-1,30	0,088588	1,30	0,072913	1,29
11744605_a_at	57187	THOC2	THO complex 2	1,47E-05	-1,77	0,000257	-1,71	0,866664	-1,04	2,43E-07	-2,00	0,07056	-1,17	0,148722	-1,13
11734769_a_at	57187	THOC2	THO complex 2	3,26E-05	-1,91	0,000391	-1,84	0,895608	-1,04	6,70E-06	-2,01	0,427042	-1,09	0,644335	-1,05
11734766_a_at	57187	THOC2	THO complex 2	5,98E-05	-2,22	0,000723	-2,09	0,867925	-1,06	5,40E-05	-2,19	0,781205	-1,04	0,93695	1,01
11764244_s_at	57187	THOC2	THO complex 2	0,000131	-2,04	0,00101	-1,98	0,932241	-1,03	0,00017	-1,98	0,996805	1,00	0,840143	1,03
11759635_x_at	57187	THOC2	THO complex 2	0,000256	-1,96	0,00134	-1,94	0,986937	-1,01	0,000828	-1,80	0,603579	1,08	0,561638	1,08
11725969_a_at	55623	THUMPD1	THUMP domain containing 1	3,17E-05	-1,69	0,00032	-1,65	0,924449	-1,02	2,76E-05	-1,67	0,914575	-1,01	0,916679	1,01
11725967_a_at	55623	THUMPD1	THUMP domain containing 1	0,001065	-2,21	0,00849	-2,04	0,854284	-1,08	0,368018	-1,24	0,006318	1,65	0,000638	1,78
11723481_a_at	7072	TIA1	TIA1 cytotoxic granule-associated RNA binding protein	0,001782	-2,13	0,006889	-2,10	0,980334	-1,01	0,397535	-1,23	0,003627	1,71	0,001279	1,74
11757620_s_at	7072	TIA1	TIA1 cytotoxic granule-associated RNA binding protein	0,004001	-1,64	0,006199	-1,73	0,880167	1,05	0,302415	-1,20	0,005996	1,44	0,010801	1,37
11723482_s_at	7072	TIA1	TIA1 cytotoxic granule-associated RNA binding protein	0,001583	-1,75	0,004961	-1,77	0,981555	1,01	0,086867	-1,34	0,039563	1,32	0,034366	1,31
11716949_s_at	56889	TM9SF3	transmembrane 9 superfamily member 3	0,002662	-1,66	0,020814	-1,55	0,817524	-1,07	0,000268	-1,89	0,120893	-1,22	0,305629	-1,14

11725311_s_at	51014 /// 100302736	TMED7 /// TMED7-TICAM2	transmembrane emp24 protein transport domain containing 7 /// TMED7-TICAM2 readthrough	0,01878	-1,82	0,030305	-1,89	0,943772	1,04	0,020977	1,81	4,15E-08	3,42	1,07E-08	3,29
11721215_a_at	54664	TMEM106B	transmembrane protein 106B	0,001114	-2,16	0,009453	-1,98	0,839909	-1,09	0,821432	-1,06	0,000512	1,87	2,42E-05	2,04
11721214_a_at	54664	TMEM106B	transmembrane protein 106B	0,000498	-1,82	0,007422	-1,66	0,742542	-1,10	0,486776	-1,13	0,002335	1,47	5,65E-05	1,62
11739231_a_at	54664	TMEM106B	transmembrane protein 106B	0,000358	-1,99	0,002941	-1,90	0,894995	-1,05	0,238453	-1,24	0,002459	1,54	0,000291	1,61
11721216_s_at	54664	TMEM106B	transmembrane protein 106B	0,000204	-2,19	0,001869	-2,08	0,900568	-1,05	0,098644	-1,38	0,006459	1,51	0,001045	1,59
11721217_s_at	54664	TMEM106B	transmembrane protein 106B	0,00062	-2,00	0,00529	-1,87	0,859499	-1,07	0,000799	-1,95	0,813384	-1,04	0,890912	1,02
11736851_a_at	84314	TMEM107	transmembrane protein 107	0,041746	-1,74	0,018107	-2,12	0,680674	1,22	0,78365	-1,09	0,00226	1,95	0,020595	1,60
11715735_a_at	114908	TMEM123	transmembrane protein 123	8,87E-05	-2,50	0,002066	-2,20	0,732433	-1,14	9,33E-05	-2,44	0,563816	-1,11	0,89902	1,03
11752036_s_at	114908	TMEM123	transmembrane protein 123	0,001651	-1,64	0,007551	-1,61	0,955742	-1,02	0,066626	-1,32	0,098406	1,22	0,054851	1,24
11745789_a_at	65084	TMEM135	transmembrane protein 135	0,001661	-1,83	0,014494	-1,69	0,818155	-1,08	0,093052	-1,37	0,144675	1,24	0,032595	1,34
11739809_at	80008	TMEM156	transmembrane protein 156	0,00485	-1,60	0,018692	-1,56	0,948456	-1,02	0,000485	-1,83	0,224316	-1,17	0,285066	-1,15
11743574_x_at	55751	TMEM184C	transmembrane protein 184C	0,001952	-1,60	0,01436	-1,52	0,849403	-1,05	0,751583	-1,05	0,001706	1,44	0,000115	1,52
11743573_at	55751	TMEM184C	transmembrane protein 184C	0,002101	-1,96	0,018338	-1,78	0,804043	-1,10	0,644659	-1,11	0,004901	1,60	0,000282	1,76
11727474_a_at	54916	TMEM260	transmembrane protein 260	0,021421	-1,57	0,022084	-1,68	0,853971	1,07	0,788997	1,06	0,000259	1,78	0,000433	1,66
11722798_s_at	90488	TMEM263	transmembrane protein 263	0,00544	-1,78	0,020792	-1,72	0,945035	-1,03	2,94E-05	-2,58	0,012533	-1,49	0,013465	-1,45
11722797_a_at	90488	TMEM263	transmembrane protein 263	0,027493	-1,53	0,047093	-1,56	0,966939	1,02	0,00147	-1,90	0,199842	-1,22	0,140089	-1,24
11759179_at	55151	TMEM38B	transmembrane protein 38B	0,00211	-1,64	0,010835	-1,59	0,924842	-1,03	0,132138	-1,27	0,063882	1,26	0,024509	1,30
11729940_x_at	148534	TMEM56	transmembrane protein 56	0,034609	-1,89	0,023953	-2,21	0,776952	1,17	0,416765	1,29	2,62E-05	2,86	7,63E-05	2,44
11733932_s_at	137695	TMEM68	transmembrane protein 68	0,002747	-1,95	0,01833	-1,81	0,855193	-1,08	0,500974	-1,17	0,010529	1,55	0,001259	1,67
11755127_a_at	7110	TMF1	TATA element modulatory factor 1	0,000225	-2,34	0,002888	-2,15	0,823069	-1,09	0,071309	-1,47	0,021649	1,46	0,002445	1,60
11724752_at	7110	TMF1	TATA element modulatory factor 1	0,000217	-2,03	0,003607	-1,85	0,761801	-1,10	0,004447	-1,67	0,477677	1,11	0,134695	1,22
11721096_a_at	81542	TMX1	thioredoxin-related transmembrane protein 1	0,008888	-2,19	0,024344	-2,17	0,987226	-1,01	0,277756	1,39	7,78E-06	3,02	1,19E-06	3,05
11721097_at	81542	TMX1	thioredoxin-related transmembrane protein 1	0,010563	-1,49	0,020835	-1,52	0,965039	1,02	0,076864	-1,32	0,270338	1,15	0,315221	1,13
11721732_at	54495	TMX3	thioredoxin-related transmembrane protein 3	0,002008	-1,83	0,009221	-1,78	0,945761	-1,03	0,032888	-1,50	0,260311	1,19	0,167659	1,22
11721730_at	54495	TMX3	thioredoxin-related transmembrane protein 3	0,003748	-2,08	0,017167	-1,98	0,924182	-1,05	0,007386	-1,97	0,972973	1,01	0,794457	1,06
11739879_a_at	23043	TNIK	TRAF2 and NCK interacting kinase	0,002066	-1,52	0,005826	-1,53	0,973558	1,01	0,988823	-1,00	6,55E-05	1,53	2,34E-05	1,51

11716619_a_at	3842	TNPO1	transportin 1	0,000276	-2,14	0,03134	-1,61	0,436827	-1,33	0,000184	-2,16	0,048776	-1,35	0,946227	-1,01
11716621_a_at	3842	TNPO1	transportin 1	0,000199	-1,79	0,005216	-1,61	0,663166	-1,12	0,002573	-1,56	0,819668	1,03	0,207808	1,15
11716620_a_at	3842	TNPO1	transportin 1	0,001248	-2,18	0,008143	-2,05	0,896045	-1,06	0,00044	-2,37	0,454642	-1,15	0,67699	-1,09
11717437_x_at	7150	TOP1	topoisomerase (DNA) I	9,05E-05	-2,97	0,000919	-2,77	0,893435	-1,07	0,001103	-2,34	0,404281	1,19	0,215971	1,27
11717439_s_at	7150 /// 7151	TOP1 /// TOP1P1	topoisomerase (DNA) I /// topoisomerase (DNA) I pseudogene 1	0,000779	-1,95	0,00841	-1,79	0,796554	-1,09	0,189715	-1,28	0,02484	1,39	0,002316	1,52
11720970_at	7153	TOP2A	topoisomerase (DNA) II alpha 170kDa	0,001318	-2,16	0,01631	-1,89	0,748667	-1,14	0,000245	-2,46	0,151173	-1,30	0,482361	-1,14
11716247_s_at	7155	TOP2B	topoisomerase (DNA) II beta 180kDa	0,006038	-1,53	0,014227	-1,55	0,973715	1,01	0,000184	-1,86	0,138134	-1,20	0,096377	-1,21
11749531_x_at	7155	TOP2B	topoisomerase (DNA) II beta 180kDa	0,004025	-1,92	0,008176	-2,00	0,932425	1,04	0,000297	-2,37	0,357879	-1,18	0,228897	-1,23
11757323_x_at	7155	TOP2B	topoisomerase (DNA) II beta 180kDa	0,010942	-1,58	0,024297	-1,59	0,985079	1,01	0,000476	-1,94	0,164225	-1,22	0,129091	-1,23
11743824_s_at	11073	TOPBP1	topoisomerase (DNA) II binding protein 1	0,003659	-1,77	0,019781	-1,68	0,890772	-1,05	9,61E-05	-2,26	0,048683	-1,35	0,089329	-1,28
11732260_a_at	10210	TOPORS	topoisomerase I binding, arginine/serine-rich, E3 ubiquitin protein ligase	0,001127	-1,70	0,019654	-1,52	0,682132	-1,12	0,02184	-1,43	0,653836	1,06	0,14635	1,19
11721439_a_at	26092	TOR1AIP1	torsin A interacting protein 1	0,006976	-1,71	0,012936	-1,77	0,934763	1,04	0,348547	1,21	3,65E-06	2,15	1,59E-06	2,07
11721440_at	26092	TOR1AIP1	torsin A interacting protein 1	0,001222	-2,15	0,00456	-2,15	0,998261	1,00	0,479974	-1,18	0,000941	1,82	0,000342	1,82
11729643_s_at	7163	TPD52	tumor protein D52	0,000712	-1,66	0,005522	-1,59	0,877754	-1,04	5,29E-05	-1,87	0,146371	-1,18	0,277853	-1,13
11756600_a_at	7163	TPD52	tumor protein D52	0,000294	-1,85	0,002348	-1,79	0,91818	-1,03	0,001456	-1,68	0,631927	1,07	0,439082	1,10
11727482_a_at	7175	TPR	translocated promoter region, nuclear basket protein	0,000117	-2,75	0,000676	-2,71	0,977959	-1,02	0,000854	-2,29	0,382351	1,18	0,321042	1,20
11727483_a_at	7175	TPR	translocated promoter region, nuclear basket protein	0,00221	-1,72	0,012164	-1,65	0,909126	-1,04	0,00021	-1,98	0,187522	-1,20	0,296608	-1,15
11746133_a_at	29896	TRA2A	transformer 2 alpha homolog (Drosophila)	0,001222	-2,31	0,016961	-1,98	0,7285	-1,16	0,001884	-2,22	0,593405	-1,12	0,860229	1,04
11752349_a_at	60684	TRAPPC11	trafficking protein particle complex 11	0,002868	-1,71	0,015219	-1,64	0,905786	-1,04	0,034493	-1,45	0,397169	1,13	0,224247	1,18
11720257_a_at	122553	TRAPPC6B	trafficking protein particle complex 6B	0,002743	-1,87	0,043019	-1,60	0,667695	-1,17	0,080607	-1,43	0,492764	1,12	0,076892	1,31
11736267_at	22878	TRAPPC8	trafficking protein particle complex 8	0,000405	-1,63	0,000998	-1,69	0,907614	1,03	1,26E-05	-1,88	0,289808	-1,12	0,15078	-1,15
11736266_a_at	22878	TRAPPC8	trafficking protein particle complex 8	0,001039	-1,54	0,002061	-1,60	0,869597	1,04	0,000234	-1,64	0,851599	-1,02	0,553176	-1,06
11745633_a_at	22878	TRAPPC8	trafficking protein particle complex 8	0,001078	-2,08	0,001783	-2,26	0,832904	1,09	0,005567	-1,83	0,211752	1,24	0,453305	1,14
11739281_at	373	TRIM23	tripartite motif containing 23	0,000516	-1,96	0,008816	-1,75	0,714489	-1,12	0,009114	-1,62	0,618854	1,08	0,159865	1,21
11758692_s_at	8805	TRIM24	tripartite motif containing 24	0,000623	-2,02	0,015305	-1,72	0,635606	-1,17	0,01656	-1,59	0,645848	1,08	0,103751	1,27
11729816_x_at	8805	TRIM24	tripartite motif containing 24	0,001607	-1,85	0,026185	-1,62	0,680674	-1,15	0,014489	-1,59	0,927555	1,02	0,302596	1,16

11749468_a_at	8805	TRIM24	tripartite motif containing 24	0,004471	-1,90	0,043493	-1,68	0,747948	-1,14	0,052353	-1,54	0,66584	1,09	0,221505	1,23
11720259_a_at	51592	TRIM33	tripartite motif containing 33	0,006463	-1,54	0,023799	-1,51	0,946248	-1,02	0,001357	-1,69	0,370246	-1,12	0,469312	-1,10
11742759_a_at	9321	TRIP11	thyroid hormone receptor interactor 11	0,003104	-1,57	0,008301	-1,59	0,974981	1,01	0,008426	-1,49	0,616881	1,07	0,679388	1,05
11746421_a_at	54482	TRMT13	tRNA methyltransferase 13 homolog (S. cerevisiae)	0,001609	-2,11	0,025065	-1,80	0,690826	-1,17	0,000469	-2,33	0,155709	-1,29	0,610554	-1,10
11722902_a_at	54482	TRMT13	tRNA methyltransferase 13 homolog (S. cerevisiae)	0,004471	-1,76	0,012605	-1,77	0,993976	1,00	0,064562	-1,44	0,187302	1,23	0,175987	1,22
11728213_at	81627	TRMT1L	tRNA methyltransferase 1 homolog (S. cerevisiae)-like	0,001492	-1,85	0,014006	-1,70	0,805537	-1,09	0,961386	-1,01	0,000467	1,68	1,54E-05	1,83
11728212_at	81627	TRMT1L	tRNA methyltransferase 1 homolog (S. cerevisiae)-like	0,000477	-2,20	0,010696	-1,87	0,659993	-1,18	0,001422	-2,01	0,700323	-1,07	0,601826	1,10
11758288_s_at	81627	TRMT1L	tRNA methyltransferase 1 homolog (S. cerevisiae)-like	0,000712	-2,02	0,017575	-1,71	0,628221	-1,18	0,023568	-1,56	0,578793	1,10	0,079079	1,29
11736177_s_at	51095	TRNT1	tRNA nucleotidyl transferase, CCA-adding, 1	0,000712	-2,05	0,013397	-1,77	0,68488	-1,15	0,013984	-1,64	0,645685	1,08	0,144918	1,25
11736176_a_at	51095	TRNT1	tRNA nucleotidyl transferase, CCA-adding, 1	0,001457	-2,01	0,034786	-1,67	0,607616	-1,21	0,063819	-1,48	0,49768	1,13	0,050004	1,36
11723121_at	54822	TRPM7	transient receptor potential cation channel, subfamily M, member 7	0,001849	-1,80	0,013419	-1,69	0,854454	-1,07	0,001067	-1,87	0,517264	-1,10	0,833748	-1,04
11719573_a_at	142940	TRUB1	TruB pseudouridine (psi) synthase family member 1	0,001239	-1,81	0,016469	-1,63	0,736084	-1,11	0,102358	-1,34	0,151675	1,22	0,018505	1,36
11727000_x_at	7257	TSNAX	translin-associated factor X	8,91E-06	-3,27	0,000135	-3,08	0,896929	-1,06	6,36E-05	-2,67	0,428992	1,16	0,237935	1,23
11736114_a_at	151613	TTC14	tetratricopeptide repeat domain 14	0,000708	-2,11	0,01529	-1,79	0,655177	-1,18	0,000176	-2,31	0,121635	-1,29	0,595146	-1,10
11715639_a_at	7267 /// 286495	TTC3 /// TTC3P1	tetratricopeptide repeat domain 3 /// tetratricopeptide repeat domain 3 pseudogene 1	0,0002	-2,06	0,001672	-1,98	0,913849	-1,04	2,18E-05	-2,31	0,271227	-1,17	0,404536	-1,13
11745438_x_at	7267 /// 286495	TTC3 /// TTC3P1	tetratricopeptide repeat domain 3 /// tetratricopeptide repeat domain 3 pseudogene 1	0,000361	-2,53	0,001521	-2,55	0,990808	1,01	0,00404	-2,03	0,238368	1,26	0,234361	1,25
11715641_a_at	7267 /// 286495	TTC3 /// TTC3P1	tetratricopeptide repeat domain 3 /// tetratricopeptide repeat domain 3 pseudogene 1	0,003096	-1,85	0,020032	-1,73	0,856699	-1,07	0,004947	-1,79	0,833445	-1,04	0,865012	1,03
11725094_s_at	23548	TTC33	tetratricopeptide repeat domain 33	0,004903	-1,79	0,035196	-1,64	0,812266	-1,09	0,064703	-1,46	0,497352	1,12	0,191684	1,23
11722191_at	9652	TTC37	tetratricopeptide repeat domain 37	0,000362	-2,01	0,001521	-2,02	0,990613	1,01	0,014358	-1,57	0,071458	1,29	0,061863	1,29
11747342_a_at	8458	TTF2	transcription termination factor, RNA polymerase II	0,000129	-1,91	0,002862	-1,74	0,723912	-1,10	0,027715	-1,40	0,073967	1,24	0,005588	1,36
11739204_a_at	150465	TTL	tubulin tyrosine ligase	0,003	-1,69	0,010696	-1,68	0,979328	-1,01	0,032797	-1,45	0,299334	1,16	0,245982	1,17
11747999_a_at	51174	TUBD1	tubulin, delta 1	0,000232	-1,67	0,006689	-1,51	0,63906	-1,11	0,00415	-1,46	0,769226	1,03	0,155497	1,15

11735121_at	27229	TUBGCP4	tubulin, gamma complex associated protein 4	0,000153	-1,71	0,006195	-1,52	0,587043	-1,13	0,002246	-1,50	0,93673	1,01	0,183032	1,14
11725397_at	221830	TWISTNB	TWIST neighbor	0,00225	-1,87	0,034381	-1,62	0,680135	-1,15	0,481724	-1,16	0,031871	1,40	0,001008	1,61
11719691_x_at	57045	TWSG1	twisted gastrulation BMP signaling modulator 1	0,00068	-2,18	0,026207	-1,74	0,545674	-1,26	0,034431	-1,58	0,612726	1,10	0,045113	1,38
11719690_at	57045	TWSG1	twisted gastrulation BMP signaling modulator 1	0,000914	-2,06	0,034005	-1,65	0,541354	-1,25	0,049898	-1,50	0,580717	1,10	0,038359	1,37
11759194_a_at	55787	TXLNG	taxilin gamma	3,96E-05	-2,09	0,000893	-1,91	0,754362	-1,09	0,071952	-1,33	0,0037	1,44	0,000116	1,57
11759193_a_at	55787	TXLNG	taxilin gamma	0,00214	-1,67	0,01872	-1,55	0,802713	-1,08	0,013577	-1,50	0,805608	1,04	0,398076	1,12
11723856_at	57544	TXNDC16	thioredoxin domain containing 16	0,002567	-1,65	0,021731	-1,53	0,798659	-1,08	0,012393	-1,51	0,902575	1,02	0,479472	1,10
11747485_a_at	23350	U2SURP	U2 snRNP-associated SURP domain containing	0,000472	-2,09	0,004068	-1,96	0,870676	-1,06	0,082363	-1,41	0,030713	1,40	0,005908	1,48
11749584_a_at	10054	UBA2	ubiquitin-like modifier activating enzyme 2	0,000629	-2,32	0,006203	-2,11	0,82393	-1,10	0,041618	-1,61	0,134631	1,32	0,0307	1,45
11717531_x_at	10054	UBA2	ubiquitin-like modifier activating enzyme 2	0,000401	-1,68	0,005022	-1,57	0,78349	-1,07	0,004354	-1,49	0,631834	1,06	0,240479	1,13
11724535_a_at	79876	UBA5	ubiquitin-like modifier activating enzyme 5	0,000404	-1,91	0,010345	-1,66	0,636834	-1,15	0,007179	-1,59	0,765335	1,05	0,152053	1,20
11740962_a_at	79876	UBA5	ubiquitin-like modifier activating enzyme 5	0,000477	-1,83	0,009233	-1,63	0,68719	-1,12	0,011998	-1,50	0,547482	1,08	0,10787	1,21
11740963_x_at	79876	UBA5	ubiquitin-like modifier activating enzyme 5	0,00065	-1,73	0,00859	-1,60	0,762154	-1,09	0,010792	-1,48	0,551217	1,08	0,171954	1,17
11724536_a_at	79876	UBA5	ubiquitin-like modifier activating enzyme 5	0,000949	-1,85	0,02749	-1,56	0,583609	-1,18	0,040172	-1,44	0,575372	1,09	0,05494	1,29
11730893_a_at	55236	UBA6	ubiquitin-like modifier activating enzyme 6	0,000906	-1,84	0,009505	-1,69	0,795625	-1,09	0,211855	-1,25	0,024787	1,36	0,002269	1,47
11730894_a_at	55236	UBA6	ubiquitin-like modifier activating enzyme 6	0,000256	-1,68	0,001568	-1,66	0,960128	-1,01	0,000519	-1,61	0,818774	1,03	0,714335	1,04
11758226_s_at	51465	UBE2J1	ubiquitin-conjugating enzyme E2, J1	0,001438	-1,98	0,002723	-2,12	0,865219	1,07	0,035491	-1,54	0,050866	1,37	0,108705	1,28
11728225_at	51465	UBE2J1	ubiquitin-conjugating enzyme E2, J1	0,028765	-1,34	0,007754	-1,53	0,579111	1,14	0,016065	-1,39	0,393027	1,10	0,765323	-1,04
11716199_x_at	92912	UBE2Q2	ubiquitin-conjugating enzyme E2Q family member 2	0,006701	-1,66	0,039165	-1,55	0,847336	-1,07	0,616219	1,11	0,000288	1,71	1,32E-05	1,83
11754112_a_at	7336	UBE2V2	ubiquitin-conjugating enzyme E2 variant 2	4,75E-05	-1,95	0,000394	-1,91	0,948456	-1,02	2,28E-06	-2,21	0,220129	-1,15	0,279758	-1,13
11717640_s_at	7337	UBE3A	ubiquitin protein ligase E3A	0,002798	-1,63	0,019748	-1,53	0,840988	-1,06	0,000874	-1,74	0,326661	-1,13	0,618841	-1,07
11732687_a_at	165324	UBXN2A	UBX domain protein 2A	0,00146	-1,77	0,030038	-1,54	0,63906	-1,15	0,500302	-1,13	0,023575	1,36	0,000406	1,56
11753513_a_at	165324	UBXN2A	UBX domain protein 2A	0,001101	-1,65	0,00905	-1,56	0,847103	-1,05	0,230172	-1,20	0,019683	1,31	0,002643	1,38
11749832_x_at	165324	UBXN2A	UBX domain protein 2A	0,002067	-1,72	0,015004	-1,61	0,849202	-1,06	0,447536	-1,14	0,010037	1,41	0,001118	1,50
11719878_at	137886	UBXN2B	UBX domain protein 2B	0,000469	-1,55	0,000855	-1,62	0,850436	1,04	0,019392	-1,32	0,024978	1,23	0,059171	1,18

11742836_a_at	23190	UBXN4	UBX domain protein 4	0,000272	-1,75	0,002796	-1,67	0,864903	-1,05	1,13E-06	-2,23	0,009952	-1,33	0,020868	-1,27
11751000_a_at	23190	UBXN4	UBX domain protein 4	0,00052	-2,20	0,004475	-2,05	0,87103	-1,07	0,038181	-1,56	0,096003	1,32	0,027085	1,41
11755910_a_at	26043	UBXN7	UBX domain protein 7	0,001566	-1,88	0,01554	-1,71	0,790075	-1,10	0,359825	-1,20	0,018211	1,43	0,001395	1,57
11755885_a_at	26043	UBXN7	UBX domain protein 7	0,00079	-1,78	0,01385	-1,59	0,696022	-1,12	0,001791	-1,69	0,665441	-1,06	0,714067	1,05
11751587_a_at	51377	UCHL5	ubiquitin carboxyl-terminal hydrolase L5	0,001234	-1,87	0,015451	-1,68	0,750014	-1,11	0,013899	-1,58	0,711818	1,06	0,251121	1,18
11751145_a_at	23376	UFL1	UFM1-specific ligase 1	0,000175	-2,46	0,006552	-2,01	0,594783	-1,22	0,00019	-2,40	0,318948	-1,19	0,905016	1,03
11743509_a_at	23376	UFL1	UFM1-specific ligase 1	0,000736	-2,04	0,017167	-1,73	0,638767	-1,18	0,000787	-2,01	0,34625	-1,16	0,955094	1,01
11743510_at	23376	UFL1	UFM1-specific ligase 1	0,006522	-1,82	0,047067	-1,64	0,797919	-1,11	0,001054	-2,11	0,150097	-1,28	0,394316	-1,16
11732156_a_at	51569	UFM1	ubiquitin-fold modifier 1	0,006508	-1,87	0,043212	-1,70	0,817294	-1,10	0,668329	1,11	0,000493	1,89	1,85E-05	2,09
11757182_a_at	127933	UHMK1	U2AF homology motif (UHM) kinase 1	0,003021	-1,90	0,027394	-1,71	0,780248	-1,11	0,86764	-1,04	0,003094	1,64	0,000119	1,82
11743930_at	23074	UHRF1BP1L	UHRF1 binding protein 1-like	0,01238	-1,59	0,025713	-1,61	0,978584	1,01	0,097383	-1,36	0,263203	1,18	0,283286	1,17
11745896_a_at	26019	UPF2	UPF2 regulator of nonsense transcripts homolog (yeast)	0,000631	-1,91	0,003001	-1,90	0,984877	-1,01	0,000193	-2,04	0,630221	-1,08	0,66596	-1,07
11755113_a_at	26019	UPF2	UPF2 regulator of nonsense transcripts homolog (yeast)	0,001945	-1,78	0,004735	-1,83	0,942867	1,03	0,000446	-1,95	0,678349	-1,07	0,532031	-1,10
11751257_a_at	65109	UPF3B	UPF3 regulator of nonsense transcripts homolog B (yeast)	1,33E-05	-2,17	0,000292	-2,01	0,792928	-1,08	2,87E-06	-2,27	0,341563	-1,13	0,733474	-1,05
11723549_a_at	65109	UPF3B	UPF3 regulator of nonsense transcripts homolog B (yeast)	0,00056	-2,08	0,004293	-1,97	0,891787	-1,06	0,001212	-1,96	0,968615	1,01	0,721211	1,06
11716441_at	8615	USO1	USO1 vesicle transport factor	8,97E-05	-2,87	0,001755	-2,52	0,764957	-1,14	0,07268	-1,55	0,009932	1,63	0,000481	1,86
11716440_s_at	8615	USO1	USO1 vesicle transport factor	9,02E-05	-2,17	0,00049	-2,16	0,995893	-1,00	0,004389	-1,68	0,068967	1,29	0,051901	1,29
11727789_a_at	7398	USP1	ubiquitin specific peptidase 1	0,000811	-2,05	0,005247	-1,96	0,90988	-1,05	0,005324	-1,78	0,583472	1,10	0,379692	1,15
11732118_a_at	9100	USP10	ubiquitin specific peptidase 10	0,004509	-1,98	0,026037	-1,83	0,865219	-1,08	0,100016	-1,48	0,260101	1,24	0,101214	1,34
11735996_x_at	9097	USP14	ubiquitin specific peptidase 14 (tRNA-guanine transglycosylase)	2,14E-05	-2,23	0,003328	-1,78	0,457902	-1,25	2,94E-05	-2,13	0,166872	-1,20	0,743563	1,05
11725414_a_at	10600	USP16	ubiquitin specific peptidase 16	0,000131	-2,18	0,005083	-1,84	0,597412	-1,18	0,000854	-1,90	0,849883	-1,03	0,346487	1,15
11725415_a_at	10600	USP16	ubiquitin specific peptidase 16	0,006617	-1,58	0,033604	-1,50	0,880988	-1,05	0,007496	-1,58	0,752219	-1,05	0,983581	1,00
11744377_s_at	23358	USP24	ubiquitin specific peptidase 24	0,000574	-1,80	0,003156	-1,78	0,964594	-1,02	0,031679	-1,41	0,07291	1,25	0,04101	1,28
11755051_a_at	29761	USP25	ubiquitin specific peptidase 25	0,040702	-1,41	0,032554	-1,51	0,812513	1,08	0,838855	1,04	0,000763	1,57	0,00207	1,46
11730069_a_at	23032	USP33	ubiquitin specific peptidase 33	0,00056	-1,81	0,004718	-1,72	0,872047	-1,05	0,687582	1,07	4,73E-06	1,84	1,75E-07	1,94

11723681_a_at	9736	USP34	ubiquitin specific peptidase 34	0,004904	-1,78	0,030079	-1,66	0,849178	-1,08	0,000105	-2,35	0,028601	-1,42	0,068588	-1,32
11754852_a_at	57695	USP37	ubiquitin specific peptidase 37	0,0005	-1,96	0,005937	-1,80	0,793493	-1,09	0,002167	-1,77	0,927484	1,02	0,489532	1,11
11743616_a_at	55031	USP47	ubiquitin specific peptidase 47	0,000306	-1,65	0,002796	-1,59	0,887247	-1,04	3,74E-07	-2,15	0,002798	-1,35	0,004357	-1,31
11753990_a_at	55031	USP47	ubiquitin specific peptidase 47	2,05E-05	-3,86	0,000496	-3,26	0,728007	-1,18	0,000284	-2,87	0,580751	1,14	0,154765	1,34
11724569_a_at	84196	USP48	ubiquitin specific peptidase 48	0,000122	-2,21	0,002379	-2,00	0,758577	-1,11	0,027365	-1,51	0,057623	1,32	0,005259	1,46
11726705_a_at	9101	USP8	ubiquitin specific peptidase 8	0,000782	-2,01	0,004501	-1,95	0,938301	-1,03	0,557558	-1,13	0,000548	1,72	7,85E-05	1,78
11724334_a_at	9101	USP8	ubiquitin specific peptidase 8	0,000238	-1,81	0,003631	-1,68	0,776952	-1,08	0,077842	-1,30	0,02794	1,29	0,002244	1,39
11731501_at	84294	UTP23	UTP23, small subunit (SSU) processome component, homolog (yeast)	4,83E-05	-2,16	0,000939	-1,98	0,780187	-1,09	0,000127	-2,01	0,941188	-1,01	0,602594	1,08
11747291_a_at	55813	UTP6	UTP6, small subunit (SSU) processome component, homolog (yeast)	0,015257	-1,62	0,03553	-1,62	0,998261	-1,00	0,223645	-1,28	0,137692	1,26	0,117786	1,26
11760288_s_at	8674	VAMP4	vesicle-associated membrane protein 4	0,001667	-1,59	0,010739	-1,53	0,887901	-1,04	0,002563	-1,56	0,889293	-1,02	0,867063	1,02
11763324_a_at	8674	VAMP4	vesicle-associated membrane protein 4	0,004635	-1,81	0,047682	-1,60	0,735255	-1,13	0,018496	-1,64	0,913952	-1,02	0,544426	1,11
11752069_a_at	80124	VCPIP1	valosin containing protein (p97)/p47 complex interacting protein 1	2,11E-05	-2,14	0,000204	-2,11	0,957642	-1,02	0,026041	-1,41	0,00119	1,49	0,000249	1,52
11739800_a_at	80124	VCPIP1	valosin containing protein (p97)/p47 complex interacting protein 1	6,81E-05	-2,07	0,000443	-2,06	0,986271	-1,01	0,013973	-1,50	0,014014	1,37	0,007055	1,38
11739801_x_at	80124	VCPIP1	valosin containing protein (p97)/p47 complex interacting protein 1	4,75E-05	-1,82	0,000391	-1,79	0,951365	-1,02	0,006249	-1,44	0,032818	1,24	0,01373	1,27
11744674_a_at	55591	VEZT	vezatin, adherens junctions transmembrane protein	0,001097	-1,92	0,015752	-1,71	0,722551	-1,13	4,54E-05	-2,35	0,031329	-1,38	0,161829	-1,23
11745241_a_at	55591	VEZT	vezatin, adherens junctions transmembrane protein	0,001215	-1,80	0,01611	-1,62	0,736602	-1,11	5,01E-05	-2,17	0,033784	-1,34	0,158305	-1,21
11722570_a_at	55591	VEZT	vezatin, adherens junctions transmembrane protein	0,003193	-1,72	0,04239	-1,52	0,696068	-1,13	0,000147	-2,09	0,02452	-1,37	0,155997	-1,21
11759041_x_at	154807	VKORC1L1	vitamin K epoxide reductase complex, subunit 1-like 1	0,000289	-1,88	0,011598	-1,60	0,569366	-1,17	3,69E-05	-2,08	0,040086	-1,30	0,439134	-1,10
11759043_at	154807	VKORC1L1	vitamin K epoxide reductase complex, subunit 1-like 1	0,003898	-1,63	0,02039	-1,56	0,8947	-1,05	0,525095	-1,12	0,011079	1,39	0,001974	1,46
11743391_a_at	55737	VPS35	vacuolar protein sorting 35 homolog (S. cerevisiae)	0,006921	-1,79	0,013275	-1,85	0,940338	1,03	0,034214	-1,57	0,35333	1,17	0,461366	1,13
11739263_x_at	9525	VPS4B	vacuolar protein sorting 4 homolog B (S. cerevisiae)	0,001735	-1,81	0,017366	-1,65	0,783237	-1,10	0,730649	-1,07	0,002783	1,54	0,000106	1,69

11739262_at	9525	VPS4B	vacuolar protein sorting 4 homolog B (S. cerevisiae)	0,000454	-1,59	0,001453	-1,61	0,948456	1,02	0,001088	-1,52	0,551118	1,06	0,671936	1,05
11715225_s_at	51542	VPS54	vacuolar protein sorting 54 homolog (S. cerevisiae)	0,000304	-1,79	0,004887	-1,64	0,745982	-1,09	0,001025	-1,66	0,928375	-1,01	0,552842	1,08
11720041_x_at	51534	VTA1	vesicle (multivesicular body) trafficking 1	0,007121	-1,59	0,020725	-1,58	0,984645	-1,01	0,892021	-1,03	0,001604	1,54	0,000505	1,55
11720039_a_at	51534	VTA1	vesicle (multivesicular body) trafficking 1	0,008043	-1,55	0,023723	-1,53	0,978705	-1,01	0,917222	-1,02	0,001796	1,50	0,000527	1,51
11730898_a_at	23063	WAPAL	wings apart-like homolog (Drosophila)	0,000577	-2,11	0,00565	-1,95	0,834009	-1,08	0,004693	-1,80	0,654106	1,08	0,322262	1,17
11750329_x_at	23063	WAPAL	wings apart-like homolog (Drosophila)	0,002534	-1,80	0,01532	-1,70	0,881995	-1,06	0,037648	-1,48	0,375024	1,15	0,182152	1,21
11716244_s_at	10163	WASF2	WAS protein family, member 2	0,01813	-1,72	0,034088	-1,76	0,970852	1,02	0,021393	-1,71	0,900631	1,03	0,974347	1,01
11754884_s_at	57590	WDFY1	WD repeat and FYVE domain containing 1	0,004532	-1,71	0,019868	-1,65	0,926048	-1,04	0,504143	1,14	3,32E-05	1,88	2,63E-06	1,95
11749440_a_at	55255	WDR41	WD repeat domain 41	0,000409	-1,71	0,003211	-1,65	0,900487	-1,04	0,112327	-1,25	0,012513	1,32	0,002442	1,37
11747166_a_at	55255	WDR41	WD repeat domain 41	0,001026	-1,59	0,003415	-1,60	0,977584	1,01	0,191993	-1,19	0,005451	1,34	0,003607	1,33
11749354_x_at	55255	WDR41	WD repeat domain 41	0,023349	-1,48	0,040605	-1,51	0,964924	1,02	0,67005	-1,08	0,016498	1,39	0,015053	1,37
11717308_a_at	84128	WDR75	WD repeat domain 75	0,001653	-1,86	0,018231	-1,68	0,764273	-1,11	0,00056	-2,00	0,257116	-1,19	0,660787	-1,07
11717309_x_at	84128	WDR75	WD repeat domain 75	0,003227	-1,68	0,034911	-1,52	0,736924	-1,11	0,000711	-1,85	0,151622	-1,22	0,504117	-1,10
11755442_a_at	54904	WHSC1L1	Wolf-Hirschhorn syndrome candidate 1-like 1	0,002309	-1,83	0,02029	-1,67	0,796362	-1,09	0,143801	-1,33	0,128583	1,26	0,02348	1,38
11718569_a_at	7456	WIPF1	WAS/WASL interacting protein family, member 1	0,019877	-1,53	0,044431	-1,53	0,998196	-1,00	0,961386	1,01	0,002852	1,54	0,001212	1,54
11741202_x_at	54739	XAF1	XIAP associated factor 1	0,010158	-1,94	0,026801	-1,92	0,989279	-1,01	0,177337	-1,42	0,136457	1,35	0,107581	1,36
11726770_x_at	54739	XAF1	XIAP associated factor 1	0,010851	-1,89	0,032115	-1,85	0,968241	-1,02	0,209098	-1,37	0,134144	1,34	0,088856	1,37
11726769_a_at	54739	XAF1	XIAP associated factor 1	0,009984	-2,03	0,035945	-1,93	0,934631	-1,05	0,175568	-1,45	0,193415	1,33	0,107324	1,39
11757857_s_at	7503	XIST	X inactive specific transcript (non-protein coding)	0,208641	-4,45	0,041971	-15,87	0,539318	3,57	0,378097	-2,95	0,071183	5,37	0,686508	1,51
11716623_a_at	7514	XPO1	exportin 1	0,004103	-1,87	0,015748	-1,83	0,957321	-1,03	0,212772	-1,31	0,051168	1,39	0,024872	1,43
11748586_x_at	7514	XPO1	exportin 1	0,003795	-1,76	0,01289	-1,75	0,981036	-1,01	0,168707	-1,31	0,055988	1,34	0,035077	1,35
11748585_a_at	7514	XPO1	exportin 1	0,006993	-1,63	0,021328	-1,61	0,978096	-1,01	0,226069	-1,25	0,071183	1,29	0,045276	1,31
11716624_s_at	7514	XPO1	exportin 1	0,004799	-1,65	0,03333	-1,54	0,821084	-1,08	0,00656	-1,63	0,710064	-1,06	0,923444	1,02
11743781_a_at	54464	XRN1	5'-3' exoribonuclease 1	0,006246	-1,69	0,022708	-1,65	0,948692	-1,03	0,009304	-1,65	0,987536	-1,00	0,895243	1,02
11753770_a_at	22803	XRN2	5'-3' exoribonuclease 2	0,004628	-2,20	0,049219	-1,86	0,728257	-1,18	0,00308	-2,31	0,334962	-1,24	0,855412	-1,05
11743117_a_at	22803	XRN2	5'-3' exoribonuclease 2	0,004134	-1,99	0,022509	-1,86	0,885369	-1,07	0,016532	-1,77	0,816617	1,05	0,547049	1,12

11720454_a_at	10730	YME1L1	YME1-like 1 ATPase	1,98E-05	-2,19	0,000204	-2,16	0,96775	-1,01	0,045888	-1,37	0,000348	1,57	6,71E-05	1,60
11720453_x_at	10730	YME1L1	YME1-like 1 ATPase	5,35E-06	-2,79	0,00016	-2,52	0,765441	-1,11	0,001795	-1,83	0,029018	1,38	0,002118	1,52
11719826_x_at	51776	ZAK	sterile alpha motif and leucine zipper containing kinase AZK	3,62E-06	-3,18	3,74E-05	-3,20	0,989279	1,01	5,45E-09	-4,50	0,0344	-1,41	0,021303	-1,42
11719824_at	51776	ZAK	sterile alpha motif and leucine zipper containing kinase AZK	0,002641	-1,66	0,005845	-1,71	0,937334	1,03	2,06E-06	-2,45	0,005174	-1,44	0,001169	-1,48
11749191_a_at	51776	ZAK	sterile alpha motif and leucine zipper containing kinase AZK	0,000361	-1,70	0,001646	-1,70	0,996345	-1,00	1,68E-06	-2,14	0,032306	-1,26	0,023254	-1,26
11745435_a_at	22890	ZBTB1	zinc finger and BTB domain containing 1	0,067803	-1,40	0,034176	-1,57	0,721165	1,12	0,85234	-1,04	0,005004	1,51	0,031258	1,34
11723141_at	27107	ZBTB11	zinc finger and BTB domain containing 11	0,01437	-1,60	0,040668	-1,58	0,96775	-1,02	0,52003	1,14	0,000168	1,80	2,89E-05	1,83
11723140_at	27107	ZBTB11	zinc finger and BTB domain containing 11	0,005832	-1,72	0,011854	-1,77	0,946515	1,03	0,11974	-1,36	0,082401	1,31	0,104232	1,27
11728734_at	253461	ZBTB38	zinc finger and BTB domain containing 38	0,001583	-1,63	0,00323	-1,70	0,891968	1,04	0,439534	1,13	3,32E-07	1,92	2,07E-07	1,84
11728733_at	253461	ZBTB38	zinc finger and BTB domain containing 38	0,015566	-1,52	0,034284	-1,53	0,993738	1,00	0,881266	-1,03	0,004287	1,48	0,002213	1,48
11748686_a_at	55854	ZC3H15	zinc finger CCCH-type containing 15	0,000752	-2,04	0,003727	-2,00	0,969636	-1,02	0,019348	-1,60	0,155608	1,25	0,107094	1,27
11752239_a_at	55854	ZC3H15	zinc finger CCCH-type containing 15	0,001076	-2,07	0,005236	-2,01	0,955654	-1,03	0,015268	-1,68	0,286303	1,20	0,199796	1,23
11720155_a_at	29066	ZC3H7A	zinc finger CCCH-type containing 7A	0,006242	-1,78	0,04413	-1,62	0,803163	-1,10	0,081196	-1,44	0,499639	1,12	0,185219	1,24
11726463_a_at	54819	ZCCHC10	zinc finger, CCHC domain containing 10	0,000141	-2,37	0,005022	-1,98	0,6122	-1,20	2,31E-05	-2,62	0,079039	-1,33	0,540036	-1,11
11749394_a_at	54819	ZCCHC10	zinc finger, CCHC domain containing 10	0,000488	-1,70	0,003134	-1,66	0,937334	-1,02	0,011919	-1,43	0,196638	1,16	0,11159	1,18
11749395_x_at	54819	ZCCHC10	zinc finger, CCHC domain containing 10	0,001	-1,54	0,005343	-1,51	0,939918	-1,02	0,009614	-1,39	0,410383	1,09	0,282369	1,11
11750669_x_at	23318	ZCCHC11	zinc finger, CCHC domain containing 11	0,002582	-2,15	0,033725	-1,83	0,707267	-1,18	0,029023	-1,72	0,783039	1,06	0,240651	1,25
11744628_x_at	84186	ZCCHC7	zinc finger, CCHC domain containing 7	0,050822	-1,39	0,014984	-1,61	0,604554	1,16	0,001495	-1,75	0,57481	-1,08	0,066305	-1,26
11721520_at	23390	ZDHHC17	zinc finger, DHHC-type containing 17	0,002596	-1,73	0,011331	-1,69	0,947699	-1,03	0,000119	-2,09	0,124705	-1,24	0,159482	-1,21
11721528_at	51201	ZDHHC2	zinc finger, DHHC-type containing 2	0,013759	-1,78	0,025561	-1,82	0,961678	1,03	0,502707	1,18	5,59E-05	2,15	2,31E-05	2,10
11747837_a_at	79752	ZFAND1	zinc finger, AN1-type domain 1	0,004195	-1,63	0,024944	-1,54	0,863683	-1,06	0,357002	-1,17	0,038502	1,31	0,007495	1,39
11723758_a_at	51663	ZFR	zinc finger RNA binding protein	0,009472	-1,66	0,041511	-1,57	0,894744	-1,05	0,835177	-1,05	0,007659	1,50	0,001227	1,58
11723756_at	51663	ZFR	zinc finger RNA binding protein	0,001244	-1,93	0,009069	-1,81	0,871175	-1,06	0,009582	-1,67	0,615421	1,09	0,345037	1,16
11741640_a_at	7543	ZFX	zinc finger protein, X-linked	0,000649	-2,20	0,002246	-2,25	0,961663	1,02	0,000579	-2,20	0,909513	1,02	0,997999	-1,00

11732214_at	7586	ZKSCAN1	zinc finger with KRAB and SCAN domains 1	8,91E-06	-3,47	0,000266	-3,02	0,737741	-1,15	8,10E-05	-2,76	0,650716	1,10	0,201698	1,26
11732213_x_at	7586	ZKSCAN1	zinc finger with KRAB and SCAN domains 1	3,20E-05	-2,81	0,000546	-2,55	0,809316	-1,10	2,46E-05	-2,77	0,65618	-1,09	0,952519	1,01
11732212_at	7586	ZKSCAN1	zinc finger with KRAB and SCAN domains 1	0,000909	-2,09	0,004466	-2,04	0,962945	-1,02	0,000648	-2,12	0,829019	-1,04	0,926764	-1,02
11752025_s_at	7750	ZMYM2	zinc finger, MYM-type 2	0,005335	-1,78	0,044884	-1,60	0,772208	-1,11	0,000469	-2,14	0,07547	-1,33	0,255909	-1,20
11716677_x_at	64397	ZNF106	zinc finger protein 106	0,000701	-1,79	0,003452	-1,77	0,971074	-1,01	2,42E-05	-2,14	0,136457	-1,21	0,149398	-1,19
11719616_x_at	7559	ZNF12	zinc finger protein 12	0,001754	-1,81	0,025625	-1,60	0,70007	-1,13	0,001118	-1,86	0,302348	-1,16	0,867234	-1,03
11752335_x_at	7559	ZNF12	zinc finger protein 12	0,002734	-1,66	0,019644	-1,56	0,837969	-1,07	0,005124	-1,60	0,855549	-1,03	0,808293	1,04
11737217_s_at	7678	ZNF124	zinc finger protein 124	0,029752	-1,55	0,038485	-1,62	0,912502	1,05	0,05786	-1,47	0,575217	1,10	0,779511	1,05
11743851_a_at	7705	ZNF146	zinc finger protein 146	0,00019	-2,39	0,00427	-2,07	0,70007	-1,16	0,00248	-1,94	0,739438	1,06	0,205522	1,23
200050_PM_at	7705	ZNF146	zinc finger protein 146	0,000733	-2,00	0,013957	-1,74	0,682423	-1,15	0,001217	-1,92	0,54271	-1,10	0,816809	1,04
11741070_a_at	7705	ZNF146	zinc finger protein 146	0,000802	-2,21	0,012746	-1,91	0,714509	-1,16	0,001257	-2,12	0,587566	-1,11	0,837546	1,04
11745436_a_at	7707	ZNF148	zinc finger protein 148	2,75E-05	-2,05	0,000274	-2,01	0,946785	-1,02	2,77E-06	-2,24	0,377953	-1,11	0,477201	-1,09
11754166_a_at	7707	ZNF148	zinc finger protein 148	0,000222	-1,87	0,00228	-1,78	0,878994	-1,05	9,11E-06	-2,17	0,103216	-1,22	0,200095	-1,16
11728314_at	7707	ZNF148	zinc finger protein 148	0,000862	-1,96	0,041333	-1,57	0,507654	-1,25	2,88E-05	-2,43	0,003736	-1,55	0,141476	-1,24
11728315_at	7707	ZNF148	zinc finger protein 148	0,00054	-1,74	0,00488	-1,65	0,856699	-1,05	4,92E-05	-1,95	0,160977	-1,18	0,328442	-1,12
11731710_x_at	90338	ZNF160	zinc finger protein 160	0,001195	-1,78	0,032546	-1,52	0,590363	-1,17	3,28E-05	-2,18	0,006747	-1,44	0,110046	-1,23
11728483_at	339318	ZNF181	zinc finger protein 181	0,000361	-2,17	0,009139	-1,84	0,64168	-1,18	0,001816	-1,91	0,842559	-1,04	0,428316	1,14
11728482_a_at	55900 /// 339318	ZNF181 /// ZNF302	zinc finger protein 181 /// zinc finger protein 302	0,003733	-1,84	0,022451	-1,72	0,864457	-1,07	0,008333	-1,74	0,957733	-1,01	0,749598	1,06
11728481_a_at	55900 /// 339318 /// 100129800	ZNF181 /// ZNF302 /// ZNF807	zinc finger protein 181 /// zinc finger protein 302 /// zinc finger protein 807	0,001254	-2,15	0,011352	-1,95	0,82164	-1,10	0,013356	-1,76	0,594093	1,11	0,264628	1,22
11721059_a_at	7743	ZNF189	zinc finger protein 189	0,003472	-1,83	0,027189	-1,67	0,805169	-1,09	0,498348	-1,16	0,020517	1,45	0,001925	1,58
11744992_a_at	7743	ZNF189	zinc finger protein 189	0,002992	-1,81	0,019489	-1,69	0,855918	-1,07	0,39672	-1,19	0,021013	1,42	0,003138	1,52
11739919_a_at	7748	ZNF195	zinc finger protein 195	0,002308	-1,87	0,017092	-1,73	0,838203	-1,08	0,001555	-1,92	0,531313	-1,11	0,879887	-1,03
11742100_x_at	7748	ZNF195	zinc finger protein 195	0,005024	-1,57	0,024361	-1,51	0,900353	-1,04	0,000987	-1,73	0,28944	-1,15	0,455437	-1,10
11755401_a_at	7748	ZNF195	zinc finger protein 195	0,00308	-1,82	0,02096	-1,69	0,844398	-1,08	0,002005	-1,88	0,520982	-1,11	0,857254	-1,03
11755402_x_at	7748	ZNF195	zinc finger protein 195	0,00341	-1,85	0,018955	-1,75	0,887472	-1,06	0,002446	-1,90	0,626716	-1,09	0,888547	-1,03
11739920_x_at	7748	ZNF195	zinc finger protein 195	0,00392	-1,76	0,03558	-1,59	0,769562	-1,11	0,002231	-1,84	0,360284	-1,15	0,805126	-1,04

11743147_at	7570	ZNF22	zinc finger protein 22	0,007909	-1,69	0,031209	-1,62	0,926108	-1,04	7,34E-05	-2,33	0,018515	-1,44	0,024565	-1,38
11724695_a_at	7770	ZNF227	zinc finger protein 227	0,000541	-1,96	0,02587	-1,59	0,520962	-1,23	0,01714	-1,55	0,864712	1,03	0,085328	1,26
11723261_a_at	7572	ZNF24	zinc finger protein 24	0,001557	-1,70	0,012574	-1,60	0,837969	-1,06	1,25E-05	-2,21	0,010669	-1,38	0,027479	-1,30
11727523_x_at	10308	ZNF267	zinc finger protein 267	0,002045	-2,11	0,023782	-1,84	0,740099	-1,15	0,711332	1,10	0,000188	2,03	2,55E-06	2,32
11738623_s_at	10795	ZNF268	zinc finger protein 268	0,001058	-1,73	0,017844	-1,55	0,690068	-1,12	4,51E-05	-2,05	0,025476	-1,32	0,166782	-1,18
11750557_x_at	10778	ZNF271	zinc finger protein 271	5,98E-05	-2,22	0,00049	-2,15	0,939069	-1,03	6,56E-06	-2,47	0,337058	-1,15	0,443161	-1,11
11744580_x_at	10778	ZNF271	zinc finger protein 271	0,000107	-2,06	0,000325	-2,14	0,909898	1,04	2,41E-05	-2,19	0,864519	-1,03	0,646079	-1,07
11750300_a_at	11179	ZNF277	zinc finger protein 277	0,000996	-2,03	0,012135	-1,82	0,762331	-1,12	0,000152	-2,31	0,141954	-1,27	0,438903	-1,14
11743670_s_at	23036	ZNF292	zinc finger protein 292	0,00329	-2,03	0,039889	-1,74	0,714509	-1,16	0,000132	-2,65	0,024062	-1,52	0,13717	-1,30
11718789_x_at	55900	ZNF302	zinc finger protein 302	0,004085	-1,78	0,019773	-1,70	0,910902	-1,05	0,014308	-1,63	0,809306	1,04	0,592522	1,09
11750396_x_at	55900	ZNF302	zinc finger protein 302	0,009204	-1,56	0,027256	-1,54	0,973128	-1,01	0,085933	-1,34	0,314612	1,15	0,249413	1,16
11755529_s_at	79692	ZNF322	zinc finger protein 322	0,003021	-1,72	0,020853	-1,61	0,840988	-1,07	0,099861	-1,34	0,206614	1,20	0,062574	1,28
11728270_a_at	27309	ZNF330	zinc finger protein 330	0,000147	-1,68	0,002923	-1,56	0,742062	-1,07	0,003029	-1,46	0,5004	1,07	0,130983	1,15
11751515_a_at	27309	ZNF330	zinc finger protein 330	0,000364	-1,76	0,003326	-1,67	0,865219	-1,05	0,006788	-1,49	0,337565	1,12	0,14489	1,18
11732343_at	7581	ZNF33A	zinc finger protein 33A	6,23E-06	-2,43	0,000204	-2,21	0,74273	-1,10	2,36E-05	-2,16	0,884325	1,02	0,366869	1,12
11732346_at	7581	ZNF33A	zinc finger protein 33A	0,001917	-1,92	0,03773	-1,62	0,636182	-1,18	0,578859	-1,13	0,022768	1,44	0,000374	1,70
11725475_at	6940	ZNF354A	zinc finger protein 354A	0,000639	-1,59	0,002848	-1,59	0,997281	-1,00	0,004513	-1,45	0,380733	1,10	0,358689	1,10
11744188_at	147923	ZNF420	zinc finger protein 420	0,002505	-1,90	0,012218	-1,82	0,927899	-1,04	0,000796	-2,06	0,470822	-1,13	0,629252	-1,09
11740148_x_at	353088	ZNF429	zinc finger protein 429	7,73E-05	-2,79	0,0023	-2,38	0,697801	-1,17	0,000201	-2,53	0,76619	-1,06	0,616111	1,10
11736804_at	7594 /// 100287226	ZNF43 /// ZNF729	zinc finger protein 43 /// zinc finger protein 729	0,001491	-1,97	0,02397	-1,70	0,686786	-1,16	1,76E-06	-3,11	0,000275	-1,82	0,002467	-1,58
11736805_x_at	7594 /// 100287226	ZNF43 /// ZNF729	zinc finger protein 43 /// zinc finger protein 729	0,00294	-1,81	0,031209	-1,62	0,745768	-1,12	2,68E-06	-2,85	0,000283	-1,76	0,001379	-1,58
11736436_x_at	80264	ZNF430	zinc finger protein 430	0,000472	-1,83	0,003146	-1,77	0,930112	-1,03	7,27E-05	-2,00	0,351667	-1,13	0,47944	-1,10
11742195_s_at	26036	ZNF451	zinc finger protein 451	0,003853	-1,76	0,018789	-1,68	0,910902	-1,05	0,001494	-1,88	0,48146	-1,12	0,678194	-1,07
11733207_a_at	147657	ZNF480	zinc finger protein 480	0,000693	-1,93	0,008815	-1,75	0,76683	-1,10	0,001506	-1,83	0,800454	-1,04	0,724768	1,06
11746809_a_at	27300	ZNF544	zinc finger protein 544	0,001084	-1,68	0,01671	-1,52	0,709374	-1,11	0,001219	-1,67	0,470222	-1,10	0,951185	1,01
11735368_x_at	256051	ZNF549	zinc finger protein 549	0,000495	-1,69	0,012545	-1,50	0,635793	-1,12	0,00087	-1,63	0,485179	-1,08	0,780057	1,04
11720737_a_at	84527	ZNF559	zinc finger protein 559	0,005119	-1,75	0,016976	-1,73	0,974733	-1,01	0,002387	-1,86	0,667123	-1,08	0,732924	-1,06
11755313_x_at	84527	ZNF559	zinc finger protein 559	0,008671	-1,73	0,023158	-1,73	0,991386	-1,01	0,008686	-1,74	0,95716	-1,01	0,979022	-1,01
11740629_a_at	81856	ZNF611	zinc finger protein 611	0,00153	-1,57	0,002941	-1,64	0,876332	1,04	0,047378	-1,31	0,03968	1,25	0,080423	1,20
11726449_at	80110	ZNF614	zinc finger protein 614	0,010879	-1,59	0,046544	-1,51	0,894857	-1,05	0,002919	-1,74	0,340721	-1,15	0,534734	-1,10

11745586_x_at	27332 /// 100507113	ZNF638 /// ZNF638-IT1	zinc finger protein 638 /// ZNF638 intronic transcript 1 (non-protein coding)	0,000631	-2,27	0,004516	-2,15	0,903186	-1,06	6,41E-05	-2,68	0,219343	-1,25	0,351554	-1,18
11756880_x_at	27332 /// 100507113	ZNF638 /// ZNF638-IT1	zinc finger protein 638 /// ZNF638 intronic transcript 1 (non-protein coding)	0,001331	-2,16	0,007923	-2,05	0,912043	-1,05	0,000435	-2,36	0,462376	-1,15	0,6523	-1,09
11721559_s_at	27332 /// 100507113	ZNF638 /// ZNF638-IT1	zinc finger protein 638 /// ZNF638 intronic transcript 1 (non-protein coding)	0,006278	-1,92	0,045901	-1,72	0,796211	-1,12	0,000614	-2,35	0,095042	-1,36	0,275128	-1,22
11748201_a_at	84146	ZNF644	zinc finger protein 644	0,004241	-1,80	0,038101	-1,62	0,768994	-1,11	0,897372	-1,03	0,004748	1,57	0,000187	1,75
11729261_a_at	84146	ZNF644	zinc finger protein 644	0,000454	-2,10	0,004134	-1,96	0,859946	-1,07	0,064895	-1,44	0,044346	1,36	0,008794	1,46
11729259_a_at	84146	ZNF644	zinc finger protein 644	0,000467	-2,50	0,00323	-2,37	0,91847	-1,05	0,011996	-1,86	0,206821	1,28	0,105005	1,35
11758209_s_at	84146	ZNF644	zinc finger protein 644	0,000688	-2,15	0,004887	-2,04	0,898945	-1,06	0,00995	-1,74	0,368988	1,17	0,196512	1,23
11729260_a_at	84146	ZNF644	zinc finger protein 644	0,000907	-2,35	0,009339	-2,10	0,798515	-1,12	0,024523	-1,74	0,337257	1,21	0,098772	1,35
11729516_a_at	84146	ZNF644	zinc finger protein 644	0,000905	-1,80	0,022647	-1,55	0,6122	-1,16	0,031952	-1,44	0,577784	1,08	0,070725	1,25
11730998_at	55279	ZNF654	zinc finger protein 654	0,000207	-2,03	0,00118	-2,00	0,97721	-1,01	0,003316	-1,69	0,223959	1,18	0,173137	1,20
11741101_a_at	79027	ZNF655	zinc finger protein 655	0,00096	-2,40	0,009505	-2,14	0,805026	-1,12	0,363366	-1,27	0,007954	1,69	0,000538	1,89
11724414_a_at	7552	ZNF711	zinc finger protein 711	0,005535	-2,06	0,037723	-1,85	0,818275	-1,11	0,003248	-2,18	0,43909	-1,18	0,810005	-1,06
11723787_x_at	90321	ZNF766	zinc finger protein 766	0,000177	-2,14	0,013549	-1,70	0,485495	-1,26	9,40E-05	-2,20	0,080903	-1,29	0,886732	-1,02
11739307_s_at	54989	ZNF770	zinc finger protein 770	0,000351	-2,13	0,005167	-1,91	0,751938	-1,12	0,000195	-2,18	0,402744	-1,14	0,896265	-1,02
11736418_a_at	168850	ZNF800	zinc finger protein 800	0,000495	-2,46	0,007042	-2,15	0,752167	-1,14	0,956151	1,02	6,74E-05	2,18	8,96E-07	2,50
11722324_a_at	7637	ZNF84	zinc finger protein 84	0,002248	-1,91	0,029405	-1,67	0,713981	-1,14	0,001456	-1,97	0,320537	-1,18	0,865462	-1,03
11731622_x_at	7644	ZNF91	zinc finger protein 91	2,14E-05	-3,61	0,000325	-3,27	0,838792	-1,10	6,09E-07	-4,60	0,096236	-1,41	0,228063	-1,27
11751203_a_at	54680	ZNHIT6	zinc finger, HIT-type containing 6	0,007167	-1,68	0,029622	-1,62	0,919664	-1,04	0,836156	-1,05	0,00419	1,54	0,000745	1,61
11722645_s_at	54680	ZNHIT6	zinc finger, HIT-type containing 6	0,000715	-1,71	0,00295	-1,71	0,995739	1,00	0,035499	-1,37	0,058121	1,25	0,046342	1,25
11722644_at	54680	ZNHIT6	zinc finger, HIT-type containing 6	0,001638	-1,74	0,02271	-1,56	0,714509	-1,12	0,009571	-1,56	0,996436	-1,00	0,42234	1,12
11728651_a_at	9406	ZRANB2	zinc finger, RAN-binding domain containing 2	0,000173	-2,08	0,004675	-1,82	0,664728	-1,14	0,044181	-1,43	0,086217	1,27	0,003912	1,46
11728652_x_at	9406	ZRANB2	zinc finger, RAN-binding domain containing 2	0,004177	-2,22	0,043254	-1,89	0,737022	-1,18	0,070325	-1,64	0,547681	1,15	0,149117	1,35
11754687_a_at	55055	ZWILCH	zwilch kinetochore protein	0,000413	-2,08	0,007773	-1,82	0,697138	-1,14	0,112849	-1,36	0,051198	1,34	0,002443	1,53

11721828_x_at	55055	ZWILCH	zwilch kinetochore protein	0,001199	-2,01	0,009724	-1,86	0,847203	-1,08	0,358009	-1,22	0,008583	1,53	0,000885	1,65
11721827_a_at	55055	ZWILCH	zwilch kinetochore protein	0,002056	-2,00	0,010056	-1,93	0,933234	-1,04	0,482777	-1,18	0,004129	1,64	0,000848	1,70
11744887_s_at	55055	ZWILCH	zwilch kinetochore protein	0,003532	-1,75	0,030768	-1,59	0,784337	-1,10	0,040059	-1,47	0,621158	1,08	0,234578	1,19
11718367_a_at	26009	ZZZ3	zinc finger, ZZ-type containing 3	0,001667	-1,86	0,010573	-1,76	0,890772	-1,05	0,002921	-1,79	0,93673	-1,01	0,823484	1,04
11725476_at	9744	ACAP1	ArfGAP with coiled-coil, ankyrin repeat and PH domains 1	0,01193	1,67	0,011158	1,83	0,804562	-1,10	0,623757	-1,11	2,09E-05	-2,04	4,38E-05	-1,86
11749525_a_at	208	AKT2	v-akt murine thymoma viral oncogene homolog 2	0,04844	1,47	0,025177	1,66	0,722746	-1,13	0,006536	-1,73	3,22E-09	-2,87	7,87E-09	-2,54
11724313_a_at	126133	ALDH16A1	aldehyde dehydrogenase 16 family, member A1	0,001783	1,40	0,001413	1,50	0,697141	-1,07	0,761171	1,04	1,38E-05	-1,45	9,14E-05	-1,35
11759522_a_at	84266	ALKBH7	alkB, alkylation repair homolog 7 (E. coli)	0,001082	1,58	0,001595	1,67	0,812083	-1,06	0,955958	-1,01	2,63E-06	-1,68	4,25E-06	-1,59
11763990_a_at	84266	ALKBH7	alkB, alkylation repair homolog 7 (E. coli)	0,001382	1,56	0,002943	1,62	0,891842	-1,04	0,802313	1,04	4,62E-05	-1,56	4,31E-05	-1,50
11740162_a_at	116985	ARAP1	ArfGAP with RhoGAP domain, ankyrin repeat and PH domain 1	0,005163	1,81	0,030617	1,68	0,854171	1,08	0,519575	1,15	0,022046	-1,46	0,0033	-1,57
11719038_a_at	116985	ARAP1	ArfGAP with RhoGAP domain, ankyrin repeat and PH domain 1	0,000993	1,75	0,011439	1,61	0,773457	1,09	0,045527	1,38	0,238542	-1,16	0,04883	-1,27
11742838_at	408	ARRB1	arrestin, beta 1	0,018586	1,48	0,007762	1,69	0,652302	-1,14	0,743197	-1,06	2,13E-05	-1,79	0,000246	-1,57
11721767_s_at	55210 /// 83858	ATAD3A /// ATAD3B	ATPase family, AAA domain containing 3A /// ATPase family, AAA domain containing 3B	0,002671	1,51	0,000459	1,79	0,477274	-1,19	0,887858	-1,02	1,48E-07	-1,83	1,54E-05	-1,54
11724354_x_at	89849	ATG16L2	autophagy related 16-like 2 (S. cerevisiae)	0,021671	1,53	0,025461	1,62	0,88503	-1,06	0,871865	-1,04	0,000571	-1,68	0,000768	-1,59
11720313_a_at	533	ATP6V0B	ATPase, H+ transporting, lysosomal 21kDa, V0 subunit b	0,016031	1,53	0,040533	1,52	0,984167	1,01	0,425528	-1,16	8,50E-05	-1,76	1,67E-05	-1,77
200078_PM_s_at	533	ATP6V0B	ATPase, H+ transporting, lysosomal 21kDa, V0 subunit b	0,009834	1,52	0,019813	1,55	0,965306	-1,02	0,954067	-1,01	0,000569	-1,57	0,000306	-1,54
11735895_at	10018	BCL2L11	BCL2-like 11 (apoptosis facilitator)	0,001019	1,62	0,011008	1,51	0,788815	1,07	6,28E-05	1,84	0,073447	1,22	0,231901	1,14
11741810_x_at	274	BIN1	bridging integrator 1	0,009834	1,45	0,007673	1,57	0,76055	-1,08	0,935039	-1,01	6,92E-05	-1,59	0,000267	-1,47
11757435_x_at	274	BIN1	bridging integrator 1	0,007164	1,58	0,008801	1,68	0,848511	-1,06	0,995341	-1,00	0,000138	-1,68	0,00023	-1,58
11719631_s_at	274	BIN1	bridging integrator 1	0,034317	1,57	0,034111	1,69	0,859698	-1,08	0,854593	-1,05	0,000931	-1,76	0,001657	-1,64
11718002_at	64115	C10orf54	chromosome 10 open reading frame 54	0,002868	1,81	0,045627	1,56	0,662653	1,16	2,37E-05	2,49	0,002423	1,59	0,025854	1,37
11737038_at	255809	C19orf38	chromosome 19 open reading frame 38	0,002057	1,82	0,020684	1,65	0,772453	1,10	0,040305	1,47	0,461276	-1,12	0,136515	-1,24
11732773_at	284001	CCDC57	coiled-coil domain containing 57	0,001	1,48	0,002848	1,50	0,941607	-1,02	0,333607	1,12	0,001005	-1,34	0,000771	-1,32
11755564_x_at	6349 /// 414062	CCL3L1 /// CCL3L3	chemokine (C-C motif) ligand 3-like 1 /// chemokine (C-C motif) ligand 3-like 3	0,00846	2,08	0,030612	1,98	0,93977	1,05	0,000827	2,63	0,200828	1,33	0,269275	1,27
11735864_a_at	11126	CD160	CD160 molecule	0,000687	1,85	0,005405	1,75	0,875568	1,05	4,21E-05	2,16	0,122531	1,23	0,238484	1,17

11728503_at	10871	CD300C	CD300c molecule	0,000557	1,76	0,014265	1,55	0,633117	1,14	6,63E-06	2,19	0,00403	1,42	0,052705	1,25
11750408_a_at	976	CD97	CD97 molecule	0,006844	1,74	0,039521	1,62	0,849178	1,08	0,081818	1,43	0,453851	-1,13	0,201349	-1,22
11763717_a_at	1028	CDKN1C	cyclin-dependent kinase inhibitor 1C (p57, Kip2)	0,016413	1,86	0,010419	2,18	0,732898	-1,17	4,21E-05	3,24	0,053085	1,49	0,003676	1,74
11729118_a_at	1028	CDKN1C	cyclin-dependent kinase inhibitor 1C (p57, Kip2)	0,030873	1,60	0,021136	1,79	0,771737	-1,12	0,000128	2,48	0,058363	1,39	0,006077	1,55
11763718_x_at	1028	CDKN1C	cyclin-dependent kinase inhibitor 1C (p57, Kip2)	0,047697	1,82	0,026614	2,18	0,737442	-1,20	0,000155	3,47	0,051546	1,59	0,003725	1,91
11718590_at	79643	CHMP6	charged multivesicular body protein 6	0,010823	1,46	0,014839	1,52	0,890087	-1,04	0,59893	1,09	0,004203	-1,40	0,00668	-1,34
11723714_a_at	56265	CPXM1	carboxypeptidase X (M14 family), member 1	0,107967	1,27	0,004179	1,68	0,413308	-1,32	0,753701	-1,05	5,64E-06	-1,77	0,008089	-1,34
11721704_a_at	1396	CRIP1	cysteine-rich protein 1 (intestinal)	0,000775	2,56	0,047837	1,82	0,477274	1,40	0,431959	1,24	0,06528	-1,47	0,000204	-2,06
11754298_x_at	1453	CSNK1D	casein kinase 1, delta	0,002947	1,42	0,003291	1,51	0,786228	-1,06	0,009072	1,36	0,266884	-1,11	0,635856	-1,05
11743972_a_at	54541	DDIT4	DNA-damage-inducible transcript 4	0,0982	1,65	0,014126	2,41	0,491904	-1,46	0,144808	1,57	0,079053	-1,53	0,858651	-1,05
11724979_a_at	64174	DPEP2	dipeptidase 2	0,00194	1,94	0,033483	1,66	0,661624	1,17	0,081103	1,44	0,397539	-1,15	0,049282	-1,35
11741041_x_at	29952	DPP7	dipeptidyl-peptidase 7	0,002832	1,53	0,002406	1,67	0,716783	-1,09	0,510688	-1,10	2,79E-07	-1,84	1,07E-06	-1,69
11749517_x_at	10938	EHD1	EH-domain containing 1	0,00056	1,81	0,008855	1,64	0,729496	1,10	0,000234	1,89	0,28096	1,15	0,772601	1,04
11762854_a_at	1973 /// 26781 /// 652965 /// 652966 /// 101928634	EIF4A1 /// LOC101928634 /// SNORA48 /// SNORA67 /// SNORD10	eukaryotic translation initiation factor 4A1 /// uncharacterized LOC101928634 /// small	0,217509	1,25	0,04794	1,52	0,550535	-1,21	0,005553	-1,68	1,00E-08	-2,54	2,34E-07	-2,10
11748205_a_at	2015	EMR1	egf-like module containing, mucin-like, hormone receptor-like 1	0,000101	1,66	0,002886	1,53	0,686786	1,09	6,67E-05	1,67	0,361636	1,09	0,972053	1,00
11731775_a_at	2015	EMR1	egf-like module containing, mucin-like, hormone receptor-like 1	0,001772	1,69	0,021202	1,54	0,740203	1,10	0,009544	1,53	0,977799	-1,00	0,446415	-1,10
11716926_a_at	11336	EXOC3	exocyst complex component 3	0,000435	1,52	0,000557	1,61	0,784337	-1,06	0,177995	1,16	0,000271	-1,38	0,000916	-1,31
11729638_a_at	162427	FAM134C	family with sequence similarity 134, member C	0,040747	1,42	0,02213	1,58	0,728568	-1,11	0,860826	1,04	0,00217	-1,52	0,013105	-1,37
11726708_a_at	348262	FAM195B	family with sequence similarity 195, member B	0,004114	1,43	0,004332	1,51	0,788108	-1,06	0,121122	-1,21	1,95E-08	-1,83	2,78E-08	-1,72
11744294_a_at	348262	FAM195B	family with sequence similarity 195, member B	0,000642	1,53	0,003723	1,51	0,947465	1,02	0,628757	1,06	0,000233	-1,42	3,25E-05	-1,44

11744281_a_at	9679	FAM53B	family with sequence similarity 53, member B	0,019831	1,55	0,024368	1,63	0,890618	-1,05	0,026437	-1,53	3,98E-08	-2,49	1,96E-08	-2,37
11744081_at	54461	FBXW5	F-box and WD repeat domain containing 5	6,77E-05	1,73	0,000638	1,68	0,912109	1,03	0,007485	1,40	0,054026	-1,20	0,017951	-1,24
11745742_s_at	89846	FGD3	FYVE, RhoGEF and PH domain containing 3	0,00404	1,65	0,024261	1,56	0,863369	1,06	0,424914	1,15	0,025135	-1,35	0,004252	-1,43
11731599_a_at	2355	FOSL2	FOS-like antigen 2	0,018214	1,56	0,015087	1,70	0,794147	-1,09	0,818518	-1,05	0,000136	-1,79	0,000389	-1,64
11741793_a_at	282969	FUOM	fucose mutarotase	0,00194	1,46	0,000637	1,65	0,550535	-1,13	0,365474	1,12	4,96E-05	-1,48	0,001909	-1,31
11716871_at	2548	GAA	glucosidase, alpha; acid	0,000996	1,96	0,026341	1,64	0,597797	1,19	0,048211	1,47	0,492579	-1,12	0,04541	-1,33
11749866_x_at	2584	GALK1	galactokinase 1	0,014865	1,46	0,012665	1,57	0,794147	-1,08	0,697459	1,07	0,001839	-1,47	0,006243	-1,36
11738990_x_at	23193	GANAB	glucosidase, alpha; neutral AB	0,391099	1,24	0,043241	1,76	0,447888	-1,42	0,013209	-1,84	3,90E-08	-3,25	7,25E-06	-2,29
11738989_a_at	23193	GANAB	glucosidase, alpha; neutral AB	0,350412	1,21	0,040057	1,59	0,455446	-1,32	0,063804	-1,44	6,52E-07	-2,30	0,000138	-1,74
11755270_a_at	23193	GANAB	glucosidase, alpha; neutral AB	0,474129	1,19	0,047171	1,71	0,431149	-1,43	0,073143	-1,53	1,29E-06	-2,61	0,000512	-1,82
11763328_a_at	23353 /// 51608	GET4 /// SUN1	golgi to ER traffic protein 4 homolog (S. cerevisiae) /// Sad1 and UNC84 domain contain	0,004451	1,42	0,00323	1,54	0,707925	-1,08	0,560606	1,08	0,000269	-1,42	0,001949	-1,32
11722372_a_at	23062	GGA2	golgi-associated, gamma adaptin ear containing, ARF binding protein 2	4,20E-05	1,61	0,000855	1,52	0,769917	1,06	0,505645	1,07	3,05E-05	-1,42	4,55E-07	-1,50
11741396_s_at	2678 /// 2679 /// 91227 /// 92086 /// 728441 /// 100132705 /// 102724197	GGT1 /// GGT2 /// GGT3P /// GGTLC1 /// GGTLC2 /// LOC100132705 /// LOC102724197	gamma-glutamyltransferase 1 /// gamma-glutamyltransferase 2 /// gamma-glutamyltransfera	0,007388	1,78	0,019822	1,78	0,997281	1,00	0,040225	1,55	0,445845	-1,14	0,425402	-1,15
11756226_a_at	56850	GRIPAP1	GRIP1 associated protein 1	0,127411	1,33	0,026967	1,61	0,552086	-1,21	0,003647	-1,75	1,34E-09	-2,81	2,56E-08	-2,32
11733846_a_at	2870	GRK6	G protein-coupled receptor kinase 6	0,00625	1,66	0,020792	1,63	0,966217	1,02	0,433052	1,16	0,019046	-1,40	0,008052	-1,43
11729480_a_at	2987	GUK1	guanylate kinase 1	0,002426	1,53	0,008884	1,52	0,981486	1,01	0,070262	1,28	0,113421	-1,19	0,081246	-1,20
11753827_x_at	2994	GYPB	glycophorin B (MNS blood group)	0,005072	1,77	0,024056	1,68	0,905786	1,05	3,16E-05	2,52	0,010682	1,50	0,016271	1,42
11728560_at	3003	GZMK	granzyme K (granzyme 3; tryptase II)	0,000474	2,91	0,03134	2,02	0,477274	1,44	1,33E-05	4,02	0,002287	1,99	0,132528	1,38
11718484_s_at	3005	H1F0	H1 histone family, member 0	0,0953	1,35	0,02271	1,61	0,574236	-1,19	0,28328	-1,22	7,05E-06	-1,96	0,000194	-1,64
11715564_s_at	8971	H1FX	H1 histone family, member X	0,040194	1,45	0,023972	1,62	0,747184	-1,11	0,105661	-1,35	6,31E-07	-2,18	1,91E-06	-1,96

11755386_a_at	3067	HDC	histidine decarboxylase	0,210636	1,39	0,015192	2,10	0,431149	-1,51	0,03117	1,77	0,427721	-1,19	0,238855	1,27
11732453_s_at	3081	HGD	homogentisate 1,2-dioxygenase	0,001485	2,51	0,019901	2,11	0,723977	1,19	0,000704	2,69	0,276857	1,28	0,774411	1,07
11720837_a_at	7290	HIRA	histone cell cycle regulator	0,034756	1,45	0,022607	1,59	0,763875	-1,10	0,000672	1,88	0,234933	1,18	0,044427	1,30
11759110_a_at	8345	HIST1H2BH	histone cluster 1, H2bh	0,003615	1,92	0,007278	2,01	0,928105	-1,04	0,001312	2,09	0,838392	1,04	0,660015	1,09
11759111_x_at	8345	HIST1H2BH	histone cluster 1, H2bh	0,014687	1,62	0,035038	1,62	0,995181	1,00	0,01273	1,65	0,914274	1,02	0,92838	1,02
11728049_s_at	8294 /// 8359 /// 8360 /// 8361 /// 8362 /// 8363 /// 8364 /// 8365 /// 8366 /// 8367 /	HIST1H4A /// HIST1H4B /// HIST1H4C /// HIST1H4D /// HIST1H4E /// HIST1H4F /// HIST1H4H /// HIST1H4I /// HIST1H4J /// HIST1H4K /// HIST1H4L /// HIST2H4A /// HIST2H4B /// HIST4H4	histone cluster 1, H4a /// histone cluster 1, H4b /// histone cluster 1, H4c /// histon	0,005787	1,66	0,013724	1,69	0,973558	-1,01	0,001049	1,87	0,504551	1,11	0,430159	1,12
11753898_x_at	3117 /// 100509457	HLA-DQA1 /// LOC100509457	major histocompatibility complex, class II, DQ alpha 1 /// HLA class II histocompatibil	0,291221	1,60	0,032856	2,92	0,4599	-1,83	0,711912	-1,19	0,000364	-3,48	0,046116	-1,91
11758340_x_at	3117 /// 100509457	HLA-DQA1 /// LOC100509457	major histocompatibility complex, class II, DQ alpha 1 /// HLA class II histocompatibil	0,401107	1,35	0,040109	2,28	0,436827	-1,68	0,793698	-1,11	0,000888	-2,52	0,123336	-1,50
11715826_x_at	10362	HMG20B	high mobility group 20B	0,003889	1,49	0,007711	1,53	0,927108	-1,03	0,273382	-1,16	5,48E-07	-1,78	2,39E-07	-1,73

11715670_a_at	8519	IFITM1	interferon induced transmembrane protein 1	0,240712	1,41	0,047699	1,94	0,534516	-1,38	0,002877	2,45	0,328821	1,26	0,010115	1,73
11743475_a_at	3687	ITGAX	integrin, alpha X (complement component 3 receptor 4 subunit)	0,004657	2,08	0,031984	1,87	0,825344	1,11	0,000243	2,72	0,063493	1,45	0,168011	1,31
11725966_a_at	3695	ITGB7	integrin, beta 7	0,001667	1,98	0,020089	1,76	0,742062	1,13	0,002047	1,95	0,545143	1,11	0,939522	-1,02
11759966_at	3707	ITPKB	inositol-trisphosphate 3-kinase B	0,027662	1,59	0,042366	1,64	0,945761	-1,03	0,028664	1,60	0,886518	-1,03	0,986851	1,00
11726814_x_at	8284	KDM5D	lysine (K)-specific demethylase 5D	0,218643	2,08	0,035051	4,18	0,50415	-2,02	0,737382	1,25	0,009272	-3,36	0,265835	-1,67
11745012_a_at	8284	KDM5D	lysine (K)-specific demethylase 5D	0,168505	1,80	0,040988	2,73	0,574523	-1,52	0,76895	1,15	0,010309	-2,37	0,170458	-1,56
11755369_a_at	8284	KDM5D	lysine (K)-specific demethylase 5D	0,175852	1,98	0,038858	3,33	0,555183	-1,68	0,735398	1,21	0,011092	-2,75	0,202824	-1,64
11715939_a_at	10657	KHDRBS1	KH domain containing, RNA binding, signal transduction associated 1	0,000877	1,47	8,81E-05	1,75	0,422048	-1,20	0,076395	1,21	3,57E-05	-1,44	0,018253	-1,21
11729229_at	51274	KLF3	Kruppel-like factor 3 (basic)	0,001148	1,73	0,004006	1,75	0,984633	-1,01	0,121163	1,29	0,016254	-1,36	0,011916	-1,35
11743721_at	3956	LGALS1	lectin, galactoside-binding, soluble, 1	0,002101	2,29	0,045385	1,82	0,61249	1,26	0,199372	1,40	0,213089	-1,30	0,010615	-1,63
11731798_a_at	3957	LGALS2	lectin, galactoside-binding, soluble, 2	0,001101	4,72	0,024572	3,23	0,632339	1,46	0,000103	6,76	0,038112	2,09	0,311287	1,43
11759824_a_at	11024	LILRA1	leukocyte immunoglobulin-like receptor, subfamily A (with TM domain), member 1	0,002766	1,69	0,02039	1,58	0,830498	1,07	0,000433	1,89	0,184629	1,20	0,409742	1,12
11745791_a_at	1E+08	LOC100288152	uncharacterized LOC100288152	0,108969	1,46	0,009957	2,06	0,45045	-1,41	0,503705	1,19	0,003593	-1,74	0,257163	-1,24
11747650_a_at	10489	LRRC41	leucine rich repeat containing 41	0,00677	1,44	0,005119	1,56	0,737741	-1,08	0,218545	-1,18	1,25E-07	-1,85	3,59E-07	-1,71
11723156_a_at	4046	LSP1	lymphocyte-specific protein 1	0,003349	2,02	0,044723	1,72	0,692951	1,18	0,825087	1,06	0,009135	-1,62	0,000195	-1,90
11724206_a_at	8379	MAD1L1	MAD1 mitotic arrest deficient-like 1 (yeast)	0,003967	1,80	0,030038	1,64	0,810582	1,09	0,990491	-1,00	0,001611	-1,65	7,25E-05	-1,80
11733087_a_at	4094	MAF	v-maf avian musculoaponeurotic fibrosarcoma oncogene homolog	0,021632	1,81	0,041333	1,83	0,979128	-1,02	5,12E-05	3,17	0,007552	1,73	0,003051	1,75
11721458_s_at	11253	MAN1B1	mannosidase, alpha, class 1B, member 1	0,047983	1,41	0,025513	1,57	0,728262	-1,11	0,244051	-1,23	6,00E-06	-1,94	2,67E-05	-1,74
11757369_s_at	4125	MAN2B1	mannosidase, alpha, class 2B, member 1	0,003024	1,65	0,027506	1,52	0,778649	1,09	0,681357	1,08	0,008244	-1,41	0,000427	-1,53
11744570_x_at	4123	MAN2C1	mannosidase, alpha, class 2C, member 1	0,018135	1,52	0,017711	1,63	0,833117	-1,07	0,26053	-1,23	3,15E-06	-2,00	4,18E-06	-1,86
11722177_a_at	4123	MAN2C1	mannosidase, alpha, class 2C, member 1	0,001585	1,55	0,002796	1,62	0,853121	-1,05	0,928306	1,01	1,79E-05	-1,60	2,25E-05	-1,53
11755586_x_at	4123	MAN2C1	mannosidase, alpha, class 2C, member 1	0,02171	1,49	0,019269	1,60	0,818441	-1,08	0,366817	-1,18	1,07E-05	-1,88	1,84E-05	-1,75
11740300_x_at	51257	MARCH2	membrane-associated ring finger (C3HC4) 2, E3 ubiquitin protein ligase	0,00194	2,85	0,033956	2,22	0,657899	1,29	0,003108	2,70	0,4645	1,22	0,853523	-1,06

11724409_a_at	51257	MARCH2	membrane-associated ring finger (C3HC4) 2, E3 ubiquitin protein ligase	0,002671	2,90	0,048054	2,19	0,64096	1,32	0,007908	2,54	0,614115	1,16	0,648687	-1,14
11731227_a_at	4145	MATK	megakaryocyte-associated tyrosine kinase	0,110064	1,28	0,023966	1,50	0,556224	-1,17	0,009629	-1,51	5,90E-09	-2,27	1,13E-07	-1,93
11744359_s_at	53615	MBD3	methyl-CpG binding domain protein 3	0,017209	1,45	0,008346	1,63	0,678445	-1,12	0,550894	-1,11	6,72E-06	-1,80	5,37E-05	-1,60
11745701_a_at	284207	METRNL	meteorin, glial cell differentiation regulator-like	0,002952	1,71	0,010801	1,69	0,975719	1,01	0,122387	1,31	0,070881	-1,28	0,044143	-1,30
11733599_s_at	284207	METRNL	meteorin, glial cell differentiation regulator-like	0,004414	2,58	0,015005	2,52	0,975918	1,02	0,135035	1,64	0,094868	-1,54	0,062553	-1,58
11719686_a_at	64780	MICAL1	microtubule associated monooxygenase, calponin and LIM domain containing 1	0,003071	1,64	0,02905	1,50	0,770053	1,09	0,435742	-1,14	4,54E-05	-1,72	7,07E-07	-1,87
11738859_s_at	80700 /// 100616371	MIR4746 /// UBXN6	microRNA 4746 /// UBX domain protein 6	0,001774	1,65	0,006732	1,64	0,983113	1,01	0,160646	1,24	0,023977	-1,32	0,012993	-1,33
11718608_at	7318 /// 100847079	MIR5193 /// UBA7	microRNA 5193 /// ubiquitin-like modifier activating enzyme 7	0,018778	1,45	0,022365	1,53	0,881995	-1,05	0,584351	-1,10	7,30E-05	-1,68	8,11E-05	-1,60
11717168_a_at	5187 /// 102465532	MIR6883 /// PER1	microRNA 6883 /// period circadian clock 1	0,041634	1,86	0,012177	2,47	0,599631	-1,32	0,880782	-1,05	0,000128	-2,60	0,002815	-1,96
11716228_x_at	9526	MPDU1	mannose-P-dolichol utilization defect 1	0,006719	1,47	0,000665	1,81	0,436827	-1,23	0,877623	-1,03	2,91E-07	-1,86	8,67E-05	-1,51
11723864_s_at	10903	MTMR11	myotubularin related protein 11	5,98E-05	1,97	0,007726	1,60	0,461895	1,23	2,81E-05	2,01	0,054361	1,26	0,882687	1,02
11737388_s_at	10903	MTMR11	myotubularin related protein 11	0,000238	1,71	0,009457	1,50	0,575558	1,14	0,00112	1,58	0,673493	1,05	0,450087	-1,08
11719249_at	4084	MXD1	MAX dimerization protein 1	0,181433	1,29	0,045699	1,56	0,580039	-1,20	0,334276	1,21	0,099946	-1,29	0,694047	-1,07
11722495_a_at	10514	MYBBP1A	MYB binding protein (P160) 1a	0,031914	1,48	0,018001	1,65	0,729889	-1,12	0,226578	-1,25	2,21E-06	-2,07	8,78E-06	-1,85
11720624_a_at	4615	MYD88	myeloid differentiation primary response 88	0,004085	1,66	0,038335	1,51	0,76197	1,10	0,072434	1,37	0,488898	-1,11	0,140585	-1,21
11755830_a_at	64005	MYO1G	myosin IG	0,010852	1,70	0,036848	1,65	0,943039	1,03	0,711712	-1,09	0,00046	-1,80	6,78E-05	-1,86
11721719_at	27163	NAAA	N-acylethanolamine acid amidase	0,001349	1,88	0,039889	1,56	0,572117	1,20	0,000895	1,93	0,159206	1,23	0,884078	1,03
11754339_a_at	55191	NADSYN1	NAD synthetase 1	0,00194	1,52	0,002714	1,61	0,80825	-1,06	0,931166	-1,01	6,16E-06	-1,63	1,11E-05	-1,54
11759971_at	9437	NCR1	natural cytotoxicity triggering receptor 1	0,000288	2,44	0,003841	2,19	0,795479	1,11	7,03E-05	2,67	0,279007	1,22	0,634142	1,10
11737423_a_at	22861	NLRP1	NLR family, pyrin domain containing 1	0,001877	1,73	0,022744	1,56	0,735255	1,11	0,027844	1,45	0,629898	-1,07	0,186368	-1,19
11727215_a_at	3084	NRG1	neuregulin 1	0,00194	1,86	0,032772	1,60	0,666059	1,16	0,000309	2,10	0,077967	1,31	0,430081	1,13

11720083_a_at	84304	NUDT22	nudix (nucleoside diphosphate linked moiety X)-type motif 22	0,004597	1,51	0,009341	1,55	0,939496	-1,02	0,496578	-1,11	5,92E-06	-1,72	2,54E-06	-1,68
11726364_x_at	8638	OASL	2'-5'-oligoadenylate synthetase-like	0,011736	1,55	0,037016	1,51	0,954447	1,02	0,000715	1,85	0,142745	1,22	0,175139	1,20
11744920_a_at	440836	ODF3B	outer dense fiber of sperm tails 3B	0,003678	1,73	0,013142	1,71	0,973558	1,01	0,003825	1,73	0,932391	1,01	0,997689	1,00
11727757_at	5008	OSM	oncostatin M	0,326978	1,26	0,041995	1,70	0,469931	-1,35	0,028076	1,65	0,890508	-1,03	0,10918	1,32
11730955_a_at	5023	P2RX1	purinergic receptor P2X, ligand-gated ion channel, 1	0,006805	1,54	0,024459	1,50	0,948456	1,02	0,851225	1,03	0,002756	-1,45	0,000611	-1,49
11735662_a_at	5029	P2RY2	purinergic receptor P2Y, G-protein coupled, 2	0,007043	1,52	0,013593	1,55	0,941964	-1,02	0,012293	1,48	0,696388	-1,05	0,843069	-1,03
11731978_s_at	9159	PCSK7	proprotein convertase subtilisin/kexin type 7	0,001234	1,52	0,005199	1,51	0,97894	1,01	0,985171	-1,00	4,33E-05	-1,51	7,06E-06	-1,52
11759682_at	10611	PDLIM5	PDZ and LIM domain 5	0,07441	1,39	0,044601	1,54	0,76081	-1,11	0,006526	1,68	0,583252	1,09	0,187209	1,21
11757843_s_at	8682	PEA15	phosphoprotein enriched in astrocytes 15	0,002603	1,53	0,005735	1,57	0,936392	-1,02	0,009485	1,44	0,440125	-1,09	0,574964	-1,07
11747463_a_at	5261	PHKG2	phosphorylase kinase, gamma 2 (testis)	0,00265	1,42	0,002347	1,53	0,721435	-1,07	0,780636	-1,04	1,65E-06	-1,59	6,92E-06	-1,48
11756358_a_at	1263	PLK3	polo-like kinase 3	0,006926	1,87	0,017033	1,88	0,985835	-1,01	0,005088	1,93	0,914587	1,02	0,879712	1,03
11724891_s_at	5442	POLRMT	polymerase (RNA) mitochondrial (DNA directed)	0,001199	1,57	0,003211	1,60	0,93698	-1,02	0,94866	-1,01	1,10E-05	-1,62	5,05E-06	-1,58
11754832_a_at	54776	PPP1R12C	protein phosphatase 1, regulatory subunit 12C	0,006879	1,76	0,009732	1,87	0,881913	-1,06	0,030529	-1,57	6,78E-09	-2,93	3,21E-09	-2,76
11743140_x_at	54776	PPP1R12C	protein phosphatase 1, regulatory subunit 12C	0,018803	1,41	0,015703	1,52	0,796335	-1,07	0,088816	-1,29	1,69E-07	-1,95	2,65E-07	-1,82
11753564_x_at	94274	PPP1R14A	protein phosphatase 1, regulatory (inhibitor) subunit 14A	0,0053	1,60	0,027473	1,53	0,884058	1,05	0,007223	1,58	0,825107	1,03	0,922382	-1,02
11747295_a_at	5551	PRF1	perforin 1 (pore forming protein)	0,001239	1,91	0,035755	1,59	0,579111	1,20	5,16E-05	2,34	0,00978	1,48	0,159365	1,23
11729036_x_at	5616	PRKY	protein kinase, Y-linked, pseudogene	0,104779	1,37	0,03468	1,61	0,6393	-1,18	0,417078	1,18	0,044711	-1,36	0,334772	-1,16
11737470_at	400668	PRSS57	protease, serine, 57	0,218658	1,19	0,01112	1,52	0,413308	-1,28	0,39486	-1,13	3,85E-06	-1,72	0,003747	-1,35
11715516_x_at	5709	PSMD3	proteasome (prosome, macropain) 26S subunit, non-ATPase, 3	0,018578	1,37	0,004169	1,59	0,536251	-1,16	0,122122	-1,24	1,79E-08	-1,97	5,57E-07	-1,70
11715515_a_at	5709	PSMD3	proteasome (prosome, macropain) 26S subunit, non-ATPase, 3	0,011939	1,44	0,011242	1,53	0,807245	-1,07	0,374578	-1,14	3,35E-06	-1,75	5,78E-06	-1,64
11718061_at	5816	PVALB	parvalbumin	0,004267	1,95	0,028697	1,78	0,834827	1,09	0,004657	1,95	0,656809	1,09	0,992648	-1,00
11756422_s_at	2889	RAPGEF1	Rap guanine nucleotide exchange factor (GEF) 1	0,000141	1,62	0,002661	1,52	0,759383	1,06	0,932586	1,01	1,53E-05	-1,50	1,87E-07	-1,60
11722248_a_at	11186	RASSF1	Ras association (RalGDS/AF-6) domain family member 1	0,093637	1,31	0,013549	1,59	0,492775	-1,22	0,879561	-1,03	0,000157	-1,63	0,012524	-1,34
11756449_x_at	10616	RBCK1	RanBP-type and C3HC4-type zinc finger containing 1	0,010873	1,48	0,016961	1,53	0,917942	-1,03	0,804589	1,04	0,001769	-1,47	0,001902	-1,42
11745856_x_at	153830	RNF145	ring finger protein 145	0,030924	1,55	0,046196	1,60	0,943772	-1,03	0,035048	1,54	0,849238	-1,04	0,9847	-1,00

11723494_a_at	115992	RNF166	ring finger protein 166	0,00111	1,98	0,002957	2,05	0,93176	-1,04	0,035731	1,52	0,057878	-1,35	0,080287	-1,30
11756860_x_at	6050	RNH1	ribonuclease/angiogenin inhibitor 1	0,001086	1,78	0,009643	1,66	0,827916	1,07	0,020341	1,48	0,401279	-1,12	0,151068	-1,20
11763726_a_at	6134 /// 26778	RPL10 /// SNORA70	ribosomal protein L10 /// small nucleolar RNA, H/ACA box 70	0,02156	1,46	0,018367	1,57	0,808986	-1,08	0,073749	-1,35	1,62E-07	-2,11	2,21E-07	-1,97
11752267_a_at	8986	RPS6KA4	ribosomal protein S6 kinase, 90kDa, polypeptide 4	0,001953	1,55	0,009505	1,52	0,935661	1,02	0,210791	1,19	0,025014	-1,27	0,008315	-1,30
11743428_a_at	864	RUNX3	runt-related transcription factor 3	0,004202	1,98	0,019842	1,88	0,913104	1,05	0,063818	1,55	0,305751	-1,22	0,168068	-1,28
11752664_a_at	53637	S1PR5	sphingosine-1-phosphate receptor 5	0,001383	1,82	0,028544	1,57	0,640693	1,16	8,89E-05	2,15	0,025852	1,37	0,225457	1,18
11722269_a_at	54440	SASH3	SAM and SH3 domain containing 3	0,002309	1,94	0,019108	1,77	0,81094	1,10	0,203587	-1,31	1,94E-06	-2,33	3,49E-08	-2,55
11722270_at	54440	SASH3	SAM and SH3 domain containing 3	0,002644	1,75	0,019981	1,62	0,826943	1,08	0,368439	-1,18	1,18E-05	-1,92	2,95E-07	-2,06
11755102_a_at	83642	SELO	selenoprotein O	0,002954	1,54	0,007732	1,56	0,970561	-1,01	0,099553	-1,26	4,73E-08	-1,97	8,45E-09	-1,94
11720763_a_at	6404	SELPLG	selectin P ligand	0,018275	1,49	0,032395	1,51	0,959652	-1,02	0,066645	1,36	0,454707	-1,11	0,539055	-1,09
11715368_a_at	710	SERPING1	serpin peptidase inhibitor, clade G (C1 inhibitor), member 1	0,308458	1,24	0,040533	1,64	0,469931	-1,32	0,044474	-1,53	3,23E-07	-2,50	4,26E-05	-1,90
11723217_x_at	81855	SFXN3	sideroflexin 3	0,032199	1,47	0,028315	1,58	0,830006	-1,08	0,243125	1,24	0,091643	-1,27	0,228274	-1,18
11749207_a_at	117157	SH2D1B	SH2 domain containing 1B	0,000681	1,95	0,031209	1,58	0,520962	1,23	0,000342	2,02	0,090871	1,28	0,826274	1,04
11740157_at	117157	SH2D1B	SH2 domain containing 1B	0,000741	2,22	0,022869	1,79	0,581505	1,24	0,000391	2,31	0,148746	1,29	0,836645	1,04
11754777_a_at	9047	SH2D2A	SH2 domain containing 2A	0,001126	1,84	0,010672	1,70	0,814129	1,08	0,000164	2,06	0,175247	1,21	0,417936	1,12
11738124_x_at	89790	SIGLEC10	sialic acid binding Ig-like lectin 10	0,013456	1,56	0,028991	1,57	0,985561	-1,01	1,24E-07	3,12	4,37E-06	1,99	6,43E-07	2,00
11732800_a_at	10326	SIRPB1	signal-regulatory protein beta 1	0,000528	1,64	0,003291	1,61	0,938933	1,02	2,10E-05	1,89	0,127219	1,18	0,172403	1,15
11732801_at	10326	SIRPB1	signal-regulatory protein beta 1	0,00176	3,11	0,013748	2,72	0,841088	1,14	0,000209	4,01	0,162708	1,47	0,354044	1,29
11763339_a_at	10572	SIVA1	SIVA1, apoptosis-inducing factor	0,000505	1,52	0,001528	1,55	0,940197	-1,02	0,510432	-1,08	1,21E-07	-1,67	3,67E-08	-1,64
11734993_at	6497	SKI	SKI proto-oncogene	0,193953	1,25	0,038312	1,50	0,537979	-1,20	0,464904	1,14	0,04049	-1,32	0,516436	-1,10
11763528_s_at	6560	SLC12A4	solute carrier family 12 (potassium/chloride transporter), member 4	0,002541	1,45	0,001936	1,57	0,696068	-1,09	0,16454	1,18	0,002599	-1,33	0,020764	-1,23
11715517_s_at	51629	SLC25A39	solute carrier family 25, member 39	0,006747	1,42	0,006288	1,51	0,786563	-1,07	0,366567	1,13	0,003816	-1,34	0,014406	-1,26
11741149_x_at	6520	SLC3A2	solute carrier family 3 (amino acid transporter heavy chain), member 2	0,0002	1,58	0,00254	1,51	0,835506	1,04	0,883818	1,02	1,97E-05	-1,49	5,55E-07	-1,55
11754529_x_at	6520	SLC3A2	solute carrier family 3 (amino acid transporter heavy chain), member 2	0,000227	1,62	0,003364	1,53	0,782198	1,06	0,725485	1,05	0,000106	-1,46	2,09E-06	-1,55
11754980_a_at	6604	SMARCD3	SWI/SNF related, matrix associated, actin dependent regulator of chromatin, subfamily d	0,000993	1,58	0,003308	1,59	0,975937	-1,01	0,029579	1,33	0,086547	-1,20	0,087661	-1,18

11763697_s_at	677844 /// 735301	SNHG9 /// SNORA78	small nucleolar RNA host gene 9 (non-protein coding) /// small nucleolar RNA, H/ACA box	0,051066	1,40	0,027252	1,56	0,730263	-1,11	0,58447	-1,11	0,000111	-1,73	0,000606	-1,55
11734700_a_at	9238 /// 677795	SNORA5B /// TBRG4	small nucleolar RNA, H/ACA box 5B /// transforming growth factor beta regulator 4	0,004551	1,40	0,001602	1,58	0,572092	-1,12	0,927441	-1,01	2,17E-06	-1,60	5,91E-05	-1,42
11747624_x_at	9784	SNX17	sorting nexin 17	0,005921	1,49	0,009166	1,55	0,893631	-1,04	0,930125	1,01	0,000248	-1,52	0,000274	-1,47
11722639_a_at	23648	SSBP3	single stranded DNA binding protein 3	0,295802	1,19	0,028268	1,51	0,441747	-1,27	0,012813	-1,51	1,38E-08	-2,27	2,79E-06	-1,79
11763451_s_at	29101	SSU72	SSU72 RNA polymerase II CTD phosphatase homolog (S. cerevisiae)	0,011978	1,32	0,001569	1,54	0,464366	-1,16	0,356372	1,11	0,000282	-1,38	0,034872	-1,19
11758753_a_at	10948	STARD3	StAR-related lipid transfer (START) domain containing 3	0,001075	1,60	0,002757	1,65	0,920502	-1,03	0,750075	-1,05	1,95E-06	-1,73	9,56E-07	-1,68
11748030_x_at	10494	STK25	serine/threonine kinase 25	0,008455	1,47	0,015318	1,50	0,938697	-1,02	0,743164	-1,05	8,92E-05	-1,58	5,27E-05	-1,54
11730917_at	10629 /// 374395	TAF6L /// TMEM179B	TAF6-like RNA polymerase II, p300/CBP-associated factor (PCAF)-associated factor, 65kDa	0,000495	1,48	0,000523	1,57	0,74029	-1,06	0,26559	1,13	8,76E-05	-1,39	0,000415	-1,31
11732538_at	30009	TBX21	T-box 21	0,003028	1,85	0,033782	1,64	0,735255	1,13	0,000283	2,20	0,068398	1,34	0,284528	1,18
11760584_a_at	10730	TCRDV2 /// TRDC /// TRDC /// YME1L1	SubName: Full=cDNA FLJ52034; /// RecName: Full=T-cell receptor delta chain C region; //	0,006102	1,58	0,026238	1,52	0,916245	1,04	0,000331	1,87	0,10942	1,23	0,169383	1,19
11755219_a_at	7056	THBD	thrombomodulin	0,014718	1,62	0,020154	1,71	0,904099	-1,05	0,000105	2,31	0,051263	1,36	0,015467	1,43
11758842_at	7057	THBS1	thrombospondin 1	0,021613	1,68	0,032691	1,75	0,935703	-1,04	0,002839	2,01	0,455078	1,15	0,313502	1,20
11717535_a_at	91304	TMEM259	transmembrane protein 259	0,020045	1,44	0,003112	1,76	0,479337	-1,22	0,0604	-1,35	1,78E-09	-2,37	1,19E-07	-1,94
11717536_x_at	91304	TMEM259	transmembrane protein 259	0,070486	1,29	0,014634	1,51	0,545719	-1,16	0,080828	-1,29	1,19E-07	-1,94	3,46E-06	-1,66
11718807_at	7133	TNFRSF1B	tumor necrosis factor receptor superfamily, member 1B	0,000976	2,44	0,017391	2,03	0,680135	1,20	0,0003	2,68	0,166558	1,33	0,657893	1,10
11735898_s_at	10043	TOM1	target of myb1 (chicken)	0,002122	1,49	0,004961	1,52	0,938277	-1,02	0,036509	1,30	0,117445	-1,17	0,159638	-1,14
11721107_a_at	80305	TRABD	TraB domain containing	0,013175	1,52	0,016387	1,60	0,878832	-1,05	0,018493	-1,49	7,96E-09	-2,38	3,98E-09	-2,26
11746721_a_at	54210	TREM1	triggering receptor expressed on myeloid cells 1	0,006815	2,15	0,048159	1,89	0,799127	1,14	0,000797	2,68	0,11405	1,42	0,313174	1,25
11716996_a_at	7106	TSPAN4	tetraspanin 4	0,007924	1,69	0,02061	1,69	0,999252	1,00	0,559533	1,13	0,009398	-1,50	0,005151	-1,50
11740991_a_at	7106	TSPAN4	tetraspanin 4	0,111194	1,30	0,030256	1,51	0,598165	-1,16	0,991619	1,00	0,001831	-1,51	0,034204	-1,30
11722449_x_at	1890	TYMP	thymidine phosphorylase	0,000784	2,11	0,010095	1,88	0,758692	1,12	0,000152	2,36	0,175067	1,25	0,517339	1,12
11748775_a_at	7378	UPP1	uridine phosphorylase 1	0,006025	1,84	0,049047	1,64	0,774515	1,12	4,37E-06	3,17	0,000203	1,93	0,000733	1,72
11763837_s_at	7404	UTY	ubiquitously transcribed tetratricopeptide repeat containing, Y-linked	0,06854	1,54	0,016155	1,95	0,57014	-1,27	0,22381	1,34	0,048841	-1,45	0,491038	-1,14

11725645_a_at	64743	WDR13	WD repeat domain 13	0,007857	1,48	0,005767	1,61	0,737741	-1,09	0,8439	-1,03	2,14E-05	-1,67	9,21E-05	-1,53
11728227_a_at	11152	WDR45	WD repeat domain 45	0,002406	1,53	0,005938	1,56	0,95325	-1,02	0,760909	1,05	0,000285	-1,49	0,000161	-1,46
11759628_at	7456	WIPF1	WAS/WASL interacting protein family, member 1	0,009185	1,56	0,020276	1,58	0,979105	-1,01	0,015447	1,52	0,795245	-1,04	0,852775	-1,03
11736645_at	11060	WWP2	WW domain containing E3 ubiquitin protein ligase 2	0,004794	1,63	0,015485	1,62	0,981793	1,01	0,10744	1,32	0,133401	-1,23	0,098686	-1,24
11735394_s_at	6375 /// 6846	XCL1 /// XCL2	chemokine (C motif) ligand 1 /// chemokine (C motif) ligand 2	0,001866	1,75	0,042321	1,50	0,606529	1,17	5,77E-05	2,15	0,008497	1,44	0,117345	1,23
11721356_s_at	83719	YPEL3	yippee-like 3 (Drosophila)	0,007247	1,64	0,034427	1,56	0,890145	1,05	0,833364	1,04	0,005581	-1,49	0,000772	-1,57
11728780_a_at	7535	ZAP70	zeta-chain (TCR) associated protein kinase 70kDa	0,000441	2,00	0,036411	1,55	0,457902	1,29	0,014077	1,57	0,933835	1,01	0,080547	-1,27
11759750_at	400713	ZNF880	zinc finger protein 880	0,041877	1,45	0,009027	1,76	0,547779	-1,21	0,825774	1,05	0,000456	-1,68	0,016711	-1,38

Abbreviations: q, q-value; FC, Fold Change
