# Supplementary material for: CALR mutational status identifies different disease subtypes of essential thrombocythemia showing distinct expression profiles
Source: Blood Cancer J. 2017 Dec 8;7(12):638. doi: 10.1038/s41408-017-0010-2 (PMC5802509; doi:10.1038/s41408-017-0010-2)
Supplement: Supplementary file 5 — Supplementary Table S2 [file 41408_2017_10_MOESM5_ESM.pdf]

Table S2. DEM in the pairwise comparison CALR-mutated vs JAK2V617F-positive ET

Probeset ID	q (ET CALR vs. PV)	FC (ET CALR vs. PV)	q (ET CALR vs. ET V617F)	FC (ET CALR vs. ET V617F)	q (ET V617F vs. PV)	FC (ET V617F vs. PV)	q (ET CALR vs. BM CTR)	FC (ET CALR vs. BM CTR)	q (ET V617F vs. BM CTR)	FC (ET V617F vs. BM CTR)	q (PV vs. BM CTR)	FC (PV vs. BM CTR)
age-miR-101_st	0,025012	1,69	0,00180887	2,08	0,898605	-1,23	0,00258769	2,01	0,943236	-1,04	0,521644	1,19
age-miR-19a_st	0,0136433	2,91	0,00226667	3,90	0,953262	-1,34	0,00223573	3,81	0,984026	-1,02	0,622869	1,31
age-miR-21_st	0,00349428	2,95	0,00374534	3,13	0,999697	-1,06	0,00245995	3,21	0,977766	1,03	0,90482	1,09
age-miR-30b_st	0,00248618	3,30	0,00188691	3,64	0,999697	-1,10	0,0875795	2,20	0,282602	-1,65	0,3626	-1,50
ame-miR-210_st	0,00217402	1,76	0,00273156	1,80	0,999697	-1,02	0,00124959	1,86	0,936887	1,03	0,88072	1,05
bmo-miR-210_st	0,00012432	2,18	0,0129169	1,75	0,817193	1,25	0,000210234	2,20	0,373837	1,26	0,984668	1,01
bmo-miR-2792-3p_st	0,000589456	3,70	0,000137351	4,53	0,990861	-1,22	0,000411601	4,07	0,897377	-1,11	0,891935	1,10
bna-miR166c_st	0,00022041	1,99	0,00396819	1,79	0,978332	1,12	0,0011478	1,90	0,876122	1,06	0,888317	-1,05
bta-let-7b_st	1,65E-05	-1,53	1,05E-05	-1,58	0,999697	1,03	0,0218185	-1,28	0,0234539	1,24	0,045358	1,20
bta-miR-127_st	0,217565	2,43	0,0450761	3,78	0,935558	-1,55	0,0226248	4,05	0,962136	1,07	0,446078	1,66
bta-miR-142_st	0,0327824	1,45	0,0264311	1,50	0,999697	-1,04	0,00624035	1,59	0,861519	1,06	0,659826	1,10
bta-miR-148a_st	0,00155492	1,64	0,000600433	1,75	0,999697	-1,07	0,000817171	1,73	0,972396	-1,01	0,855926	1,05
bta-miR-148b_st	0,000837768	1,86	0,000118671	2,11	0,960366	-1,13	0,000966855	1,89	0,735266	-1,11	0,967676	1,02
bta-miR-15a_st	0,0575189	3,09	0,0223113	4,05	0,990861	-1,31	0,494055	1,81	0,18352	-2,24	0,383597	-1,71
bta-miR-181b_st	0,0128955	-1,67	0,028102	-1,65	0,999697	-1,01	0,00015813	-2,21	0,199842	-1,34	0,171735	-1,32
bta-miR-18b_st	0,00169406	3,09	0,000540787	3,64	0,992747	-1,18	0,00135327	3,30	0,896028	-1,10	0,928554	1,07
bta-miR-194_st	0,0387984	2,52	0,0340011	2,76	0,999697	-1,10	0,252586	1,89	0,521695	-1,46	0,590593	-1,33
bta-miR-199c_st	0,019523	1,55	0,00604322	1,70	0,990861	-1,10	0,00204652	1,78	0,908757	1,05	0,528299	1,15
bta-miR-19a_st	0,00367849	2,88	0,000289374	3,90	0,932209	-1,36	0,00054573	3,70	0,952733	-1,05	0,583402	1,29
bta-miR-22-3p_st	0,00870861	4,47	0,0422461	3,76	0,999697	1,19	0,00320826	5,60	0,650008	1,49	0,803473	1,25
bta-miR-2302_st	0,0100517	1,44	0,000788423	1,62	0,922285	-1,13	0,00963427	1,45	0,60389	-1,11	0,966496	1,01
bta-miR-2382_st	9,26E-06	2,72	5,12E-06	3,01	0,992747	-1,11	6,12E-05	2,66	0,743864	-1,13	0,96362	-1,02
bta-miR-2389_st	3,12E-05	1,63	2,90E-05	1,68	0,999697	-1,03	4,55E-05	1,71	0,954971	1,02	0,822179	1,05
bta-miR-2411_st	7,32E-10	5,15	1,09E-09	5,53	0,999697	-1,07	7,63E-09	5,06	0,857015	-1,09	0,974913	-1,02
bta-miR-2411-star_st	0,0247996	1,68	0,0453459	1,67	0,999697	1,01	0,111972	1,52	0,817918	-1,10	0,764827	-1,11
bta-miR-2430_st	0,00755467	3,17	0,0235582	2,97	0,999697	1,07	0,0306015	2,73	0,932373	-1,09	0,838329	-1,16
bta-miR-2887_st	0,113251	-1,60	0,0452082	-1,81	0,990861	1,13	0,41427	-1,37	0,38652	1,32	0,64282	1,17
bta-miR-2904_st	0,00327193	-1,98	0,00222169	-2,12	0,999697	1,07	0,0335694	-1,72	0,521695	1,23	0,641934	1,15
bta-miR-29c_st	0,0262959	1,63	0,00697193	1,84	0,978177	-1,13	0,00296903	1,91	0,934025	1,04	0,535332	1,17
bta-miR-301a_st	0,00619272	1,57	0,00300176	1,67	0,999697	-1,06	0,00326049	1,64	0,974796	-1,01	0,861521	1,05
bta-miR-30b-5p_st	0,166232	2,53	0,0215006	4,16	0,914635	-1,64	0,750629	1,42	0,0484567	-2,92	0,354424	-1,78
bta-miR-339b_st	0,0132005	2,60	0,0439057	2,41	0,999697	1,08	0,216356	1,83	0,633173	-1,32	0,418201	-1,42
bta-miR-379_st	0,0164774	2,29	0,0205401	2,38	0,999697	-1,04	0,0124445	2,42	0,985996	1,02	0,935614	1,06
bta-miR-382_st	0,00248618	2,77	0,0110398	2,55	0,999697	1,09	0,00226252	2,91	0,835507	1,14	0,944654	1,05
bta-miR-409_st	0,00467853	1,56	0,00367267	1,62	0,999697	-1,04	0,00463578	1,59	0,958598	-1,02	0,959353	1,02
bta-miR-411_st	0,00498732	1,63	0,00559119	1,67	0,999697	-1,03	0,00180464	1,75	0,900548	1,05	0,789964	1,07
bta-miR-431_st	0,0128897	1,91	0,015585	1,97	0,999697	-1,03	0,0959243	1,65	0,6467	-1,20	0,6645	-1,16
bta-miR-487b_st	0,083738	2,59	0,0399435	3,19	0,996817	-1,23	0,00907054	3,80	0,852471	1,19	0,516833	1,47
bta-miR-542-5p_st	0,0175716	1,42	0,00312526	1,56	0,958936	-1,10	0,0231258	1,41	0,666011	-1,10	0,99666	-1,00

bta-miR-654_st	0,00188112	1,55	0,00208008	1,58	0,999697	-1,02	0,00627718	1,49	0,829617	-1,06	0,888971	-1,04
cbr-miR-90_st	0,0029939	2,11	0,0257565	1,88	0,992099	1,12	0,00165758	2,27	0,63206	1,20	0,875663	1,07
cel-miR-1832_st	0,0169997	1,49	0,0267865	1,50	0,999697	-1,01	0,0367787	1,46	0,929982	-1,03	0,937655	-1,03
cfa-let-7b_st	9,26E-06	-1,54	8,57E-06	-1,58	0,999697	1,03	0,0188926	-1,27	0,017449	1,24	0,0247069	1,21
cfa-miR-142_st	0,0125114	1,80	0,0254478	1,78	0,999697	1,01	0,0283463	1,73	0,954971	-1,03	0,929597	-1,04
cfa-miR-148a_st	0,00307165	1,68	0,0085483	1,64	0,999697	1,02	0,00149096	1,78	0,808808	1,08	0,84783	1,06
cfa-miR-148b_st	0,000785957	1,86	0,00997874	1,69	0,990861	1,11	0,00136254	1,85	0,7749	1,10	0,988521	-1,01
cfa-miR-152_st	0,00333049	2,47	0,00612964	2,47	0,999697	-1,00	0,000576356	3,00	0,698291	1,21	0,624677	1,22
cfa-miR-15a_st	0,118542	2,63	0,0422461	3,50	0,990861	-1,33	0,657882	1,53	0,160894	-2,28	0,366354	-1,72
cfa-miR-1843_st	0,000175331	1,78	0,00103685	1,71	0,999697	1,04	0,000475509	1,76	0,933274	1,03	0,970881	-1,01
cfa-miR-18b_st	0,0213581	2,37	0,00471778	2,95	0,970567	-1,24	0,0123613	2,59	0,850053	-1,14	0,894556	1,09
cfa-miR-192_st	0,0441762	1,87	0,0441702	1,96	0,999697	-1,05	0,555209	1,33	0,241976	-1,48	0,264725	-1,41
cfa-miR-194_st	0,00922346	2,64	0,00416461	3,04	0,999697	-1,15	0,0359942	2,33	0,636089	-1,31	0,839949	-1,13
cfa-miR-19a_st	0,0213678	2,60	0,001686	3,74	0,907667	-1,44	0,00396233	3,31	0,880032	-1,13	0,649406	1,27
cfa-miR-21_st	0,00183778	2,89	0,000506694	3,42	0,990861	-1,18	0,000333389	3,56	0,962423	1,04	0,644919	1,23
cfa-miR-210_st	0,00125874	1,90	0,00158049	1,94	0,999697	-1,02	0,000958553	1,97	0,969941	1,02	0,914572	1,04
cfa-miR-224_st	0,00375564	1,95	0,022489	1,81	0,999697	1,08	0,00683745	1,92	0,905761	1,06	0,974918	-1,02
cfa-miR-29b_st	2,84E-05	1,67	0,000194924	1,62	0,999697	1,04	5,70E-05	1,73	0,753605	1,07	0,884542	1,03
cfa-miR-29c_st	0,00655921	1,70	0,0269176	1,62	0,999697	1,05	0,0050356	1,76	0,820304	1,08	0,927423	1,04
cfa-miR-301a_st	0,00125558	1,72	0,00219346	1,72	0,999697	-1,00	0,000872587	1,78	0,924945	1,04	0,903491	1,04
cfa-miR-331_st	0,0820557	1,61	0,0485225	1,75	0,999697	-1,09	0,103488	1,60	0,8449	-1,09	0,990312	-1,01
cfa-miR-370_st	0,000837768	1,96	0,0035414	1,87	0,999697	1,05	0,00122151	1,97	0,903503	1,05	0,992258	1,00
cfa-miR-379_st	0,00639699	2,17	0,00870286	2,23	0,999697	-1,03	0,0013372	2,55	0,785997	1,15	0,671161	1,18
cfa-miR-423a_st	0,0219206	-1,72	0,00567796	-1,96	0,980005	1,14	0,678682	-1,19	0,0193915	1,65	0,0880754	1,45
cfa-miR-487b_st	0,053187	2,95	0,0439874	3,35	0,999697	-1,13	0,008237	4,17	0,817404	1,25	0,602975	1,41
cgr-miR-21_st	0,000560976	3,10	0,000192956	3,57	0,996089	-1,15	0,000389047	3,36	0,932941	-1,06	0,893457	1,08
cin-miR-4031-5p_st	0,0299288	-1,83	0,0243001	-1,95	0,999697	1,07	0,770093	-1,17	0,0501021	1,67	0,0697875	1,56
cqu-miR-92_st	0,0183495	-1,46	0,017189	-1,50	0,999697	1,03	0,00368118	-1,59	0,841687	-1,06	0,666072	-1,09
csi-miR166_st	0,01067	1,71	0,00962427	1,79	0,999697	-1,04	0,00159808	1,97	0,801018	1,10	0,604842	1,15
dan-miR-210_st	0,0409884	1,55	0,0416484	1,60	0,999697	-1,04	0,0162037	1,67	0,932941	1,04	0,825684	1,08
dan-miR-92b_st	0,00248618	-2,28	0,00374534	-2,30	0,999697	1,01	0,00159808	-2,43	0,930999	-1,06	0,899338	-1,07
der-miR-210_st	0,0247018	1,51	0,0387056	1,52	0,999697	-1,01	0,0224237	1,54	0,979534	1,01	0,965974	1,02
der-miR-289_st	0,0051186	-2,68	0,00597046	-2,82	0,999697	1,05	0,782588	1,22	2,64E-05	3,45	2,49E-05	3,28
der-miR-92b_st	0,0219849	-1,97	0,0312669	-2,00	0,999697	1,02	0,0045375	-2,31	0,776262	-1,15	0,675938	-1,18
dgr-miR-92b_st	0,00493744	-1,93	0,00450559	-2,02	0,999697	1,05	0,00149848	-2,15	0,896528	-1,07	0,752347	-1,12
dme-miR-210_st	8,69E-06	2,05	2,17E-05	1,97	0,999697	1,04	0,000310173	1,78	0,680641	-1,11	0,430084	-1,15
dme-miR-289_st	0,0385766	-2,69	0,0225349	-3,15	0,999697	1,17	0,888155	1,17	0,000444138	3,67	0,00136225	3,14
dme-miR-92b_st	0,0387984	-1,73	0,040925	-1,80	0,999697	1,04	0,0147817	-1,90	0,926749	-1,05	0,818889	-1,10
dmo-miR-210_st	0,0176657	1,62	0,0331829	1,62	0,999697	1,00	0,056725	1,53	0,893787	-1,06	0,865333	-1,06
dmo-miR-92b_st	0,013905	-1,87	0,0155922	-1,94	0,999697	1,04	0,00428495	-2,08	0,884435	-1,08	0,765983	-1,12
dpe-miR-210_st	0,000175331	2,00	0,000402751	1,99	0,999697	1,01	0,000434557	1,98	0,995331	-1,00	0,98127	-1,01
dpe-miR-289_st	0,00372563	-3,06	0,00442501	-3,20	0,999697	1,05	0,981089	-1,03	0,000378988	3,11	0,000388059	2,97
dps-miR-210_st	0,000498966	1,93	0,00312526	1,81	0,999697	1,07	0,00407773	1,77	0,956128	-1,03	0,751856	-1,09
dps-miR-2532_st	0,0155755	1,68	0,00256244	1,94	0,953262	-1,16	0,014319	1,72	0,727917	-1,13	0,963357	1,02

dps-miR-289_st	0,0220692	-2,60	0,0313313	-2,67	0,999697	1,03	0,844066	1,20	0,000444138	3,21	0,000356211	3,12
dps-miR-92b_st	0,0181767	-2,32	0,02132	-2,44	0,999697	1,05	0,00822638	-2,60	0,932966	-1,07	0,847415	-1,12
dre-let-7b_st	2,29E-05	-1,54	2,30E-05	-1,57	0,999697	1,02	0,0359576	-1,27	0,0296593	1,24	0,0319309	1,21
dre-miR-101a_st	0,00245634	1,86	0,000208281	2,19	0,935558	-1,18	0,000483483	2,10	0,925664	-1,04	0,668737	1,13
dre-miR-126b-star_st	0,00338479	1,76	0,00222169	1,87	0,999697	-1,06	0,000576356	2,00	0,863381	1,07	0,616772	1,13
dre-miR-140_st	0,042078	1,68	0,0104151	1,95	0,970567	-1,16	0,0490626	1,69	0,728606	-1,15	0,99052	1,01
dre-miR-142a-5p_st	0,0324135	1,47	0,00873762	1,62	0,978332	-1,10	0,0146952	1,55	0,907712	-1,04	0,850612	1,06
dre-miR-181b_st	0,00248618	-1,87	0,00165911	-1,98	0,999697	1,06	1,73E-05	-2,63	0,233022	-1,33	0,0760487	-1,41
dre-miR-194a_st	0,0687198	2,23	0,035854	2,61	0,999697	-1,17	0,532887	1,50	0,221798	-1,74	0,378998	-1,49
dre-miR-19a_st	0,0148859	2,82	0,00138708	4,04	0,925823	-1,43	0,00478297	3,37	0,813341	-1,20	0,779489	1,20
dre-miR-21_st	0,000560976	1,74	0,000506694	1,79	0,999697	-1,03	0,00016033	1,88	0,879013	1,05	0,738323	1,08
dre-miR-22a_st	0,000883302	2,67	0,00721454	2,35	0,997807	1,13	0,00071031	2,83	0,70461	1,20	0,915329	1,06
dre-miR-22b_st	0,000837768	2,34	0,00180887	2,32	0,999697	1,01	0,000434557	2,54	0,852582	1,10	0,851359	1,09
dre-miR-29a_st	0,293652	1,43	0,0440173	1,81	0,903894	-1,27	0,165766	1,55	0,717044	-1,17	0,84783	1,09
dre-miR-30e_st	0,000307132	3,64	0,000226804	4,00	0,999697	-1,10	0,000475509	3,74	0,935204	-1,07	0,974913	1,03
dse-miR-289_st	0,0344543	-2,64	0,0243378	-2,99	0,999697	1,13	0,739656	1,33	0,000127975	3,98	0,000300947	3,51
dsi-miR-210_st	0,000170793	2,01	0,00222169	1,84	0,990861	1,10	0,0011478	1,89	0,953308	1,03	0,833851	-1,07
dsi-miR-92b_st	0,00526081	-2,07	0,00539682	-2,16	0,999697	1,05	0,00175952	-2,32	0,902428	-1,07	0,775391	-1,12
dvi-miR-210_st	0,0148859	1,73	0,0139567	1,81	0,999697	-1,05	0,0462827	1,63	0,783141	-1,11	0,878649	-1,06
dvi-miR-92b_st	0,0175283	-2,07	0,0151746	-2,22	0,999697	1,07	0,00296537	-2,51	0,826839	-1,13	0,62371	-1,21
dwi-miR-92b_st	0,00328034	-2,39	0,00870739	-2,33	0,999697	-1,03	0,00243908	-2,55	0,881004	-1,09	0,912884	-1,06
dya-miR-92b_st	0,015403	-1,73	0,0346607	-1,71	0,999697	-1,02	0,0045375	-1,92	0,755839	-1,13	0,752409	-1,11
eca-miR-134_st	0,0148859	2,34	0,0259619	2,35	0,999697	-1,00	0,0050356	2,70	0,826541	1,15	0,788457	1,15
eca-miR-140-5p_st	1,17E-06	2,59	4,37E-06	2,62	0,999697	-1,01	6,20E-05	2,26	0,59938	-1,16	0,549511	-1,15
eca-miR-142-5p_st	0,000589456	1,78	0,000208281	1,91	0,996817	-1,07	7,02E-05	2,05	0,832893	1,07	0,490922	1,15
eca-miR-148a_st	0,00212264	1,70	0,00156102	1,77	0,999697	-1,04	0,000310173	1,90	0,82047	1,07	0,600709	1,12
eca-miR-148b-3p_st	0,00253627	1,84	0,00319548	1,88	0,999697	-1,02	0,00467019	1,81	0,937631	-1,04	0,974215	-1,02
eca-miR-181b_st	0,00755467	-1,72	0,0103653	-1,75	0,999697	1,02	7,08E-05	-2,32	0,219785	-1,32	0,124995	-1,35
eca-miR-18b_st	0,0451049	2,82	0,0463375	3,04	0,999697	-1,08	0,396131	1,82	0,411523	-1,67	0,434326	-1,54
eca-miR-19a_st	0,000935214	2,96	1,55E-05	4,40	0,798366	-1,49	0,000108115	3,75	0,785997	-1,17	0,563789	1,27
eca-miR-21_st	1,42E-05	3,68	4,28E-05	3,56	0,999697	1,03	0,000104443	3,40	0,947713	-1,05	0,886567	-1,08
eca-miR-29b_st	0,0095811	1,48	0,00429968	1,56	0,999697	-1,06	0,00472304	1,55	0,973589	-1,01	0,8611	1,05
eca-miR-29c_st	0,0094409	1,78	0,00112471	2,11	0,935558	-1,18	0,000857477	2,13	0,987487	1,01	0,493338	1,20
eca-miR-301a_st	1,62E-05	1,78	8,57E-06	1,88	0,999697	-1,05	7,81E-06	1,94	0,92533	1,03	0,649507	1,08
eca-miR-30e_st	0,000186064	3,68	1,03E-05	5,13	0,901717	-1,39	0,000114378	4,15	0,720443	-1,24	0,845046	1,13
eca-miR-31_st	0,00248618	2,32	0,0422461	1,93	0,958936	1,21	0,000570084	2,71	0,315235	1,40	0,691848	1,17
eca-miR-331_st	0,0459236	1,59	0,0416267	1,67	0,999697	-1,05	0,500611	1,26	0,272928	-1,32	0,325831	-1,26
eca-miR-370_st	0,00188112	1,87	0,000473435	2,08	0,990861	-1,11	0,000242615	2,16	0,937402	1,04	0,579402	1,15
eca-miR-376c_st	0,00123247	1,65	0,000954848	1,71	0,999697	-1,04	0,000434668	1,76	0,933396	1,03	0,790147	1,07
eca-miR-382_st	0,00248618	2,71	0,0240233	2,30	0,990861	1,18	0,00159808	2,93	0,635053	1,27	0,898493	1,08
eca-miR-409-3p_st	0,00495794	3,83	0,026944	3,29	0,999697	1,16	0,000576356	5,48	0,407197	1,67	0,54374	1,43
eca-miR-423-5p_st	0,063039	-1,54	0,00579253	-1,86	0,912872	1,21	0,679368	-1,18	0,0196504	1,58	0,213622	1,31
eca-miR-431_st	0,00619272	2,09	0,0125101	2,08	0,999697	1,00	0,00607711	2,15	0,962602	1,03	0,965794	1,03
eca-miR-503_st	0,344464	1,33	0,0463375	1,66	0,883835	-1,25	0,0338997	1,67	0,997459	1,00	0,36817	1,25

eca-miR-885-3p_st	0,0101306	1,67	0,00717081	1,76	0,999697	-1,06	0,0206502	1,62	0,820168	-1,09	0,94463	-1,03
ENSG00000202252_st	0,0848691	-1,52	0,0442746	-1,65	0,999697	1,09	0,0382519	-1,63	0,984975	1,01	0,840071	-1,08
ENSG00000206785_s_s	0,0403874	1,80	0,0263758	1,96	0,999697	-1,09	0,689772	1,22	0,08912	-1,60	0,145749	-1,47
ENSG00000212378_s_s	0,00248618	-1,80	0,0119724	-1,71	0,999697	-1,05	0,0268226	-1,60	0,855981	1,07	0,637235	1,13
ENSG00000252921_st	0,0212005	-1,51	0,040925	-1,51	0,999697	-1,00	0,00816336	-1,62	0,827545	-1,07	0,818457	-1,07
ENSG00000253014_st	0,118723	-1,50	0,0395869	-1,70	0,988258	1,13	0,0143203	-1,78	0,926067	-1,05	0,526476	-1,19
fru-let-7b_st	3,04E-05	-1,49	1,92E-05	-1,54	0,999697	1,03	0,0615601	-1,23	0,0108276	1,25	0,0222001	1,21
fru-miR-101a_st	0,0584544	1,44	0,0387056	1,52	0,999697	-1,06	0,00751326	1,64	0,825815	1,07	0,563086	1,14
fru-miR-140_st	0,00208313	2,08	0,00149881	2,22	0,999697	-1,06	0,00788579	1,94	0,742493	-1,14	0,862388	-1,07
fru-miR-142a_st	0,000974877	1,72	0,0027068	1,69	0,999697	1,02	0,000734814	1,78	0,868085	1,06	0,910396	1,04
fru-miR-142b_st	0,0167593	1,48	0,00997874	1,56	0,999697	-1,06	0,0161566	1,50	0,907763	-1,04	0,969231	1,01
fru-miR-181b_st	0,0101809	-1,75	0,00396819	-1,93	0,996089	1,10	6,20E-05	-2,53	0,281603	-1,32	0,0642411	-1,44
fru-miR-194_st	0,0589419	2,50	0,0347566	2,92	0,999697	-1,17	0,34224	1,82	0,408097	-1,60	0,566662	-1,37
fru-miR-19a_st	0,0039422	3,38	0,00149881	4,11	0,990861	-1,22	0,00114894	4,16	0,991314	1,01	0,734431	1,23
fru-miR-21_st	0,00579416	1,69	0,0153617	1,66	0,999697	1,02	0,0591381	1,50	0,752907	-1,11	0,626346	-1,13
fru-miR-210_st	0,0128541	1,74	0,0223147	1,75	0,999697	-1,00	0,0016757	2,03	0,668807	1,16	0,580434	1,16
fru-miR-22a_st	8,53E-05	2,75	0,000597853	2,53	0,999697	1,09	0,000139316	2,80	0,832574	1,11	0,974894	1,02
fru-miR-22b_st	0,000403433	2,13	0,000954848	2,11	0,999697	1,01	0,000434557	2,20	0,932934	1,04	0,942506	1,03
gga-let-7b_st	9,26E-06	-1,54	8,57E-06	-1,57	0,999697	1,02	0,0231258	-1,26	0,0141539	1,24	0,0183999	1,21
gga-miR-1306_st	0,40675	1,21	0,0165088	1,53	0,651692	-1,26	0,191923	1,30	0,460466	-1,17	0,753364	1,08
gga-miR-142-5p_st	0,0191829	3,52	0,00899352	4,35	0,999697	-1,23	0,0485698	3,15	0,70532	-1,38	0,908978	-1,12
gga-miR-1466_st	0,000179166	2,33	0,000485758	2,29	0,999697	1,02	7,02E-05	2,62	0,72579	1,15	0,71361	1,13
gga-miR-148a_st	0,0982919	1,39	0,0231743	1,55	0,964417	-1,12	0,0208046	1,53	0,974579	-1,01	0,667112	1,11
gga-miR-1583_st	0,0125089	2,29	0,00241622	2,81	0,970327	-1,23	0,00665641	2,50	0,845121	-1,13	0,88072	1,09
gga-miR-15a_st	0,0637552	2,82	0,00539682	4,53	0,904873	-1,60	0,623819	1,55	0,0261636	-2,92	0,265175	-1,82
gga-miR-1610_st	0,000175558	2,71	0,000591927	2,62	0,999697	1,04	0,000154178	2,89	0,849338	1,10	0,901494	1,06
gga-miR-1611_st	0,139015	2,20	0,0439057	2,87	0,974307	-1,30	0,0485501	2,70	0,957063	-1,06	0,761667	1,23
gga-miR-1759_st	0,0523691	1,49	0,0204216	1,64	0,990861	-1,10	0,0373565	1,55	0,884587	-1,06	0,923933	1,04
gga-miR-181b_st	0,0605761	-1,68	0,0156036	-1,97	0,970567	1,17	0,000333064	-2,57	0,417918	-1,31	0,0736436	-1,53
gga-miR-18b_st	0,00137931	2,88	0,000132305	3,71	0,935558	-1,29	0,00193462	2,89	0,622638	-1,28	0,995959	1,00
gga-miR-199-star_st	0,030576	1,48	0,0243001	1,55	0,999697	-1,04	0,00822638	1,61	0,918785	1,04	0,738148	1,09
gga-miR-19a_st	0,0166547	2,65	0,00230831	3,56	0,935558	-1,34	0,00152256	3,66	0,978458	1,03	0,494836	1,38
gga-miR-21_st	0,000866558	3,44	0,000485758	3,85	0,999697	-1,12	0,0013372	3,45	0,885639	-1,12	0,998056	1,00
gga-miR-29b_st	0,000755209	1,61	0,00174162	1,60	0,999697	1,01	0,000180676	1,73	0,729951	1,09	0,702724	1,08
gga-miR-301b-3p_st	0,00328957	1,63	0,00701056	1,63	0,999697	1,01	0,0317277	1,48	0,745349	-1,10	0,66347	-1,10
gga-miR-30b_st	0,27475	2,22	0,0259616	4,02	0,817193	-1,81	0,875363	1,23	0,0232277	-3,26	0,332115	-1,80
gga-miR-3537_st	0,153997	1,57	0,0495384	1,83	0,977103	-1,17	0,316212	1,45	0,525248	-1,26	0,855335	-1,09
gga-miR-449b-star_st	0,0334609	1,99	0,0387056	2,07	0,999697	-1,04	0,0408609	2,00	0,962136	-1,04	0,995736	1,00
ggo-miR-101_st	0,000227987	1,90	0,000433438	1,91	0,999697	-1,01	0,000107991	2,05	0,832207	1,07	0,784739	1,08
ggo-miR-181b_st	0,00833961	-1,74	0,0123792	-1,77	0,999697	1,01	0,000108115	-2,30	0,282152	-1,30	0,184502	-1,32
ggo-miR-19a_st	0,00730824	2,74	0,000247035	4,07	0,818077	-1,49	0,000389047	3,92	0,969228	-1,04	0,397947	1,43
ggo-miR-21_st	0,00493744	2,91	0,00278938	3,31	0,999697	-1,14	0,012174	2,72	0,765536	-1,22	0,929136	-1,07
ggo-miR-224_st	0,00367849	1,98	0,026944	1,80	0,999697	1,10	0,00238862	2,10	0,6835	1,17	0,892752	1,06
ggo-miR-30b_st	0,113025	2,23	0,0138518	3,26	0,923668	-1,46	0,2234	2,00	0,373463	-1,63	0,892752	-1,11

gma-miR166b_st	0,014103	1,62	0,00192347	1,87	0,935558	-1,15	0,000241588	2,07	0,753605	1,11	0,206837	1,28
HBII-180A_x_st	0,000358502	-2,31	0,00202241	-2,17	0,999697	-1,07	0,000138	-2,56	0,649497	-1,18	0,773604	-1,11
HBII-202_st	0,0400874	-1,48	0,0141603	-1,62	0,990861	1,09	0,316306	-1,28	0,266647	1,26	0,510623	1,15
HBII-289_st	0,0411363	-1,65	0,00636403	-1,95	0,939148	1,18	0,38446	-1,34	0,120963	1,46	0,427728	1,23
HBII-429_st	0,0261249	-1,52	0,0203002	-1,59	0,999697	1,05	0,00396233	-1,71	0,826568	-1,08	0,603338	-1,13
HBII-55_st	0,00424296	-1,90	0,00331017	-2,00	0,999697	1,06	0,0013372	-2,10	0,926067	-1,05	0,756982	-1,11
HBII-85-2_x_st	0,0191829	-1,48	0,0235582	-1,51	0,999697	1,02	0,0727868	-1,40	0,786049	1,08	0,83119	1,06
hiv1-miR-H1_st	0,0148859	1,73	0,0422461	1,67	0,999697	1,03	0,0176508	1,74	0,940765	1,04	0,990035	1,01
hp_mmu-mir-142_st	0,00427633	1,56	0,00392405	1,61	0,999697	-1,03	0,111624	1,34	0,349984	-1,20	0,404888	-1,16
hsa-let-7b_st	1,62E-05	-1,52	1,56E-05	-1,55	0,999697	1,02	0,0200458	-1,28	0,0382936	1,22	0,048143	1,19
hsa-miR-106b-star_st	0,0990713	-1,78	0,0145727	-2,29	0,935558	1,28	0,0124461	-2,25	0,979743	1,02	0,544774	-1,26
hsa-miR-1224-5p_st	0,142643	1,40	0,0316607	1,60	0,953262	-1,14	0,232675	1,35	0,534966	-1,18	0,929728	-1,03
hsa-miR-1231_st	0,00012432	2,54	0,000132305	2,64	0,999697	-1,04	0,0203594	1,84	0,186236	-1,44	0,190425	-1,38
hsa-miR-1275_st	0,00427633	-2,13	0,0165088	-2,02	0,999697	-1,05	0,10675	-1,66	0,631693	1,22	0,415405	1,28
hsa-miR-1308_st	0,228541	-1,50	0,0164469	-2,03	0,817193	1,35	0,0209362	-1,93	0,932941	1,05	0,413792	-1,28
hsa-miR-134_st	0,0051186	2,34	0,00502487	2,47	0,999697	-1,06	0,00204434	2,63	0,928381	1,06	0,809776	1,13
hsa-miR-140-5p_st	6,08E-05	2,36	2,17E-05	2,60	0,993631	-1,10	0,000104443	2,42	0,867379	-1,08	0,962759	1,02
hsa-miR-142-5p_st	0,00188112	1,83	0,00198535	1,88	0,999697	-1,03	0,000107991	2,19	0,60389	1,16	0,412679	1,20
hsa-miR-148b_st	0,000176203	2,05	0,000208281	2,10	0,999697	-1,03	0,00071433	1,97	0,871246	-1,06	0,923296	-1,04
hsa-miR-152_st	0,00752451	2,39	0,039164	2,16	0,999697	1,11	0,00498246	2,56	0,753935	1,19	0,908233	1,07
hsa-miR-181b_st	0,00738454	-1,74	0,00800097	-1,79	0,999697	1,03	6,57E-05	-2,38	0,223007	-1,33	0,10884	-1,37
hsa-miR-185-star_st	0,00272173	1,45	0,0015572	1,51	0,999697	-1,04	0,00616972	1,43	0,786049	-1,06	0,941467	-1,02
hsa-miR-1909_st	0,0430474	2,09	0,0477593	2,19	0,999697	-1,05	0,125866	1,88	0,798046	-1,17	0,849964	-1,12
hsa-miR-196b-star_st	0,0170038	2,62	0,0167748	2,83	0,999697	-1,08	0,0629769	2,30	0,756306	-1,23	0,839949	-1,14
hsa-miR-19a_st	0,0409826	2,34	0,00423878	3,24	0,931552	-1,39	0,00362413	3,24	0,999537	-1,00	0,48124	1,38
hsa-miR-21_st	0,00328957	3,06	0,000621952	3,88	0,970567	-1,27	0,00135286	3,54	0,914918	-1,10	0,813799	1,16
hsa-miR-210_st	0,000589456	2,16	0,00441627	1,98	0,999697	1,09	0,0173506	1,77	0,782114	-1,12	0,452076	-1,22
hsa-miR-24-2-star_st	0,00179562	1,91	0,00240335	1,95	0,999697	-1,02	0,0206387	1,67	0,638955	-1,16	0,620313	-1,14
hsa-miR-29b_st	0,000208803	1,52	0,000319276	1,54	0,999697	-1,01	0,000105021	1,60	0,862464	1,04	0,790147	1,05
hsa-miR-29c_st	0,0431247	1,65	0,0148687	1,85	0,990861	-1,13	0,0268226	1,73	0,888486	-1,07	0,90264	1,05
hsa-miR-301a_st	3,58E-05	1,97	0,000289374	1,86	0,999697	1,06	7,08E-05	2,01	0,798046	1,08	0,950224	1,02
hsa-miR-30e_st	0,00579624	2,92	0,00124114	3,71	0,970567	-1,27	0,0045375	3,13	0,811891	-1,18	0,926168	1,07
hsa-miR-3180-3p_st	0,00328034	2,51	0,00721454	2,47	0,999697	1,01	0,00369243	2,57	0,958627	1,04	0,971093	1,03
hsa-miR-320d_st	0,0386541	-1,39	0,0122256	-1,51	0,990861	1,08	0,00226252	-1,61	0,820111	-1,06	0,404532	-1,15
hsa-miR-361-5p_st	0,142495	-1,36	0,0453459	-1,50	0,977103	1,11	0,0292632	-1,52	0,984975	-1,01	0,632996	-1,12
hsa-miR-370_st	0,000420831	2,03	0,00278938	1,90	0,999697	1,07	0,00128299	1,97	0,937981	1,04	0,939119	-1,03
hsa-miR-376c_st	0,00125558	1,60	0,00278938	1,58	0,999697	1,01	0,00181448	1,60	0,977616	1,01	0,998694	1,00
hsa-miR-379_st	0,0030472	2,46	0,00210183	2,68	0,999697	-1,09	0,000872587	2,85	0,92962	1,06	0,738541	1,16
hsa-miR-382_st	0,0122187	2,56	0,0422461	2,37	0,999697	1,08	0,00697927	2,80	0,798046	1,18	0,892752	1,10
hsa-miR-4298_st	0,00112021	-2,66	0,00115499	-2,79	0,999697	1,05	0,00625163	-2,36	0,743297	1,18	0,798468	1,13
hsa-miR-431_st	0,00137931	2,22	0,00181058	2,27	0,999697	-1,02	0,00465474	2,08	0,864628	-1,09	0,892557	-1,06
hsa-miR-4310_st	0,000982626	1,60	0,000230014	1,72	0,990861	-1,08	0,000242615	1,72	0,996719	1,00	0,71361	1,08
hsa-miR-432_st	0,0138925	3,20	0,0442746	2,94	0,999697	1,09	0,00196846	4,38	0,543774	1,49	0,594531	1,37
hsa-miR-433_st	0,00702644	1,50	0,00274866	1,60	0,996089	-1,07	0,00338924	1,57	0,96368	-1,02	0,84856	1,05

hsa-miR-501-3p_st	0,0496376	1,72	0,0497376	1,77	0,999697	-1,03	0,00654792	2,08	0,703086	1,18	0,547688	1,21
hsa-miR-503_st	0,210207	1,79	0,0236724	2,56	0,894908	-1,43	0,0151977	2,59	0,991321	1,01	0,379495	1,45
hsa-miR-885-3p_st	0,000752771	1,75	0,0308761	1,50	0,914635	1,16	0,0118194	1,55	0,932272	1,03	0,571884	-1,12
hsa-miR-92a-1-star_st	0,00784987	-3,49	0,0371251	-3,05	0,999697	-1,15	0,00252584	-4,29	0,634616	-1,41	0,773889	-1,23
hsa-miR-92b_st	0,193189	-1,69	0,049956	-2,10	0,954992	1,25	0,0516988	-2,03	0,966224	1,03	0,670968	-1,20
hsa-miR-939_st	0,0277072	1,65	0,0495877	1,64	0,999697	1,01	0,011367	1,78	0,835086	1,09	0,835219	1,08
hvt-miR-H1_st	0,00372563	2,18	0,00298537	2,33	0,999697	-1,07	0,00380362	2,25	0,958722	-1,04	0,957361	1,03
hvt-miR-H14-star_st	0,0316574	1,73	0,0316216	1,81	0,999697	-1,04	0,260031	1,44	0,495994	-1,26	0,537395	-1,20
lca-miR-19a_st	0,000144472	3,75	1,81E-05	4,78	0,953262	-1,27	6,20E-05	4,55	0,952733	-1,05	0,692397	1,21
lla-miR-101_st	0,0102161	1,71	0,0439874	1,61	0,999697	1,06	0,0373565	1,60	0,985566	-1,01	0,850337	-1,07
lla-miR-181b_st	0,0086197	-1,74	0,013053	-1,77	0,999697	1,01	0,000190968	-2,24	0,357363	-1,27	0,249812	-1,29
lla-miR-19a_st	0,000704341	3,34	0,000132137	4,13	0,974307	-1,23	0,000343073	3,77	0,909168	-1,09	0,841057	1,13
lla-miR-30b_st	0,102217	2,18	0,0035414	3,60	0,792664	-1,65	0,0938953	2,27	0,379016	-1,59	0,966215	1,04
mcmv-miR-M23-2_st	1,31E-06	2,19	5,04E-06	2,18	0,999697	1,01	4,24E-07	2,37	0,748403	1,09	0,728899	1,08
mdo-let-7b_st	9,26E-06	-1,54	8,23E-06	-1,58	0,999697	1,03	0,0159896	-1,28	0,0194297	1,24	0,0309338	1,20
mdo-miR-140_st	0,000119486	2,74	3,85E-05	3,07	0,996817	-1,12	0,000389047	2,64	0,741036	-1,16	0,946116	-1,04
mdo-miR-15a_st	0,0179708	2,72	0,00811613	3,22	0,999697	-1,18	0,500684	1,55	0,0822661	-2,08	0,173116	-1,75
mdo-miR-181b_st	0,00137931	-1,91	0,000985789	-2,01	0,999697	1,05	7,81E-06	-2,68	0,201576	-1,34	0,0703961	-1,41
mdo-miR-19a_st	0,00898197	2,54	0,000129509	4,03	0,743481	-1,59	0,000630296	3,46	0,812843	-1,16	0,44952	1,36
mdo-miR-21_st	4,56E-05	3,87	0,000222505	3,62	0,999697	1,07	0,000219759	3,65	0,992112	1,01	0,928575	-1,06
mdo-miR-301_st	0,00099096	1,87	0,00242369	1,84	0,999697	1,02	0,000576356	1,97	0,847004	1,07	0,873799	1,06
mdo-miR-739_st	0,00272173	-1,87	0,000865557	-2,06	0,990861	1,10	0,165073	-1,43	0,0920726	1,44	0,219097	1,30
mml-let-7b_st	9,26E-06	-1,53	7,51E-06	-1,58	0,999697	1,04	0,0109634	-1,29	0,0228763	1,23	0,0523914	1,18
mml-miR-101_st	0,00099096	1,96	0,000256834	2,16	0,990861	-1,10	0,000278517	2,17	0,997854	1,00	0,734619	1,11
mml-miR-134_st	0,0220863	2,25	0,0461488	2,20	0,999697	1,02	0,00399194	2,77	0,669458	1,26	0,643055	1,23
mml-miR-140-5p_st	7,42E-05	2,34	3,45E-05	2,53	0,999697	-1,08	0,000168328	2,33	0,842722	-1,08	0,99586	-1,00
mml-miR-142-5p_st	0,000729078	1,79	0,00267732	1,73	0,999697	1,03	0,000349622	1,90	0,756457	1,10	0,839949	1,06
mml-miR-148b_st	0,00114414	1,82	0,00103685	1,88	0,999697	-1,04	0,000576356	1,93	0,959608	1,02	0,85362	1,06
mml-miR-152_st	0,00626582	2,34	0,023635	2,20	0,999697	1,07	0,00125846	2,81	0,598014	1,28	0,663741	1,20
mml-miR-181b_st	0,00496248	-1,76	0,0116664	-1,74	0,999697	-1,01	8,99E-05	-2,28	0,242435	-1,31	0,204039	-1,30
mml-miR-19a_st	0,000197489	3,29	7,18E-05	3,81	0,992747	-1,16	0,000589599	3,18	0,746461	-1,20	0,965794	-1,03
mml-miR-21_st	0,00137931	3,38	0,000436116	4,02	0,992747	-1,19	0,000529118	3,95	0,98569	-1,02	0,798468	1,17
mml-miR-224_st	0,029335	1,57	0,042635	1,58	0,999697	-1,01	0,0725644	1,50	0,893787	-1,06	0,903454	-1,05
mml-miR-301a_st	0,000153906	1,69	7,76E-05	1,77	0,999697	-1,05	0,000411601	1,67	0,81712	-1,06	0,971023	-1,01
mml-miR-30b_st	0,0180173	2,76	0,00367267	3,59	0,970327	-1,30	0,0476407	2,51	0,548476	-1,43	0,903454	-1,10
mml-miR-30e_st	0,00114998	3,73	9,19E-05	5,18	0,935558	-1,39	0,000797037	4,11	0,736767	-1,26	0,897573	1,10
mml-miR-337-5p_st	0,000507844	1,60	0,00117951	1,59	0,999697	1,01	0,000335604	1,66	0,876147	1,04	0,881886	1,04
mml-miR-370_st	0,00170341	1,88	0,013053	1,73	0,999697	1,08	0,0103781	1,72	0,989349	-1,01	0,773889	-1,09
mml-miR-379_st	0,00830552	2,35	0,000507684	3,16	0,906126	-1,34	0,000702954	3,07	0,973589	-1,03	0,479996	1,31
mml-miR-409-5p_st	0,0225776	1,48	0,015585	1,56	0,999697	-1,05	0,0147817	1,53	0,97099	-1,02	0,914152	1,03
mml-miR-431_st	0,006075	2,02	0,00396819	2,18	0,999697	-1,08	0,00593544	2,08	0,934294	-1,05	0,964573	1,03
mml-miR-487b_st	0,0255324	3,09	0,0405398	3,14	0,999697	-1,02	0,00261907	4,50	0,626166	1,43	0,519319	1,46
mml-miR-503_st	0,151205	1,83	0,02023	2,48	0,924888	-1,36	0,00273914	2,96	0,766114	1,20	0,168207	1,62
mml-miR-885-3p_st	0,0225776	1,48	0,0244912	1,52	0,999697	-1,03	0,00822638	1,58	0,917919	1,04	0,80866	1,07

mml-miR-92b_st	0,0195335	-2,32	0,00604322	-2,78	0,990861	1,20	0,00909336	-2,59	0,929224	1,07	0,855058	-1,12
mmu-let-7b_st	9,26E-06	-1,55	8,23E-06	-1,60	0,999697	1,03	0,0090506	-1,31	0,0366495	1,22	0,0636953	1,18
mmu-let-7e_st	0,00251308	-2,27	0,0276769	-1,96	0,990861	-1,15	0,187731	-1,58	0,584617	1,25	0,183342	1,44
mmu-miR-140_st	0,0184954	1,68	0,0243616	1,71	0,999697	-1,02	0,0638007	1,57	0,820332	-1,09	0,851559	-1,07
mmu-miR-142-5p_st	0,0051186	1,88	0,00332174	2,02	0,999697	-1,07	0,00307578	2,00	0,989997	-1,01	0,879268	1,06
mmu-miR-148a_st	0,0122535	1,55	0,0263758	1,54	0,999697	1,01	0,00638799	1,63	0,865578	1,06	0,876051	1,05
mmu-miR-148b_st	0,000126225	2,07	0,000473629	2,01	0,999697	1,03	0,000209334	2,10	0,919458	1,05	0,979992	1,01
mmu-miR-152_st	0,00338479	2,57	0,0119724	2,44	0,999697	1,06	0,00904498	2,42	0,99511	-1,00	0,925856	-1,06
mmu-miR-181b_st	0,0148859	-1,66	0,0118596	-1,75	0,999697	1,05	8,99E-05	-2,31	0,238132	-1,32	0,0882826	-1,39
mmu-miR-1894-3p_st	0,129268	1,51	0,0367515	1,75	0,970567	-1,15	0,178426	1,48	0,651749	-1,18	0,969231	-1,02
mmu-miR-194_st	0,0109997	3,05	0,016368	3,14	0,999697	-1,03	0,187981	2,05	0,465313	-1,53	0,423702	-1,48
mmu-miR-1951_st	0,0332107	1,57	0,015585	1,71	0,997837	-1,09	0,0156693	1,67	0,964664	-1,02	0,859027	1,06
mmu-miR-19a_st	0,00440707	2,73	0,000137351	4,02	0,817193	-1,47	0,00103605	3,32	0,754901	-1,21	0,686871	1,22
mmu-miR-21_st	2,44E-05	3,93	0,000166893	3,60	0,999697	1,09	6,20E-05	4,15	0,812084	1,15	0,933411	1,06
mmu-miR-2135_st	0,000574224	-2,46	0,000726039	-2,52	0,999697	1,02	0,627373	-1,25	0,00170499	2,01	0,00130517	1,96
mmu-miR-2140_st	4,87E-05	-3,73	1,43E-05	-4,43	0,990861	1,19	0,880715	-1,13	1,45E-06	3,93	5,59E-06	3,31
mmu-miR-2141_st	0,030652	-2,68	0,0263758	-2,96	0,999697	1,10	0,251371	-1,94	0,471819	1,53	0,541211	1,39
mmu-miR-2146_st	0,00956076	-1,85	0,00421701	-2,03	0,999697	1,10	0,369711	-1,36	0,0949324	1,50	0,184502	1,37
mmu-miR-291b-5p_st	0,0377451	2,10	0,0133802	2,49	0,990861	-1,18	0,00607711	2,60	0,956794	1,05	0,623467	1,24
mmu-miR-297c-star_st	0,0460385	1,63	0,0450761	1,70	0,999697	-1,04	0,0950368	1,56	0,852317	-1,09	0,927423	-1,04
mmu-miR-29b_st	2,44E-05	1,79	2,90E-05	1,83	0,999697	-1,02	0,00015813	1,73	0,829839	-1,06	0,891935	-1,04
mmu-miR-29c_st	0,000186064	2,20	0,000112731	2,37	0,999697	-1,08	6,57E-05	2,49	0,913233	1,05	0,667112	1,13
mmu-miR-301a_st	0,0236805	1,51	0,0304336	1,53	0,999697	-1,02	0,0106095	1,59	0,922488	1,04	0,850337	1,06
mmu-miR-30e_st	0,00153777	3,10	6,59E-05	4,38	0,894908	-1,41	0,00136254	3,26	0,577154	-1,34	0,944014	1,05
mmu-miR-326_st	0,000129753	1,63	0,00239815	1,51	0,974987	1,08	0,000455324	1,60	0,815178	1,06	0,939349	-1,02
mmu-miR-370_st	9,13E-05	2,07	0,000215593	2,05	0,999697	1,01	3,25E-05	2,35	0,642462	1,15	0,597769	1,14
mmu-miR-379_st	0,0393451	2,09	0,0269176	2,31	0,999697	-1,10	0,00686225	2,58	0,872647	1,11	0,641058	1,23
mmu-miR-409-3p_st	0,00485451	3,96	0,0245607	3,43	0,999697	1,15	0,00183146	4,81	0,659109	1,40	0,803473	1,21
mmu-miR-409-5p_st	0,0147431	1,48	0,00717081	1,58	0,999697	-1,06	0,00839513	1,54	0,95636	-1,02	0,890131	1,04
mmu-miR-423-5p_st	0,0804483	-1,58	0,00338537	-2,06	0,817193	1,30	0,514267	-1,28	0,0325726	1,61	0,427732	1,24
mmu-miR-431_st	0,00570583	2,03	0,00728728	2,08	0,999697	-1,03	0,00395119	2,14	0,963763	1,03	0,904904	1,06
mmu-miR-451_st	0,0102661	2,04	0,0302047	1,96	0,999697	1,04	0,00165819	2,42	0,603011	1,24	0,624211	1,19
mmu-miR-487b_st	0,0355386	2,80	0,0329828	3,08	0,999697	-1,10	0,00105751	4,79	0,484849	1,56	0,277718	1,71
mmu-miR-503_st	0,00646895	1,76	0,0193361	1,72	0,999697	1,03	0,00904498	1,76	0,958414	1,03	0,998011	-1,00
mmu-miR-551b_st	0,369615	1,50	0,0422461	2,12	0,817193	-1,42	0,712501	1,26	0,125033	-1,69	0,690642	-1,19
mmu-miR-714_st	0,00333049	2,83	0,00158049	3,26	0,999697	-1,15	0,016904	2,47	0,60389	-1,32	0,809848	-1,15
mmu-miR-92b_st	0,0125829	-2,17	0,0371046	-2,07	0,999697	-1,05	0,00546307	-2,40	0,781201	-1,16	0,84368	-1,11
mne-miR-101_st	3,03E-05	2,16	3,61E-05	2,22	0,999697	-1,03	5,38E-05	2,29	0,949362	1,03	0,855335	1,06
mne-miR-181b_st	0,00721384	-1,75	0,0116025	-1,77	0,999697	1,01	0,00012598	-2,28	0,317173	-1,29	0,217785	-1,30
mne-miR-19a_st	0,00149598	3,19	2,59E-05	4,91	0,801344	-1,54	0,000236237	4,03	0,75267	-1,22	0,620424	1,26
mne-miR-21_st	0,00248618	3,08	0,00396819	3,10	0,999697	-1,01	0,000958553	3,56	0,845608	1,15	0,80879	1,16
mne-miR-30b_st	0,0357144	2,63	0,001686	4,20	0,817193	-1,60	0,0206387	2,93	0,569652	-1,43	0,893572	1,11
mtr-miR166g_st	0,00149598	1,74	0,00500598	1,69	0,999697	1,03	7,58E-06	2,37	0,0482178	1,40	0,0532143	1,36
mtr-miR2616_st	0,00114366	4,57	0,000108548	6,56	0,935558	-1,44	0,00696832	3,73	0,324903	-1,76	0,77861	-1,22

nvi-miR-210_st	0,000296585	1,91	0,00329235	1,76	0,992747	1,09	0,00537505	1,69	0,925623	-1,04	0,602975	-1,13
oan-let-7b_st	1,42E-05	-1,54	9,68E-06	-1,58	0,999697	1,03	0,021675	-1,28	0,0207945	1,24	0,034616	1,20
oan-miR-1386_st	0,000183421	4,15	0,000256834	4,29	0,999697	-1,03	0,0373565	2,43	0,188095	-1,77	0,168303	-1,71
oan-miR-1415_st	0,000175331	1,70	0,000132305	1,77	0,999697	-1,04	0,000576356	1,67	0,835215	-1,06	0,948153	-1,02
oan-miR-142_st	0,0603377	1,41	0,00811613	1,60	0,935558	-1,14	0,0102784	1,56	0,94501	-1,03	0,624973	1,11
oan-miR-1421ag_st	0,00642151	1,50	0,00425576	1,57	0,999697	-1,04	0,00183146	1,61	0,931743	1,03	0,745568	1,07
oan-miR-1421ai-star_st	0,096857	1,33	0,0148687	1,50	0,935558	-1,13	0,119143	1,33	0,580885	-1,13	0,992575	-1,00
oan-miR-148_st	0,112147	1,48	0,0155129	1,77	0,932209	-1,19	0,0125263	1,75	0,98569	-1,01	0,514528	1,18
oan-miR-18_st	0,0356667	2,34	0,0138518	2,80	0,990861	-1,20	0,0414963	2,36	0,802107	-1,19	0,990312	1,01
oan-miR-181b_st	0,00382133	-1,79	0,00660301	-1,80	0,999697	1,01	6,44E-05	-2,37	0,233232	-1,32	0,163634	-1,33
oan-miR-194_st	0,0487851	2,51	0,0294596	2,91	0,999697	-1,16	0,354722	1,77	0,359201	-1,64	0,50342	-1,41
oan-miR-196b-star_st	0,0214123	2,59	0,0366389	2,61	0,999697	-1,00	0,060402	2,34	0,898241	-1,11	0,88662	-1,11
oan-miR-199-star_st	0,00206551	2,40	0,0217171	2,08	0,990861	1,16	0,00186397	2,49	0,692009	1,20	0,951438	1,04
oan-miR-19a_st	0,0112761	2,55	0,000132137	4,20	0,707584	-1,65	0,00167376	3,24	0,644977	-1,30	0,606557	1,27
oan-miR-21_st	0,000589456	3,30	0,000256834	3,73	0,999697	-1,13	0,00035429	3,64	0,978179	-1,02	0,873799	1,10
oan-miR-451_st	0,0140195	2,80	0,0230843	2,85	0,999697	-1,02	0,00427326	3,38	0,820849	1,19	0,763751	1,20
oar-miR-431_st	0,0016504	2,25	0,0058835	2,14	0,999697	1,05	0,00192082	2,29	0,904146	1,07	0,977433	1,02
odi-miR-92a_st	0,0427103	-2,00	0,0149512	-2,36	0,990861	1,18	0,00558426	-2,51	0,932373	-1,06	0,573158	-1,25
osa-miR166b_st	0,00207193	1,78	0,00800097	1,71	0,999697	1,04	8,99E-05	2,16	0,301885	1,26	0,362484	1,21
osa-miR394_st	0,0617649	1,63	0,010482	1,95	0,938111	-1,19	0,00829134	1,93	0,989997	-1,01	0,568635	1,19
peu-miR2910_st	0,0022072	-2,42	0,00185866	-2,57	0,999697	1,06	0,395575	-1,43	0,0366265	1,79	0,0437184	1,69
ppa-miR-101_st	0,00125558	1,85	0,000876554	1,94	0,999697	-1,05	0,000139521	2,13	0,785997	1,10	0,555884	1,15
ppa-miR-134_st	0,0168341	2,11	0,00736196	2,40	0,997231	-1,14	0,00138813	2,72	0,820304	1,14	0,479713	1,29
ppa-miR-181b_st	0,00947454	-1,72	0,0110554	-1,77	0,999697	1,03	0,000101266	-2,31	0,266647	-1,31	0,14703	-1,34
ppa-miR-19a_st	0,00719835	2,85	0,00298537	3,36	0,996817	-1,18	0,0021221	3,41	0,987058	1,02	0,754109	1,20
ppa-miR-21_st	0,0030472	3,02	0,00396819	3,11	0,999697	-1,03	0,00316948	3,13	0,992966	1,01	0,964573	1,04
ppa-miR-224_st	0,00438966	1,94	0,0113308	1,90	0,999697	1,02	0,0184917	1,79	0,905099	-1,06	0,842772	-1,08
ppa-miR-30a-5p_st	0,0156304	1,81	0,0263758	1,82	0,999697	-1,01	0,0814614	1,62	0,785997	-1,12	0,759093	-1,12
ppa-miR-30b_st	0,00498732	2,97	0,000230014	4,35	0,894479	-1,46	0,0430805	2,39	0,160908	-1,82	0,677995	-1,25
ppt-miR1023c-5p_st	0,0030472	1,87	0,00151756	2,03	0,999697	-1,08	0,00188543	1,97	0,959079	-1,03	0,892752	1,05
ppt-miR1023d-5p_st	0,0309316	1,45	0,00492233	1,63	0,953262	-1,12	0,13087	1,35	0,350757	-1,21	0,768285	-1,08
ppt-miR894_st	0,00518867	2,75	0,00181058	3,28	0,990861	-1,19	0,0486181	2,22	0,40299	-1,48	0,658447	-1,24
ppt-miR901_st	0,0746435	1,69	0,00774775	2,13	0,921182	-1,26	0,00341569	2,23	0,939185	1,05	0,338655	1,32
ppy-let-7b_st	0,000183018	-1,44	6,06E-05	-1,51	0,990861	1,05	0,0956491	-1,22	0,0194297	1,24	0,0620809	1,19
ppy-miR-1275_st	0,00833961	-2,12	0,030033	-2,00	0,999697	-1,06	0,0971945	-1,73	0,768753	1,15	0,563986	1,22
ppy-miR-1292_st	0,00235759	2,43	0,00348794	2,46	0,999697	-1,01	0,00228308	2,51	0,977882	1,02	0,962814	1,03
ppy-miR-134_st	0,0164726	2,06	0,0218374	2,12	0,999697	-1,03	0,00169289	2,58	0,673285	1,22	0,526476	1,25
ppy-miR-140-5p_st	0,00328034	2,06	0,000275251	2,52	0,935558	-1,22	0,00729321	2,00	0,490618	-1,26	0,949752	-1,03
ppy-miR-148a_st	0,0708659	1,44	0,0263758	1,57	0,990861	-1,09	0,0395642	1,51	0,919234	-1,04	0,881891	1,05
ppy-miR-148b_st	0,00248618	1,94	0,00811613	1,87	0,999697	1,04	0,0016523	2,05	0,830634	1,09	0,899836	1,05
ppy-miR-181b_st	0,00581284	-1,74	0,00612964	-1,80	0,999697	1,03	7,02E-05	-2,32	0,284721	-1,29	0,151117	-1,33
ppy-miR-18b_st	0,1034	2,57	0,0391904	3,33	0,990861	-1,29	0,199513	2,30	0,633407	-1,45	0,904107	-1,12
ppy-miR-194_st	0,0220863	2,68	0,00442825	3,48	0,970327	-1,30	0,0898994	2,27	0,438271	-1,53	0,804385	-1,18
ppy-miR-19a_st	0,0141498	2,49	0,000290822	3,87	0,792664	-1,55	0,00906324	2,69	0,459373	-1,43	0,908233	1,08

ppy-miR-21_st	0,00427633	2,93	0,00222169	3,37	0,999697	-1,15	0,0017134	3,38	0,995331	1,01	0,813799	1,15
ppy-miR-29c_st	0,00105728	1,93	0,000194924	2,18	0,974307	-1,13	0,00108247	1,98	0,79269	-1,10	0,956113	1,02
ppy-miR-30b_st	0,193189	2,54	0,0176576	4,59	0,860533	-1,81	0,75932	1,43	0,0369921	-3,21	0,382209	-1,77
ppy-miR-30e_st	0,00106624	3,53	0,000223797	4,37	0,989324	-1,24	0,000576356	3,98	0,913597	-1,10	0,859394	1,13
ppy-miR-31_st	0,00099096	2,18	0,0123335	1,92	0,990861	1,14	0,000212156	2,49	0,384358	1,30	0,676821	1,14
ppy-miR-320d_st	0,162927	-1,30	0,0141176	-1,52	0,866176	1,17	0,00530517	-1,57	0,931479	-1,03	0,246779	-1,21
ppy-miR-370_st	0,00323027	1,89	0,00319808	1,96	0,999697	-1,04	0,00103275	2,08	0,89215	1,06	0,764941	1,10
ppy-miR-379_st	0,0316574	2,18	0,0110554	2,60	0,990861	-1,19	0,0294346	2,26	0,825804	-1,15	0,965373	1,04
ppy-miR-382_st	0,0124274	1,80	0,023843	1,79	0,999697	1,00	0,0030334	2,02	0,753605	1,13	0,71134	1,13
ppy-miR-423-5p_st	0,234774	-1,53	0,0363001	-1,97	0,92057	1,28	0,746718	-1,20	0,0882943	1,64	0,457761	1,28
ppy-miR-431_st	0,000755209	2,17	0,000261251	2,38	0,999697	-1,10	0,00319823	2,03	0,658064	-1,17	0,871325	-1,07
ppy-miR-451_st	0,0374537	2,03	0,0368075	2,15	0,999697	-1,06	0,0105703	2,35	0,892597	1,09	0,752455	1,16
ppy-miR-487b_st	0,0261765	2,86	0,0366642	2,96	0,999697	-1,03	0,012493	3,28	0,920441	1,11	0,861165	1,15
ppy-miR-503_st	0,119667	1,98	0,0116664	2,83	0,898605	-1,43	0,00478297	3,02	0,938721	1,07	0,300852	1,52
ptr-let-7b_st	2,95E-05	-1,53	1,81E-05	-1,58	0,999697	1,03	0,0338128	-1,27	0,0240487	1,25	0,0455239	1,20
ptr-miR-101_st	0,0286515	1,50	0,0324398	1,54	0,999697	-1,02	0,0532611	1,47	0,902176	-1,05	0,953687	-1,02
ptr-miR-1275_st	0,00248618	-2,26	0,0183487	-2,02	0,999697	-1,11	0,0505935	-1,80	0,81712	1,12	0,484591	1,25
ptr-miR-148a_st	1,42E-05	1,87	8,57E-06	1,97	0,999697	-1,05	8,82E-06	2,01	0,949105	1,02	0,73385	1,07
ptr-miR-152_st	0,0236825	2,25	0,0422461	2,25	0,999697	1,00	0,0119045	2,48	0,888885	1,11	0,872351	1,10
ptr-miR-181b_st	0,0102661	-1,71	0,0179256	-1,72	0,999697	1,00	0,000219759	-2,20	0,327497	-1,28	0,241434	-1,29
ptr-miR-18b_st	0,0527596	2,41	0,010482	3,22	0,953262	-1,34	0,14353	2,12	0,457455	-1,52	0,859027	-1,14
ptr-miR-19a_st	0,0598293	2,00	0,00396819	2,78	0,894479	-1,39	0,0298513	2,21	0,668113	-1,26	0,861017	1,11
ptr-miR-21_st	0,000358502	3,59	0,00145226	3,38	0,999697	1,06	0,000966855	3,45	0,981457	1,02	0,96362	-1,04
ptr-miR-210_st	0,00349863	1,92	0,0219223	1,78	0,999697	1,08	0,00465474	1,94	0,842969	1,09	0,989121	1,01
ptr-miR-29c_st	0,0341938	1,71	0,0231743	1,84	0,999697	-1,08	0,0503395	1,69	0,853423	-1,09	0,982579	-1,01
ptr-miR-301a_st	9,26E-06	1,80	4,54E-06	1,93	0,990861	-1,07	4,24E-07	2,05	0,785999	1,06	0,367481	1,14
ptr-miR-30b_st	0,0342802	2,71	0,00396819	3,89	0,935558	-1,43	0,295378	1,89	0,133971	-2,05	0,499271	-1,43
ptr-miR-30e_st	0,000269731	3,75	0,000133283	4,29	0,999697	-1,14	0,000300469	3,99	0,932373	-1,07	0,931994	1,07
ptr-miR-320d_st	0,229324	-1,31	0,0328931	-1,54	0,909919	1,17	0,0129613	-1,59	0,935768	-1,03	0,31822	-1,21
ptr-miR-370_st	0,0016504	2,06	0,00423878	2,01	0,999697	1,03	0,000476458	2,31	0,726972	1,15	0,742911	1,12
ptr-miR-376c_st	0,0252849	1,47	0,0154733	1,55	0,999697	-1,06	0,0197239	1,51	0,933274	-1,03	0,941347	1,02
ptr-miR-379_st	0,00382133	2,58	0,00245326	2,86	0,999697	-1,11	0,000826066	3,13	0,896409	1,09	0,666072	1,21
ptr-miR-382_st	0,00333533	2,49	0,0165088	2,29	0,999697	1,09	0,00117439	2,85	0,652603	1,24	0,777546	1,14
ptr-miR-431_st	0,00495794	2,38	0,0477943	2,03	0,990861	1,17	0,0120902	2,25	0,865747	1,11	0,930971	-1,05
ptr-miR-487b_st	0,00983392	3,54	0,0122256	3,74	0,999697	-1,06	0,00316228	4,38	0,870665	1,17	0,771815	1,24
ptr-miR-503_st	0,0850175	2,07	0,00238821	3,31	0,779466	-1,60	0,00324914	3,14	0,952692	-1,06	0,301993	1,52
ptr-miR-760_st	0,0111032	1,54	0,010482	1,59	0,999697	-1,03	0,0182459	1,52	0,896349	-1,05	0,974215	-1,01
ptr-miR-885_st	0,0148859	1,45	0,00145338	1,65	0,925823	-1,14	0,0040891	1,56	0,84833	-1,06	0,736185	1,08
rno-let-7b_st	9,26E-06	-1,54	5,51E-06	-1,59	0,999697	1,03	0,00890874	-1,30	0,0221366	1,22	0,0367196	1,19
rno-let-7e_st	0,00338479	-2,19	0,0444514	-1,87	0,974307	-1,17	0,184191	-1,57	0,687294	1,19	0,234009	1,39
rno-miR-106b-star_st	0,189982	-1,76	0,0448861	-2,25	0,953262	1,28	0,0318264	-2,26	0,994982	-1,01	0,563056	-1,29
rno-miR-140_st	0,0016504	1,94	0,00186516	1,99	0,999697	-1,03	0,00539191	1,84	0,838332	-1,08	0,892544	-1,05
rno-miR-142-5p_st	0,000214862	1,82	0,000344236	1,84	0,999697	-1,01	0,0002285	1,88	0,959079	1,02	0,921908	1,03
rno-miR-143_st	0,113251	2,60	0,0390677	3,45	0,98934	-1,33	0,0104538	4,05	0,880342	1,17	0,472828	1,56

rno-miR-148b-3p_st	0,0029939	2,01	0,00200222	2,15	0,999697	-1,07	0,00226944	2,10	0,970895	-1,02	0,923296	1,05
rno-miR-152_st	0,00851753	2,31	0,0168278	2,29	0,999697	1,01	0,010801	2,31	0,989997	1,01	0,997446	1,00
rno-miR-181b_st	0,00631747	-1,75	0,00876965	-1,78	0,999697	1,02	6,44E-05	-2,38	0,199973	-1,34	0,117137	-1,36
rno-miR-194_st	0,0142085	2,28	0,00312526	2,78	0,970567	-1,22	0,0565612	2,02	0,476328	-1,37	0,828348	-1,13
rno-miR-19a_st	0,000253703	3,22	1,35E-05	4,38	0,898605	-1,36	0,000216619	3,49	0,654834	-1,25	0,893457	1,08
rno-miR-21_st	0,000208803	3,40	0,000207197	3,64	0,999697	-1,07	0,0002285	3,62	0,993805	-1,01	0,927056	1,06
rno-miR-210_st	0,00224043	2,01	0,0191683	1,81	0,992747	1,11	0,00356873	2,00	0,818628	1,10	0,990312	-1,01
rno-miR-224_st	0,00296742	1,98	0,0183487	1,83	0,999697	1,08	0,00298297	2,03	0,795475	1,11	0,962814	1,03
rno-miR-29c_st	0,0248915	1,60	0,0245607	1,65	0,999697	-1,04	0,0193067	1,64	0,989885	-1,01	0,941563	1,03
rno-miR-301a_st	0,00369673	1,63	0,000720427	1,81	0,970567	-1,11	0,000939218	1,79	0,972268	-1,02	0,70443	1,09
rno-miR-30e_st	0,000589456	3,59	4,17E-05	4,86	0,935558	-1,36	0,000576356	3,80	0,680641	-1,28	0,938879	1,06
rno-miR-361_st	0,00951836	-1,48	0,0116664	-1,50	0,999697	1,02	0,00361719	-1,57	0,900385	-1,04	0,809287	-1,06
rno-miR-362_st	0,00328034	1,98	0,0110398	1,91	0,999697	1,04	0,00125688	2,17	0,741843	1,14	0,801929	1,10
rno-miR-370_st	0,00328034	2,04	0,00312526	2,14	0,999697	-1,05	0,00361719	2,09	0,97099	-1,02	0,968219	1,02
rno-miR-379_st	0,00478608	2,63	0,00235751	2,99	0,999697	-1,14	0,00289626	2,88	0,964654	-1,04	0,88147	1,10
rno-miR-382_st	0,00125874	2,80	0,00396819	2,65	0,999697	1,05	0,00252705	2,72	0,976867	1,02	0,967921	-1,03
rno-miR-409-3p_st	0,0311755	1,98	0,016368	2,23	0,999697	-1,12	0,00493202	2,41	0,903356	1,08	0,616772	1,22
rno-miR-431_st	0,00155492	2,37	0,00338537	2,33	0,999697	1,02	0,00205415	2,39	0,973204	1,02	0,990035	1,01
rno-miR-433_st	0,0304998	1,46	0,0289702	1,51	0,999697	-1,03	0,0326071	1,48	0,953948	-1,02	0,981843	1,01
rno-miR-487b_st	0,00248618	2,87	0,00273156	3,01	0,999697	-1,05	0,000548802	3,48	0,818098	1,16	0,683403	1,22
rno-miR-503_st	0,0339217	1,48	0,00761644	1,65	0,970567	-1,11	0,00301589	1,71	0,927752	1,04	0,481926	1,16
sja-miR-219-5p_st	0,063039	1,45	0,0230843	1,59	0,990861	-1,10	0,027318	1,55	0,944837	-1,03	0,835011	1,07
sla-miR-19a_st	0,0032642	3,31	0,00115344	3,99	0,991006	-1,21	0,000352175	4,49	0,888716	1,13	0,541211	1,36
sly-miR166a_st	0,00143776	1,87	0,00298537	1,85	0,999697	1,01	0,00354904	1,81	0,967089	-1,02	0,941984	-1,03
smo-miR166c_st	7,84E-05	1,95	0,00396819	1,68	0,925823	1,16	0,000491706	1,86	0,741003	1,10	0,869483	-1,05
snR38C_st	0,0198832	-1,48	0,0138317	-1,56	0,999697	1,05	0,294473	-1,27	0,2824	1,23	0,39112	1,17
spu-miR-92c_st	0,0315619	-1,65	0,0231743	-1,75	0,999697	1,06	0,00461048	-1,91	0,83219	-1,09	0,594142	-1,16
ssc-let-7e_st	0,00244788	-2,21	0,0285946	-1,91	0,985799	-1,16	0,182673	-1,55	0,599372	1,23	0,179435	1,42
ssc-miR-142_st	0,00186959	1,69	0,00155926	1,75	0,999697	-1,04	0,00158262	1,73	0,981761	-1,01	0,936	1,03
ssc-miR-148a_st	0,0142103	1,64	0,0269176	1,63	0,999697	1,00	0,0415347	1,56	0,914804	-1,05	0,888291	-1,05
ssc-miR-148b_st	0,00156153	1,87	0,00463444	1,81	0,999697	1,03	0,0102431	1,70	0,880309	-1,06	0,757146	-1,10
ssc-miR-15a_st	0,0896394	2,68	0,0166443	3,89	0,942473	-1,45	0,709022	1,44	0,0494948	-2,69	0,251913	-1,85
ssc-miR-181b_st	0,0122187	-1,78	0,0235678	-1,77	0,999697	-1,00	0,000333389	-2,31	0,342041	-1,30	0,278207	-1,30
ssc-miR-19a_st	0,000296585	3,76	2,90E-05	4,98	0,935558	-1,33	0,000107991	4,49	0,893787	-1,11	0,741313	1,20
ssc-miR-21_st	0,00371281	2,82	0,00219346	3,18	0,999697	-1,13	0,000811654	3,47	0,913813	1,09	0,667112	1,23
ssc-miR-24-star_st	0,00299811	2,53	0,00138708	2,87	0,999697	-1,13	0,014319	2,25	0,609212	-1,27	0,815782	-1,13
ssc-miR-29c_st	0,0265498	1,59	0,0167748	1,70	0,999697	-1,07	0,00761166	1,75	0,955171	1,03	0,753856	1,10
ssc-miR-30b-5p_st	0,239201	2,55	0,0477593	4,19	0,935558	-1,64	0,878656	1,26	0,0484212	-3,34	0,284956	-2,04
ssc-miR-423-5p_st	0,206087	-1,53	0,0388649	-1,89	0,935558	1,24	0,682305	-1,23	0,132605	1,54	0,490946	1,25
ssc-miR-503_st	0,000843362	1,93	0,00039636	2,06	0,999697	-1,07	7,02E-05	2,29	0,753935	1,11	0,449725	1,19
ssc-miR-885-3p_st	0,000183421	1,81	0,00231414	1,68	0,990861	1,08	0,000108115	1,92	0,584716	1,14	0,837638	1,06
ssc-miR-92b_st	0,00369673	-2,17	0,0187506	-2,01	0,999697	-1,08	0,00117577	-2,45	0,628163	-1,22	0,755918	-1,13
tgu-let-7b_st	9,26E-06	-1,57	8,23E-06	-1,61	0,999697	1,02	0,012649	-1,30	0,0236045	1,24	0,0321238	1,21
tgu-miR-140_st	2,44E-05	2,40	3,80E-05	2,45	0,999697	-1,02	0,000576356	2,12	0,662419	-1,16	0,649917	-1,14

tgu-miR-142_st	0,00248618	1,98	0,00303492	2,03	0,999697	-1,02	0,0174393	1,77	0,717311	-1,15	0,724608	-1,12
tgu-miR-148_st	0,000154364	1,71	0,000173318	1,74	0,999697	-1,02	3,69E-05	1,91	0,70532	1,09	0,532851	1,12
tgu-miR-15a_st	0,0985096	2,68	0,0444514	3,38	0,994144	-1,26	0,589699	1,62	0,214916	-2,08	0,40021	-1,65
tgu-miR-181b_st	0,00328034	-1,80	0,00865104	-1,76	0,999697	-1,02	7,02E-05	-2,32	0,229537	-1,32	0,207875	-1,29
tgu-miR-18b_st	0,0451049	2,17	0,00870739	2,78	0,954992	-1,28	0,0373565	2,28	0,749803	-1,22	0,949007	1,05
tgu-miR-194_st	0,0457118	2,24	0,0068934	2,98	0,935558	-1,33	0,263892	1,76	0,215589	-1,70	0,616168	-1,28
tgu-miR-199-5p_st	0,00427633	2,26	0,0477943	1,93	0,989158	1,17	0,00908088	2,18	0,81712	1,13	0,956184	-1,03
tgu-miR-21_st	0,000148971	3,37	6,60E-05	3,80	0,999697	-1,13	6,44E-05	3,99	0,949118	1,05	0,710458	1,18
tgu-miR-2954-star_st	0,0264012	1,76	0,0422461	1,78	0,999697	-1,01	0,0836815	1,64	0,865054	-1,09	0,857851	-1,08
tgu-miR-2982_st	0,125447	1,77	0,0497943	2,08	0,990861	-1,17	0,204079	1,68	0,680571	-1,23	0,936533	-1,05
tgu-miR-29a_st	0,0238406	1,85	0,0105316	2,06	0,99652	-1,12	0,0146952	1,96	0,928861	-1,05	0,904916	1,06
tgu-miR-301_st	0,000144472	1,92	0,000133283	1,98	0,999697	-1,03	0,000108115	2,01	0,973257	1,02	0,88072	1,05
tni-let-7b_st	9,26E-06	-1,56	8,57E-06	-1,59	0,999697	1,02	0,0176288	-1,28	0,0226864	1,24	0,0243164	1,21
tni-miR-101a_st	0,00581284	1,79	0,00345823	1,92	0,999697	-1,07	0,00419315	1,87	0,964331	-1,02	0,912851	1,04
tni-miR-140_st	0,00272173	2,07	0,00177504	2,23	0,999697	-1,07	0,00316676	2,11	0,921406	-1,06	0,974913	1,02
tni-miR-142a_st	0,000186064	1,78	0,000227708	1,82	0,999697	-1,02	0,000108115	1,88	0,920441	1,04	0,829589	1,06
tni-miR-181b_st	0,00149598	-1,95	0,000556966	-2,12	0,999697	1,09	8,82E-06	-2,75	0,302035	-1,30	0,0766375	-1,42
tni-miR-194_st	0,0109997	3,15	0,00302389	4,00	0,990516	-1,27	0,180841	2,12	0,197584	-1,89	0,438143	-1,49
tni-miR-19a_st	0,0251384	2,53	0,00146528	3,77	0,880138	-1,49	0,00719674	3,04	0,749803	-1,24	0,755973	1,20
tni-miR-21_st	0,00427633	1,56	0,00749138	1,57	0,999697	-1,00	0,00316676	1,61	0,932941	1,03	0,909249	1,03
tni-miR-210_st	0,00299811	1,89	0,0141957	1,78	0,999697	1,06	0,0200458	1,70	0,924415	-1,05	0,73886	-1,11
tni-miR-22b_st	0,00137931	2,18	0,0477943	1,76	0,92057	1,24	0,00244332	2,16	0,566925	1,23	0,981843	-1,01
U105B_st	0,00427633	-1,70	0,00811613	-1,70	0,999697	1,00	0,00345592	-1,76	0,935071	-1,03	0,923743	-1,04
U28_st	0,00377523	-1,99	0,0040682	-2,06	0,999697	1,04	0,202094	-1,48	0,218861	1,39	0,229515	1,35
U29_st	0,0130972	-1,44	0,00272562	-1,57	0,970567	1,09	0,00904498	-1,48	0,816291	1,06	0,91836	-1,03
U33_st	0,00230081	-1,69	0,00147599	-1,77	0,999697	1,05	0,00342215	-1,68	0,881716	1,05	0,996425	1,00
U41_st	2,96E-05	-2,30	0,000112322	-2,25	0,999697	-1,02	0,00117439	-1,97	0,684527	1,14	0,542754	1,16
U41_x_st	1,42E-05	-2,08	3,17E-05	-2,07	0,999697	-1,01	0,00419798	-1,66	0,25981	1,25	0,179435	1,26
U55_st	0,0459236	-1,51	0,0231743	-1,64	0,999697	1,08	0,308109	-1,31	0,36329	1,25	0,555418	1,15
U56_x_st	0,0100517	-1,73	0,0270611	-1,69	0,999697	-1,03	0,00396233	-1,87	0,774675	-1,11	0,816142	-1,08
U57_st	0,0147979	-1,58	0,0104685	-1,66	0,999697	1,05	0,118596	-1,41	0,514158	1,18	0,633306	1,12
U76_st	0,0125089	-1,70	0,0395947	-1,64	0,999697	-1,04	0,0289033	-1,64	0,999379	1,00	0,926168	1,04
U78_s_st	0,011182	-1,86	0,0421221	-1,76	0,999697	-1,06	0,0413892	-1,72	0,969941	1,02	0,847856	1,08
vvi-miR166h_st	0,15088	1,43	0,0183487	1,72	0,914635	-1,20	0,0511157	1,57	0,81432	-1,09	0,752873	1,10
vvi-miR394b_st	0,128051	1,48	0,0366349	1,70	0,970567	-1,14	0,0057825	1,88	0,793436	1,11	0,324652	1,27
xla-miR-142_st	0,00071448	1,61	0,0010494	1,63	0,999697	-1,01	0,000333389	1,70	0,887892	1,04	0,832235	1,05
xla-miR-194_st	0,0220692	2,49	0,0116025	2,89	0,999697	-1,16	0,271014	1,77	0,281603	-1,63	0,440518	-1,41
xtr-miR-101a_st	1,62E-05	2,21	8,67E-06	2,37	0,999697	-1,07	9,05E-07	2,69	0,668807	1,13	0,332115	1,22
xtr-miR-142-5p_st	1,62E-05	1,89	4,54E-06	2,11	0,937382	-1,12	6,20E-05	1,90	0,649308	-1,11	0,988782	1,01
xtr-miR-143_st	0,0282457	2,65	0,0183487	3,03	0,999697	-1,14	0,00130717	4,08	0,651749	1,35	0,363768	1,54
xtr-miR-148b_st	0,00444062	1,77	0,00531259	1,81	0,999697	-1,03	0,00546057	1,78	0,974515	-1,02	0,984493	1,01
xtr-miR-181b_st	0,00328034	-1,81	0,00183591	-1,94	0,999697	1,07	2,01E-05	-2,55	0,238449	-1,32	0,0648851	-1,41
xtr-miR-18b_st	0,0629195	2,48	0,0153511	3,30	0,970327	-1,33	0,225541	2,02	0,390696	-1,63	0,757521	-1,23
xtr-miR-194_st	0,0249586	2,63	0,0153511	3,02	0,999697	-1,15	0,172631	2,03	0,477635	-1,49	0,629402	-1,30

xtr-miR-19a_st	0,00359674	2,94	0,00062523	3,74	0,970327	-1,27	0,0016523	3,35	0,887861	-1,12	0,835011	1,14
xtr-miR-210_st	0,00536076	1,90	0,0371046	1,74	0,999697	1,09	0,0202407	1,77	0,974579	1,02	0,857667	-1,07
xtr-miR-27c_st	0,0130972	4,09	0,00396819	5,42	0,990861	-1,32	0,00602055	4,88	0,933396	-1,11	0,851777	1,19
xtr-miR-29a_st	0,0897077	1,81	0,0245607	2,17	0,970567	-1,20	0,0207687	2,14	0,983506	-1,02	0,691798	1,18
xtr-miR-301_st	0,00148105	1,71	0,00230831	1,73	0,999697	-1,01	0,00119316	1,77	0,953781	1,02	0,927423	1,03
xtr-miR-30a-5p_st	0,0183912	1,72	0,00345823	1,99	0,968841	-1,16	0,122458	1,51	0,311191	-1,32	0,671648	-1,14
xtr-miR-30e_st	0,000755209	3,46	0,000194924	4,17	0,990861	-1,20	0,00134917	3,41	0,753605	-1,22	0,988226	-1,01
xtr-miR-34a_st	0,351498	1,67	0,0283457	2,68	0,801344	-1,60	0,041054	2,46	0,923837	-1,09	0,398321	1,47
xtr-miR-92b_st	0,0261082	-2,47	0,0149001	-2,83	0,999697	1,15	0,00359385	-3,23	0,864628	-1,14	0,577816	-1,31

Abbreviations: q, q-value; FC, Fold Change
