# Supplementary material for: CALR mutational status identifies different disease subtypes of essential thrombocythemia showing distinct expression profiles
Source: Blood Cancer J. 2017 Dec 8;7(12):638. doi: 10.1038/s41408-017-0010-2 (PMC5802509; doi:10.1038/s41408-017-0010-2)
Supplement: Supplementary file 4 [file 41408_2017_10_MOESM6_ESM.docx]

Table S3. Clinical correlations between hematological parameters and gene expression

Gene Symbols	Hematological parameter	r	q
TWSG1	Hemoglobin	0.340673	0.0196015
PDCD4	Hemoglobin	0.316108	0.0306902
ZNF148	Hemoglobin	0.306396	0.036323
PLK3	Platelet count	0.476858	0.000641002
WIPF1	Platelet count	0.401863	0.00455495
ZNF148	Platelet count	-0.519318	0.000172891
IFNAR1	Platelet count	-0.413861	0.00341981
PTEN	Platelet count	-0.408181	0.00392143
SOCS6	Platelet count	-0.387054	0.00640281
